# Optimized conditions for *Listeria*, *Salmonella* and *Escherichia* whole genome sequencing using the Illumina iSeq100 platform with point-and-click bioinformatic analysis

**DOI:** 10.1371/journal.pone.0277659

**Published:** 2022-11-30

**Authors:** Sonsiray Alvarez Narvaez, Zhenyu Shen, Lifang Yan, Brianna L. S. Stenger, Laura B. Goodman, Ailam Lim, Ruth H. Nissly, Meera Surendran Nair, Shuping Zhang, Susan Sanchez

**Affiliations:** 1 Department of Population Health, College of Veterinary Medicine, The University of Georgia, Athens, Georgia, United States of America; 2 Department of Veterinary Pathology, College of Veterinary Medicine, University of Missouri, Columbia, Missouri, United States of America; 3 Department of Pathobiology and Population Medicine, College of Veterinary Medicine, Mississippi State University, Starkville, Mississippi, United States of America; 4 Veterinary Diagnostic Laboratory, North Dakota Agricultural Experiment Station, North Dakota State University, Fargo, North Dakota, United States of America; 5 Department of Public & Ecosystem Health, College of Veterinary Medicine, Cornell University, Ithaca, New York, United States of America; 6 Wisconsin Veterinary Diagnostic Laboratory, University of Wisconsin-Madison, Madison, Wisconsin, United States of America; 7 Department of Veterinary and Biomedical Sciences, College of Agricultural Sciences, PennState University, State College, Pennsylvania, United States of America; 8 Department of Veterinary Pathobiology, Veterinary Medical Diagnostic Laboratory, College of Veterinary Medicine, University of Missouri, Columbia, Missouri, United States of America; 9 Department of Infectious Disease, College of Veterinary Medicine, The University of Georgia, Athens, Georgia, United States of America; University of Minnesota, UNITED STATES

## Abstract

Whole-genome sequencing (WGS) data have become an integral component of public health investigations and clinical diagnostics. Still, many veterinary diagnostic laboratories cannot afford to implement next generation sequencing (NGS) due to its high cost and the lack of bioinformatic knowledge of the personnel to analyze NGS data. Trying to overcome these problems, and make NGS accessible to every diagnostic laboratory, thirteen veterinary diagnostic laboratories across the United States (US) initiated the assessment of Illumina iSeq100 sequencing platform for whole genome sequencing of important zoonotic foodborne pathogens *Escherichia coli*, *Listeria monocytogenes*, and *Salmonella enterica*. The work presented in this manuscript is a continuation of this multi-laboratory effort. Here, seven AAVLD accredited diagnostic laboratories explored a further reduction in sequencing costs and the usage of user-friendly platforms for genomic data analysis. Our investigation showed that the same genomic library quality could be achieved by using a quarter of the recommended reagent volume and, therefore a fraction of the actual price, and confirmed that Illumina iSeq100 is the most affordable sequencing technology for laboratories with low WGS demand. Furthermore, we prepared step-by-step protocols for genomic data analysis in three popular user-friendly software (BaseSpace, Geneious, and GalaxyTrakr), and we compared the outcomes in terms of genome assembly quality, and species and antimicrobial resistance gene (AMR) identification. No significant differences were found in assembly quality, and the three analysis methods could identify the target bacteria species. However, antimicrobial resistance genes were only identified using BaseSpace and GalaxyTrakr; and GalaxyTrakr was the best tool for this task.

## Background

Whole-genome sequencing (WGS) has revolutionized the study and diagnosis of infectious diseases [1]. Currently, an increasing number of food, public, and animal health testing laboratories apply next-generation sequencing (NGS) to obtain the complete genetic information of microbial isolates of interest [2]. Similarly, WGS has been used for disease surveillance allowing a more rapid and accurate outbreak detection and source attribution like in the case of the SARS-CoV-2 pandemic [3–5], and antimicrobial resistance (AMR) monitoring is important in pathogens in human and veterinary medicine [6–8]. However, NGS technology is still inaccessible for many small diagnostic laboratories, especially in the field of veterinary medicine, due to the high costs associated with acquiring and implementing this technology and the lack of personnel with bioinformatics experience to analyze NGS data.

There are currently two main NGS approaches utilized for the sequencing of microbial whole genomes: (i) short-read sequencing which delivers the genomic content of a particular organism in short reads or DNA fragments of 75–400 bp length; and (ii) long-read sequencing that provides the genome information in longer reads, generally above 10,000 bps [9]. Illumina is the short-read technology most widely used, while PacBio Single Molecule Real Time (SMRT) and Oxford Nanopore are the most popular long-read sequencing alternatives. In 2018, the US FDA through the Veterinary Laboratory Investigation and Response Network (Vet-LIRN) piloted the implementation of the Illumina iSeq 100 sequencing platform in veterinary diagnostic laboratories with lower throughput needs [10]. By 2019, 13 out of the 46Vet-LIRN laboratories standardized a common library preparation protocol using Illumina iSeq100 technologies [10] for the whole-genome sequencing of three important animal and human pathogens (*Escherichia coli* [*E*. *coli*], *Listeria monocytogenes* [*L*. *monocytogenes*] and *Salmonella enterica* [*S*. *enterica*]). Furthermore, this multi-laboratory effort translated into a detailed, step-by-step protocol publicly available via protocols.io (https://dx.doi.org/10.17504/protocols.io.6qpvr44rbgmk/v1); the optimization of the maximum number of isolates that can be included per sequencing run to obtain an acceptable genome coverage; and the evidence that the iSeq100 chemistry and the Illumina DNA Prep library preparation kit is sufficient to produce quality data for antimicrobial resistance surveillance from bacterial isolates [10].

The work contained in this manuscript goes one step beyond in bringing NGS implementation closer to veterinary diagnostic laboratories. We further tested the impact of reducing reagent volume in genomic library quality and subsequent results to further minimize sequencing costs. Additionally, this project details and compares four different pipelines using both the command-line interface (CLI) and user-friendly resources to analyze Illumina iSeq 100 WGS data for bacterial species and AMR identification to overcome the potential lack of bioinformatics experience of involved laboratory personnel.

## Methods

### Bacterial isolates

Two *L*. *monocytogenes*, two S. *enterica* ser. Typhimurium and one *E*. *coli* isolate were used in this study (Table 1), and include the same isolates used in the original Vet-LIRN collaborative project [10]. Frozen isolates were sub-cultured twice on Trypticase Soy + 5% Sheep Blood Agar plates (BAPs) or equivalent media prior to DNA extraction.

**Table 1:** Bacterial isolates and reagent volumes used to analyze the data.

Run	Bacteria	NCBI accession #	Reagent Volume
1	*Listeria monocytogenes* 21	SAMN03015673	1 0.5 0.25
	*Listeria monocytogenes* 22	SAMN02922921	1 0.5 0.25
2	*Escherichia coli* 16	SAMN10221118	1 0.5 0.25
3	*Salmonella enterica* ser. Dublin 1	SAMN08868674	1 0.5
	*Salmonella enterica* ser. Dublin 2	SAMN08868685	1 0.5
4	*Listeria monocytogenes* 21	SAMN03015673	0.25
	*Escherichia coli* 16	SAMN10221118	
	*Salmonella enterica* ser. Dublin 1	SAMN08868674	
	*Salmonella enterica* ser. Dublin 2	SAMN08868685	

### Laboratory procedures

Bacterial DNA extraction and library prep were carried out following the original protocol (https://dx.doi.org/10.17504/protocols.io.bij8kcrw), using original reagent volumes (X 1) and reducing the reagent volumes to half (X 0.5) or to a quarter (X 0.25) as specified in Table 1. A step-by-step protocol including the optimized reagent volume reduced to a quarter can be found on https://dx.doi.org/10.17504/protocols.io.6qpvr44rbgmk/v1 and it is included for printing as S4 File with this article. Briefly, bacterial genomic DNA was extracted using either the DNeasy Blood & Tissue Kit (Qiagen) or the MagMAX CORE automated extraction kit (Thermo Fisher). The concentration of purified DNA was measured by Qubit fluorometry (Thermo Fisher). Barcoded sequencing libraries were prepared using the DNA Prep kit (Illumina). Sequencing was performed using iSeq100 2 × 150 bp chemistry (Illumina). First, the three bacteria species were run in separate runs: two *L*. *monocytogenes* under three library prep conditions (6 samples) were sequenced in run 1; one *E*. *coli* under three library prep conditions (3 samples) was sequenced in run 2, and the two S. *enterica* under two library prep conditions (4 samples) were sequenced in run 3. A 4^th^ mixed run with one *L*. *monocytogenes*, one *E*. *coli*, and two S. *enterica* using a quarter of the recommended reagents was performed (Table 1).

### WGS data analysis

**Fig 1:**
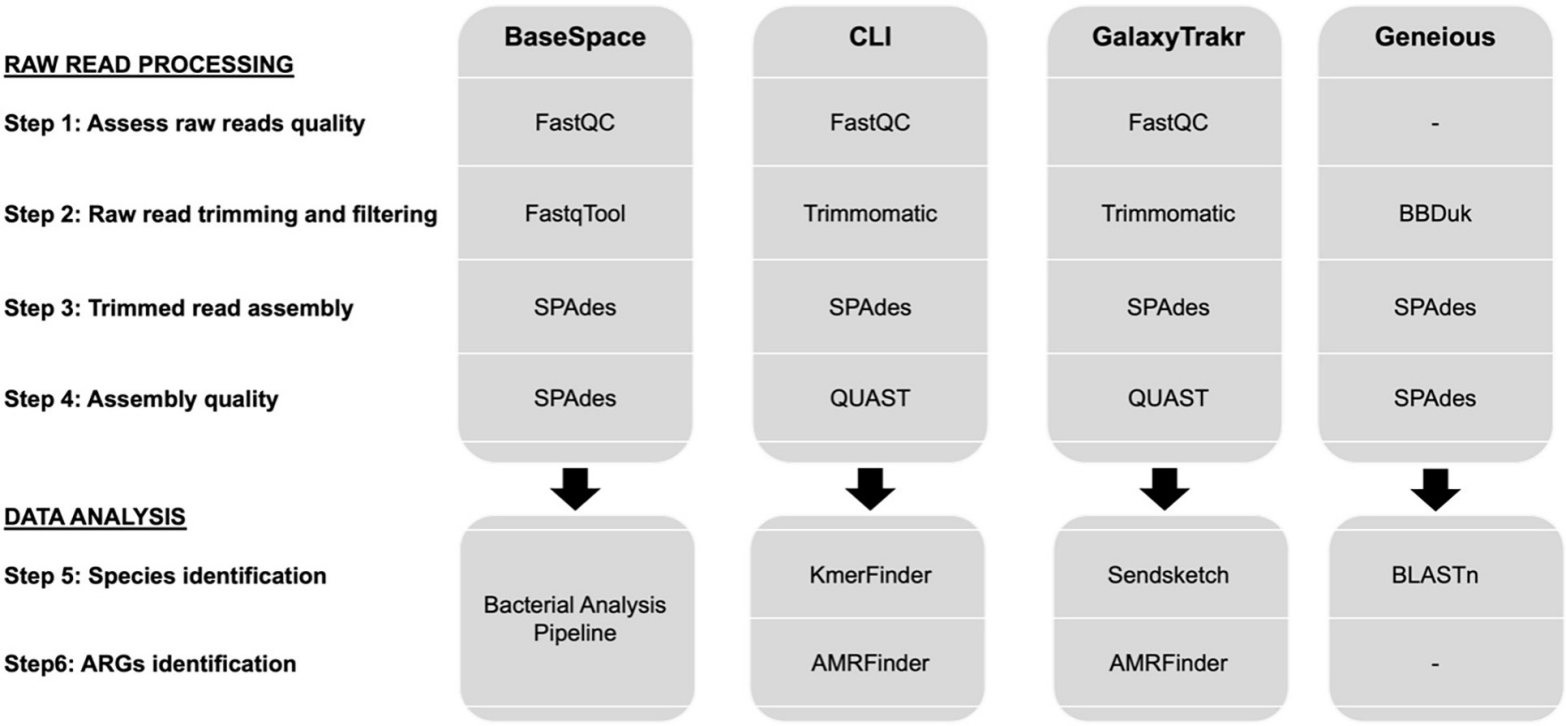
Summary of the steps and software used for data analysis in each bioinformatics platform.

Four platforms—BaseSpace, GalaxyTrakr, Geneious, and Command Line Interface (CLI)—were used to perform read trimming and assembly (Fig 1). A step-by-step protocol for each platform can be found in S1–S3 Files. In BaseSpace, raw reads from each run were quality checked using FastQC (v.1.0.0, BaseSpace Illumina) and subsequently trimmed and quality filtered using FastqTool (v.2.2.5, BaseSpace Illumina). Genome *de novo* assembly and assembly quality were performed using SPAdes (v.3.9.0, BaseSpace Illumina). BaseSpace Bacterial Analysis Pipeline was used for species determination and antimicrobial resistance genes (ARGs) identification (v.1.0.4, BaseSpace Illumina). In GalaxyTrakr and CLI, raw reads were quality checked using FastQC [11] (CLI Version 0.11.5; Galaxy Version 0.73+galaxy) and subsequently trimmed and quality filtered using Trimmomatic [12] (CLI Version 0.36; Galaxy Version 0.38.1). Genome *de novo* assembly was also performed using SPAdes [13] (CLI Version 3.11.1; Galaxy Version 3.12.0+galaxy1) and assembly quality was checked with QUAST [14] (CLI Version 5.0.0; Galaxy Version 5.0.2+galaxy1). Species identification was carried out using KmerFinder [15] (CLI Version 3.0.2; Galaxy Version 3.0.2+galaxy0), and ARGs were identified with AMRFinder in both (CLI version 3.9.8; Galaxy Version 3.8.28+galaxy1). In Geneious, there is no tool to look at the read quality, hence raw reads were directly trimmed, and quality filtered using BBDuk Trimmer (version 1.0, Biomatters Ltd.). Genome *de novo* assembly and assembly quality were done using SPAdes [13] (version 3.15.2), and species identification was performed using BLAST.

### Statistical analysis

Linear Mixed-Effects Models in statistical software GraphPad Prism 6.0 (La Jolla, USA) were used to determine significant differences in the library prep process, read recovery, and genome coverage using different reagent volumes, as well as to assess differences in assembly quality metrics between the different platforms.

### Public data submission

Sequencing data generated as part of this project is deposited in GenBank under PRJNA834767.

## Results

### Minimizing reagent usage for library preparation

With the main goal of making NGS-based diagnosis of infectious diseases accessible to most veterinary diagnostic laboratories, we first investigated the costs associated with sequencing reagents, instrument acquisition, and maintenance for three Illumina sequencing platforms: iSeq100, MiSeq, and NextSeq1000 (Table 2). Our calculations showed that NextSeq1000 had the highest machine acquisition and maintenance cost, while iSeq100 had the lowest. The reagent cost per sample was found to be the same independently of the platform or cartridge used because the Nextera XT DNA Library Preparation Kit is compatible with all Illumina sequencers (Table 2A). These high differences in sequencing associated costs (Table 2B) reside in the fact that Miseq (using the v2 300 cycles cartridge) and Nextseq1000 can sequence substantially more isolates in the same cartridge than iSeq100 or the smaller MiSeq cartridges and still reach a genome coverage of 50X (Table 2A). However, the iSeq100 platform seemed to be the most suitable option for small veterinary diagnostic laboratories because this machine and its maintenance are five times cheaper than MiSeq and ten times cheaper than NextSeq1000, and run times are comparable. The major limitation of iSeq100; however, is the fact that it can sequence only up to six bacterial genomes with a 50X coverage while a run of MiSeq could sequence up to 36 *Listeria* isolates at once (Table 2A).

**Table 2:** An overview of the cost of using iSeq100, MiSeq, and NextSeq1000 as a diagnostic tool including, expenses associated with instrument acquisition and maintenance (A), and cost-cutting associated with reagent reduction during library prep for iSeq100 (B).

A									
Instrument	Instrument cost	Maintenance cost per year*^2^	Cartridge	Cartridge cost*^1^	Reagent cost*/sample	Million reads per run*^3^	Run time	Listeria genomes/run	*E*. *coli*/Salmonella genomes/run
iSeq100	$19,900	$3,750	iSeq100 v2 (300 cycles)	$574	$49	4	~19h	~ 6	~ 4
MiSeq	$99,000	$14,070	Miseq v2 (300 cycles)	$1,194		24	~24h	~ 25	~ 15
			Miseq v2 Micro (300 cycles)	$502		8	~19h	~9	~5
			Miseq v2 Nano (300 cycles)	$333		2	~19h	~2	~1
NextSeq	$210,000	$28,000	NextSeq 1000/2000 P1 (300 Cycles)	$1,250		200	~19h	~215	~120

B												
	Cost per sample*^4^											
	1 sample			2 samples			4 samples			6 samples		
	vol. X 1	vol. X 0.5	vol. X 0.25	vol. X 1	vol. X 0.5	vol. X 0.25	vol. X 1	vol. X 0.5	vol. X 0.25	vol. X 1	vol. X 0.5	vol. X 0.25
iSeq100	$623	$598	$586	$336	$311	$299	$192	$168	$156	$145	$120	$108
MiSeq Micro	$551	$526	$514	$300	$275	$263	$174	$150	$138	$133	$108	$96
MiSeq Nano	$382	$357	$345	$215	$191	$179						
Miseq	$1,243	$1,218	$1,206	$646	$621	$609	$347	$323	$311	$248	$223	$211
NextSeq	$1,299	$1,274	$1,262	$674	$649	$637	$361	$337	$337	$257	$233	$233

Our calculations were based on prices available at https://www.illumina.com, last accession May 2022.

*Price includes Nextera XT DNA Library Preparation Kit and Nextera™ DNA CD Indexes for 24 samples.

*^1^ Cartridge iSeq100 v2 (300 cycles), Miseq v2 (300 cycles), NextSeq 1000/2000 P1 Reagents (300 Cycles).

*^2^ Advanced exchange silver plan applies to iSeq100. Silver support plan applies to MiSeq and NextSeq1000.

*^3^ For Illumina iSeq100 calculation based on 4 million 150 bp paired-end reads passing filter. For Illumina MiSeq calculation based on 24 million 150 bp paired-end reads passing filter. For Illumina NextSeq1000 calculation based on 200 million 150 bp paired-end reads passing filter.

*^4^ Including Illumina® DNA Prep kit, Nextera™ DNA CD Indexes for 24 samples and cartridges iSeq100 v2 (300 cycles), Miseq v2 Micro (300 cycles), Miseq v2 Nano (300 cycles), Miseq v2 (300 cycles) and NextSeq 1000/2000 P1 (300 Cycles). Our calculations were based on prices available at https://www.illumina.com, last accession May 2022.

Trying to reduce sequencing costs even more, we investigated the impact of using reduced volumes of genomic library prep reagents on sequencing performance and read recovery using Illumina sequencing technology (Table 2B). We decided to compare the outcomes in terms of the number of clusters generated in the sequencing machine, the number of reads obtained in each run, and genome coverage using the manufacturer’s recommended reagent volume (X1), versus half volume (X0.5) and a quarter volume (X0.25) for the sequencing of foodborne pathogens *E*. *coli* (n = 1), *L*. *monocytogenes* (n = 2), and *S*. *enterica* ser. Typhimurium (n = 2). Initial bacterial DNA used for library prep was adjusted to each reagent condition, and 300 ng, 150 ng, and 75 ng of DNA were used with X1, X0.5, and X0.25 reagent volumes, respectively. We observed that a reduction in the reagents and initial DNA used to prepare the genomic libraries prior to sequencing translated into a decreased genomic library concentration. The utilization of the manufacturer’s recommended volumes yielded library concentrations of ~80 nM depending on the bacteria species. In contrast, half (~35 nM) and a quarter (~18 nM) of these concentrations were obtained when using half and a quarter of the recommended values respectively (Table 3). Nevertheless, all library concentrations showed values higher than the minimum 1 nM recommended by the manufacturer to be used for subsequent steps of the sequencing process. Additionally, there were no significant differences in the number of sequencing clusters, the number of reads produced, or genome coverage between the different reagent volumes (Table 3), evidencing that as little as a quarter of the manufacturer’s recommended volume yields comparable sequencing performance.

**Table 3:** Impact of reducing initial DNA and reagent usage during library prep on read recovery and genome coverage.

*All Mean*	X1 Reagent vol. (n = 5)	X 0.5 Reagent vol. (n = 6)	X 0.25 Reagent vol. (n = 6)	P-value
Starting DNA concentration (ng/μl)	10	10	10	-
Starting volume (μl)	30	15	7,5	-
Library concentration before pooling (mean±SEM ng/μl)	31.5 ± 7.6	13.9 ± 3.5	7.7 ± 2.2	0.0094**
Library concentration before pooling (mean±SEM nM)	79.5 ± 19.3	35.2 ± 8.8	18.0 ± 5.6	0.0093**
Desired library concentration after pooling (nM)	4	4	4	-
Number of clusters (mean±SEM x10^6^)	0.90 ± .013	0.92 ± .013	1.1 ± .20	0.4457
Read Count (mean±SEM x10^6^)	1.8 ± .26	1.8 ± .25	2.2 ± .41	0.4457
Genome Coverage	65.2 ± 2.7	68.6 ± 5.5	73.0 ± 9.1	0.705
Read Length (bp)	151	151	151	-

** Statistical significance.

We calculated the cost-cutting associated with reagent reduction during library prep for iSeq100 and MiSeq systems (Table 2B). Both Illumina instruments require the same library prep kit. Even when reagent reduction was applied to MiSeq cost, iSeq100 appeared to be the most affordable option when a small number of samples was run (Table 2). However, increasing the number of samples per run in the MiSeq decreases, in turn, the sequencing cost per sample to $70 and $54 when sequencing 24 or 36 samples, respectively, using a quarter of the recommended reagent volume.

### Four alternative pipelines for WGS data analysis from raw reads to contigs

Another bottleneck small veterinary diagnostic laboratories may face during the introduction of WGS as a diagnostic tool is the lack of bioinformatics experience to analyze sequencing data. We tested the performance of three user-friendly platforms to analyze NGS data: GalaxyTrakr [16], BaseSpace (Illumina), and Geneious (Geneious Prime, https://www.geneious.com). Furthermore, we have compared the capabilities of these three online alternatives under default settings (see Materials and Methods) on read filtering and assembly to what was obtained by using an in-house bioinformatics pipeline in the command-line interface (CLI). Fig 1 summarizes the main steps followed for raw read processing. Individualized step-by-step protocols for each platform are available in S1–S3 Files. First, we looked at the programs available in each platform for raw sequence data processing (Table 4). The same programs were used in the different platforms, when possible, to have more comparable results (Fig 1). Except for Geneious, all platforms had a program such as FastQC [11] to visualize the quality of the raw reads prior to starting data processing. Furthermore, both BaseSpace and Geneious are missing a program to determine the quality of the read assembly. However, for both platforms de novo assembler SPAdes [13] provides an indicative table with the most common assembly quality scores (example in S1 and S2 Files).

**Table 4:** List of some available programs and tools in each user-friendly platform.

	BaseSpace	GalaxyTrakr	Geneious
Read quality checking	***FastQC***	***FastQC***	-
	DRAGEN FastQC + MultiQC	MultiQC	-
Read trimming and Filtering	***FastQ Tool Kit***	Cutadapt	***BBDuk Trimmer***
		Dada2	
		***Trimomatic***	
		FASTX toolkit	
		Fastp	
		Trim Galore!	
Assembly de novo	***SPAdes*** Velvet	ABySS	MIRA Tadpole ***SPAdes*** Velvet
		Megahit	
		Minia	
		MIRA	
		Skesa	
		***SPAdes***	
		Shovil	
		Velvet	
Assembly quality checking	***SPAdes***	***Quast***	***SPAdes***
Species identification	***Bacterial Analysis Pipeline*** (KmerFinder)*	***KmerFinder***	***BLASTn***
ARGs identification	***Bacterial Analysis Pipeline*** (ResFinder)	hAMRonize	-
		***AMRFinder***	-
		ABRicate	-
		Staramr	-
MLST	***Bacterial Analysis Pipeline*** (MLST)	***MLST***	-
		MentaList	-
		strinMLST	-
Serotype	-	***SISTR***	-

In bold and italics programs selected to analyze the data in this study.

*Bacterial Analysis Pipeline is composed by five programs. In brackets, specific program used in every identification step.

**Fig 2:**
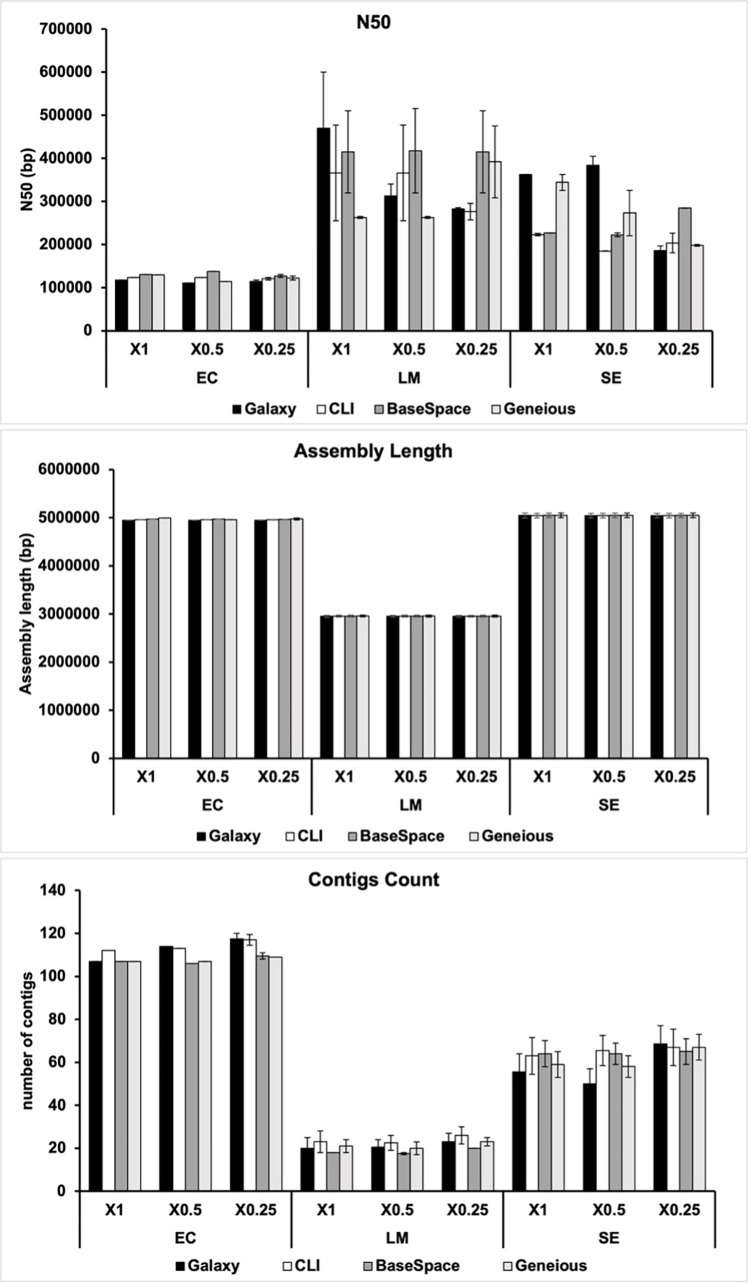
Comparing the quality of genome assemblies for *E*. *coli* (EC), *L*. *monocytogenes* (LM), and *S*. *enterica* (SE) sequencing data obtained with different reagent volumes (X1, X0.5, and X 0.25) and analyzed with different platforms (GalaxyTrakr, CLI, BaseSpace and Geneious). Statistical significance was assessed using the linear mixed model in software GraphPad Prism9 for Mac (v. 9.3.1, GraphPad Software, San Diego, California USA, www.graphpad.com).

No significant differences in assembly quality were found when comparing the libraries prepared with different reagent volumes (Fig 2). Similarly, there were no significant differences between platforms regarding the number of contigs obtained after assembly, the assembly length, or the assembly quality score N50 (Fig 2). These results further support that a quarter of the recommended reagent volumes is enough to prepare genomic libraries and evidence that any of the three platforms can be used indistinctly for raw read data processing.

### Identifying species and AMR genes

We reviewed the capacity of the three user-friendly platforms to pursue genome analysis that will help in the diagnostic process, such as species recognition, serotype and sequence type determination, and antimicrobial resistance gene (ARG) identification. Program options for each platform were identified (Table 4). Geneious did not have any already installed or plugin program among its tools to carry out the tasks mentioned above, but we used the popular alignment tool BLASTn for species identification. BaseSpace is equipped with a “Bacterial Analysis Pipeline” that claims to be able to predict the species of bacterial input genomes (using KmerFinder [15, 17]), identifying ARGs (with ResFinder [18, 19]), and, depending on the identified species (only for *Enterobacteriaceae*), performing a Multilocus Sequence Typing (MLST) classification, as well as a plasmid and virulence factor recognition. GalaxyTrakr appeared to be the best-equipped platform with a large selection of programs, including Sendsketch [20] for bacteria species determination, AMRFinder [21] for identifying AMR genes, and SISTR for serotyping.

All species tested (*E*. *coli*, *L*. *monocytogenes*, and *S*. *enterica*) were identified regardless of the reagent volume used during library prep or the bioinformatics platform that analyzed the data. Then, we looked at differences in the antimicrobial resistance gene profiles of the tested bacteria identified by the different bioinformatic approaches, and we discovered that BaseSpace failed to identify one of the *E*. *coli* ARGs recognized by GalaxyTrakr and the in-house CLI pipeline. All the ARGs were identified in *L*. *monocytogenes* (Table 5). Furthermore, GalaxyTrakr was the only platform that predicted bacteria serotypes.

**Table 5:** Antimicrobial resistance genetic profile identified by user-friendly platforms (GalaxyTrakr and BaseSpace), and an inhouse pipeline in the command line interface (CLI).

*E*. *coli 16*			*L*. *monocytogenes 21*			*S*. *enterica* ser. Dublin 1		
GalaxyTrakr	CLI	BaseSpace	GalaxyTrakr	CLI	BaseSpace	GalaxyTrakr	CLI	BaseSpace
blaHER-3	blaHER-4	blaHER-3	lin	lin		aadA12	aadA12	aadA12
tet(A)	tet(A)	tet(A)	fosX	fosX		qacEdelta1	qacEdelta1	
blaEC	blaEC					sul1	sul1	sul1
sul2	sul3	sul2				aph(3’)-Ia	aph(3’)-Ia	aph(3’)-Ia
qnrB19	qnrB20	qnrB19				tet(A)	tet(A)	tet(A)
						aph(6)-Id	aph(6)-Id	strB
						aph(3’’)-Ib	aph(3’’)-Ib	strA
						sul2	sul2	sul2
						blaCMY-2	blaCMY-2	blaCMY-2
						blaTEM-1	blaTEM-1	blaTEM-1B
						floR	floR	floR
			*L*. *monocytogenes 22*			*S*. *enterica ser*. Dublin 2		
			**GalaxyTrakr**	**CLI**	**BaseSpace**	**GalaxyTrakr**	**CLI**	**BaseSpace**
			lin	lin		aph(3’)-Ia	aph(3’)-Ia	aph(3’)-Ia
			fosX	fosX		tet(A)	tet(A)	tet(A)
						aph(6)-Id	aph(6)-Id	strB
						aph(3’’)-Ib	aph(3’’)-Ib	strA
						sul2	sul2	sul2
						blaTEM-1	blaTEM-1	blaTEM-1B
						floR	floR	floR

## Discussion

The work included in this manuscript is the continuation of a previous effort to make NGS available for all veterinary diagnostic laboratories [10]. Our first goal in this project was to determine the NGS short- read technology that would better fit the needs of a small/medium veterinary diagnostic laboratory like the Athens Veterinary Diagnostic Laboratory (ADVL) from the University of Georgia. ADVL receives around three to five cases a week that would require further species identification using non-sequencing methods, such as biochemical tests or MALDI-TOF. Hence, if WGS were used as a diagnostic tool instead, the perfect sequencing platform would allow the sequencing of up to five bacterial genomes per run. We compared the cost of acquiring, maintaining, and running the three most popular Illumina platforms iSeq100, MiSeq, and NextSeq1000. Although MiSeq, and NextSeq1000 sequencing platforms offer the potential to sequence up to 25 (MiSeq) and 200 (NextSeq1000) bacterial genomes in one single run, the upfront investment needed to implement MiSeq or NextSeq1000 sequencing is five to ten times higher than iSeq100. With higher number of samples, MiSeq and NextSeq1000 represent a more affordable option with an estimated cost per sample of $15–54 (using 96-sample library prep kits and a full cartridge), but the per-sample cost doubles when sequencing only six or fewer isolates. Therefore, we concluded that iSeq100 was the most suitable Illumina sequencing platform for laboratories with a small/medium throughput. We are aware that long-read sequencing platforms such as Oxford Nanopore MinION and Flongle adapter are becoming a very popular alternative to Illumina for bacteria WGS due to the $0 instrument acquisition and maintenance cost (Oxford Nanopore starting package includes a MinION sequencer with the first library prep kit ordered, https://store.nanoporetech.com/us/devices.html). Due to the different bioinformatics analysis tools used for long read sequencing, we did not compare the outcomes obtained from the Illumina platforms with this technology. Future work will focus on performing such comparisons, especially since recent evidence showed that several bacterial isolates can be multiplexed and sequenced simultaneously in the same MinION run with high genome coverage [22].

Genomic library preparation is the costliest step for the whole-genome sequencing of microbial isolates. That pushed several research groups to try producing genomic libraries using reduced reagent volumes to lower the cost [23, 24]. In the attempt to make NGS an affordable process for all veterinary diagnostic labs, we investigated if a reduction of the recommended bacterial DNA input for genomic library preparation with a subsequent decrease of reagent volume would impact sequencing performance and outcome data quality. Illumina recommends 4 nM as the starting genomic library concentration to reach the optimal applicable loading concentration [25]. If the concentration of the genomic library obtained is above 4 nM, the library must be diluted to 1 nM to continue with flow cell loading steps (denaturation and further dilution to 100–200 pM in loading buffer) [25]. Hence, producing genomic libraries with a concentration above 1 nM is a waste of library prep reagents and bacterial DNA. Our results showed that even when a quarter of the DNA and reagent volume recommended by Illumina was used, the genomic libraries obtained were at least three times more concentrated than the 1 nM threshold. Subsequently, we did not see any significant effect on sequencing performance, read recovery, and genome coverage (Table 3).

Besides the elevated costs, the lack of bioinformatics knowledge among lab personnel is another bottleneck that hinders NGS implementation in veterinary diagnostic laboratories [26]. We prepared step-by-step protocols for three well-known user-friendly bioinformatic software platforms—Illumina BaseSpace, GalaxyTrakr, and Geneious—and we tested them with the genomic data obtained in the four sequencing runs performed in this study. No significant differences in genome assembly quality were observed using different bioinformatics platforms (Fig 2). Similarly, all targeted species were identified regardless of the software platform used. Hence, if bacteria species identification is the final goal, any of the platforms used in this study can be used.

Geneious was designed for research purposes to assist researchers working with omics data and lacking solid bioinformatics knowledge. It is equipped with an extensive list of programs to analyze genomic, transcriptomic, and proteomic data (https://www.geneious.com). However, Geneious lacks critical programs for diagnostic purposes, such as software for bacterial species determination and ARG recognition. Geneious runs locally on computers that activate the annual license. This is a good thing when the data analysis does not require a lot of computational resources (like when analyzing small bacterial or viral genomes) because the user does not have to face the typical waiting times that happen when sending the analysis remotely to a supercomputer. However, bigger genomes such as fungal genomes may require more computer power than general local computers, and data analysis can crash or take much longer than when working remotely with a supercomputer. Additionally, annual licensing costs for Geneious are between $840 (2 computers in an academic institution) to close to $15,000 (10 computers in a corporation).

BaseSpace is a website developed by Illumina where registered users can easily store, analyze, and share genetic data (https://basespace.illumina.com). One of the benefits of BaseSpace is that users have access through their Illumina accounts, and the sequencing data from the sequencers, including iSeq100, are streamed in real-time over the Internet to BaseSpace at no additional cost. Once the sequencing data is in the BaseSpace Sequencing Hub, the user can access a limited set of free online BaseSpace apps for genomic and transcriptomic data analysis. However, most of the applications required for genomic raw data processing and bacteria species and ARG identification are not free. A yearly BaseSpace Sequence Hub Professional subscription costs $500 and includes 500 iCredits to go towards data storage or app usage. Additional credits can be purchased for ~$1 each. The complete bioinformatic analysis using this platform costs about 7 iCredits or ~ $7 per sample, of which 5 iCredits are for the Bacterial Analysis Pipeline alone. Additionally, BaseSpace failed to identify some of the ARGs found using other approaches, so it may not be the best option for diagnostic labs that require consistent, accurate results for all microorganisms.

GalaxyTrakr [16] is a free, open-source instance created by the US FDA in the bioinformatic platform Galaxy (http://galaxyproject.org) for use by laboratory scientists in the GenomeTrakr network, the first distributed network of public health and university laboratories that collect and share genomic and geographic data from foodborne pathogens [16]. GalaxyTrakr adapts the most popular bioinformatics tools used in the CLI to a user-friendly interface so researchers and clinicians without previous bioinformatic experience can benefit from the same resources experienced bioinformaticians use. Therefore, we consider GalaxyTrakr as the best option for diagnostic labs. Limitations of GalaxyTrakr include that analyses may be subject to delays as they are performed remotely in the Galaxy supercomputer, and users are limited in how much data can be stored at any given time.

Altogether, the continued improvement of NGS technologies and resources for bioinformatics is bringing down the cost of sequencing applications. With optimization of DNA input and reagent volumes, an increased number of veterinary diagnostic laboratories can adopt this important tool for tasks such as identifying antimicrobial resistance signatures with minimal or no bioinformatics training.

## Supporting information

S1 File BaseSapce protocol. (DOCX)

S2 File Geneious protocol. (DOCX)

S3 File GalaxyTrakr protocol. (DOCX)

S4 File Step-by-step library prep protocol using optimized reagent volume reduced to a quarter. (PDF)
