# Supplementary material for: Optimized conditions for Listeria, Salmonella and Escherichia whole genome sequencing using the Illumina iSeq100 platform with point-and-click bioinformatic analysis
Source: PLoS One. 2022 Nov 30;17(11):e0277659. doi: 10.1371/journal.pone.0277659 (PMC9710801; doi:10.1371/journal.pone.0277659)
Supplement: S1 File — (DOCX) [file pone.0277659.s001.docx]

*S1-Basespace*

*Step by step protocol for NGS raw data processing and bacteria species identification in 11 easy steps!*

*Sonsiray Alvarez Narvaez*

*University Of Georgia |Georgia, Athens, US*

*December 2021*

**RAW READS QUALITY CHECK**

1. Sign-in into your Illumina BaseSpace account at <https://basespace.illumina.com>, which will give you access to your SEQUENCE HUB. Of note, this manual has been produced using macOS Big Sur v11.2 and BaseSapce version of December 2021. Some of the features may appear differently presented in Windows operating systems.

2. Click on the top right symbol with three lines and then select “Apps.”


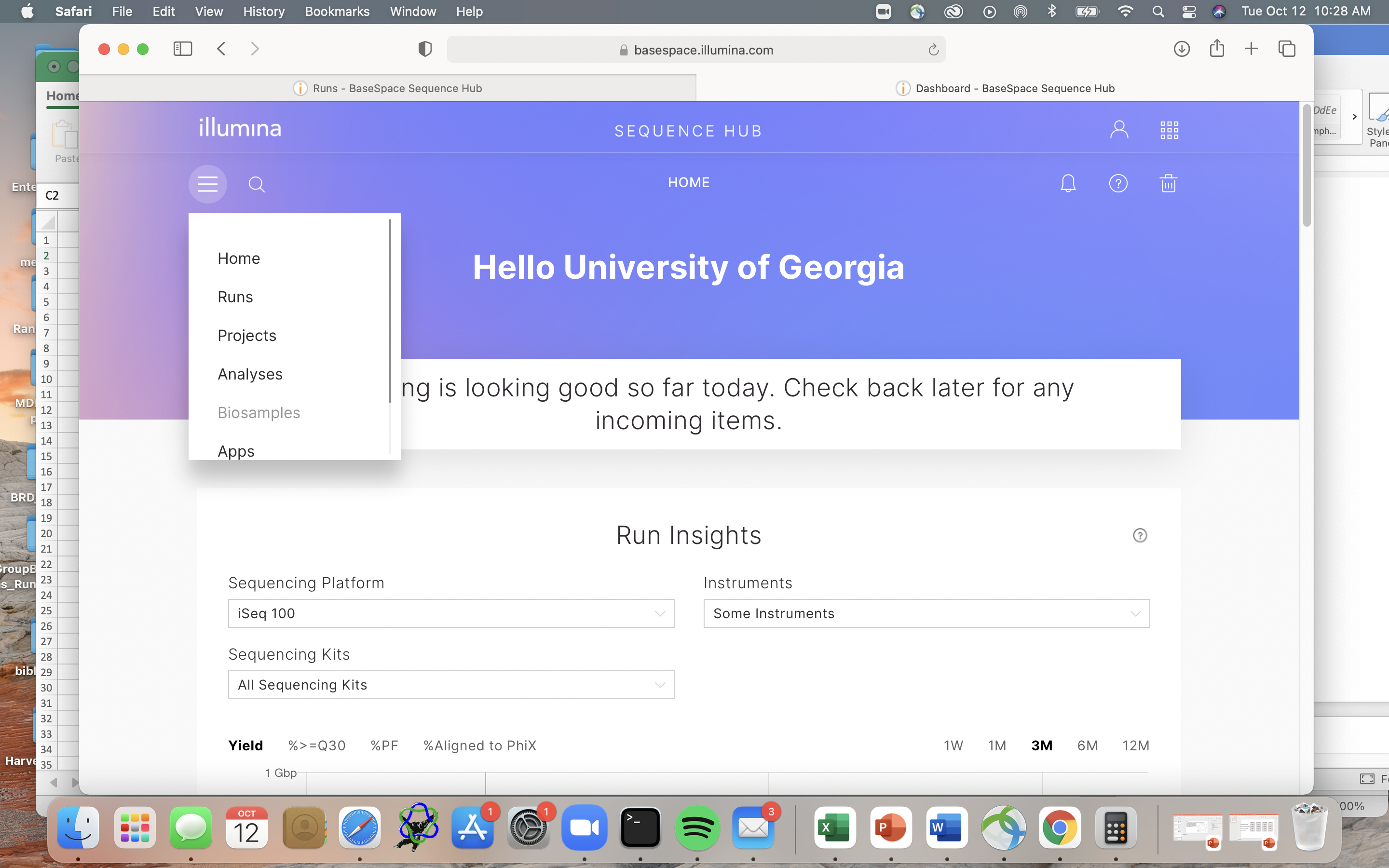


3. Once inside the Apps tab, search for program “FastQC” and click on the symbol to select.


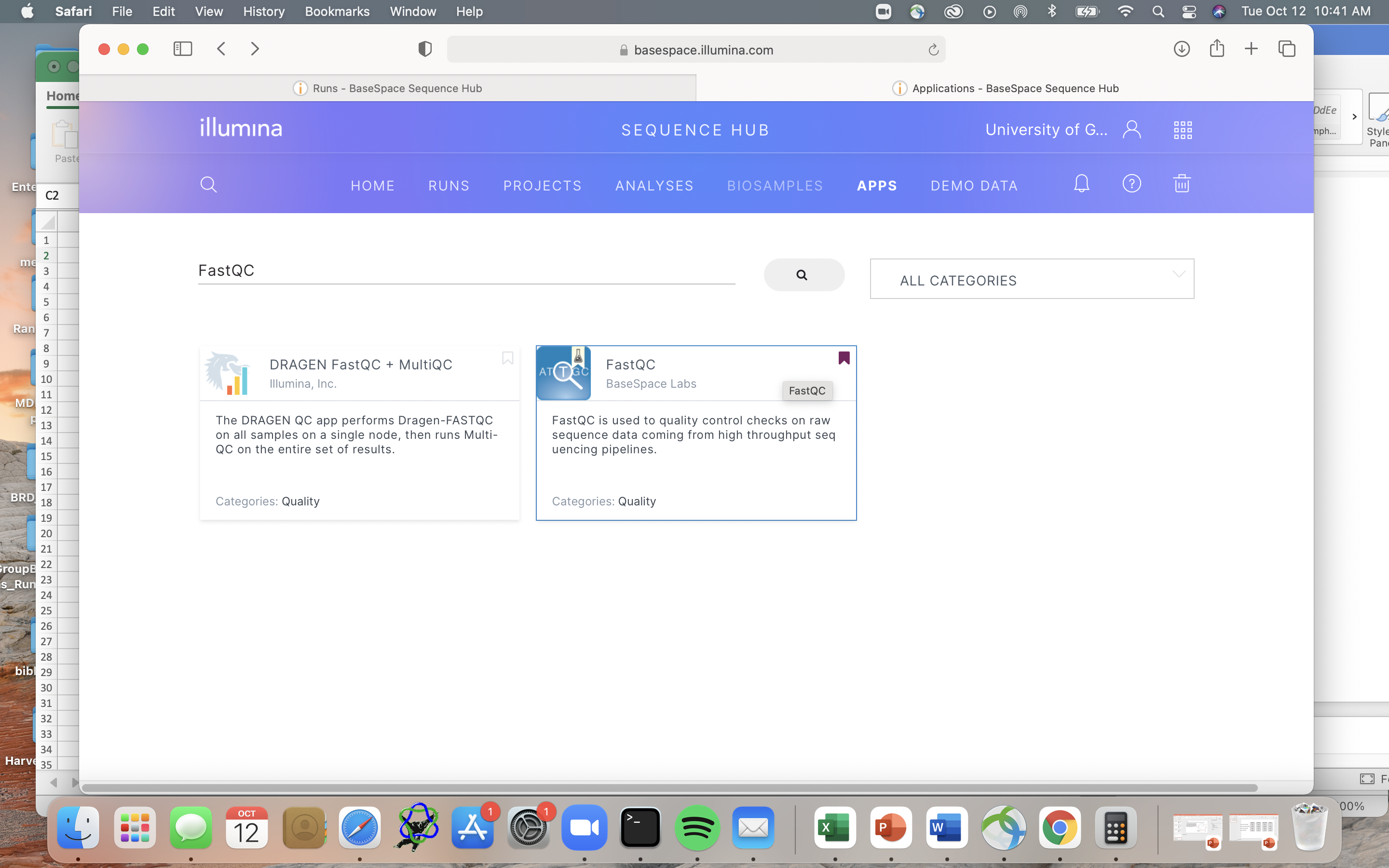


4. Once inside FastQC app., you will find a short description of the program, the price of using the app (“Pricing”), an option to choose the version of the program you want to run (“version”), and a purple button that says: “LAUNCH APPLICATION.” Click on it to select.


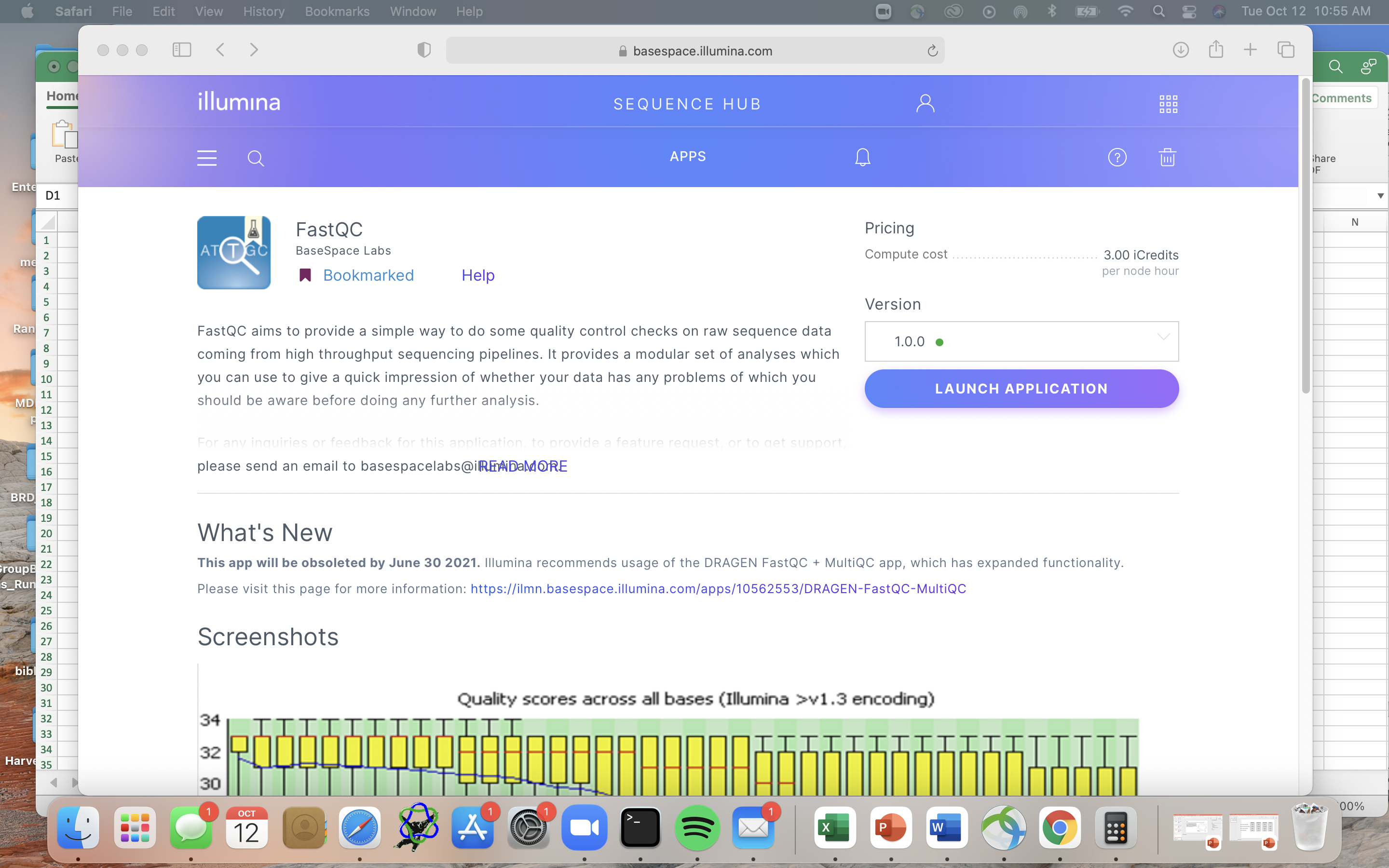


5. Once you have launched the application, under “Output,” you can change the “App Session Name” by clicking on top of the default name, and you can select in which project you will save your results by clicking on “Select Project.” Click on your desire project (in this case, Salmonella), and a blue light next to the name will inform you that this particular project was selected. When you are happy with your choice, click the purple “SELECT” button.


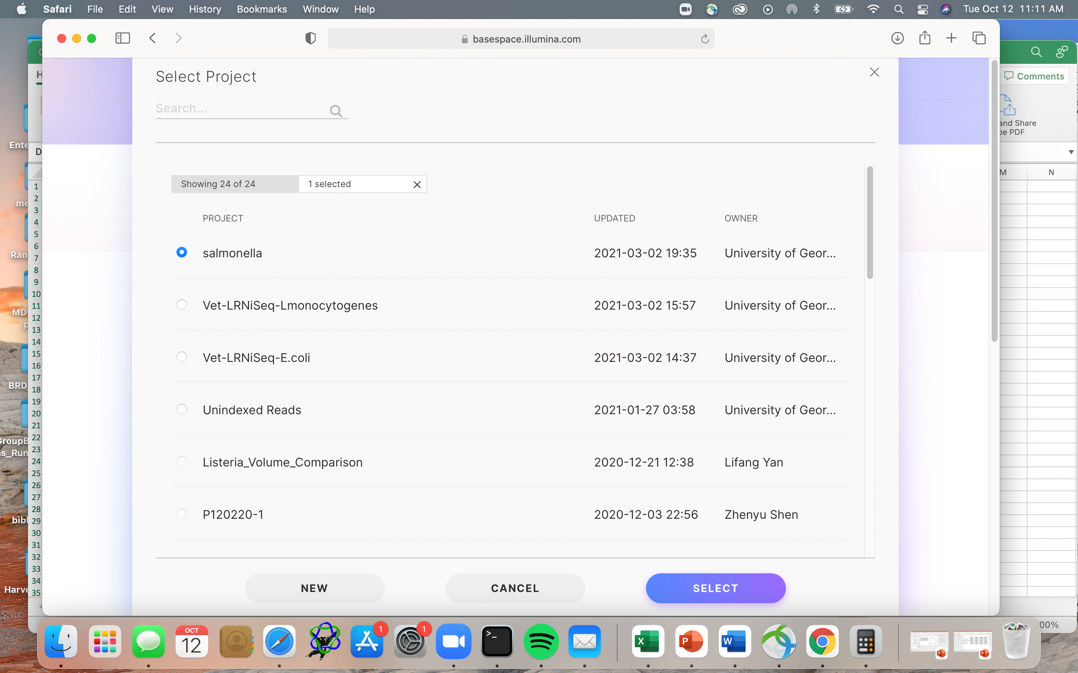

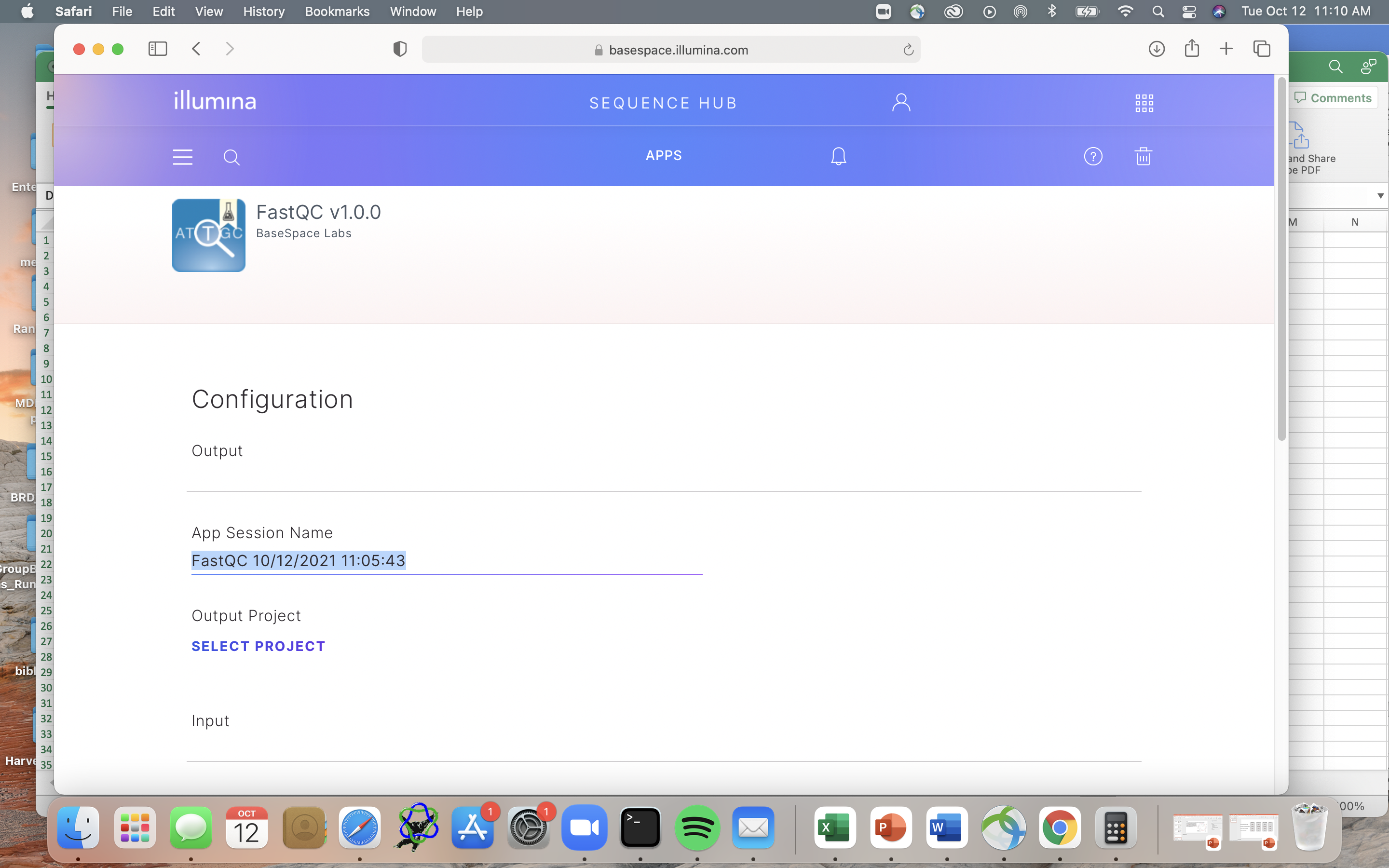


Under “Input,” click on “Select Sample” to select the sample you want to analyze. You can choose only one sample at a time. Next, click on your desire sample (in this case, salmonella2_one), and a blue light next to the name will inform you that this particular sample was selected. When you are happy with your choice, click the purple “SELECT” button.


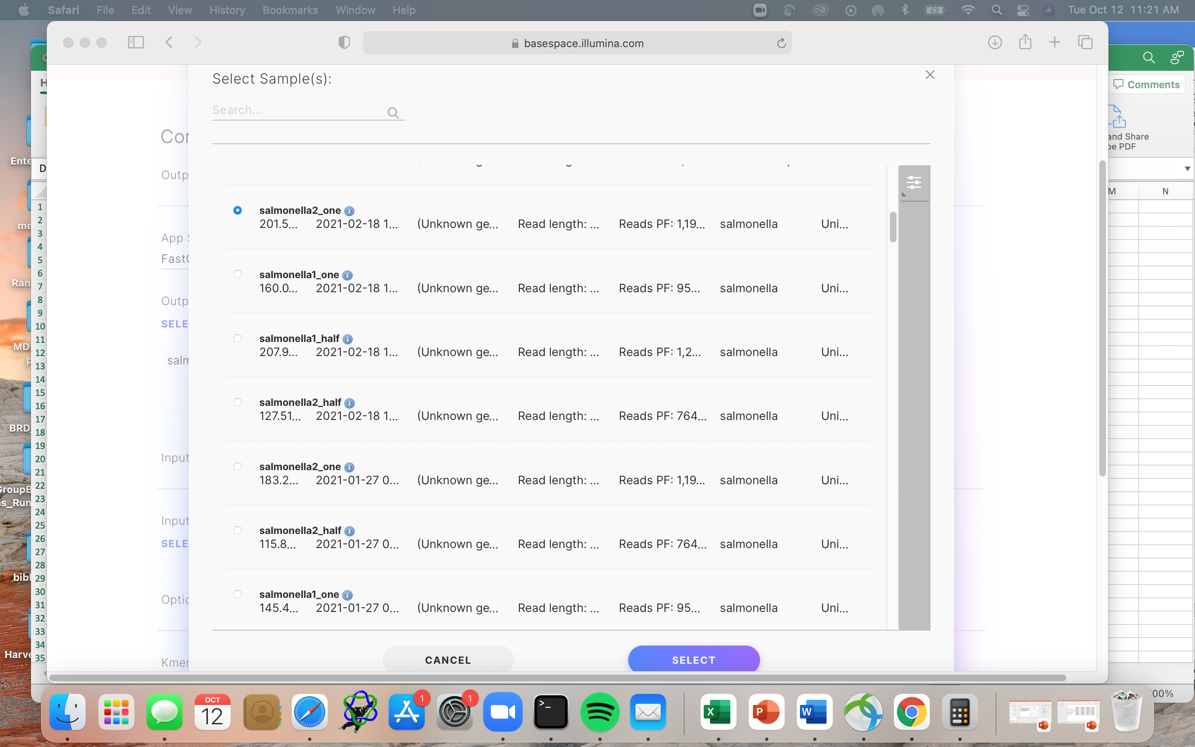

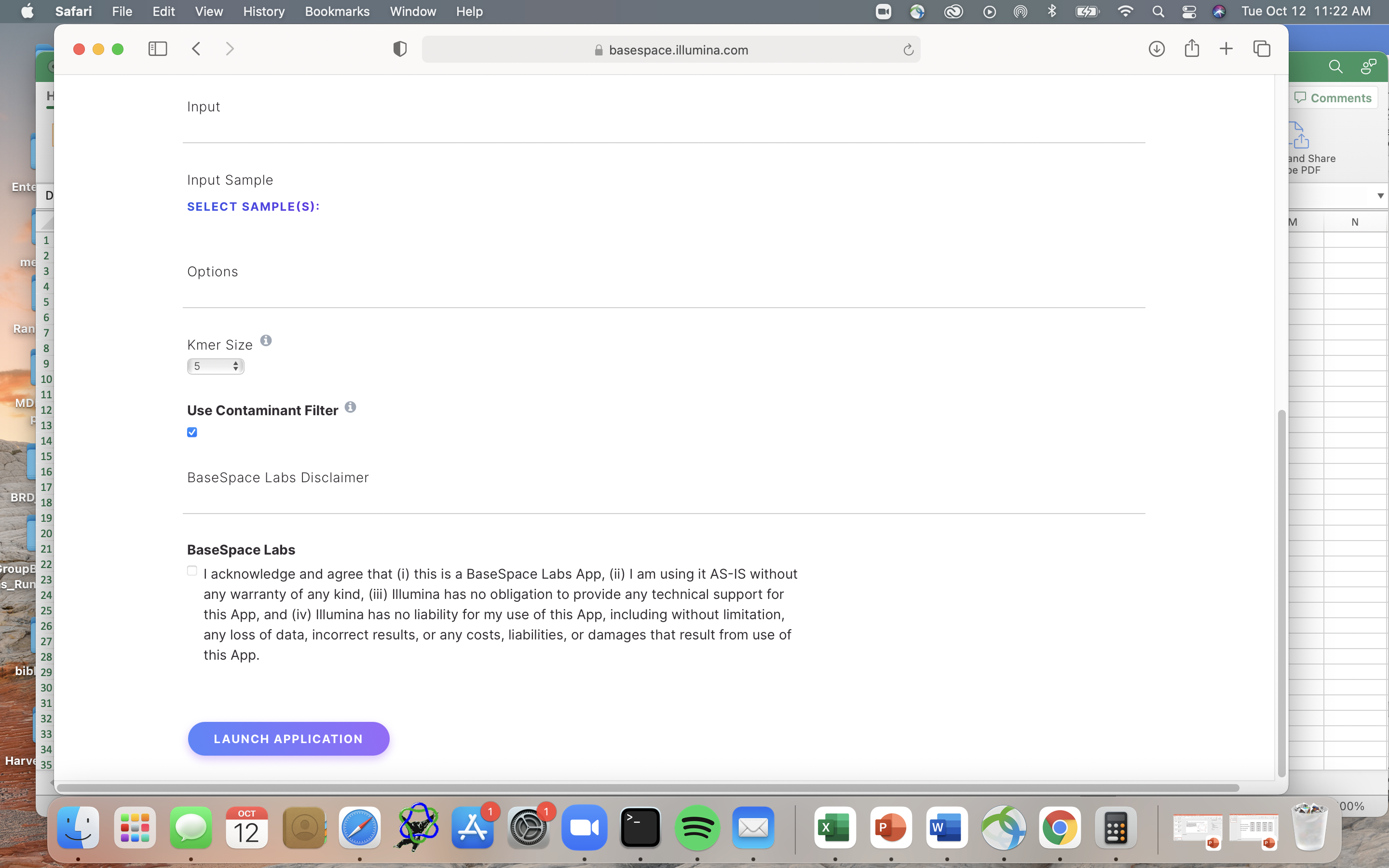


Finally, under “Options,” we will use the default parameters that include “Kmer Size” of 5 and “Use Contaminant Filter.” Accept the “BaseSpace Lab” acknowledgment when you are ready and click on the purple “LAUNCH APPLICATION” button.


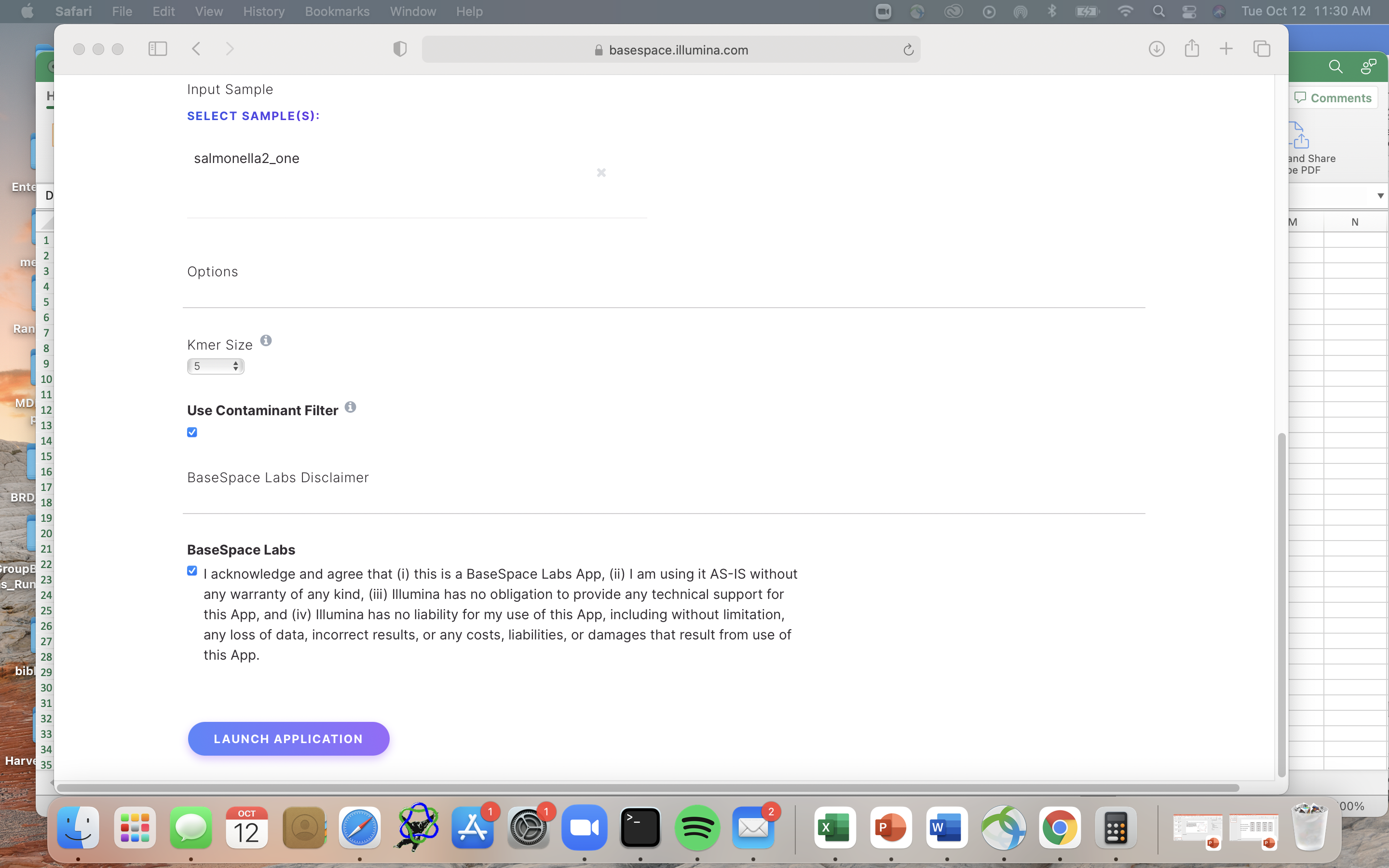


A new window will open, indicating that the program is running. Once the analysis is done, you can download or visualize the analysis. T download, click on “Download Analysis.” Next, a new window will open in which you have to select “All file types including VCF, BAM, & FASTQ” and subsequently click the “Download” purple button. Then, another new window will open in which you have to select the directory where you want to save your data by clicking on the three dots button, and finally, you can “Start Download.” Of note, in the BaseSpace version for Windows operating systems 5 icons will appear on the left below Summary. The icons include a greyed-out Launch, File, Status, Share and Delete. Under File, you will find the option to download.


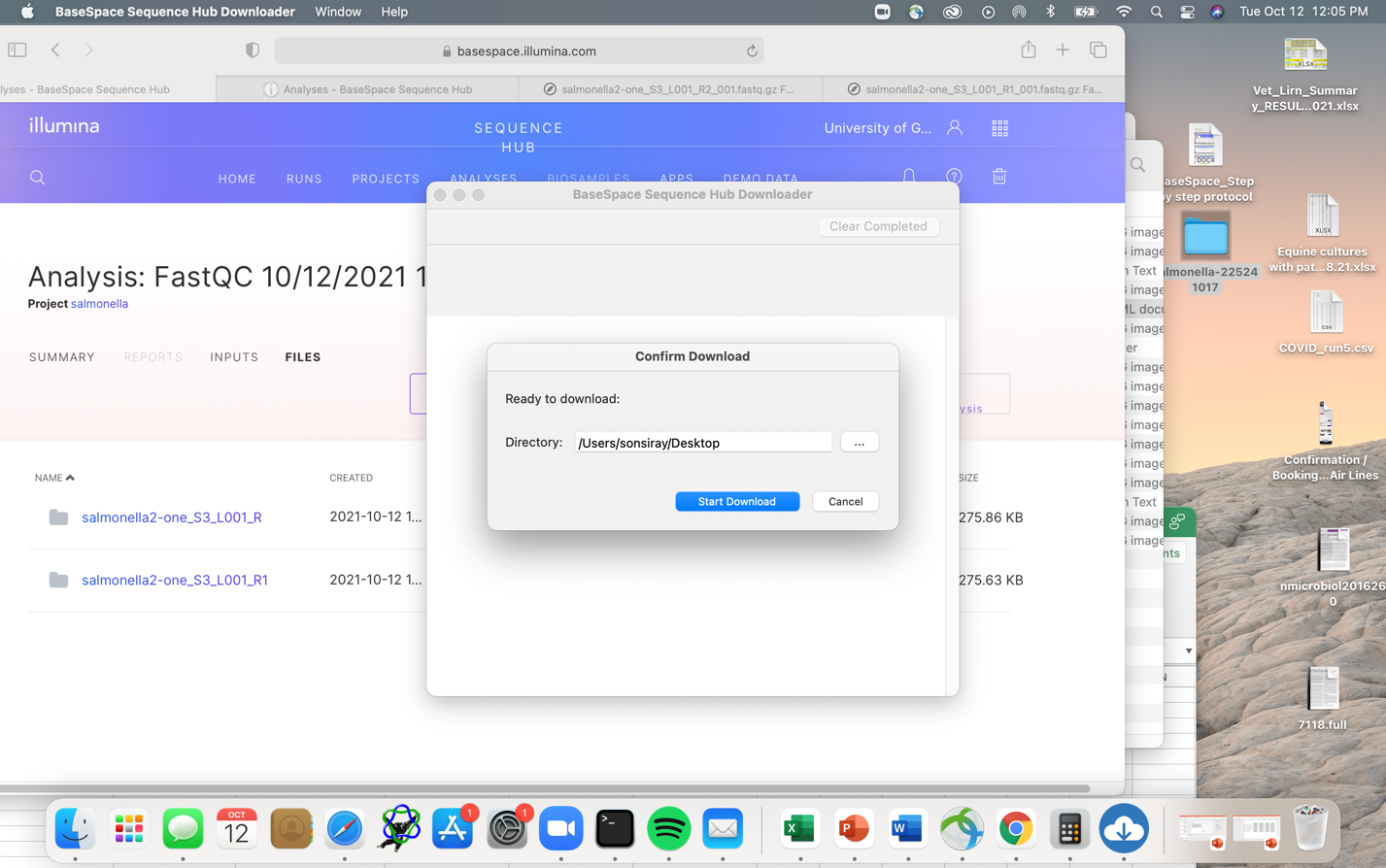

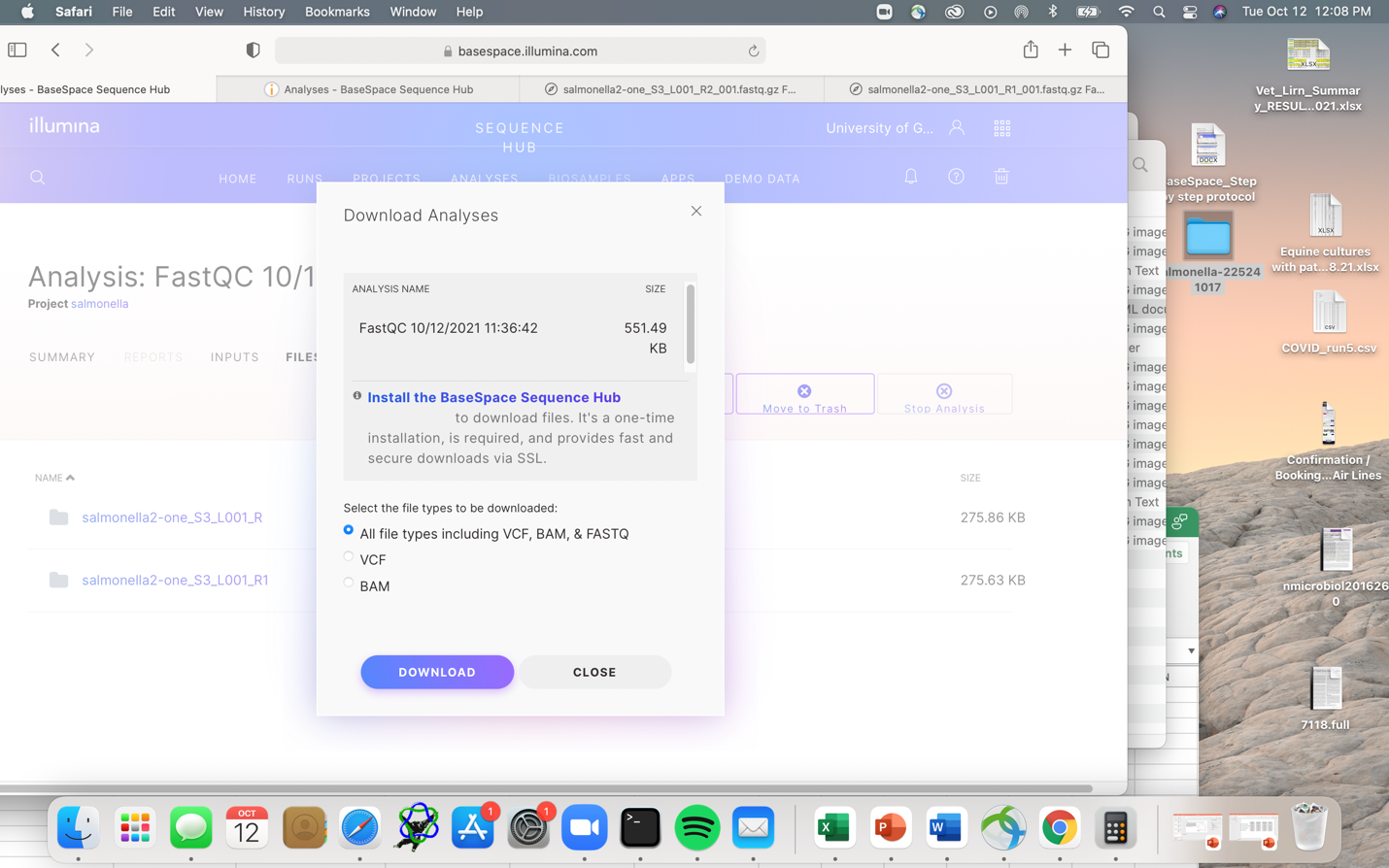

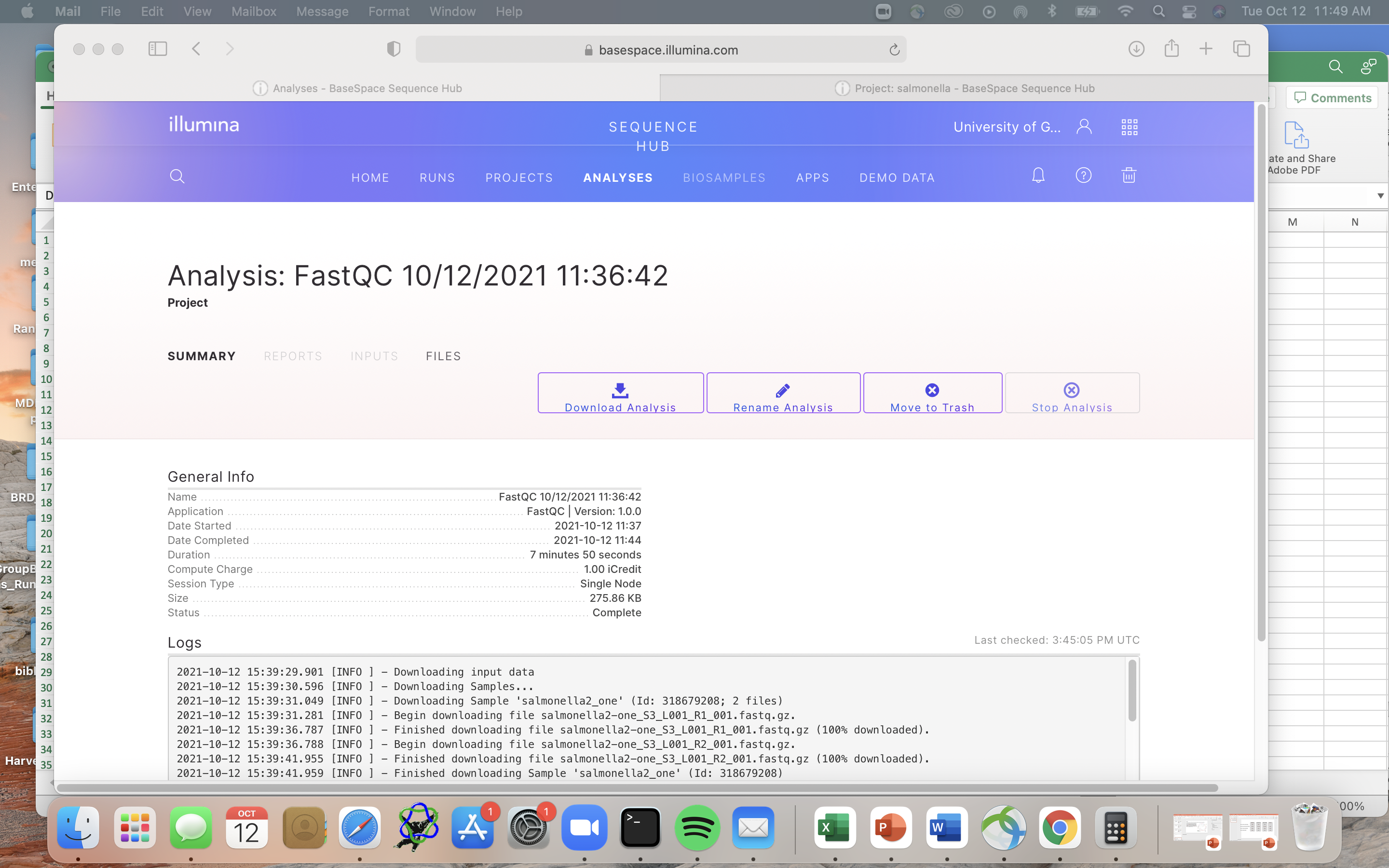


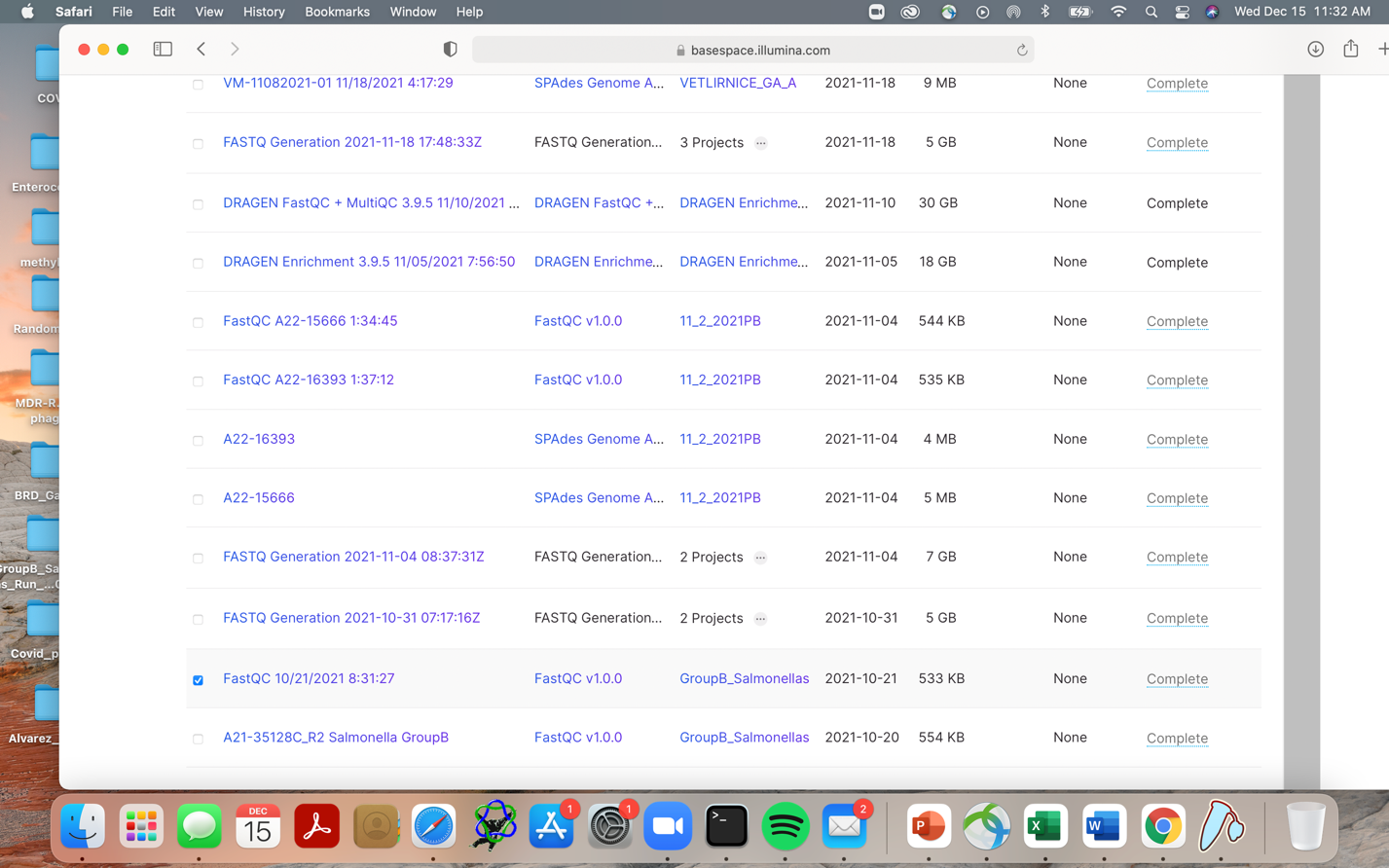
To visualize the analysis, go to the “Analyses” tab in Sequence Hub and click on your FastQC analysis.
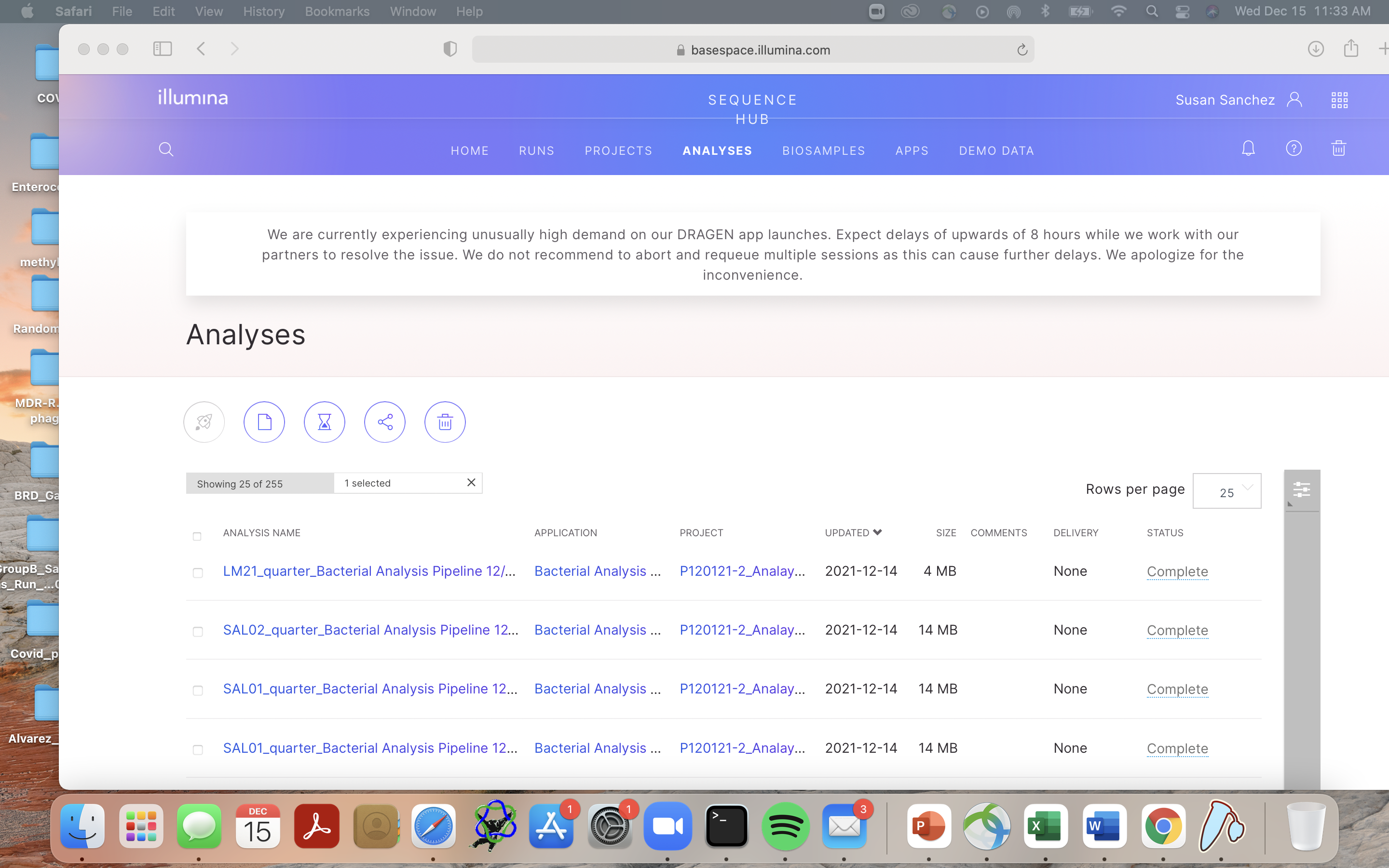


Once inside your FastQC Analysis click on “REPORTS”. If you are using a paired-end library, you will see two reports that correspond to your forward and reverse reads. Click on any of them to start visualizing your report. Please, look at the quality of both reads before proceeding with further analysis.


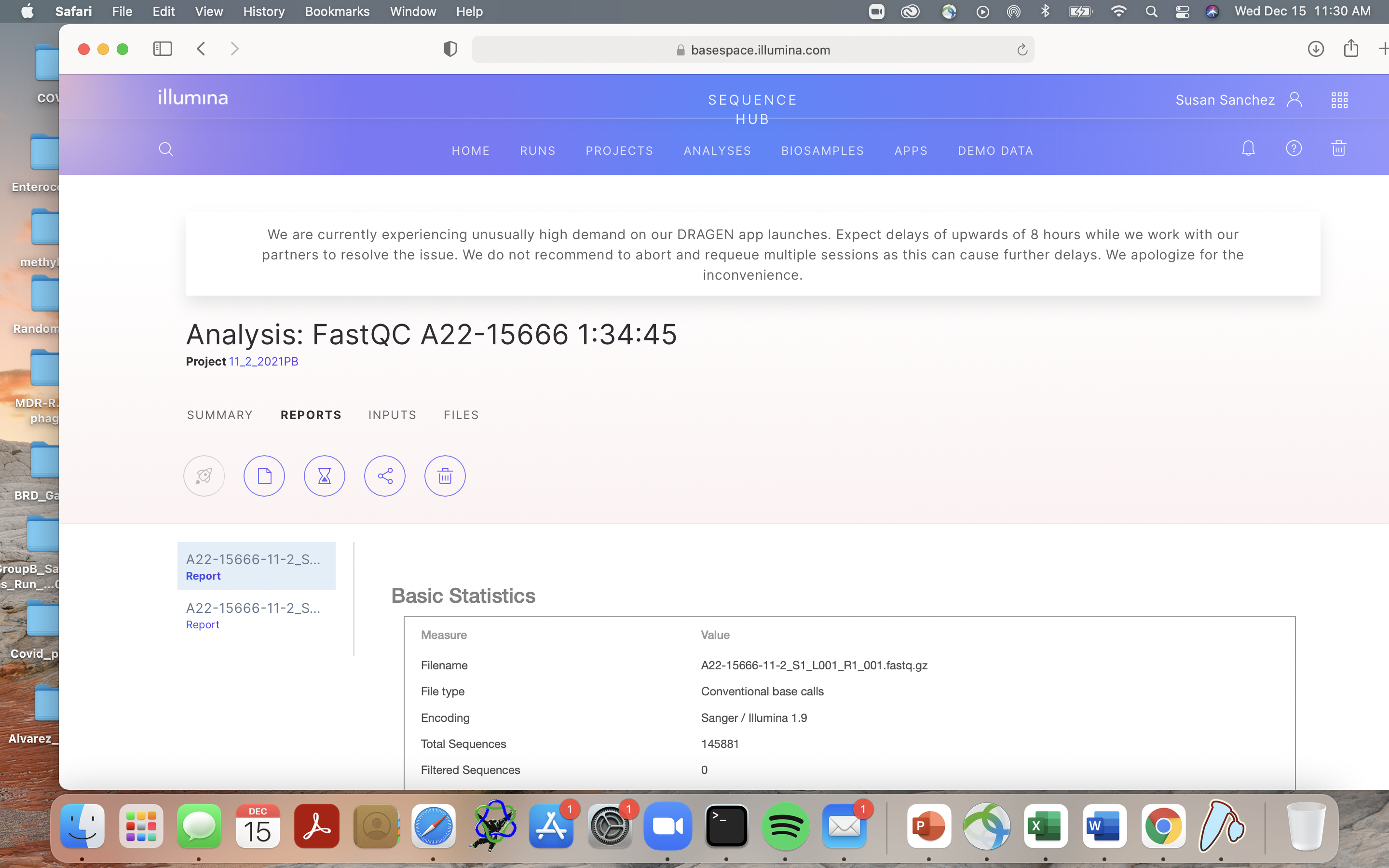


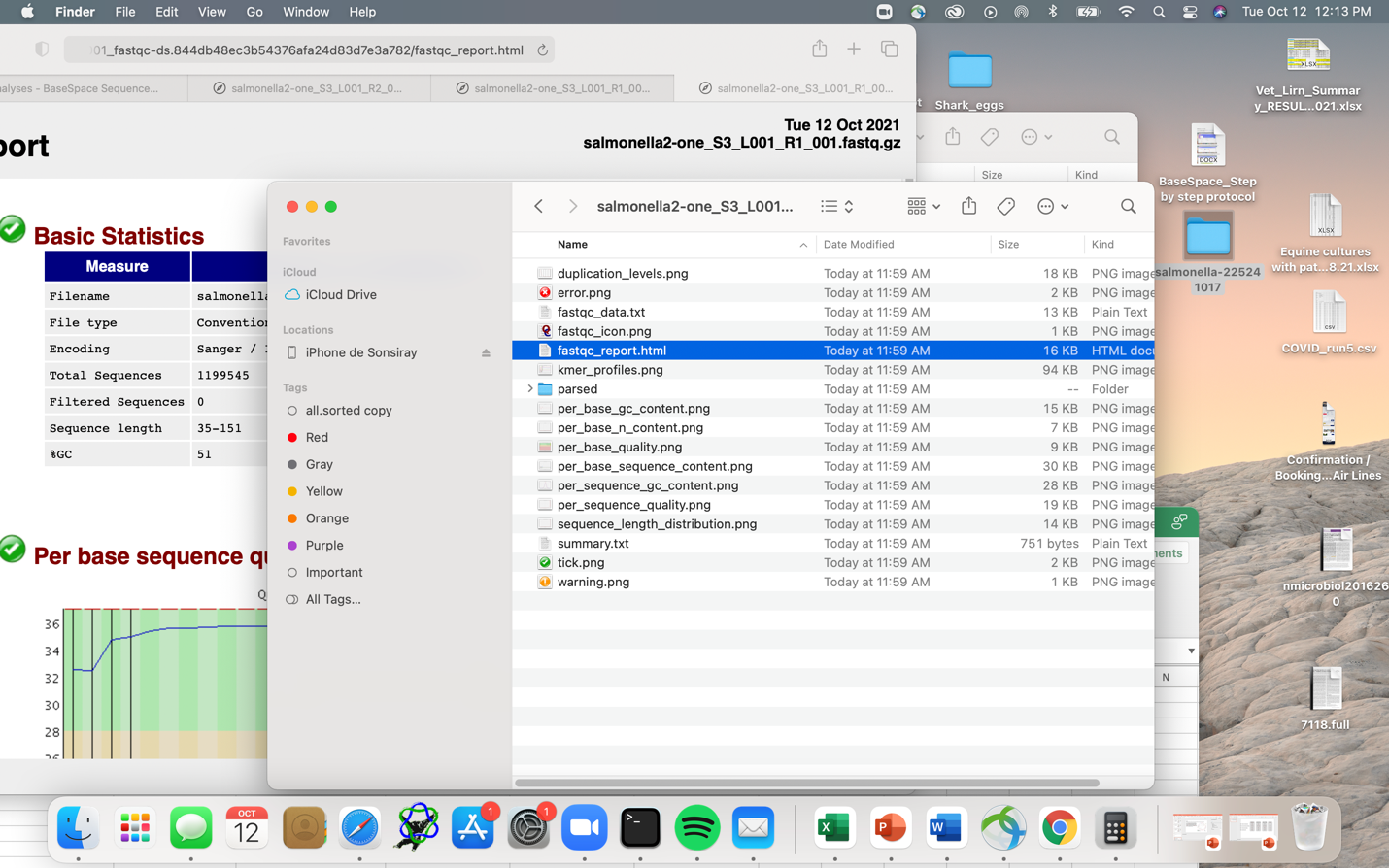
6. A folder will be downloaded to the directory of your choice. Open the file with the name “fastqc_report.html.” The first three categories, “Basic Statistics,” “Per base sequence quality,” and “Per sequence quality scores,” are what require your attention. If these tests have a green tick, that means that the quality of your reads is good, and you can continue with your analysis. “Basic Statistics” gives you a summary of your analysis. “Per base sequence quality” shows an overview of the range of quality values across all bases at each position of the read (the higher the score, the better the quality). Finally, “Per sequence quality scores” shows the distribution of quality scores of your reads (you want to observe a peak at 28 or above).


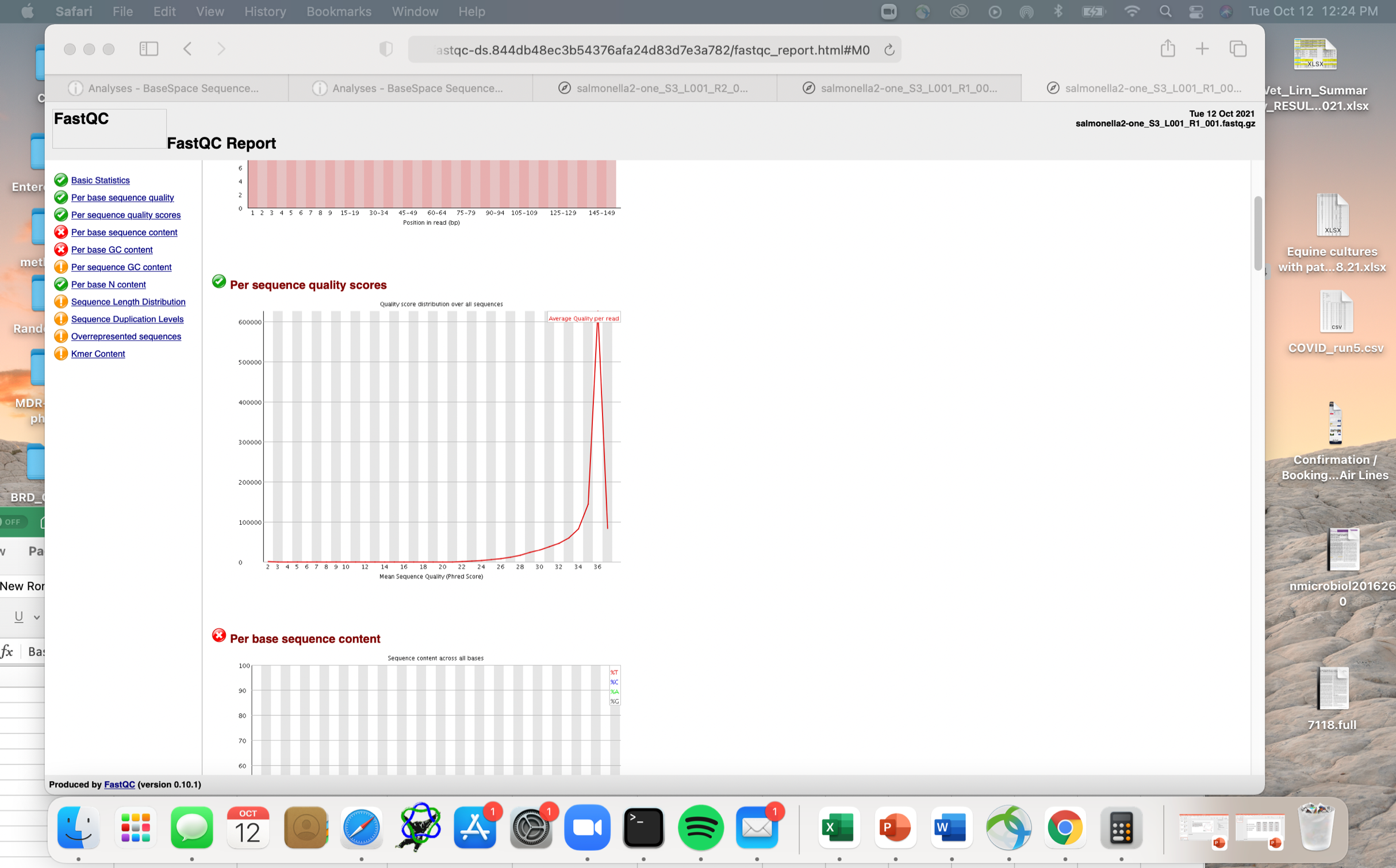

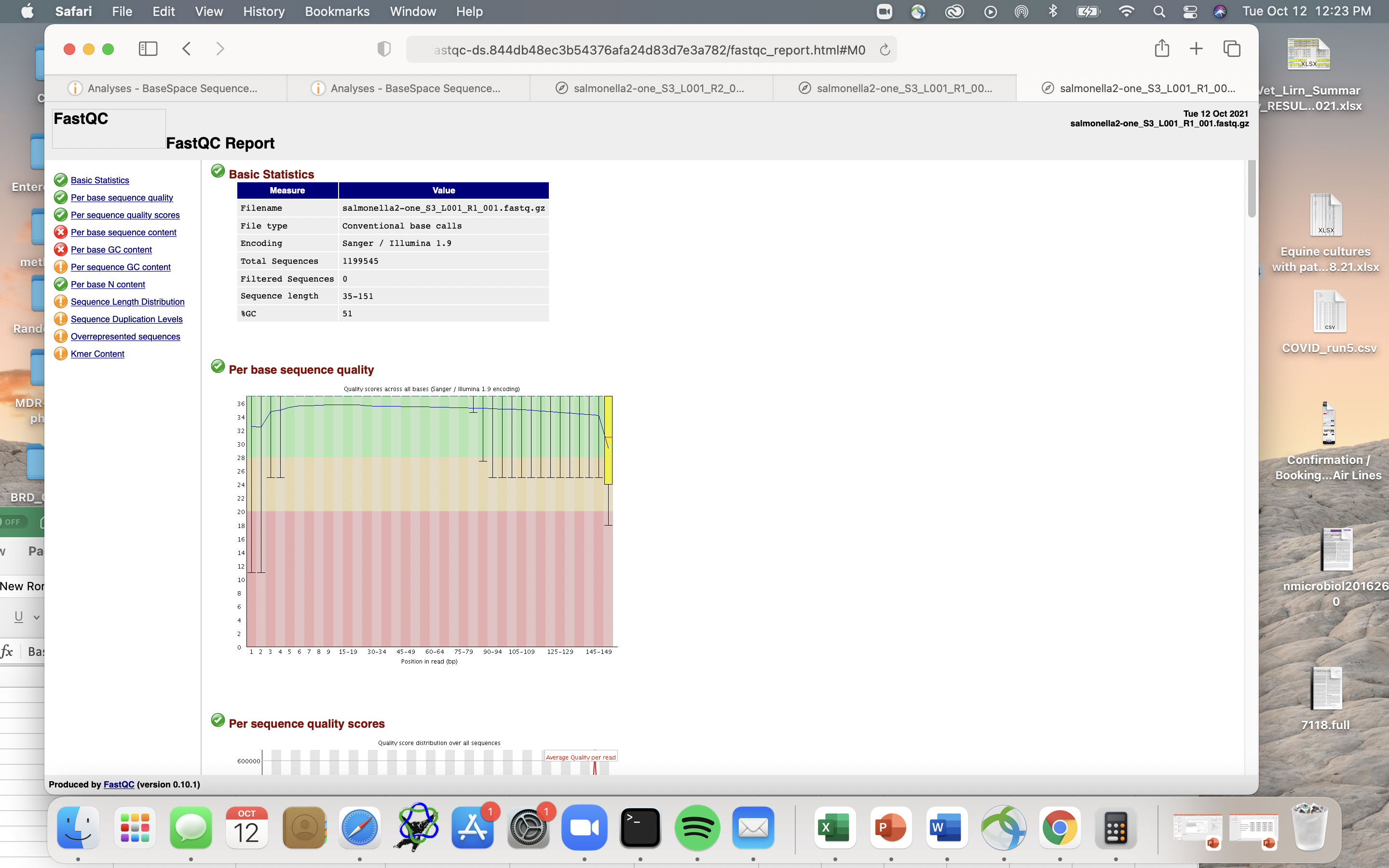

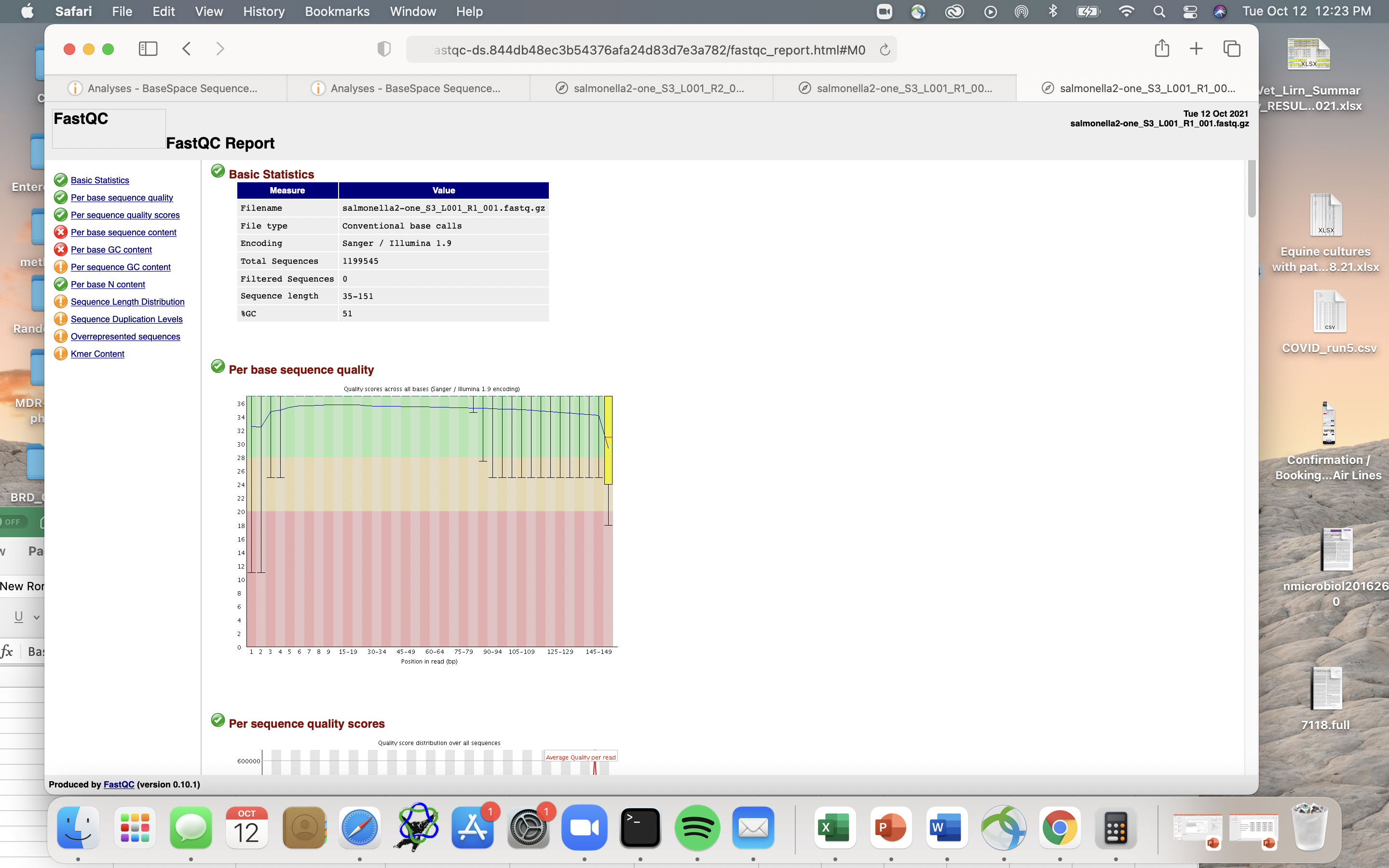


Once you have tested that the quality of your reads is optimal, you can continue with **read** **trimming** and **filtering**.

**RAW READS TRIMMING AND FILTERING**

7. Back on the SEQEUNCE HUB, go back to the “APPS” tab by clicking on it, looking for the FASTQ toolkit program, selecting it like previously done in step 3, and launching the application as you did in step 4. The FASTQ Toolkit allows manipulation of FASTQ files, including adapter trimming, quality trimming, length filtering, format conversions, and down-sampling.


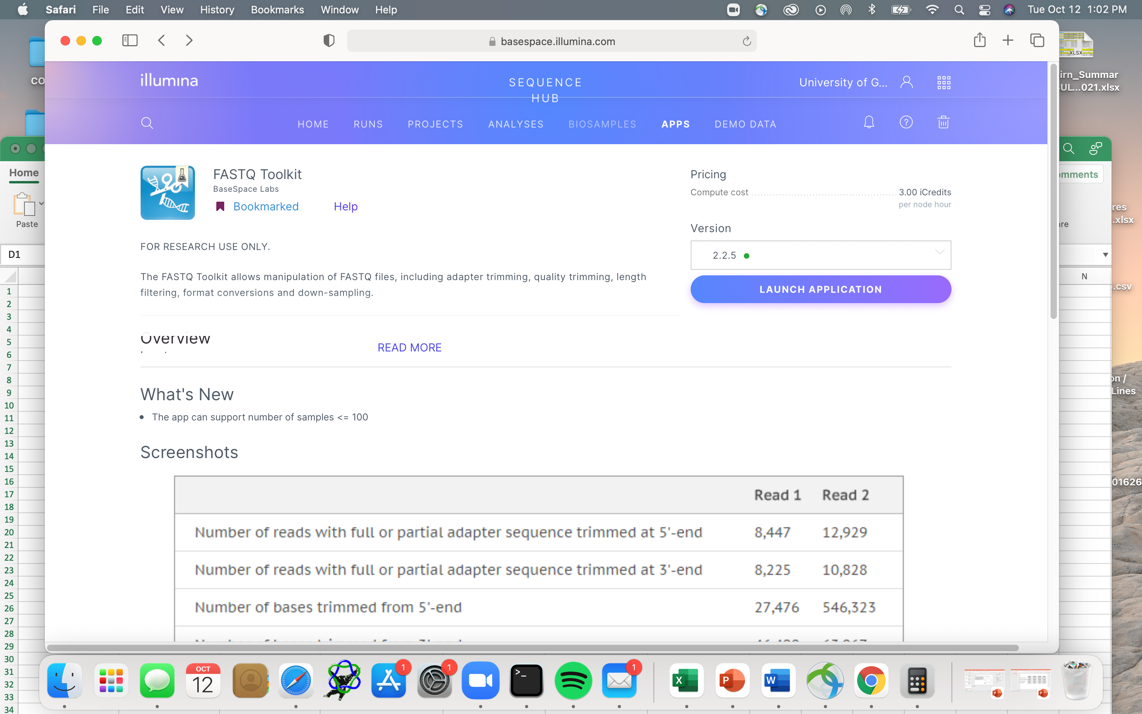

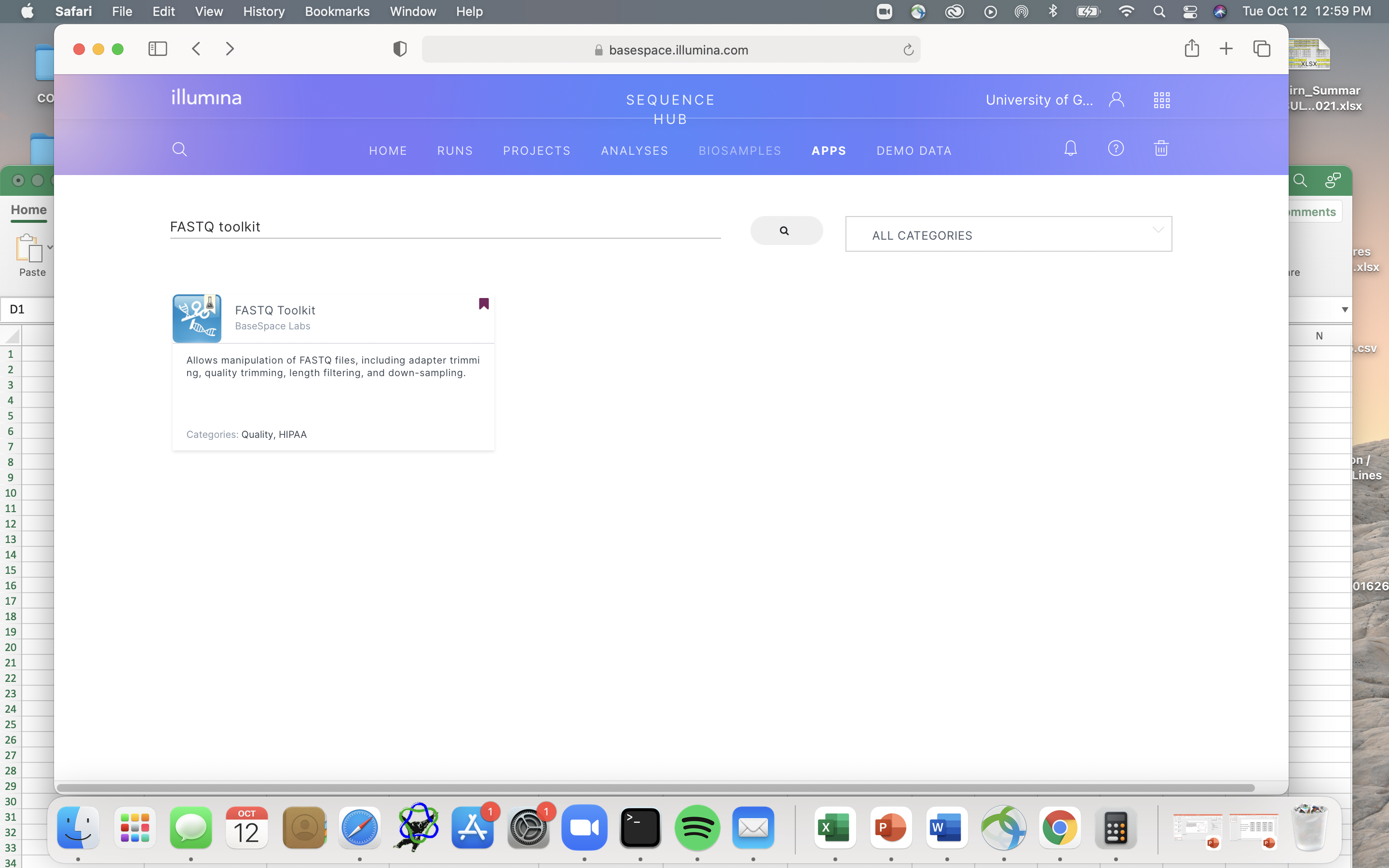


8. Once you have launched the application, you can change the “Analysis Name” by clicking on top of the default name. Under “Input Sample(s) to Process,” click “Select Sample(s),” select the samples you want to analyze, and click the purple “SELECT” button.


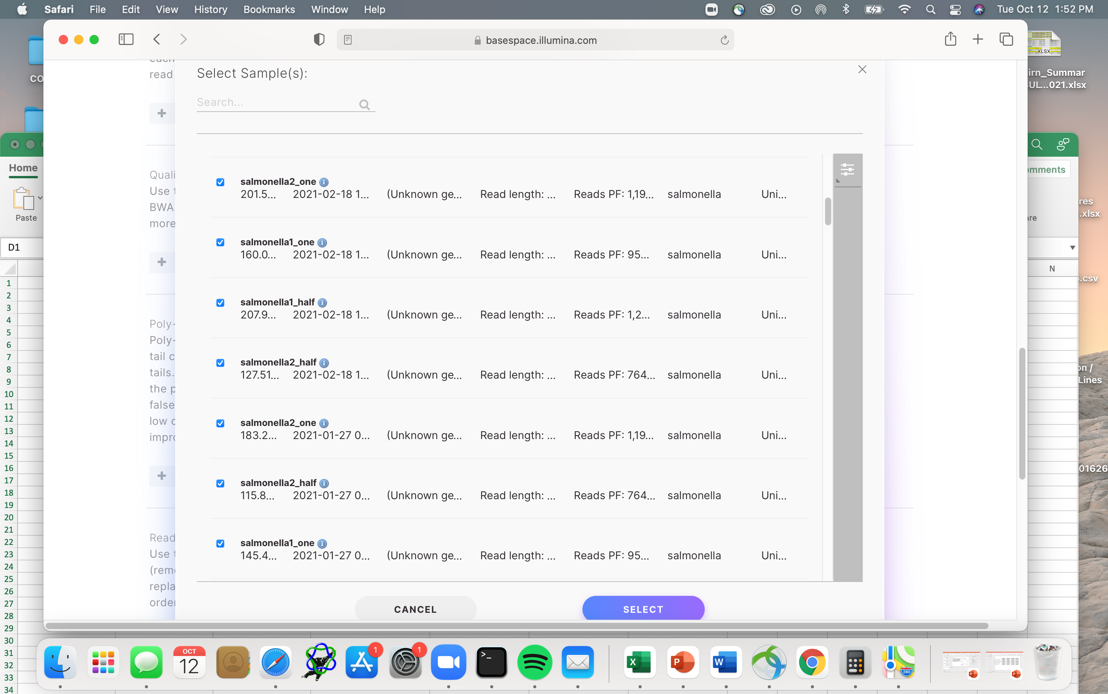

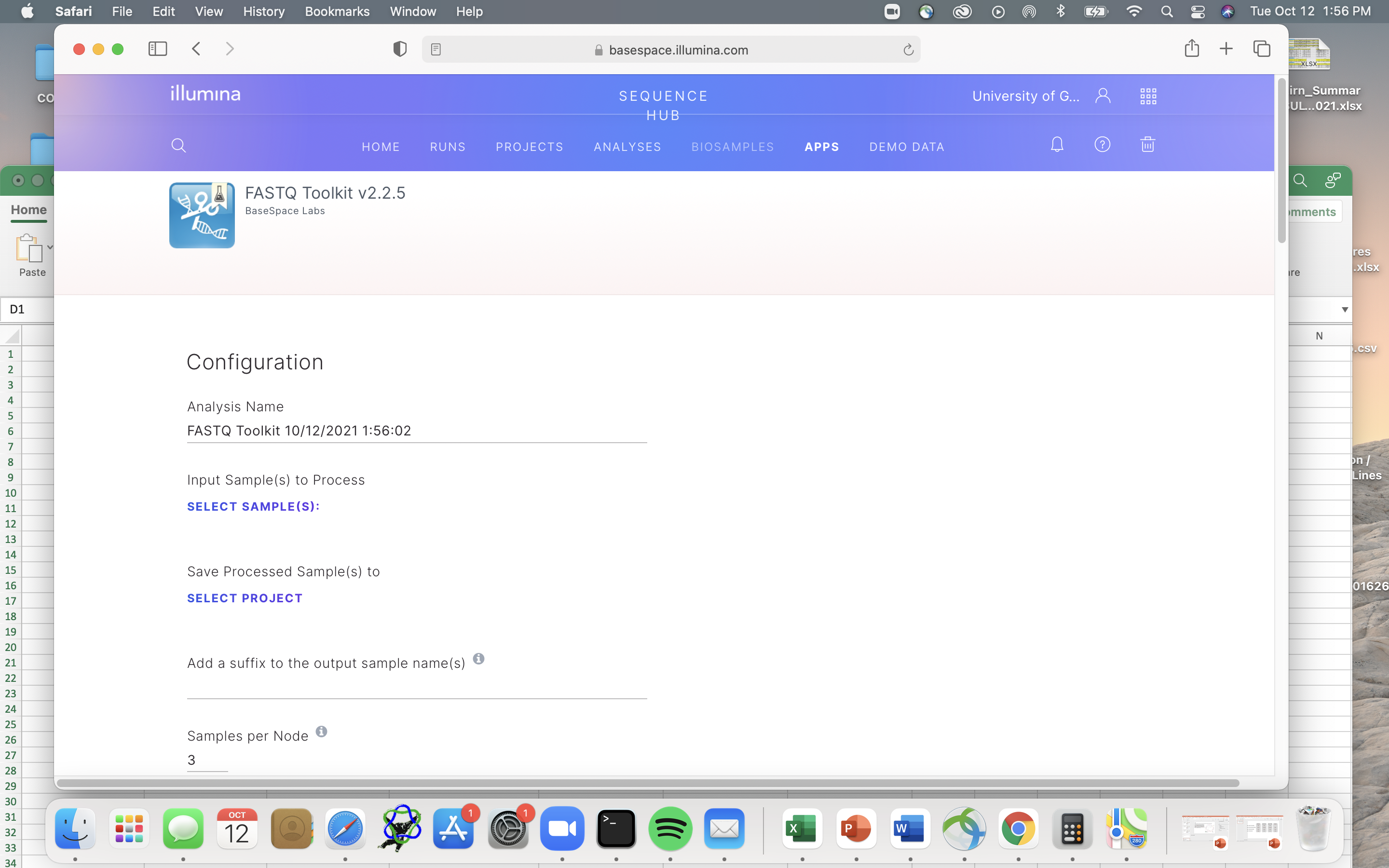


Under “Save Processed Sample(s),” click “Select Project” to choose in which project you will save your results. Next, click on your desire project (in this case, Salmonella), and a blue light next to the name will inform you that this particular project was selected. Finally, when you are happy with your choice, click the purple “SELECT” button.


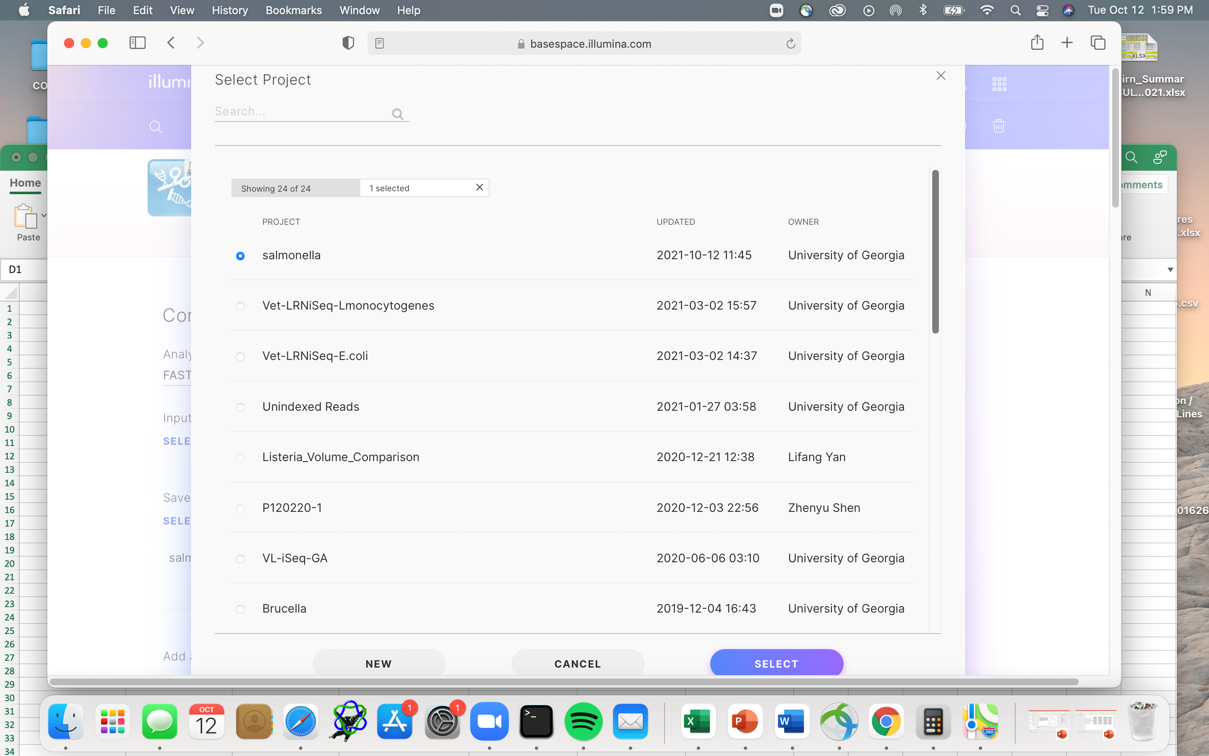

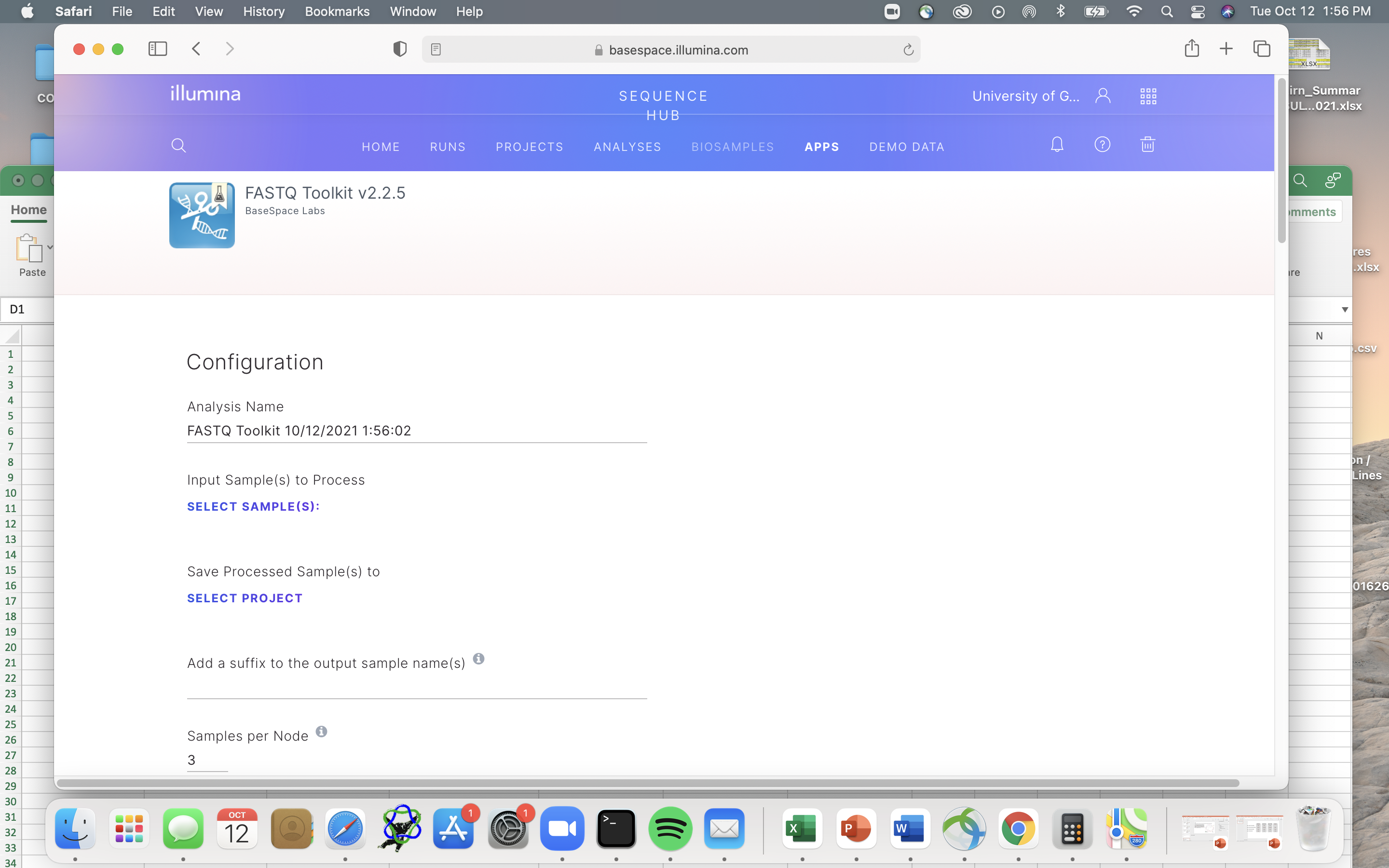


Then, “Add a suffix to the output sample name(s)” so you can recognize them more easily. In this case, we added “salmonellas_trimmed_reads.” We use the default setting, so leave “Samples per Node” in 3 and “Minimum read length” in 32 bp.


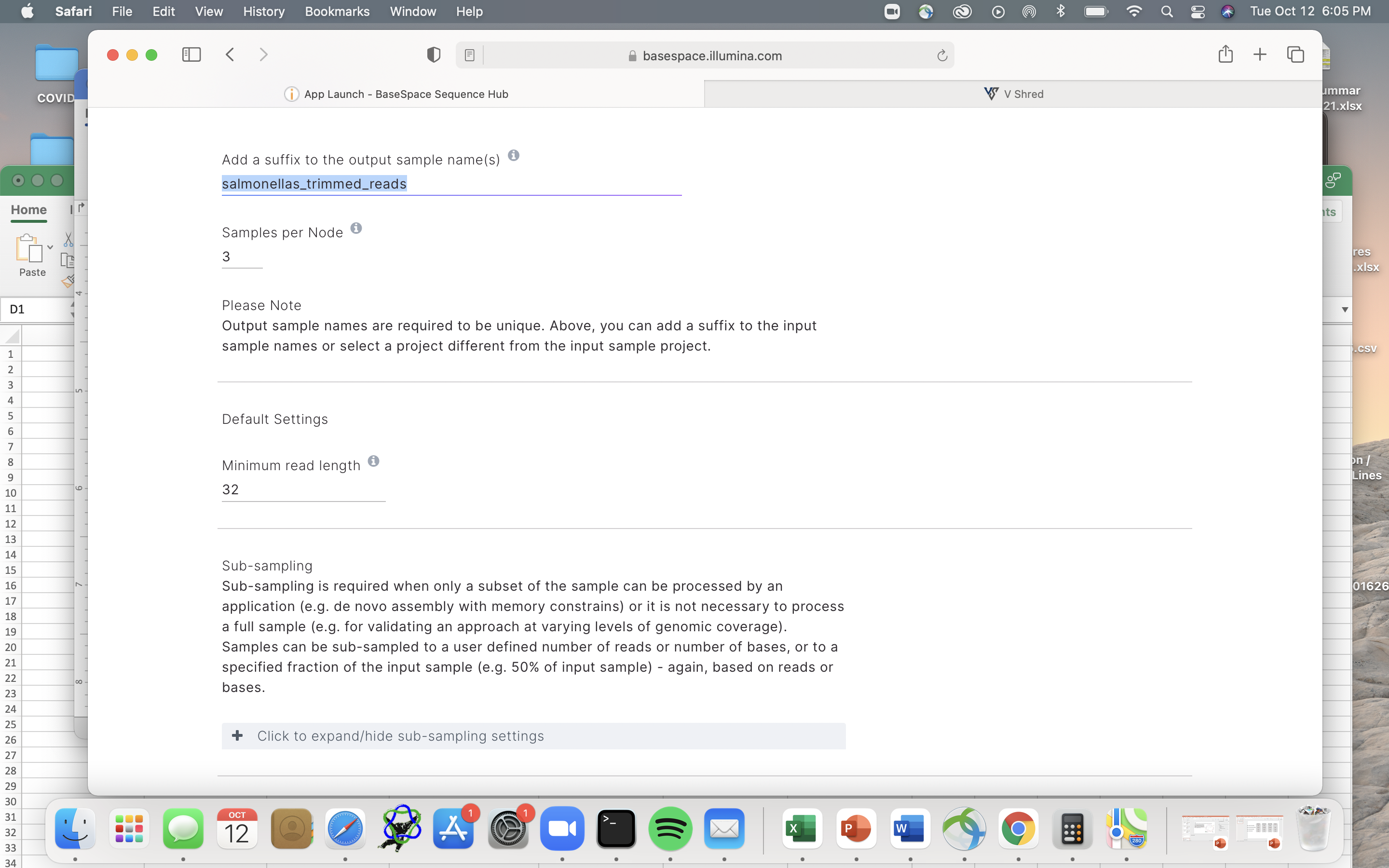


Go to the “Adapter Trimming” section and select the correct adapters you are using in your library prep (in our case, we are using TruSeq Dual Index), and choose for “Trim Ns from the 3'-end before identifying adapters”.


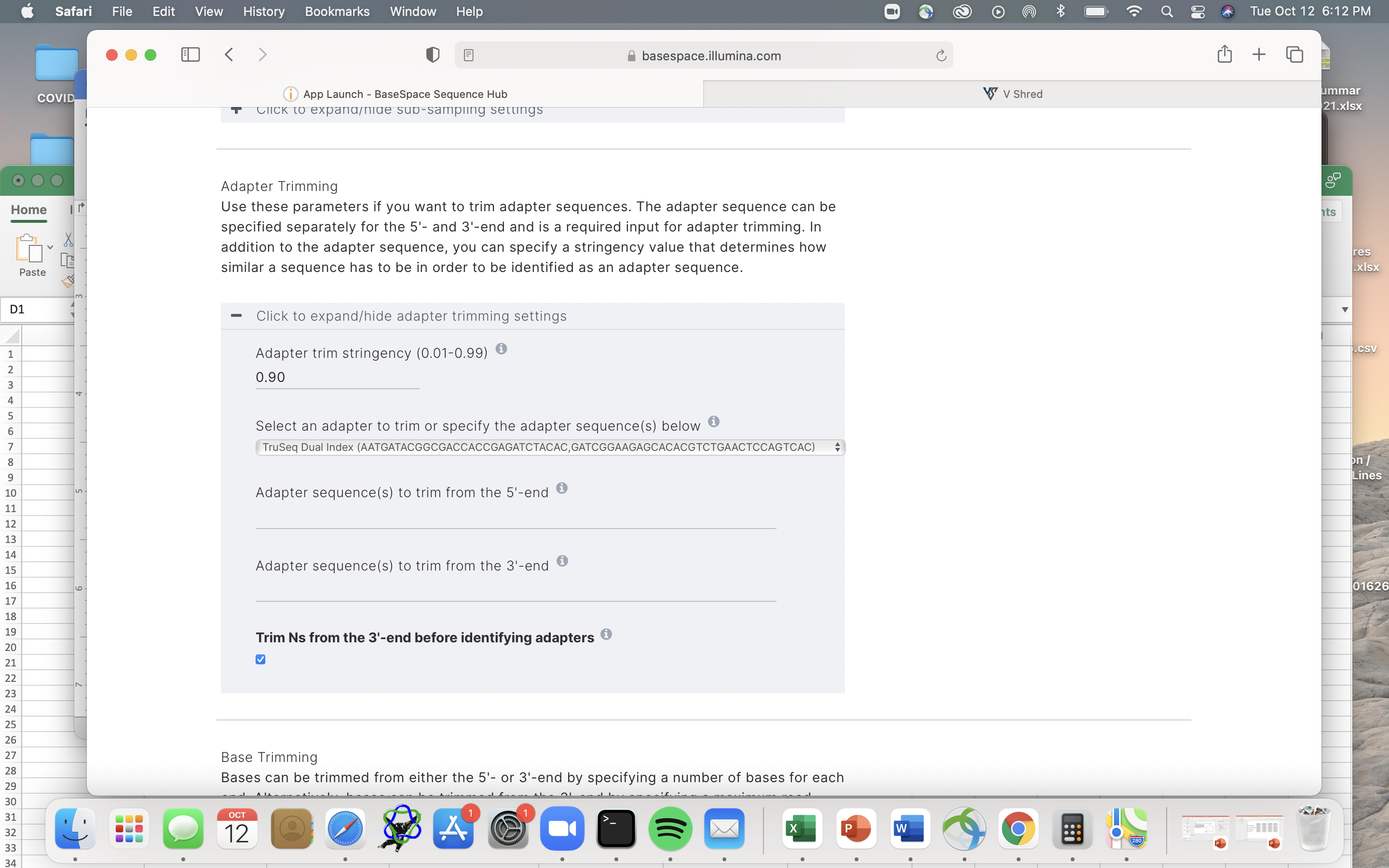


Go to the “Read Filtering” section and select “Minimum mean quality score” of 30, “Only keep reads passing filters,” and “Remove singletons.” Finally, leave every other setting as default, select the BaseSpace acknowledgment and click on the purple “LAUNCH APPLICATION” button.


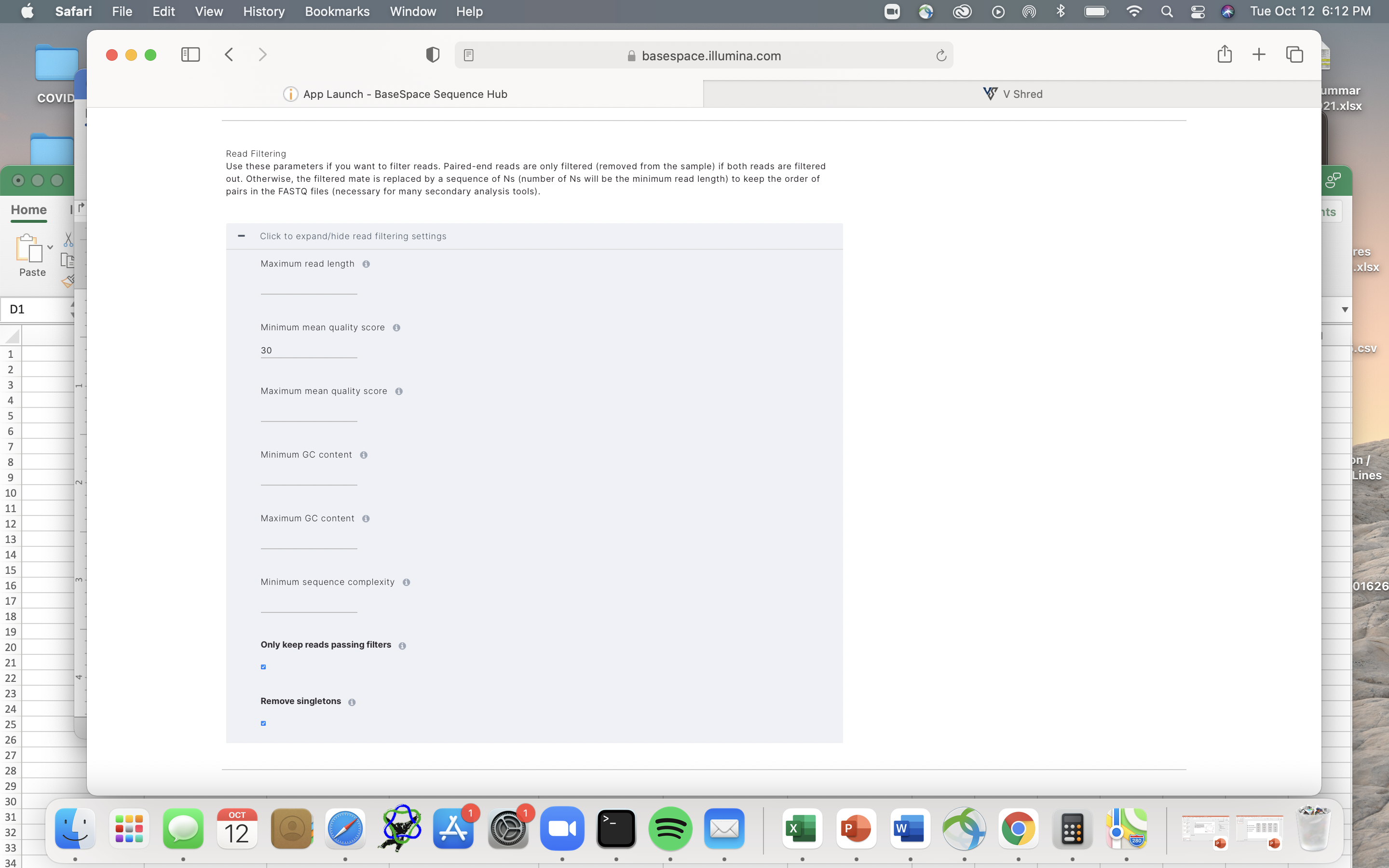


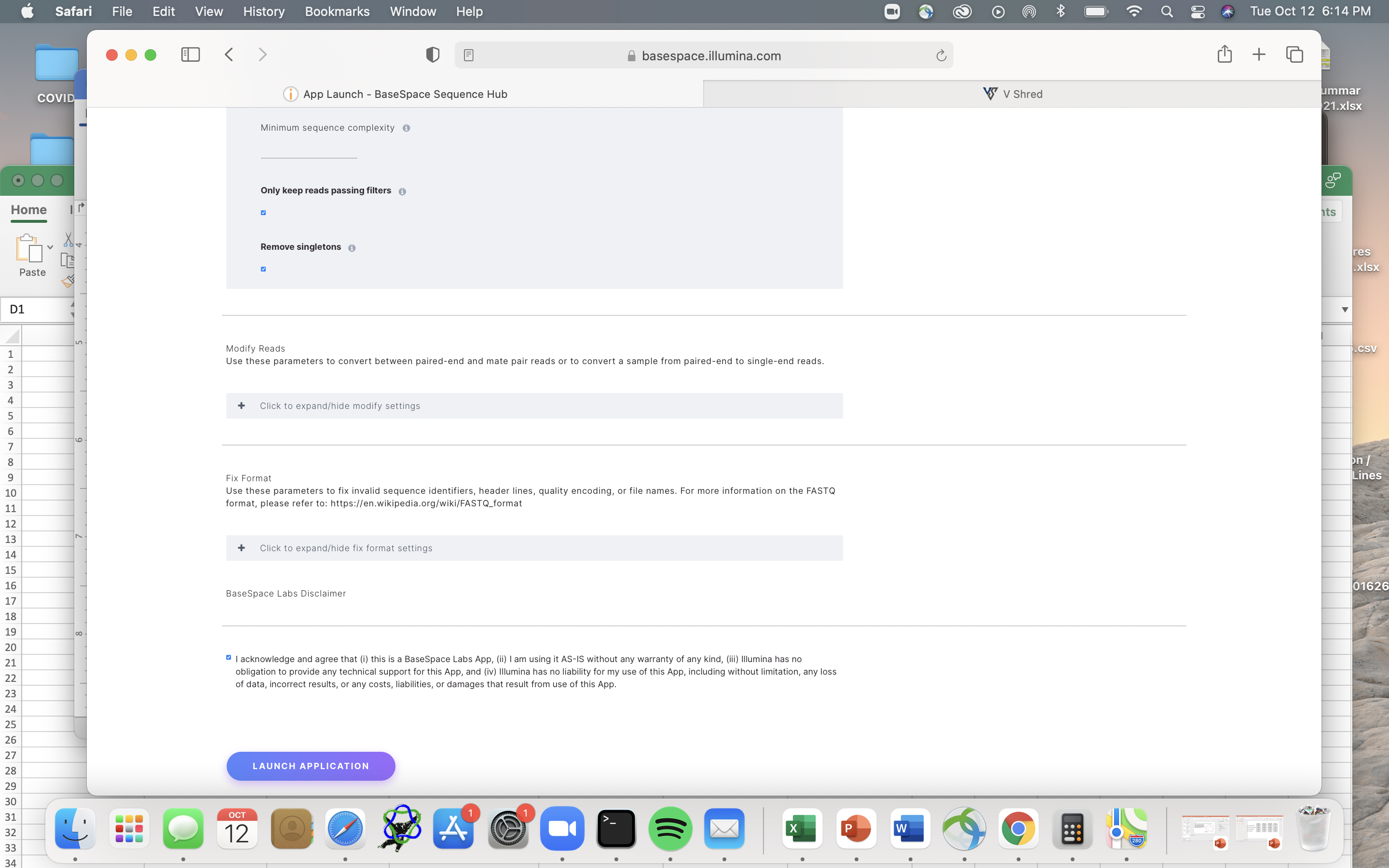


Once your reads are quality filtered and trimmed, you can recheck the quality with FastQC as described in steps 1 to 6 and use these reads for contig assembly.

**CONTIG ASSEMBLY**

9. Back on the SEQEUNCE HUB, go back to the “APPS” tab by clicking on it, looking for the SPAdes Genome Assembler program, selecting it like previously done in step 3, and launching the application as you did in step 4. SPAdes Genome Assembler is an open source tool for de novo sequencing. This application is designed to assemble small genomes from MDA single-cell and standard bacterial data sets.


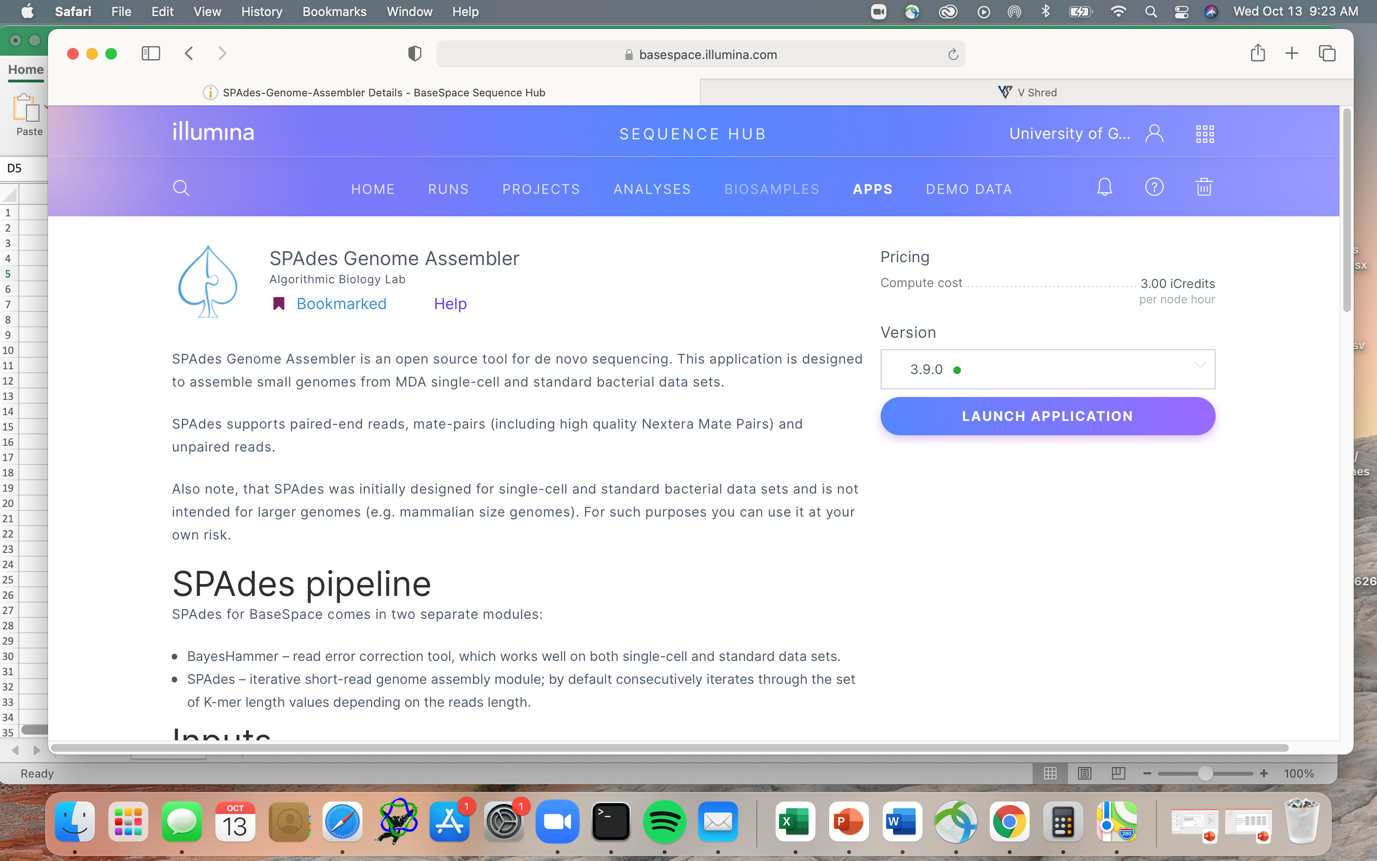

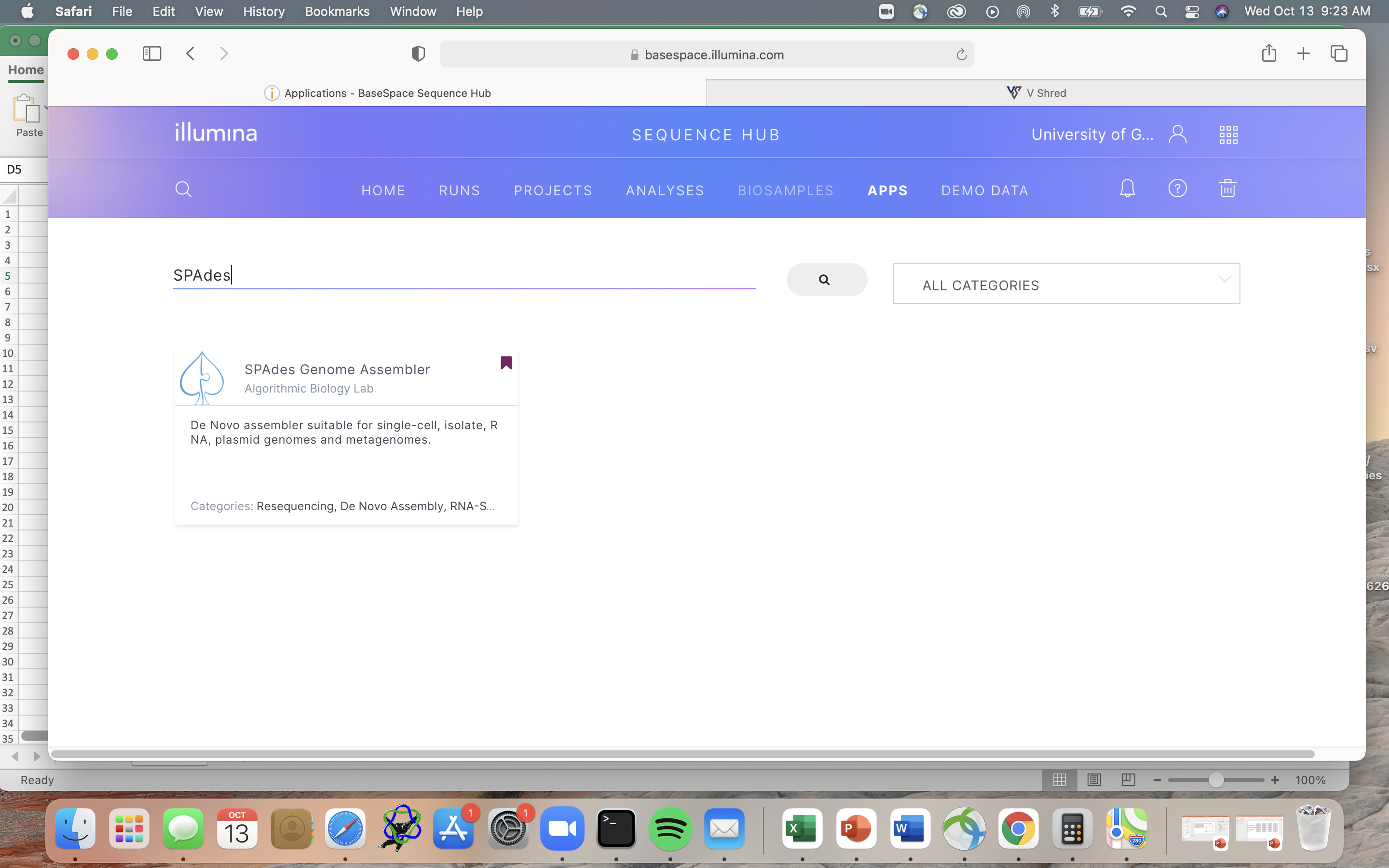


10. Once you have launched the application, you can change the “Analysis Name” by clicking on top of the default name. Under “Input,” click “Select Sample(s),” select the samples you want to analyze (trimmed filtered reads from the steps above) and click the purple “SELECT” button.


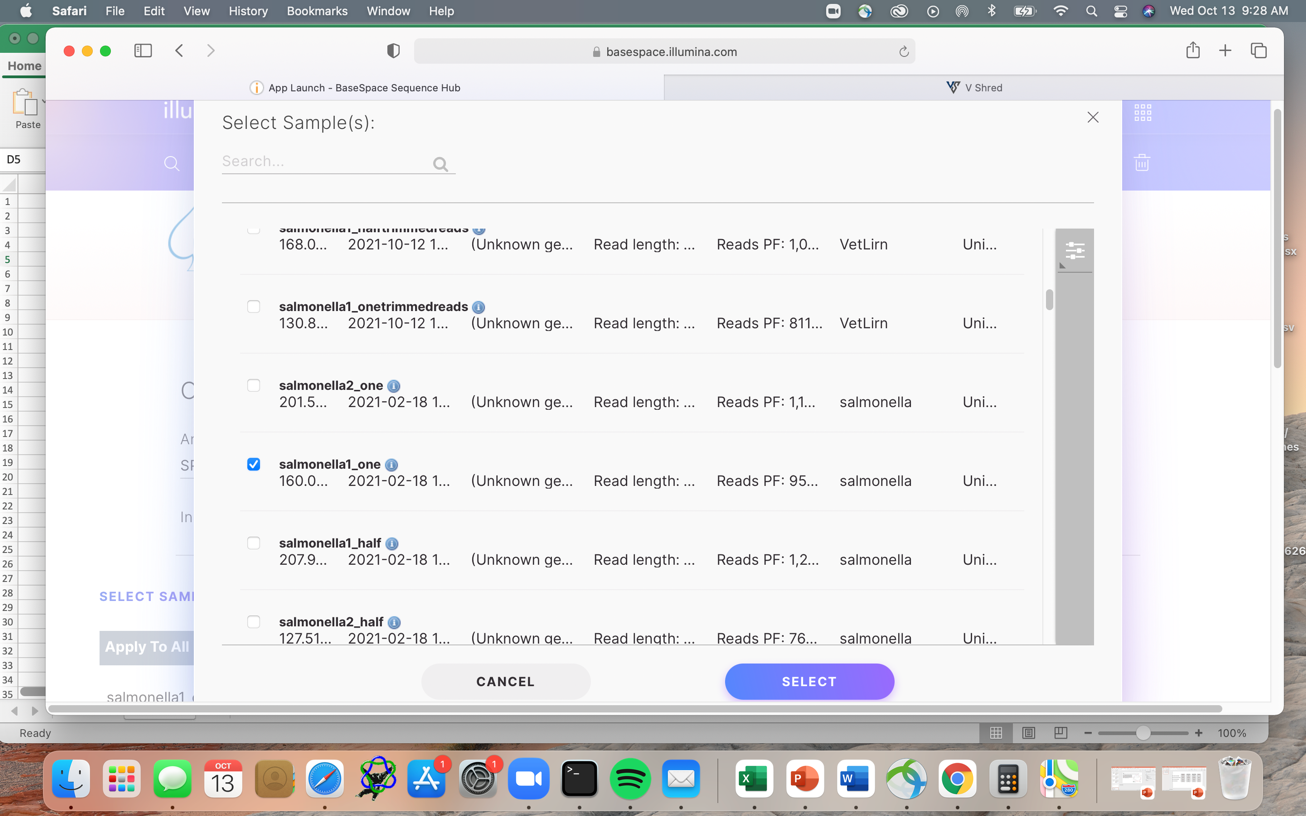

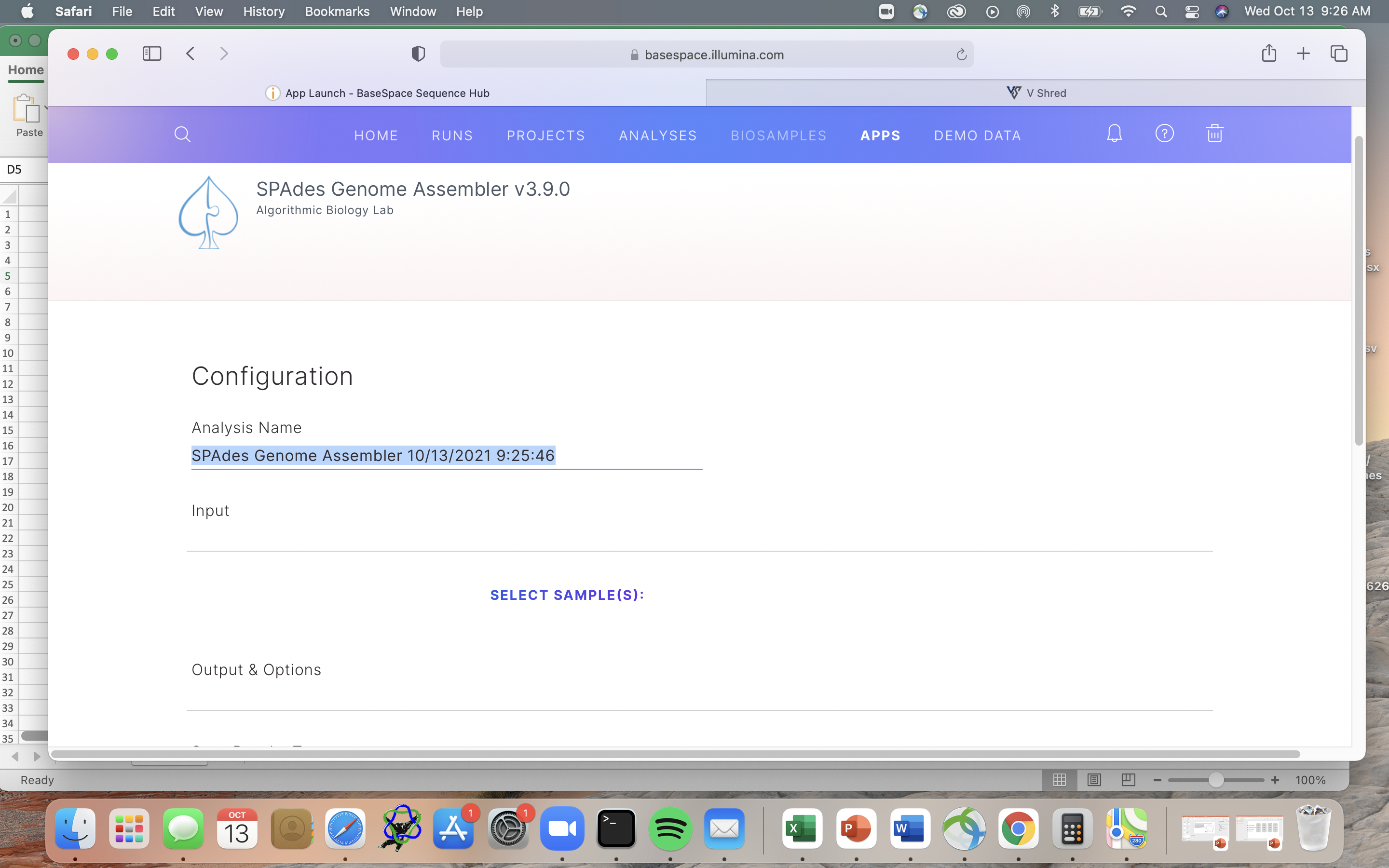


You will be able to see the selected sample under “Selected Biosample(S)”. Then, specify the type of library you used (paired-end, single, mate pairs, or nextera mate pairs), and library orientation (forward-reverse, reverse-forward, forward-forward, or reverse-reveerse).


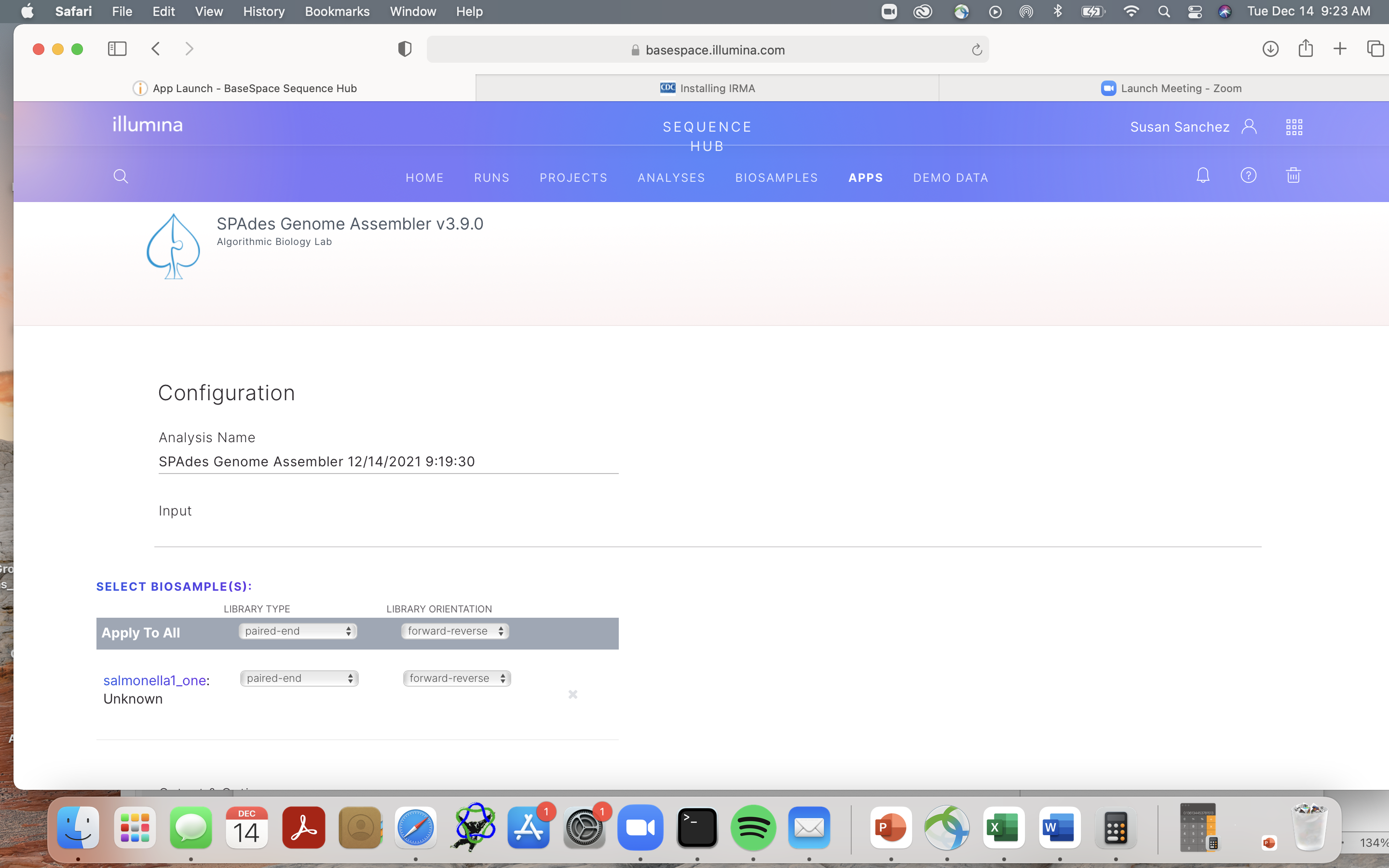


Finally, under “Output & Options,” select the project you would like your results to be saved on as you did in step 8. Then choose a Running Mode with “Error Correction & Assembly,” “Multi-Cell (Isolate)” as your Dataset Type, “Enable” Careful, and “auto” k-mer lengths. Accept the “BaseSpace Lab” acknowledgment when you are ready and click on the purple “LAUNCH APPLICATION” button.


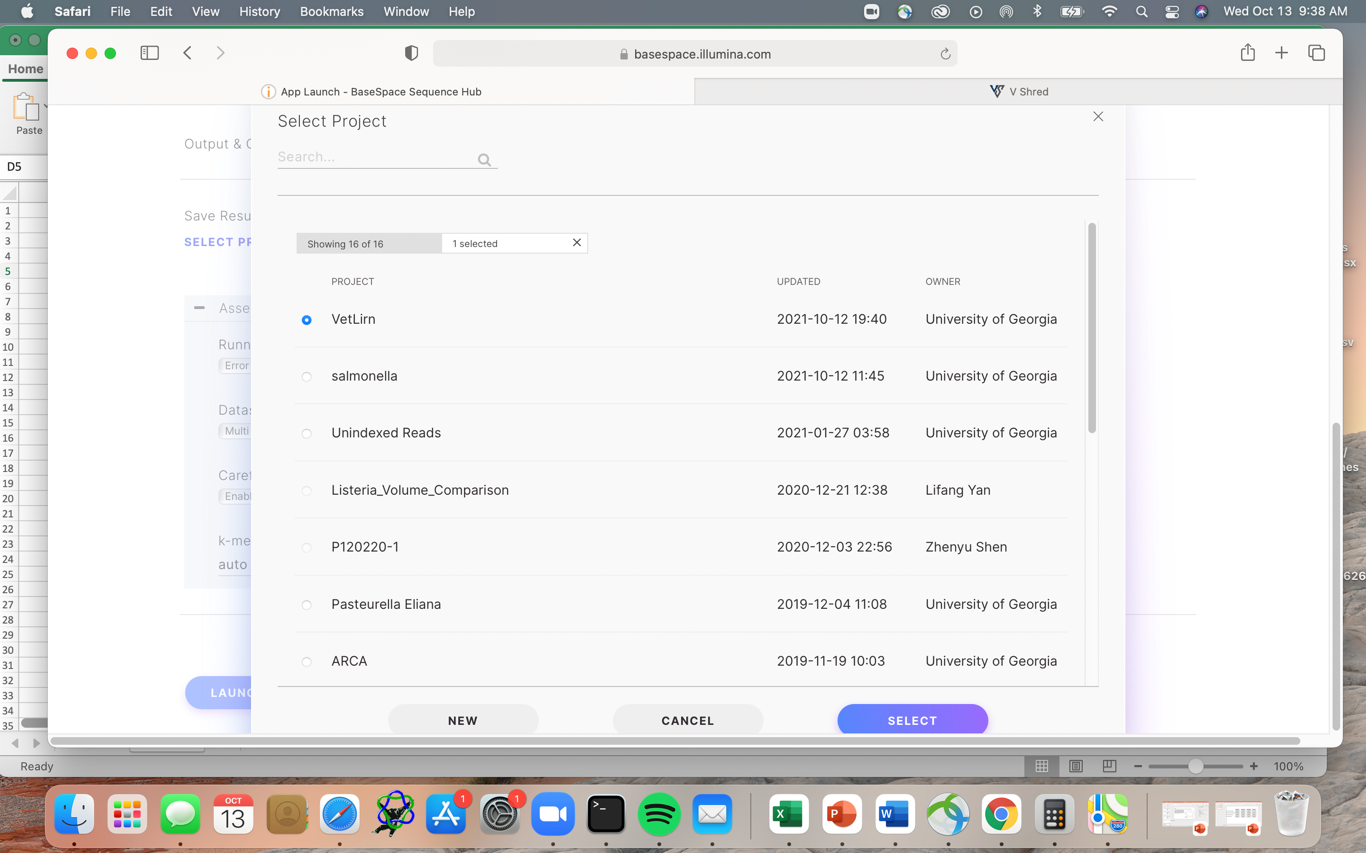

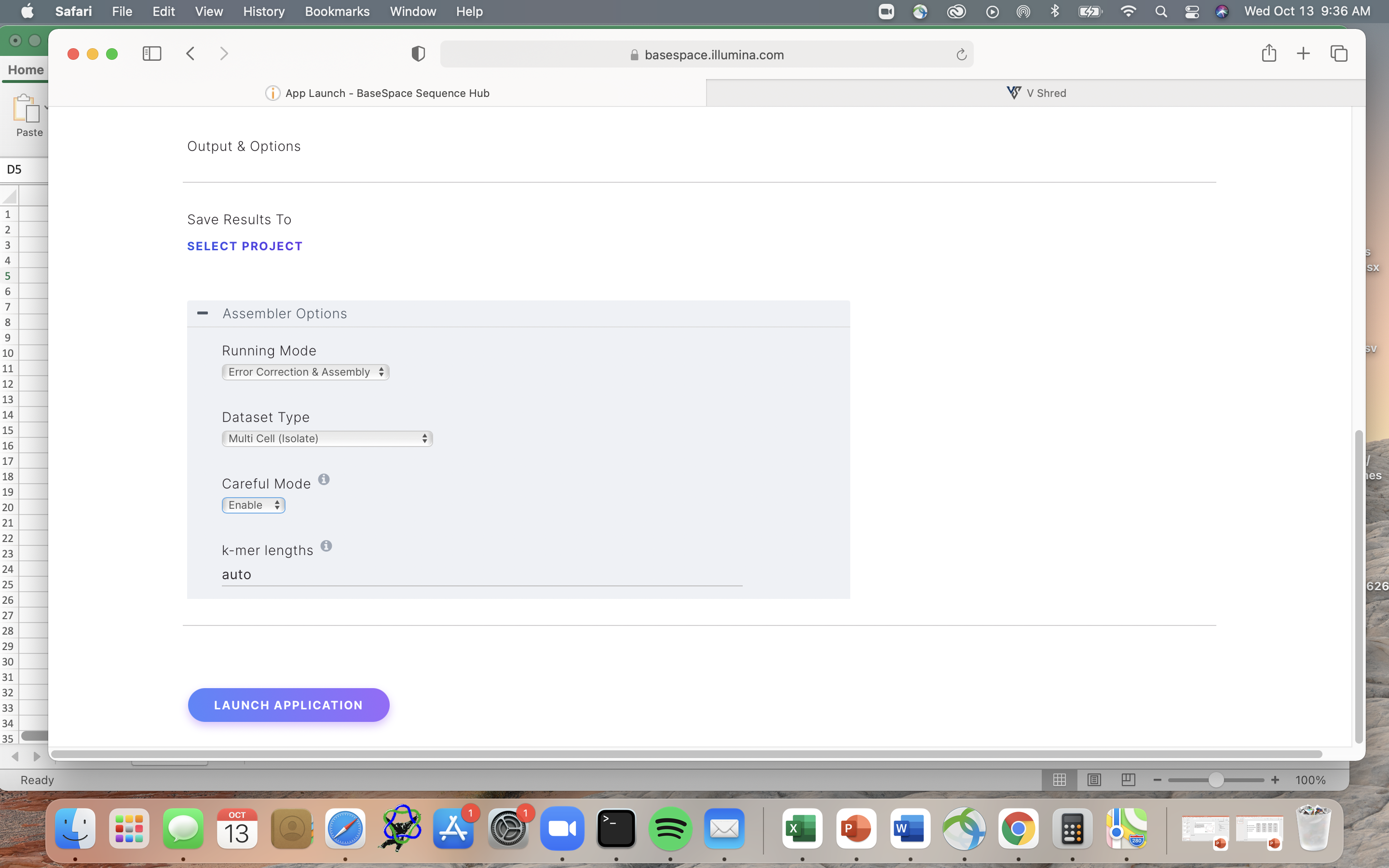


To access and/or download the assembly report go to the “Analyses” tab in Sequence Hub and click on your assembly.


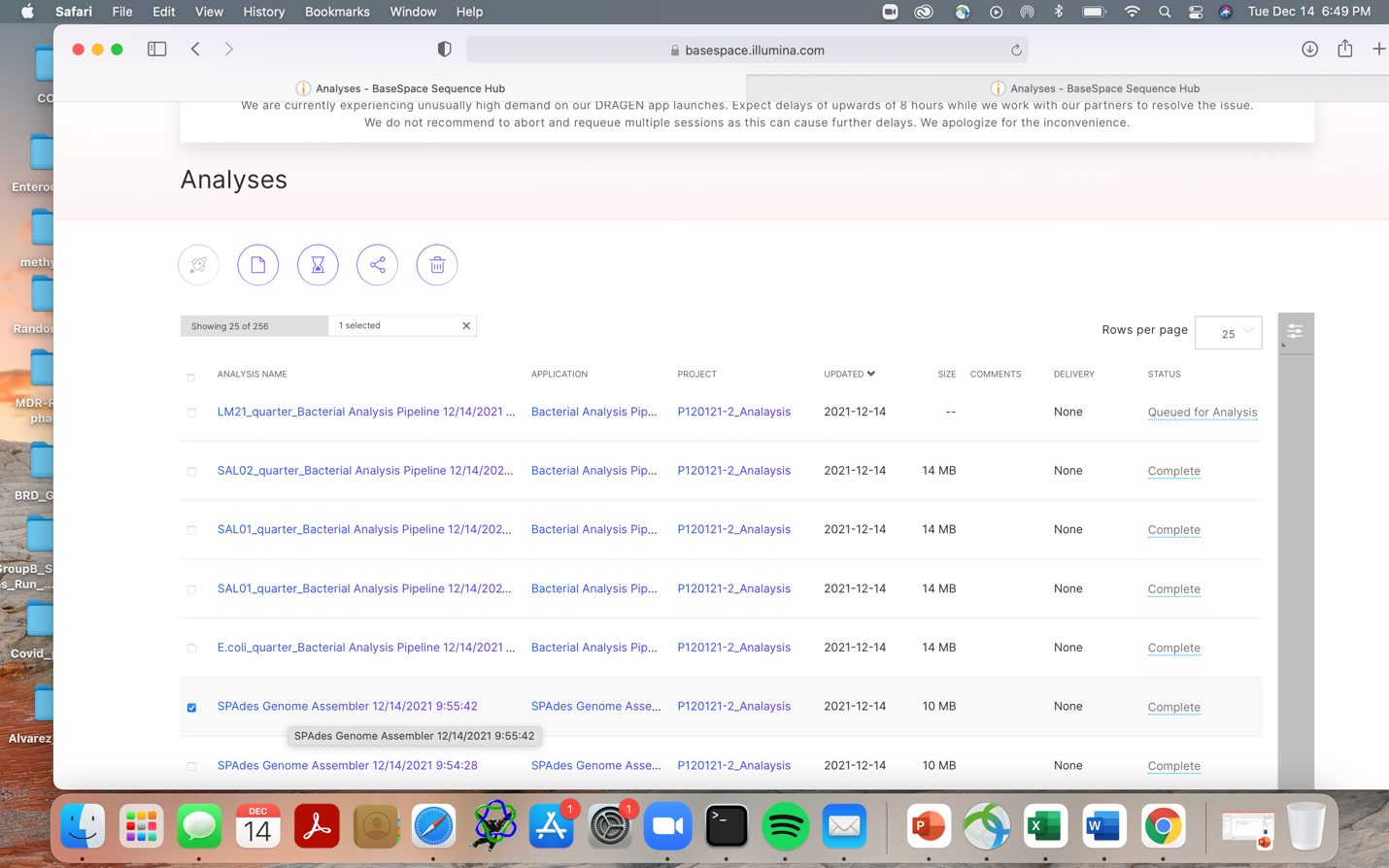

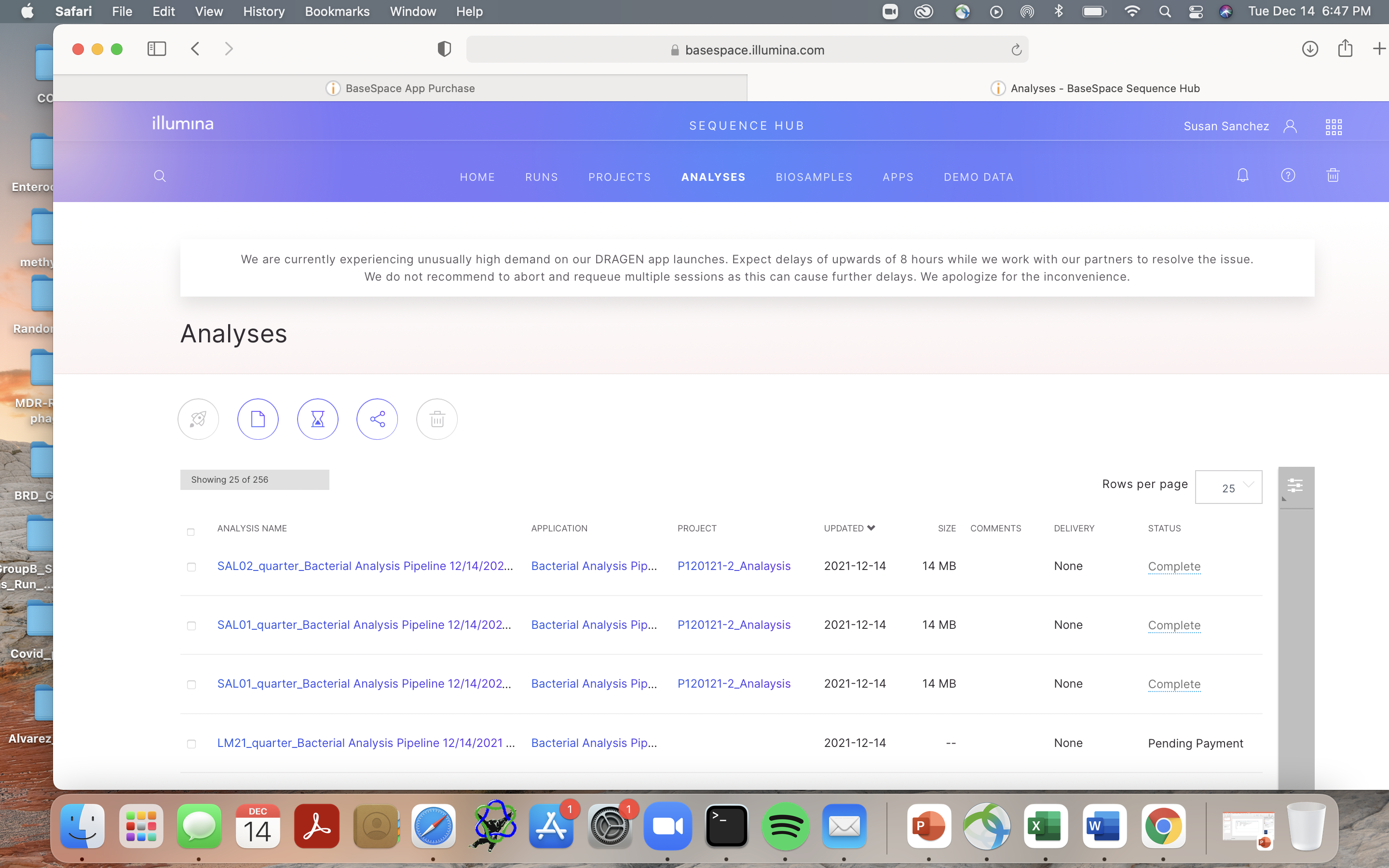


That will open a new window in which you can visualize the assembly report. To download the fasta file containing the genomic sequence of the bacteria that you just sequenced, click on “files”.


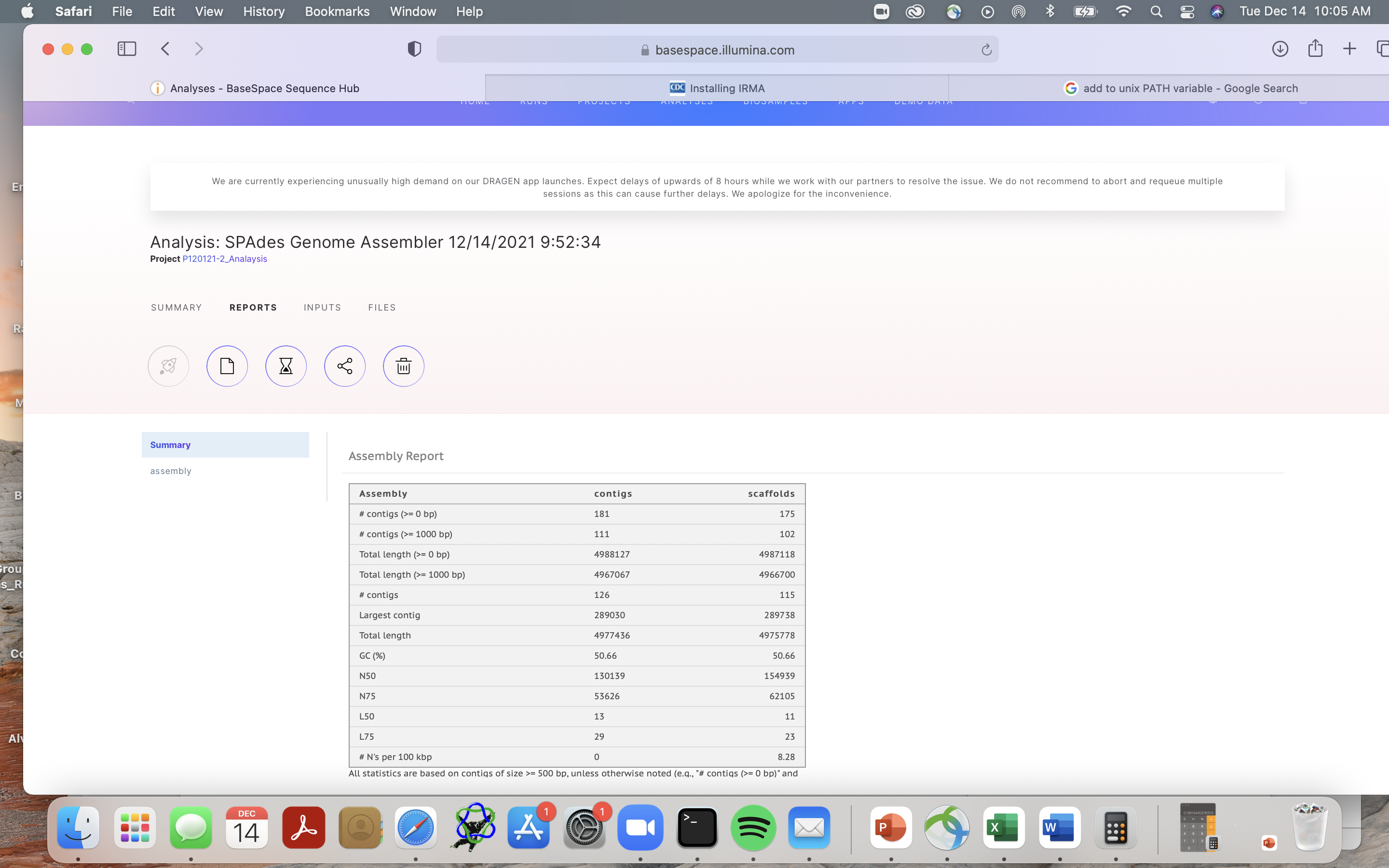


“# contigs (>=0bp)” is the total number of contigs in the assembly. This is the number of contigs that you will find in your contig.fasta file

“# contigs (>=10000bp)” is the total number of contigs with more than 1000 bps length in the assembly.

“Total length (>=0bp)” is the total number of bases in the assembly of all contigs

“Total length (>=1000bp)” is the total number of bases in the assembly of contigs bigger than 1000bps

“# contigs” is the total number of contigs with more than 500 bps length in the assembly, and what the program uses to run the rest of statitstics below.

“Largest contig” is the length of the longest contig in the assembly of contigs bigger than 500bps

“Total length” is the total number of bases in the assembly of contigs bigger than 500bps

“GC (%)” is the total number of G and C nucleotides in the assembly (of contigs bigger than 500bps), divided by the total length of the assembly (of contigs bigger than 500bps).

“N50” is the size of the shortest contig in the set of longest contigs that together cover at least 50% of the total Genome Size [the bigger the number, the better the quality of the assembly]

“N75” is the size of the shortest contig in the set of longest contigs that together cover at least 75% of the total Genome Size [the bigger the number, the better the quality of the assembly]

L50 is the number of contigs equal to or longer than N50 [the bigger the number, the better the quality of the assembly]

L75 is the number of contigs equal to or longer than N75 [the bigger the number, the better the quality of the assembly]

# N's per 100 kbp is the average number of uncalled bases (N's) per 100,000 assembly bases. [the lower the number, the better the quality of the assembly]

This same applied to the scaffolds, that are bigger contigs. You can use either or the files (contigs.fasta and scaffold.fasta) for species and ARGs identification.

A new windows will open, click in “assembly”.
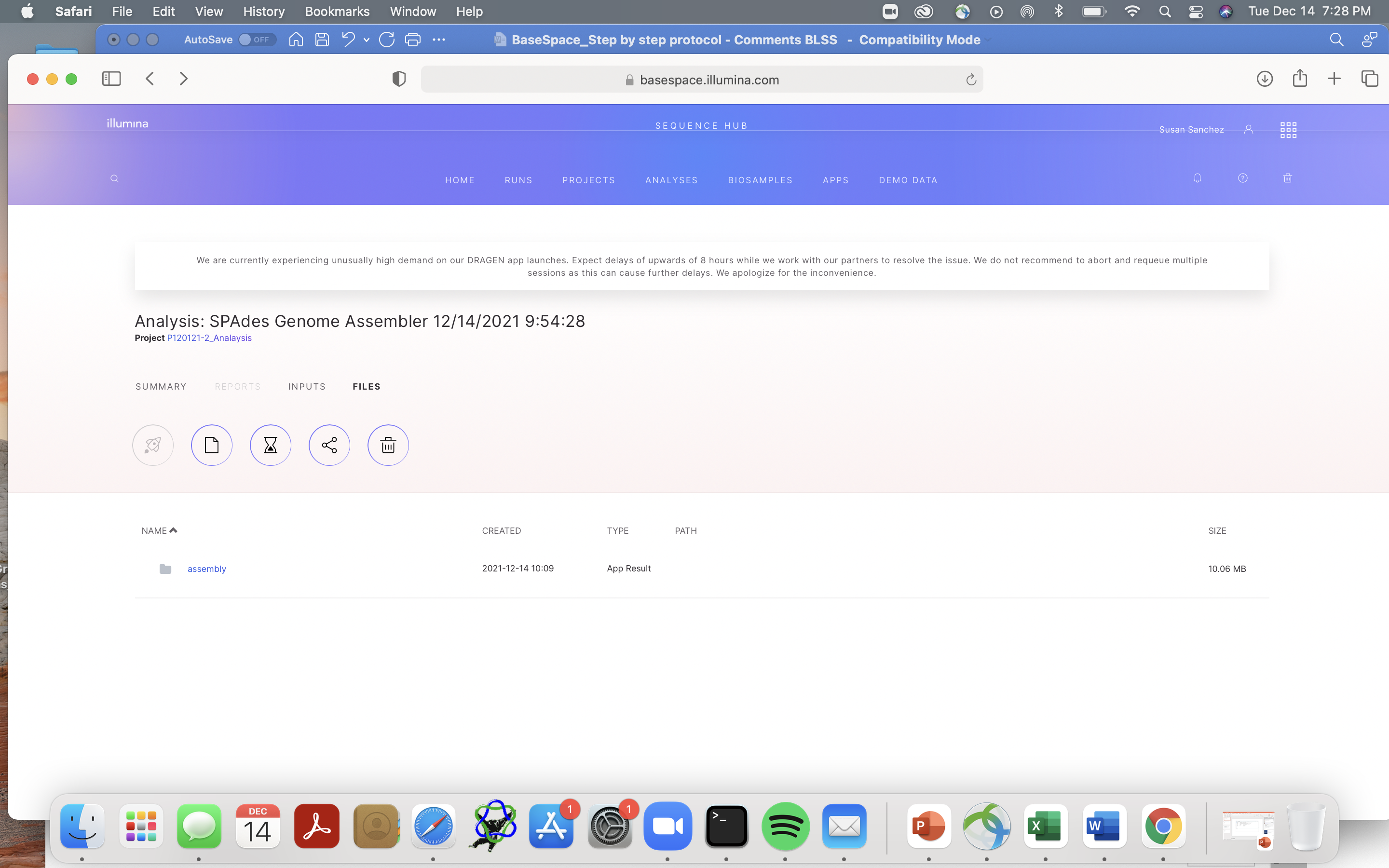


A new windows will open, click in “contigs”. That wil open a smaller window. Click the purple “download” icon and the file will download automatically. You can use that fasta file to perfrom bacteria species and amtimicrobial gene identification using any pipeline or platform of your choice.


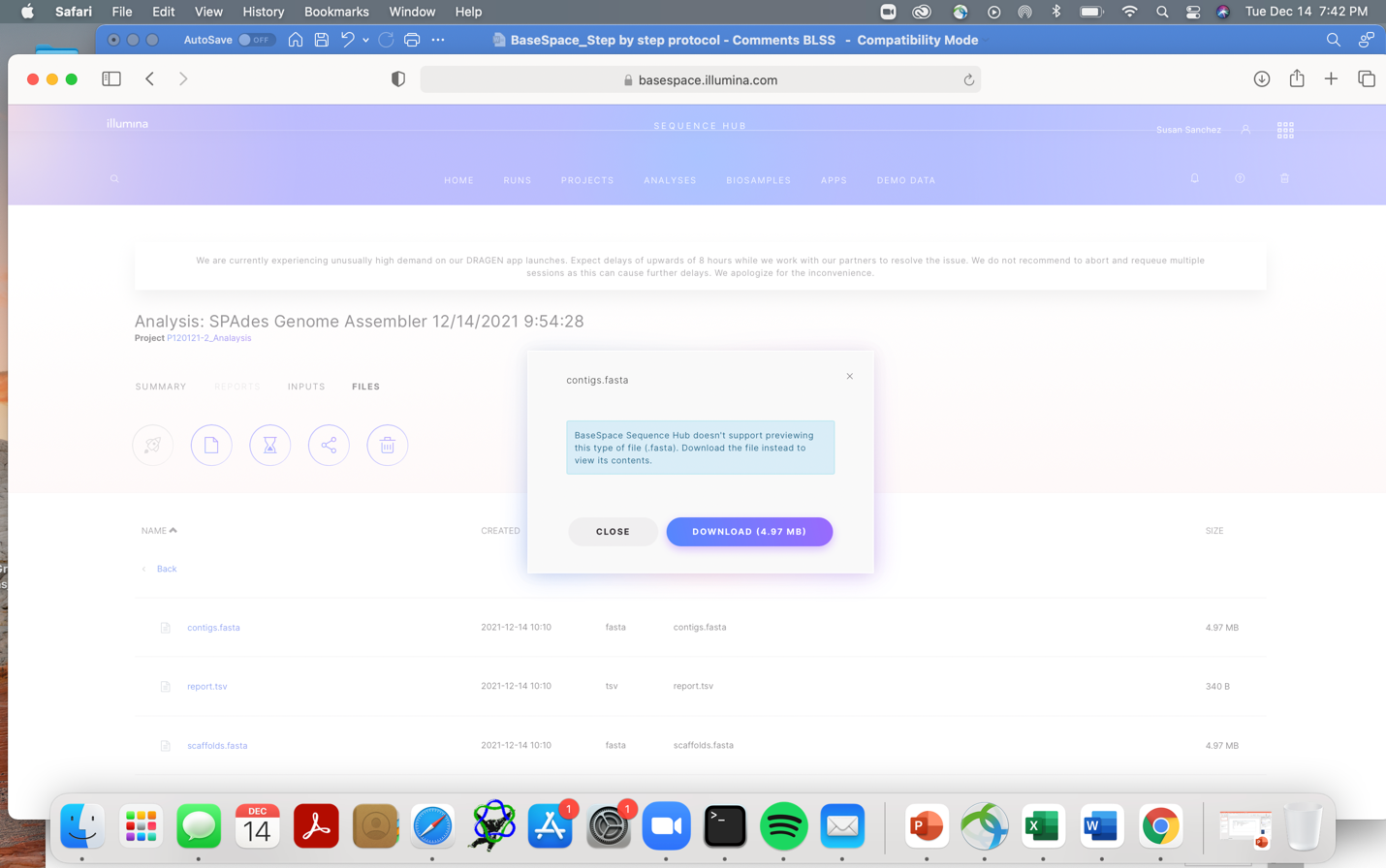

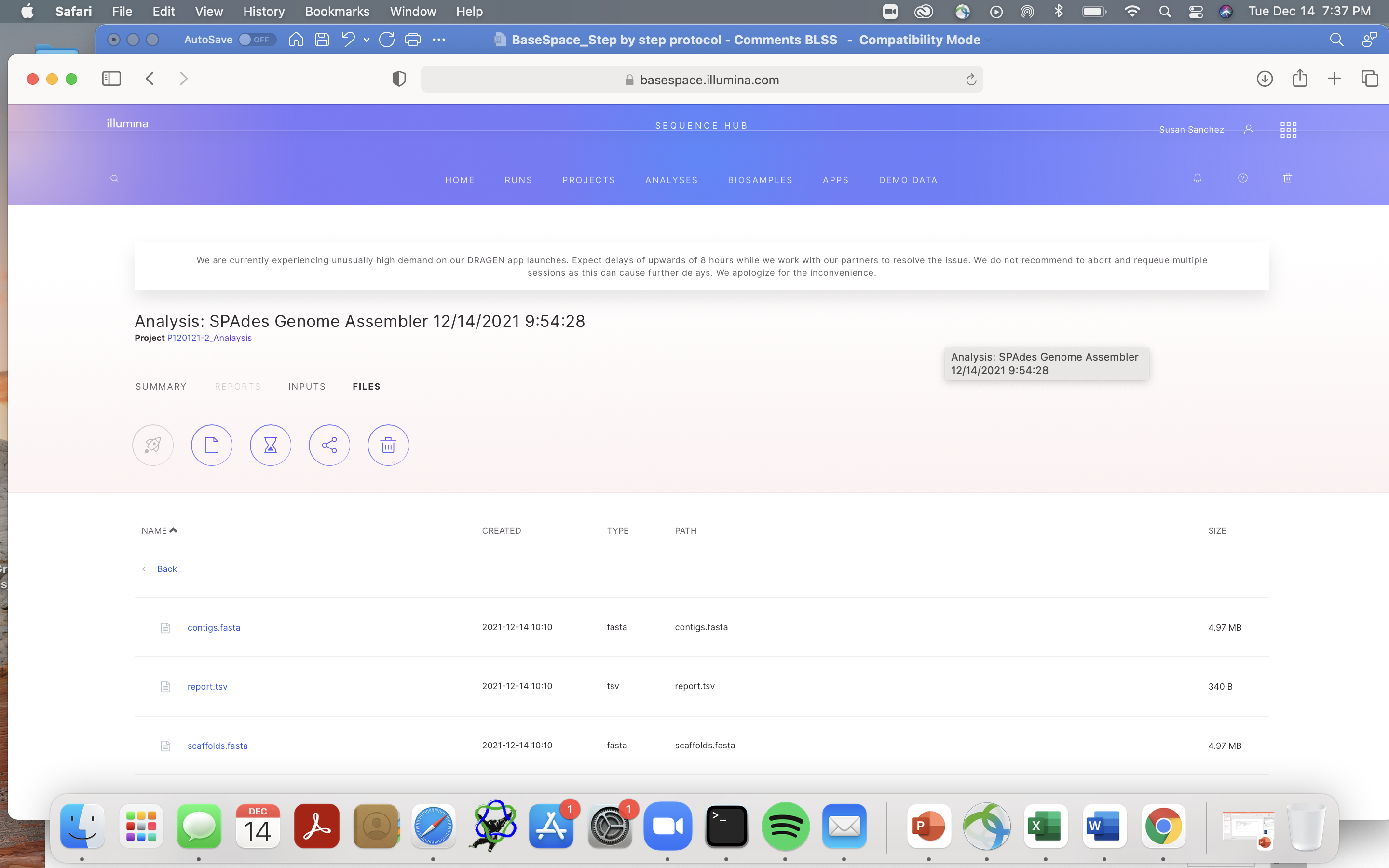


**BACTERIA SPECIES AND ARG IDENTIFICATION**

11. Back on the SEQEUNCE HUB, go back to the “APPS” tab by clicking on it, looking for the Bacteria Analysis Pipeline, selecting it like previously done in step 3, and launching the application as you did in step 4. The Bacterial Analysis Pipeline app predicts the species and ARGs of bacterial input genomes using a kmer-based approach.


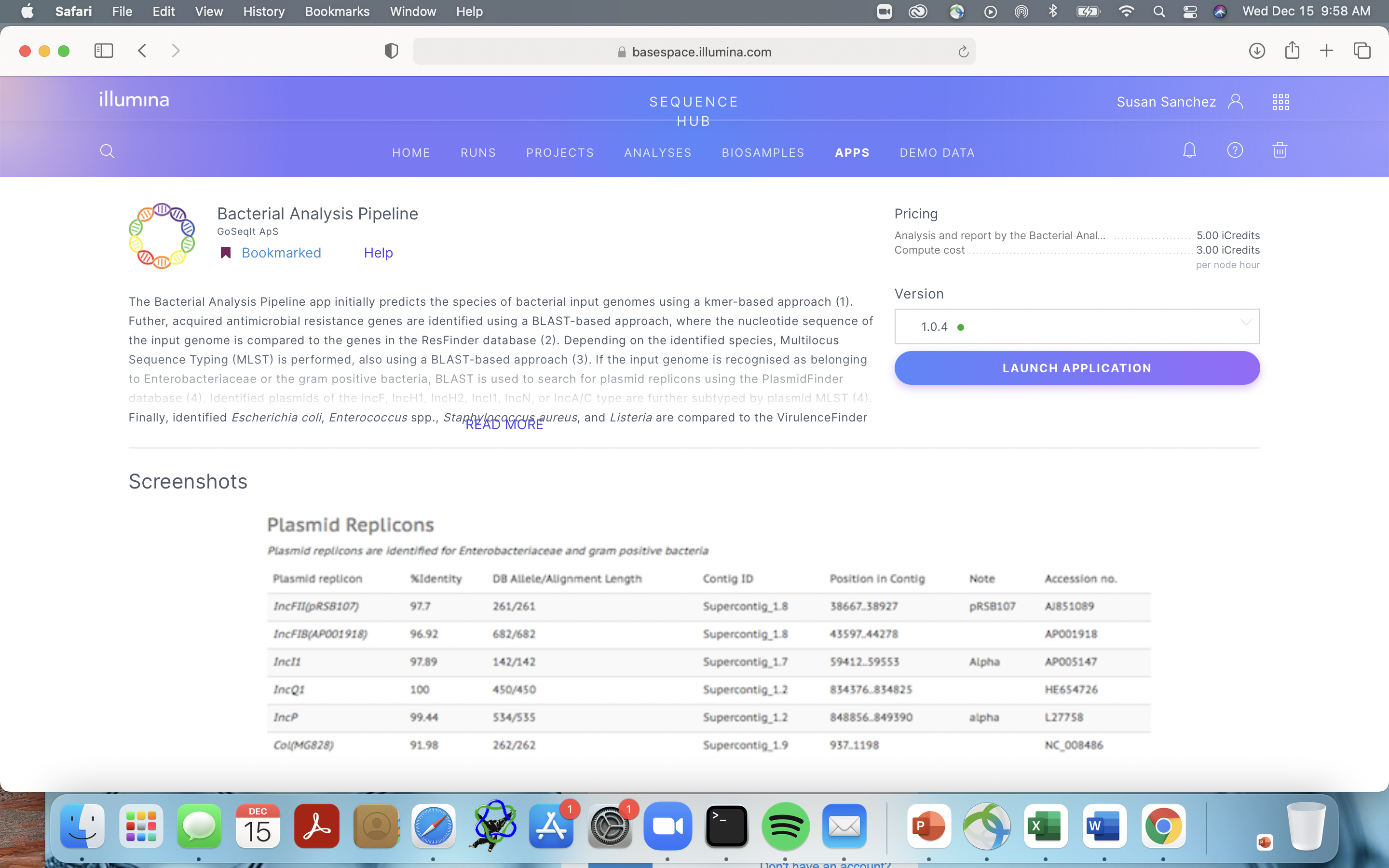


Once you have launched the application, you can change the “Analysis Name” by clicking on top of the default name. Under “Input file” click “Select Dataset file(s),” select the sample you want to analyze (contig fasta file form the step above) and click the purple “SELECT” button.


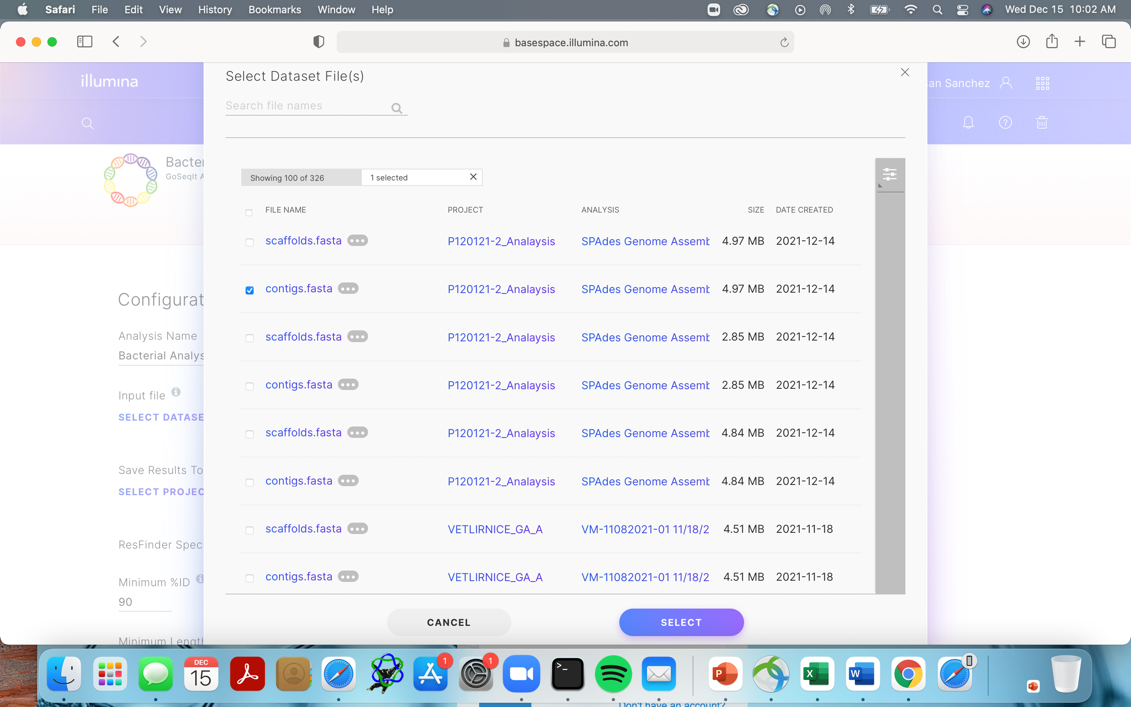

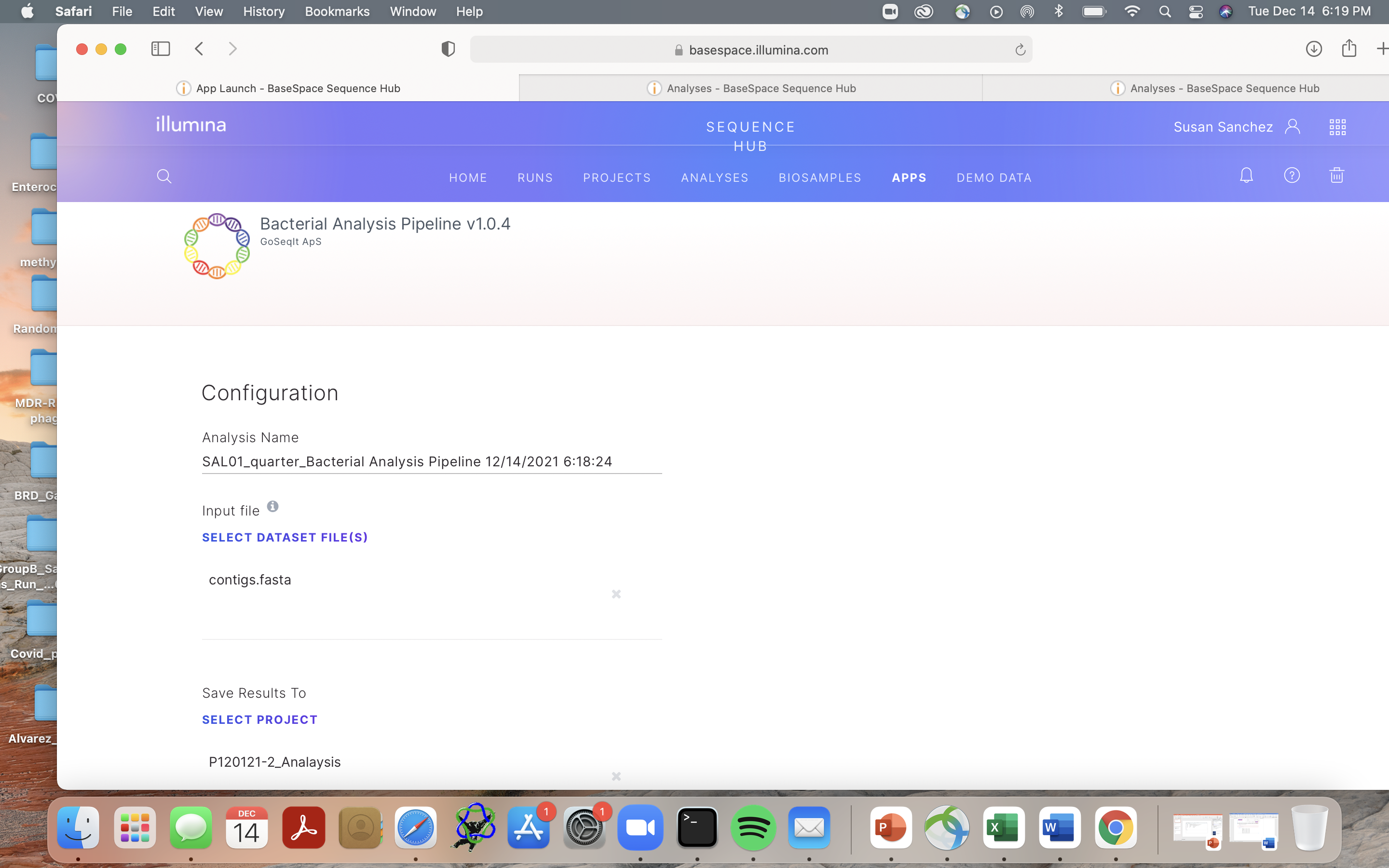


Under “Save Results To”, select the project you would like your results to be saved on as you did in step 8. Leve the “ResFinder, PlasmidFinder and VirulenceFinder Specific Settings” as default. Accept the “BaseSpace Lab” acknowledgment when you are ready and click on the purple “LAUNCH APPLICATION” button. This application requires 5 iCredits for isolate aditional to the running price.


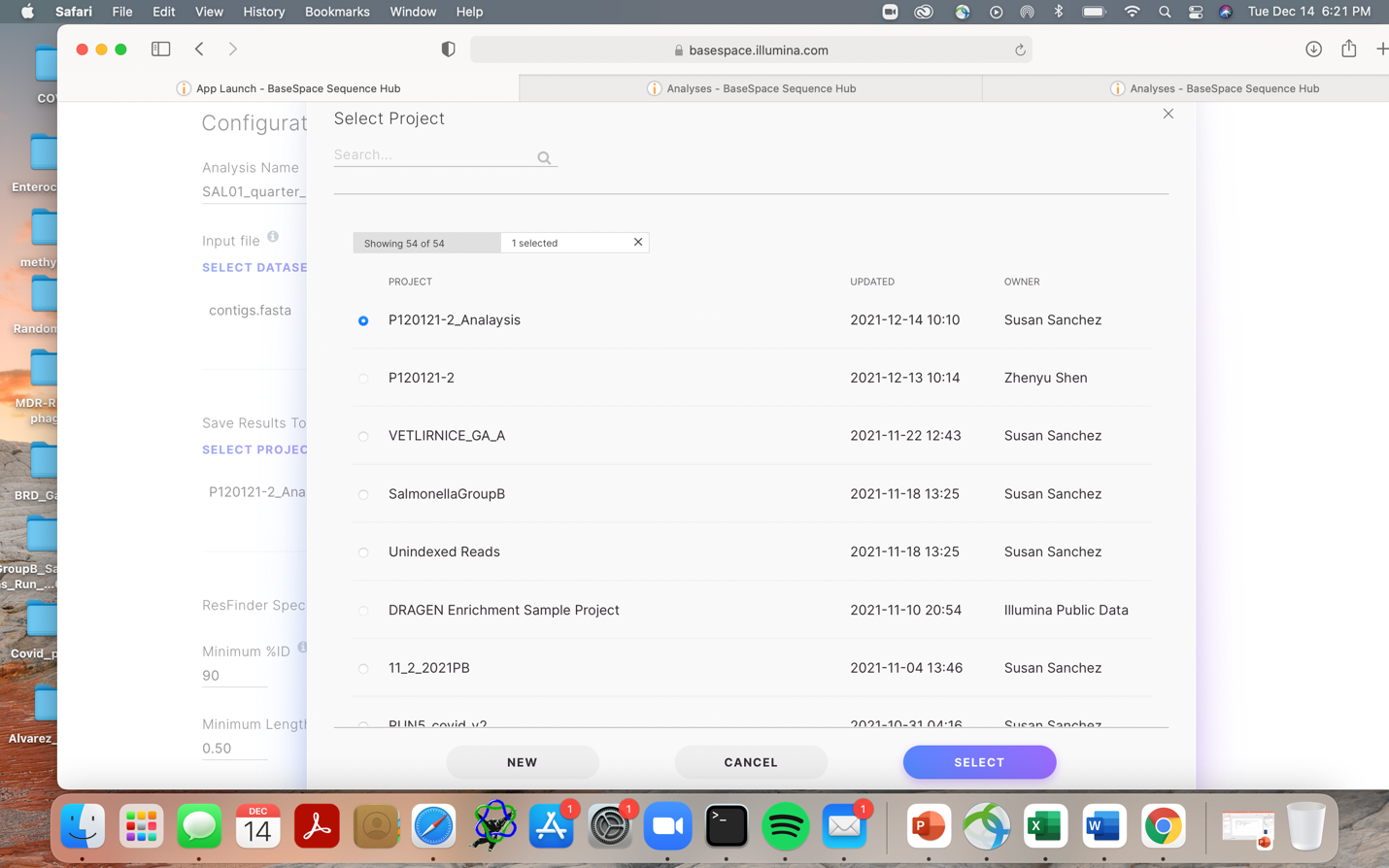

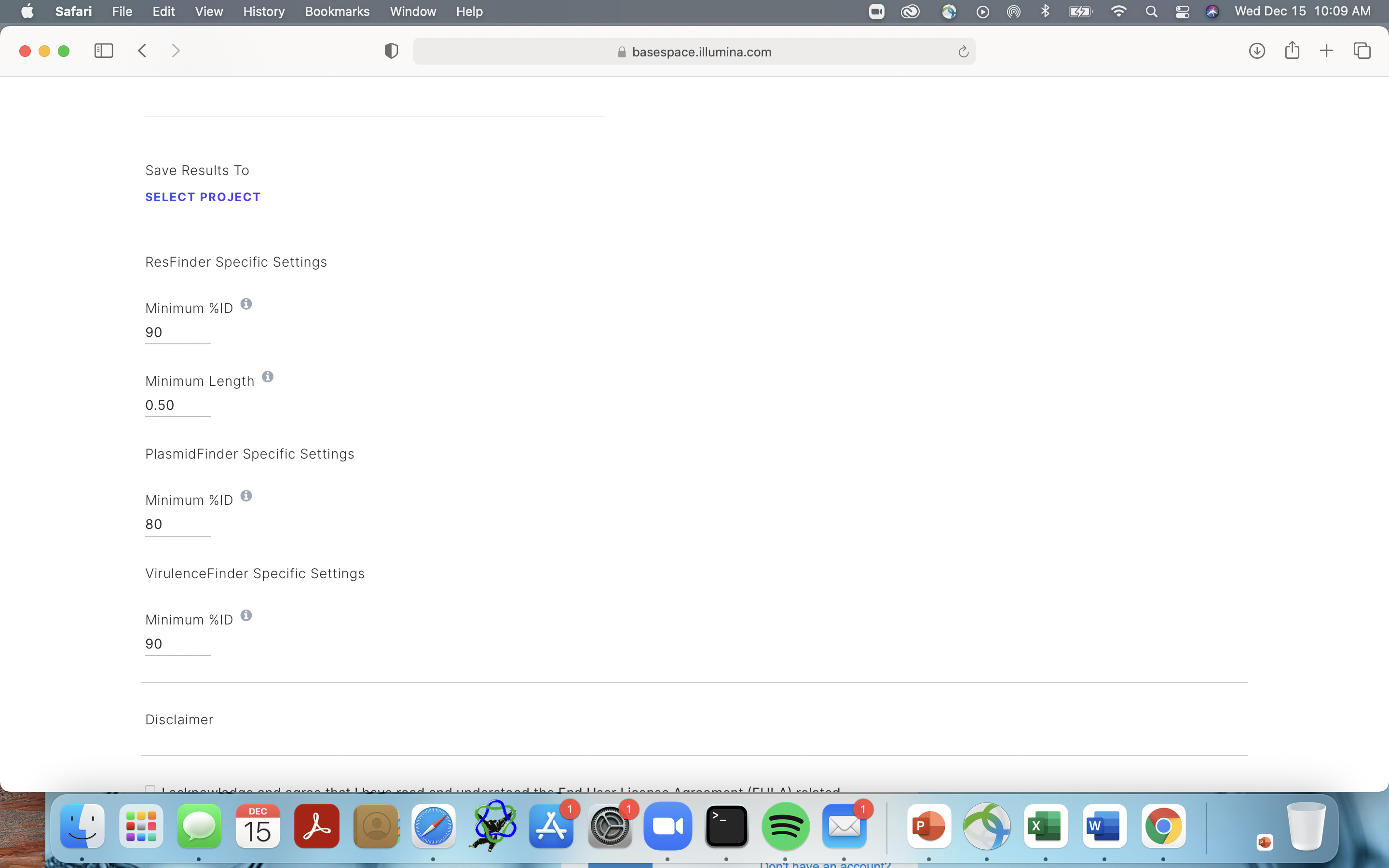


To access and/or download the Bacterial Analysis Pipeline report go to the “Analyses” tab in Sequence Hub and click on the analysis for your sample.


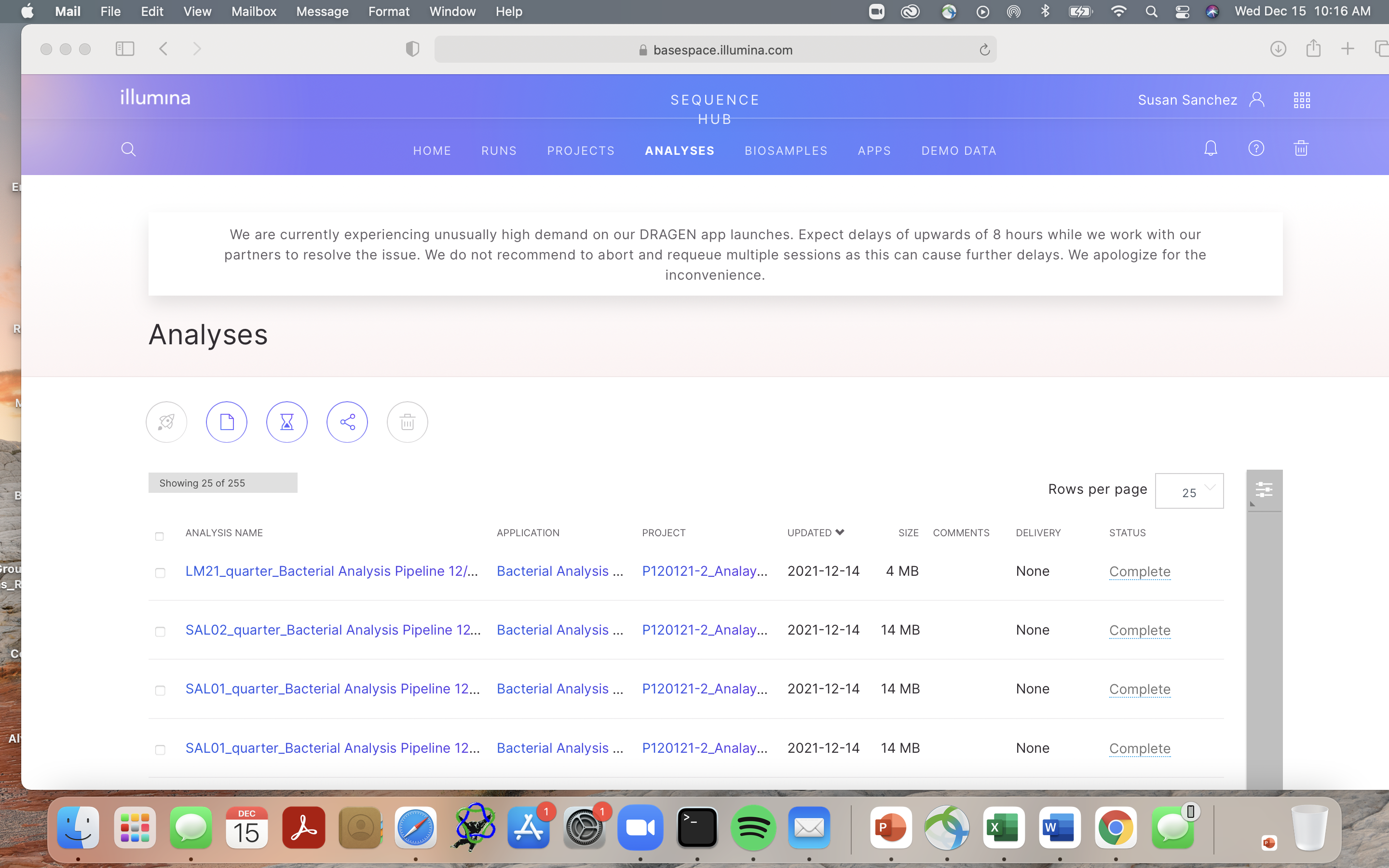


One inside the Analysis of your sample click in “Reports” to visualize the report with all the information about your sample.


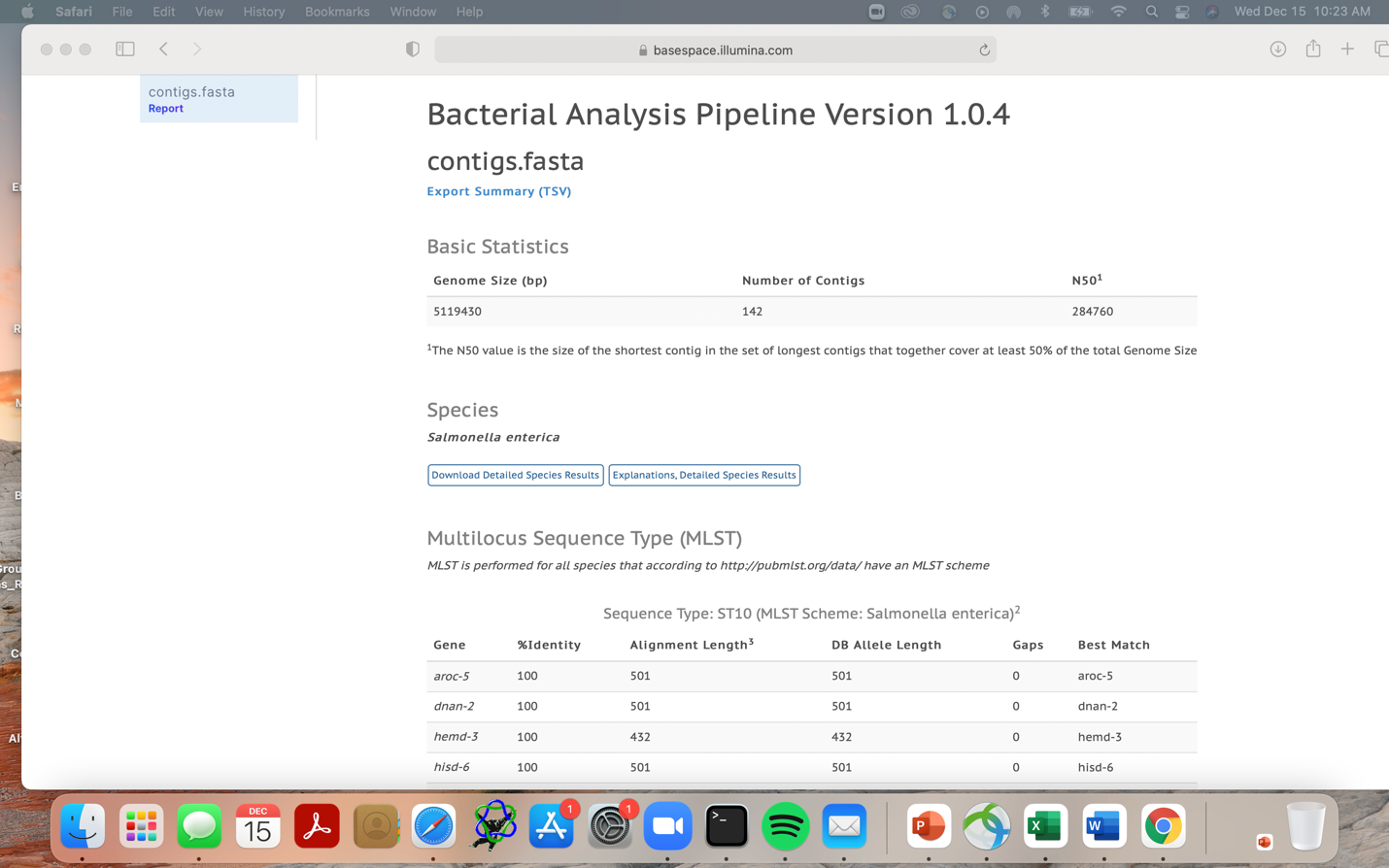

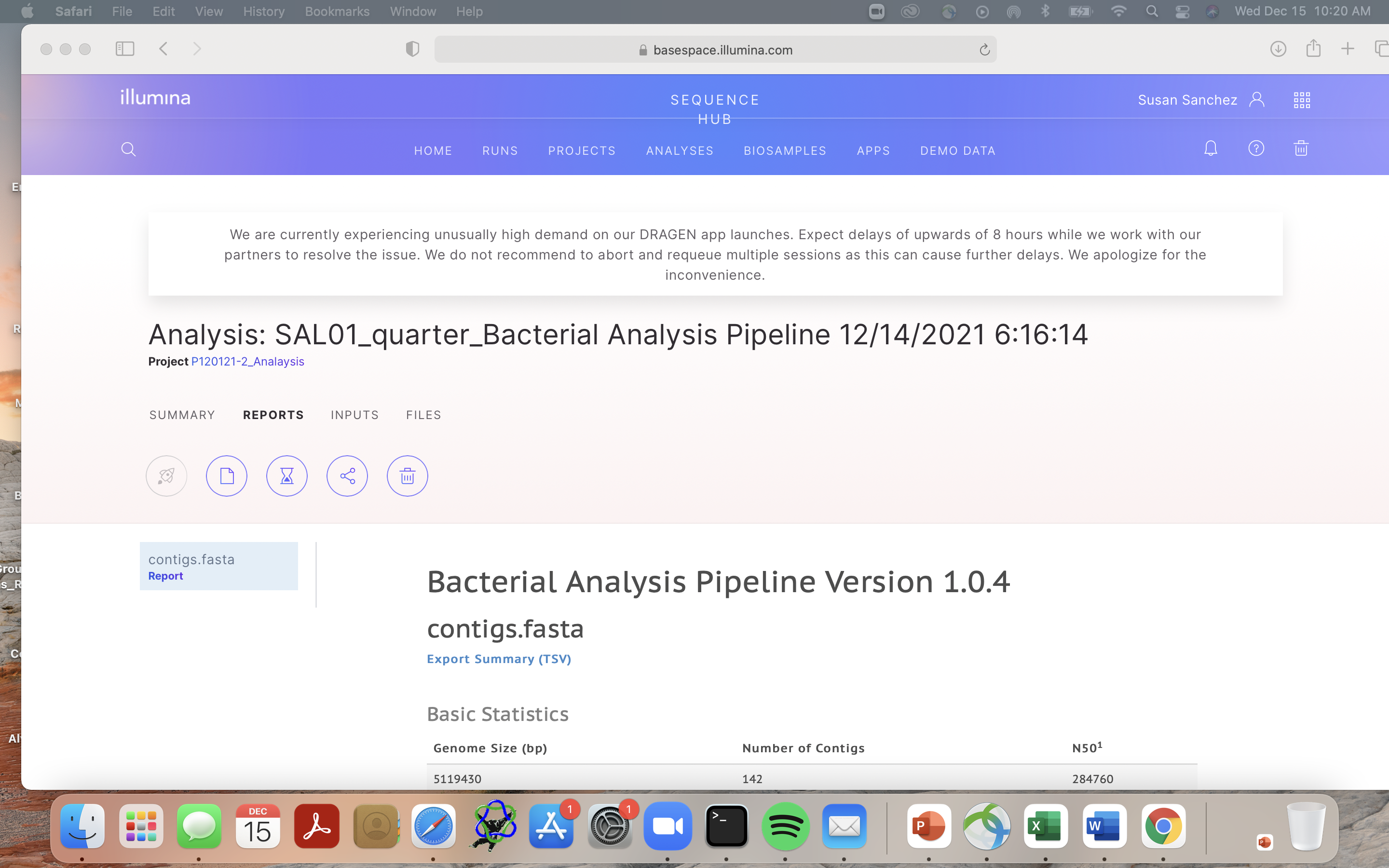


In your report, “Basic Statistics” shows you how long the genome of your bacteria is (Genome Size), how many contigs were used to assemble the genome (Number of Contigs) and the N50.

Under “Species” you will find the species identified by the software, in this case Salmonella enterica.

Some bacteria have extra in-silico genotyping methods such as “Multilocus Sequence Typing or MLST” for further genetic classification. In the example, the software identified this S. enterica to belong to the Sequence Type or ST10.


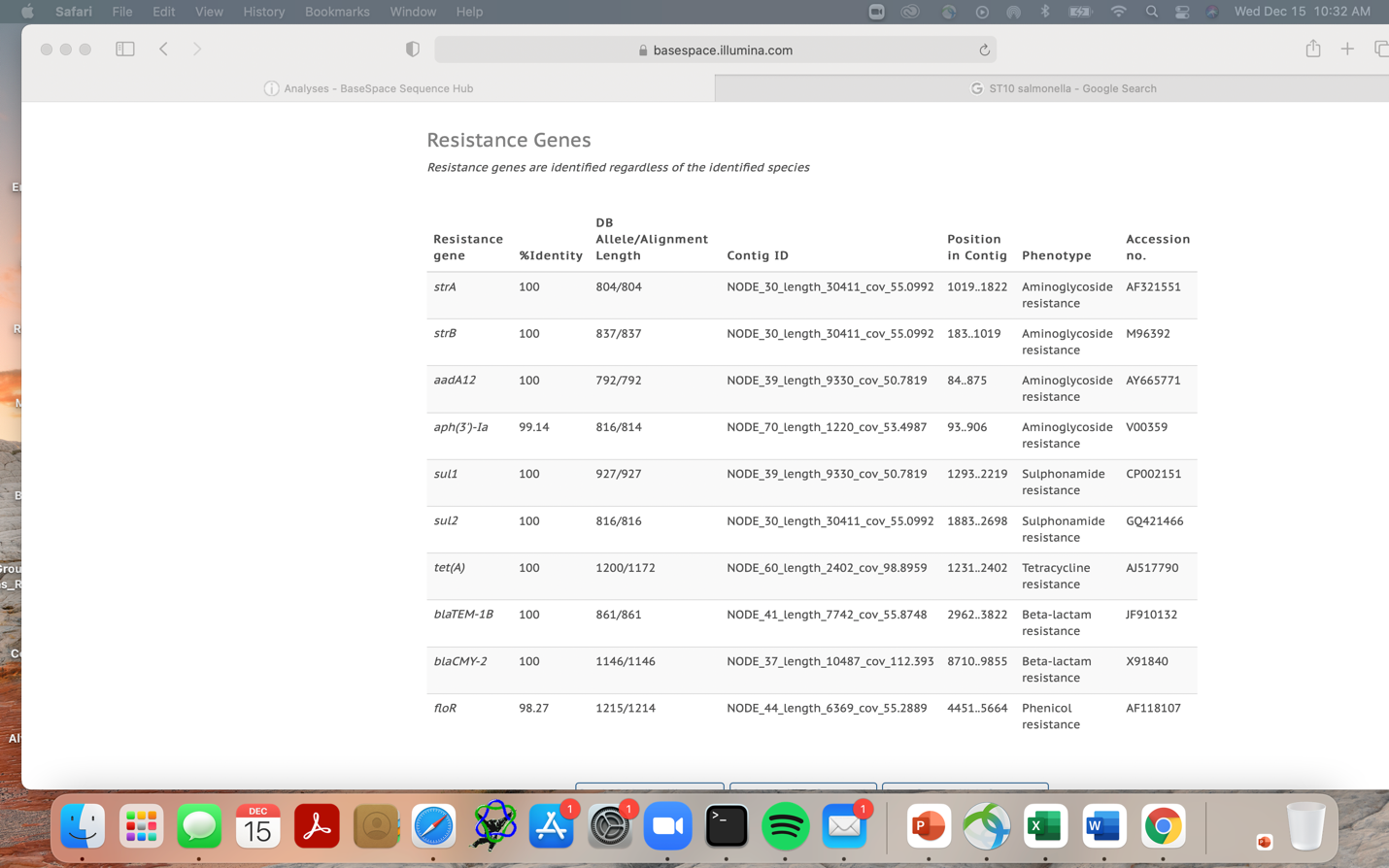
Under “Resistance Genes”, you will find a list with the ARGs identified by the program (Resistance gene), the “% identity” with the ARGs stored in the data base, how long is the gene in the Database (DB Allele) and how much of the gene is covered by the newly identified gene (Alignment length), the contig in your genomic sequence where this resistance gene was identified (Contig ID) and the specific location in bps (Position in Contig), the resistance “Phenotype” and the GeneBank “Accession no.” of the reference gene.


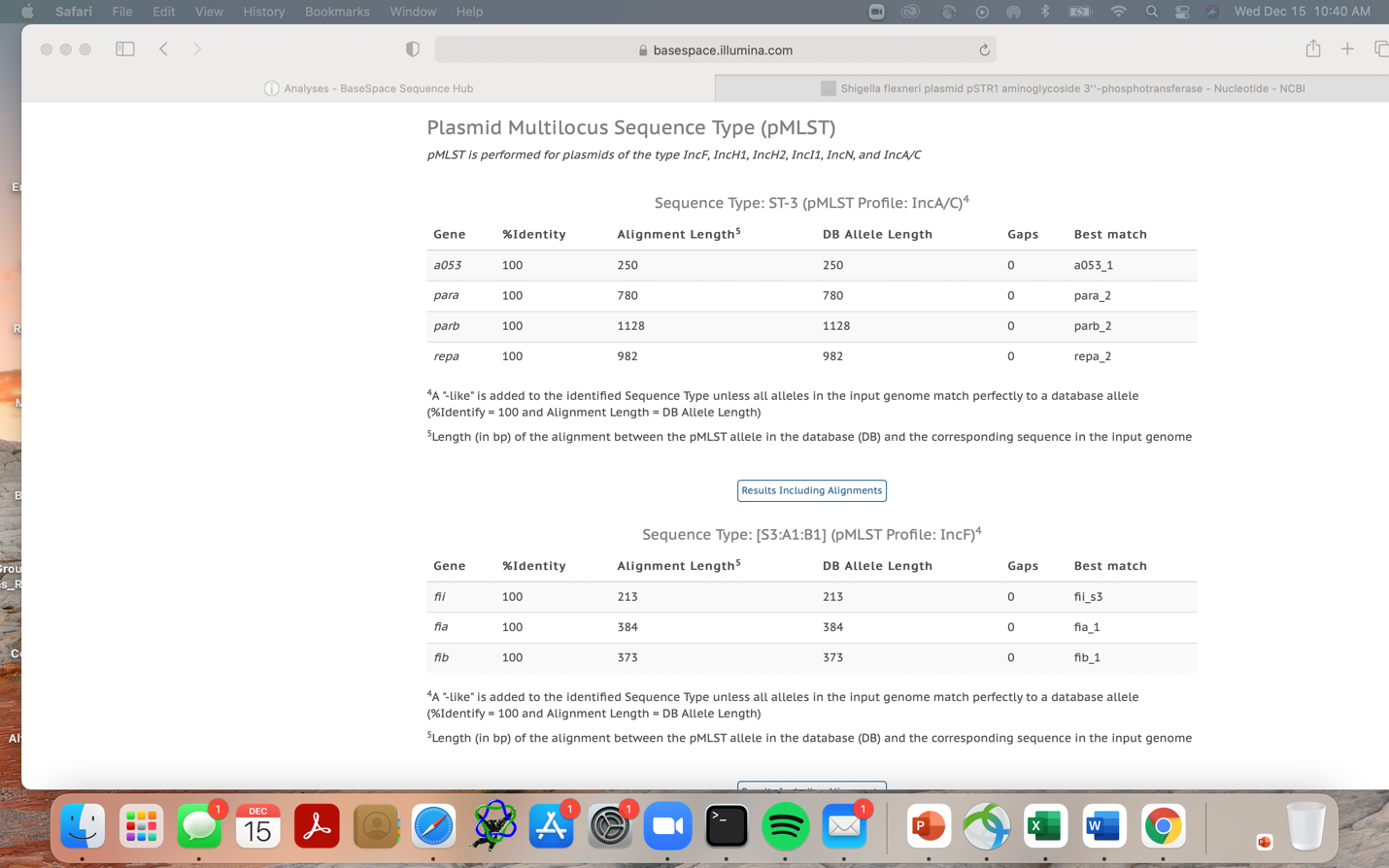


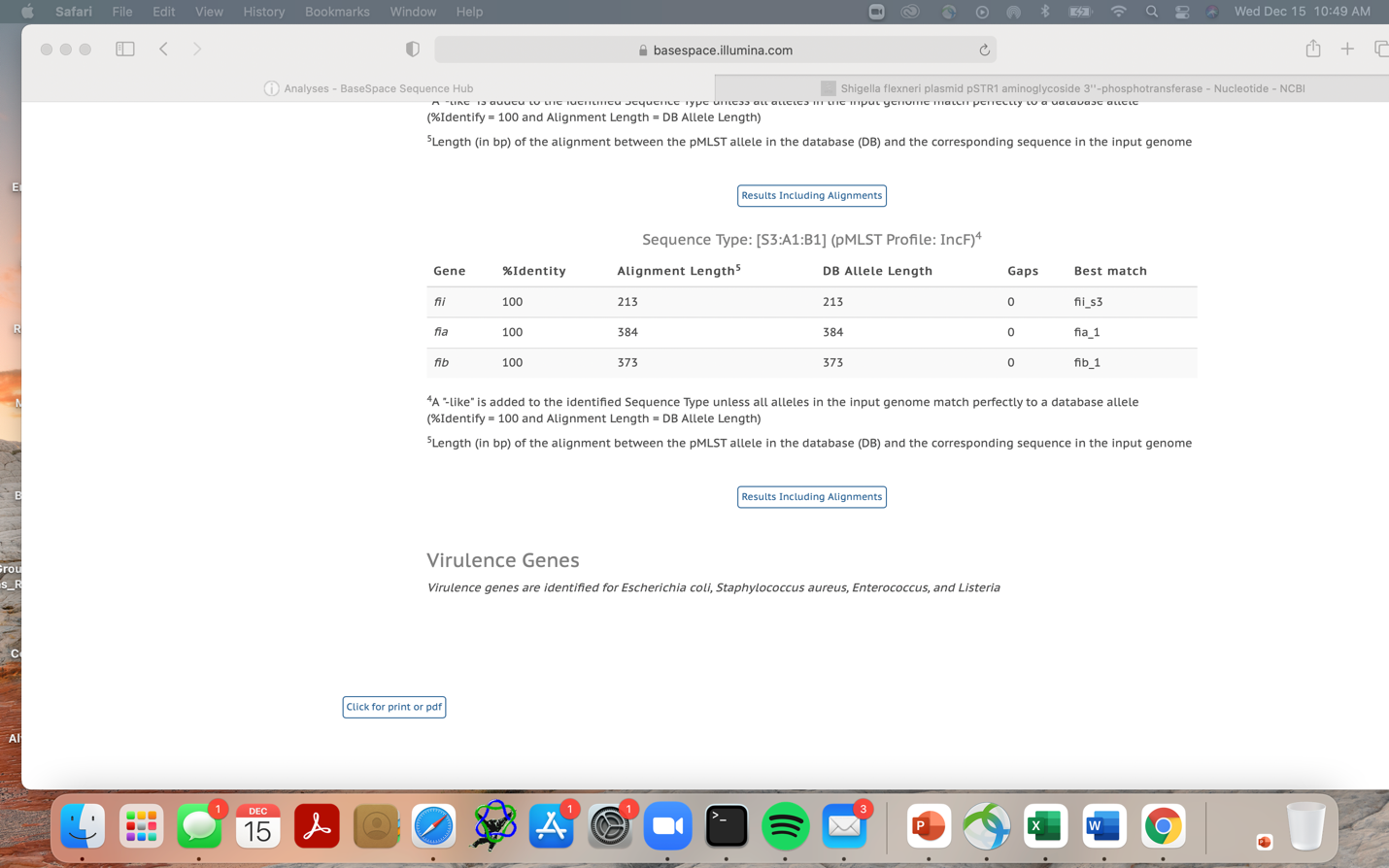
Additionally, the Bacterial Analysis Pipeline gives you information about potential extrachromosomal elements or plasmids and virulence genes present in your sample. Specifically, for each plasmid it will give you a list of genes involved in the classification of that plasmid. As above, the report shows “Gene” name, “% Identity” with the plasmid-gene stored in the data base, how long is the gene in the Database (DB Allele Length) and how much of the gene is covered by the newly identified gene (Alignment length), potential “Gaps” in the alignment and the identity of the database gene (“Best match”).

A similar table will appear under “Virulence Genes” if they are identified.

Finally, if you want to download or print this report, “Click for print or PDF” at the end of the page. Anew window will open with the report on a format that you can save as PDF or print.
