# Supplementary material for: Optimized conditions for Listeria, Salmonella and Escherichia whole genome sequencing using the Illumina iSeq100 platform with point-and-click bioinformatic analysis
Source: PLoS One. 2022 Nov 30;17(11):e0277659. doi: 10.1371/journal.pone.0277659 (PMC9710801; doi:10.1371/journal.pone.0277659)
Supplement: S2 File — (DOCX) [file pone.0277659.s002.docx]

*S2-Geneious*

*Step by step protocol for NGS raw data processing and bacteria species identification in 9 easy steps!*

*Sonsiray Alvarez Narvaez*

*University of Georgia |Georgia, Athens, US*

*December 2021*

**READ IMPORT**

1. Open Geneious and in the Local folder create a new folder by clicking in the “Add “ button and selecting “New Folder”. A new windows will open for you to name the new folder you will create.


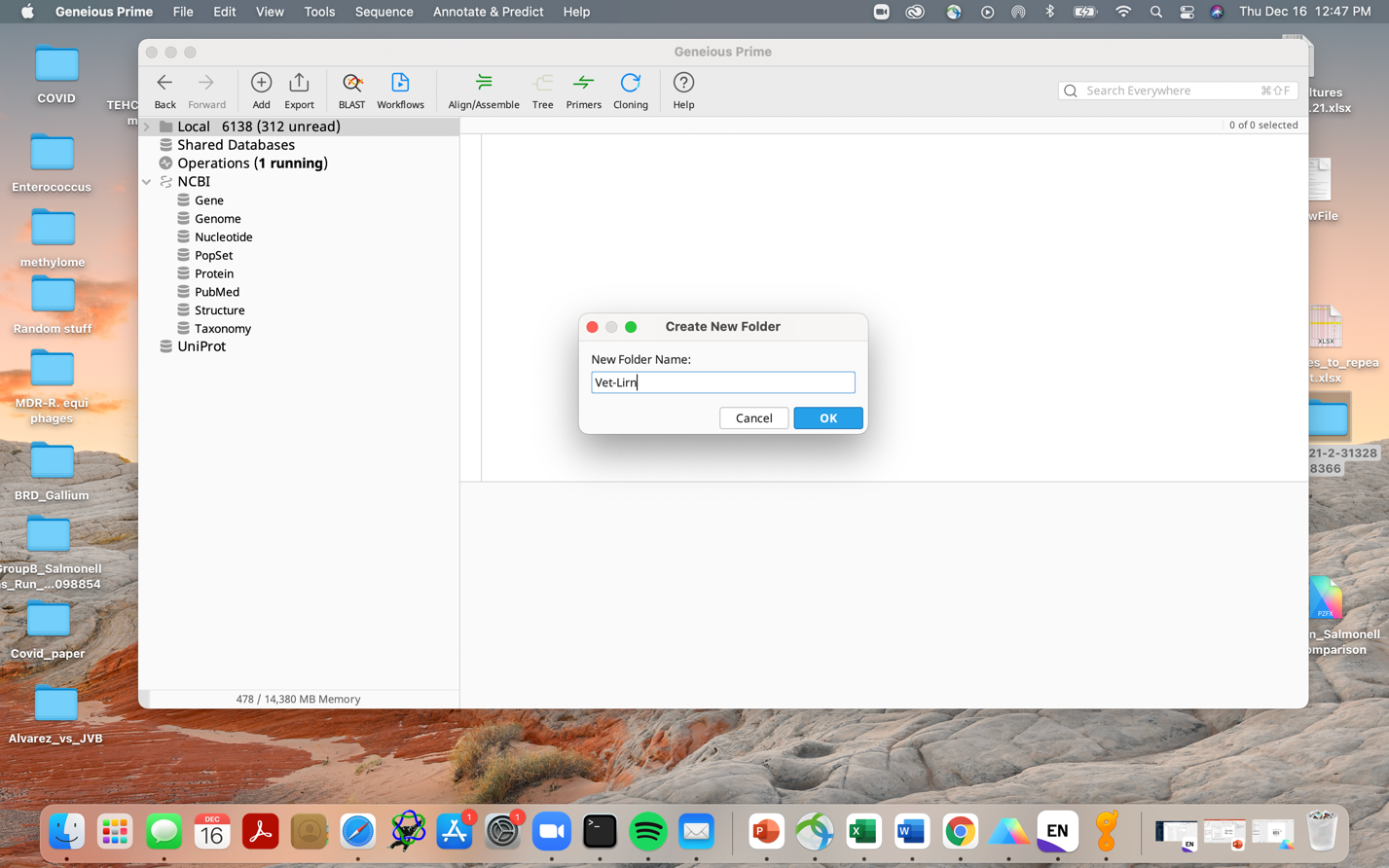

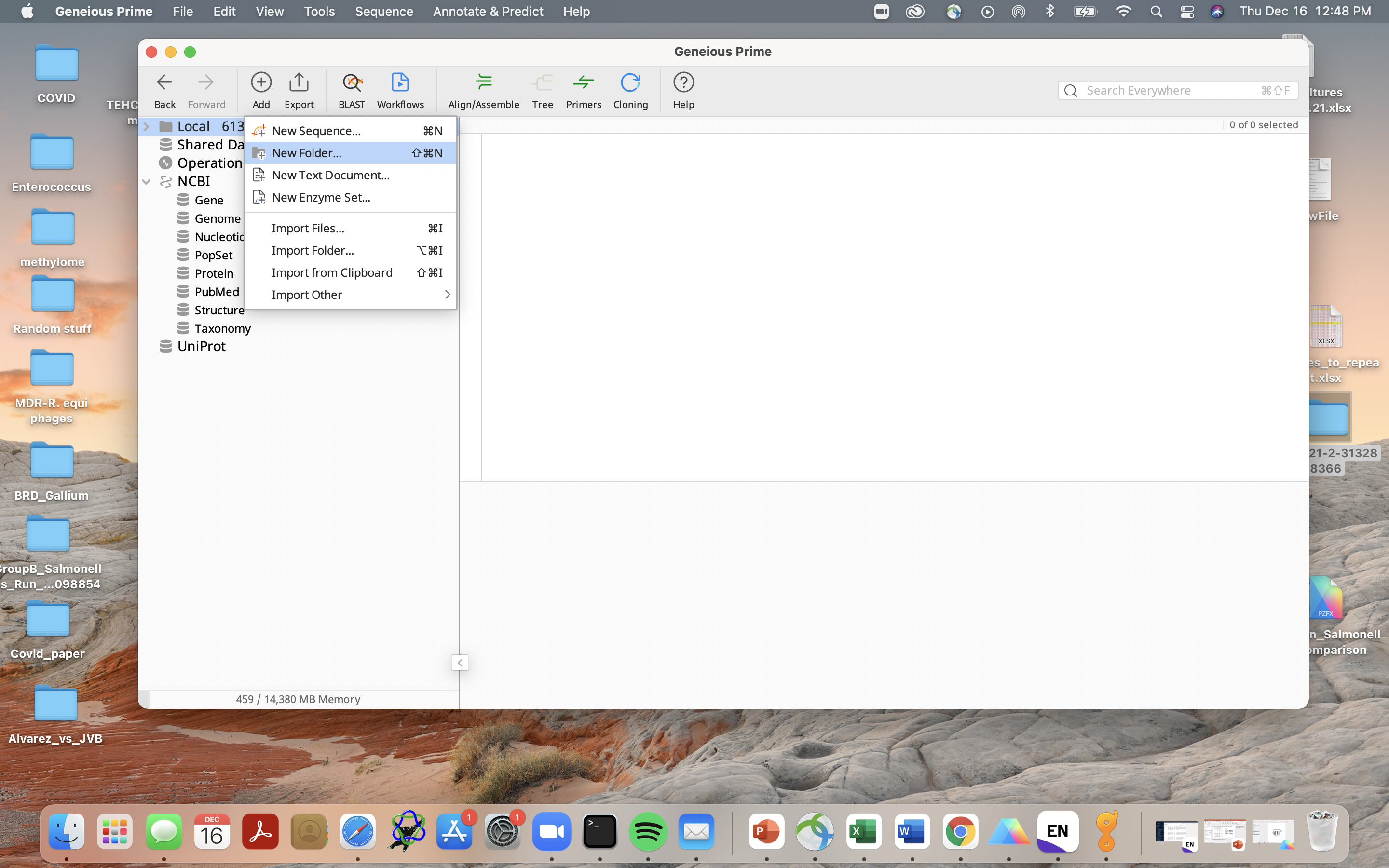


To look at the content of your folder just click on the name. You will know which folder is selected as its name will be highlighted in blue. You can see that your folder is empty (no files). You can import the raw reads generated by the sequencing machine (fastq files) by dragging them from your local computer into the empty folder. A new windows will open asking you about the “Read Technology”. Please select Illumina and “Don’t pair reads”. Then click ok.


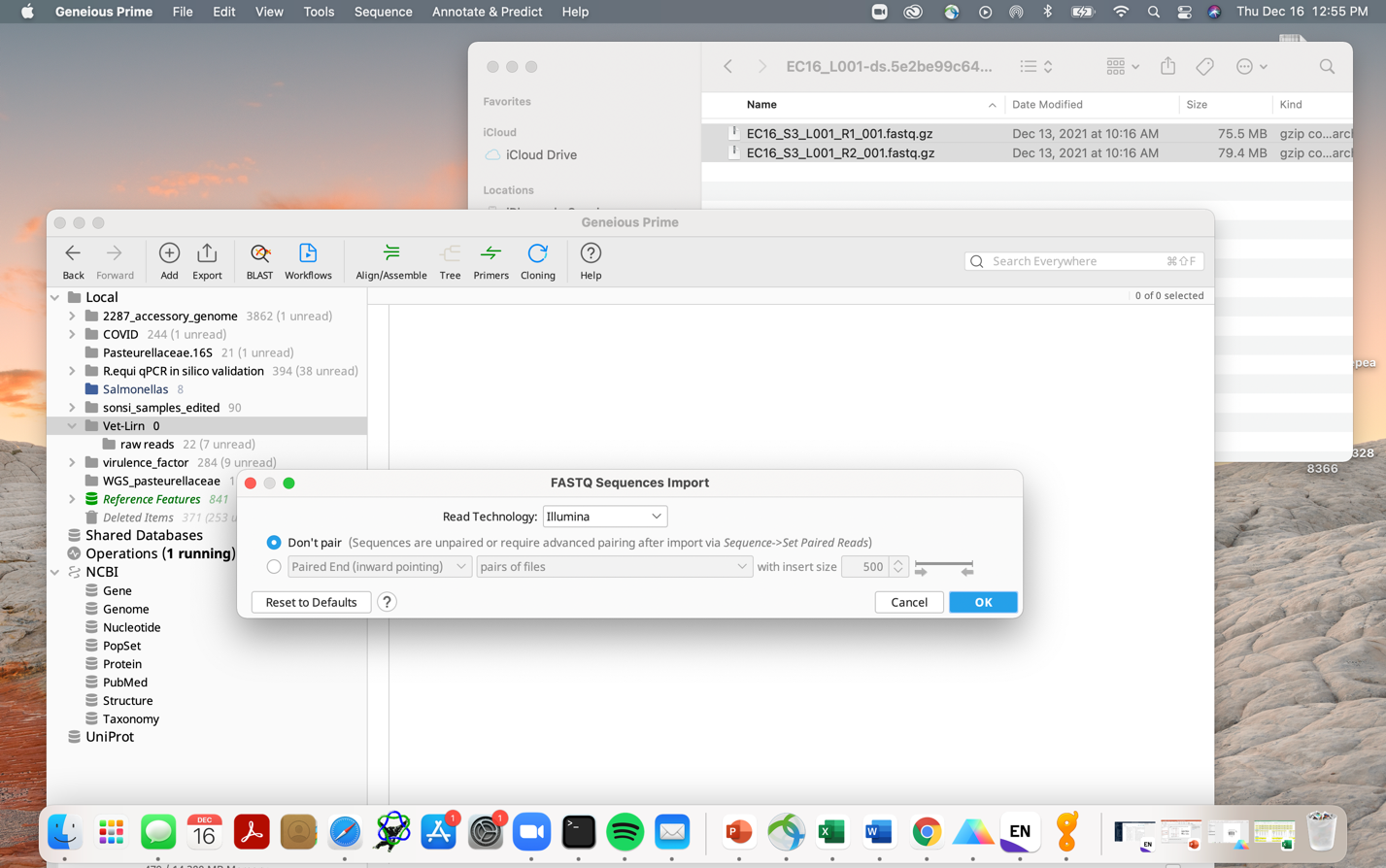

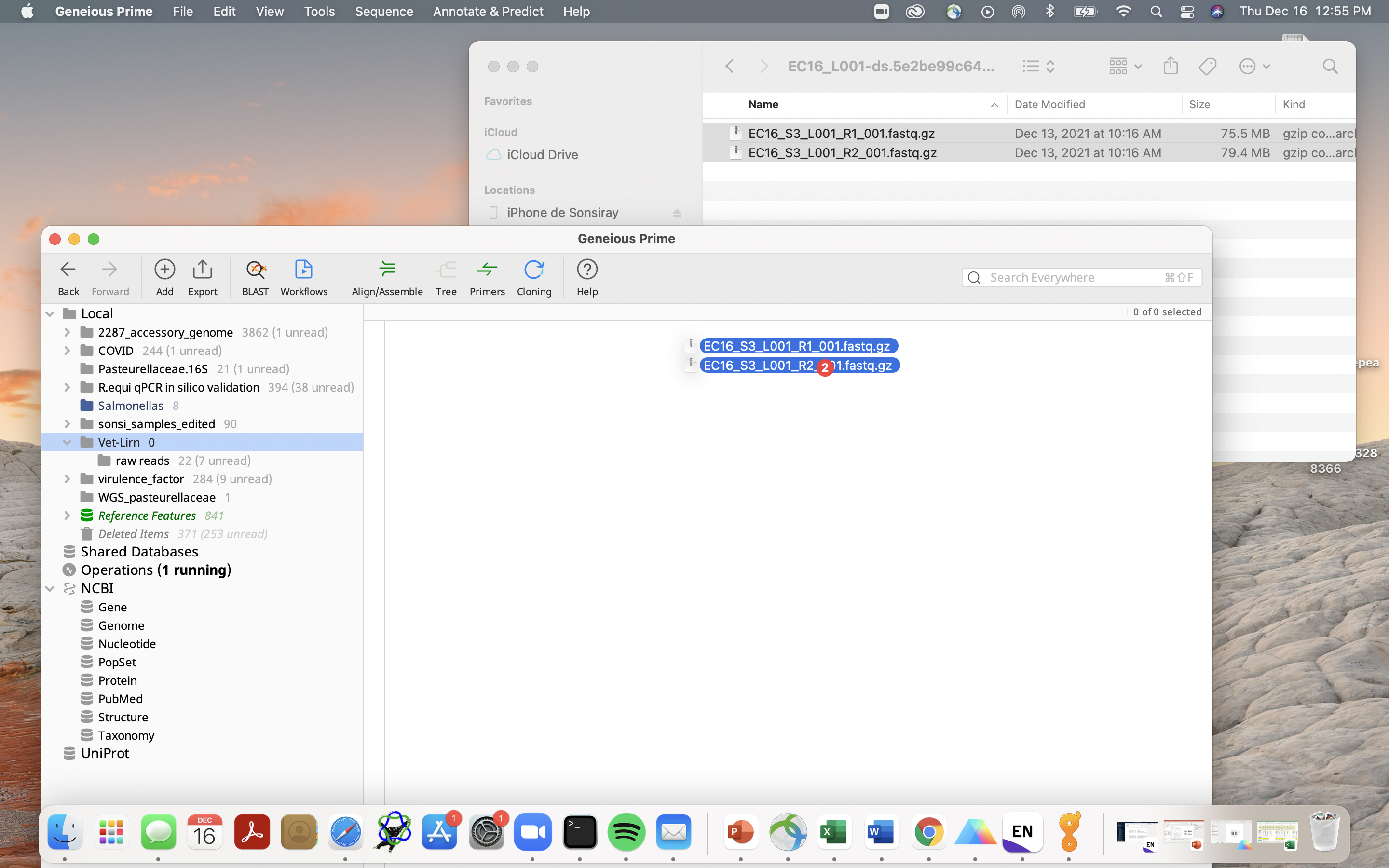


**RAW READS TRIMMING AND FILERING**

2. We will trim and filter Illumina iSeq raw reads using BBDuk read trimmer. BBDuk is a Plugin program, and it does not come pre-installed in Geneious. Make sure you install BBDuk before you start your analysis. To install BBDuk go to “Tools” and then “Plugins”.


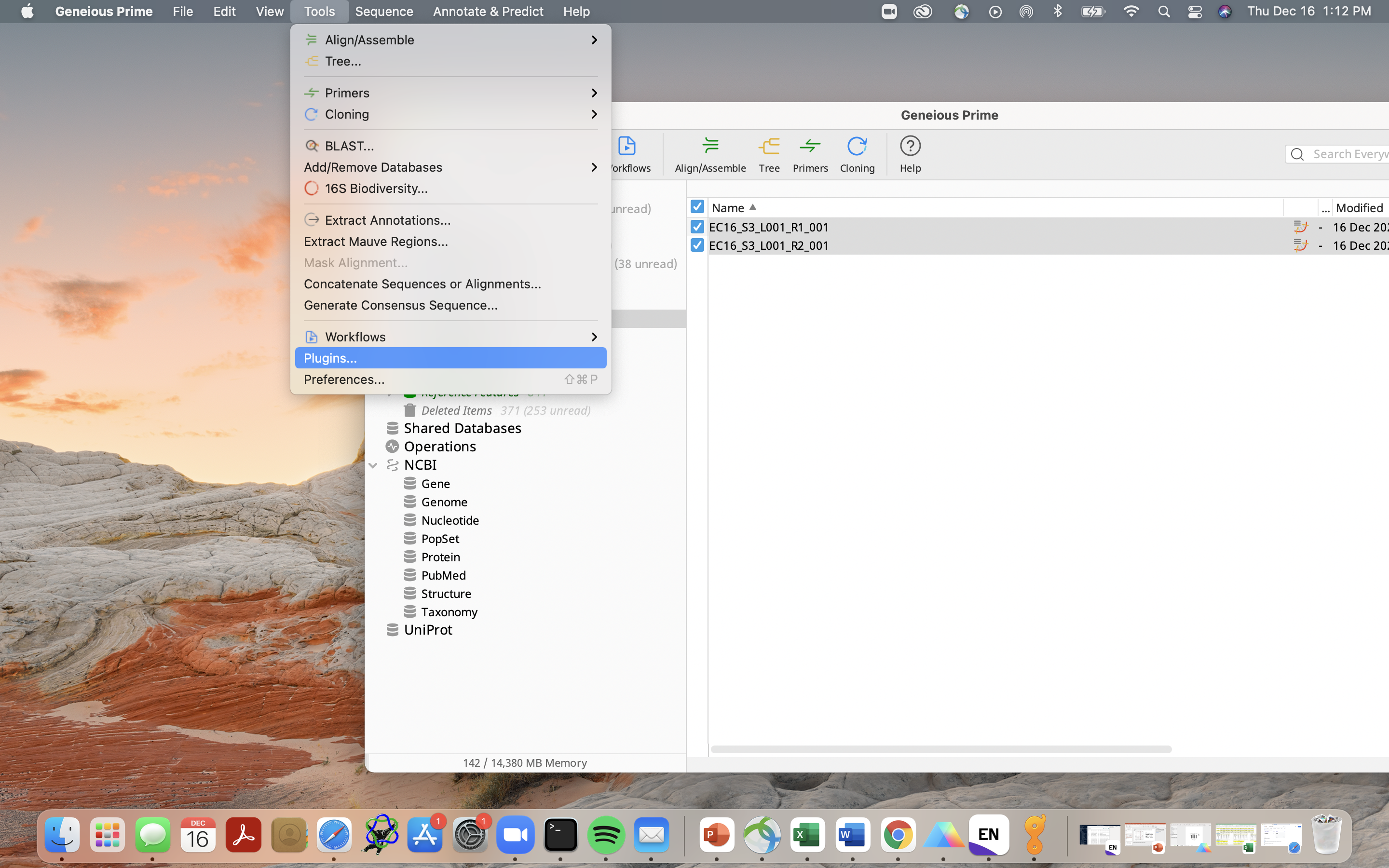


Look for BBDuk in the list of “Available Plugins”. Then click install and then “OK” both in the “Install Results” window and in the “Preferences” window.


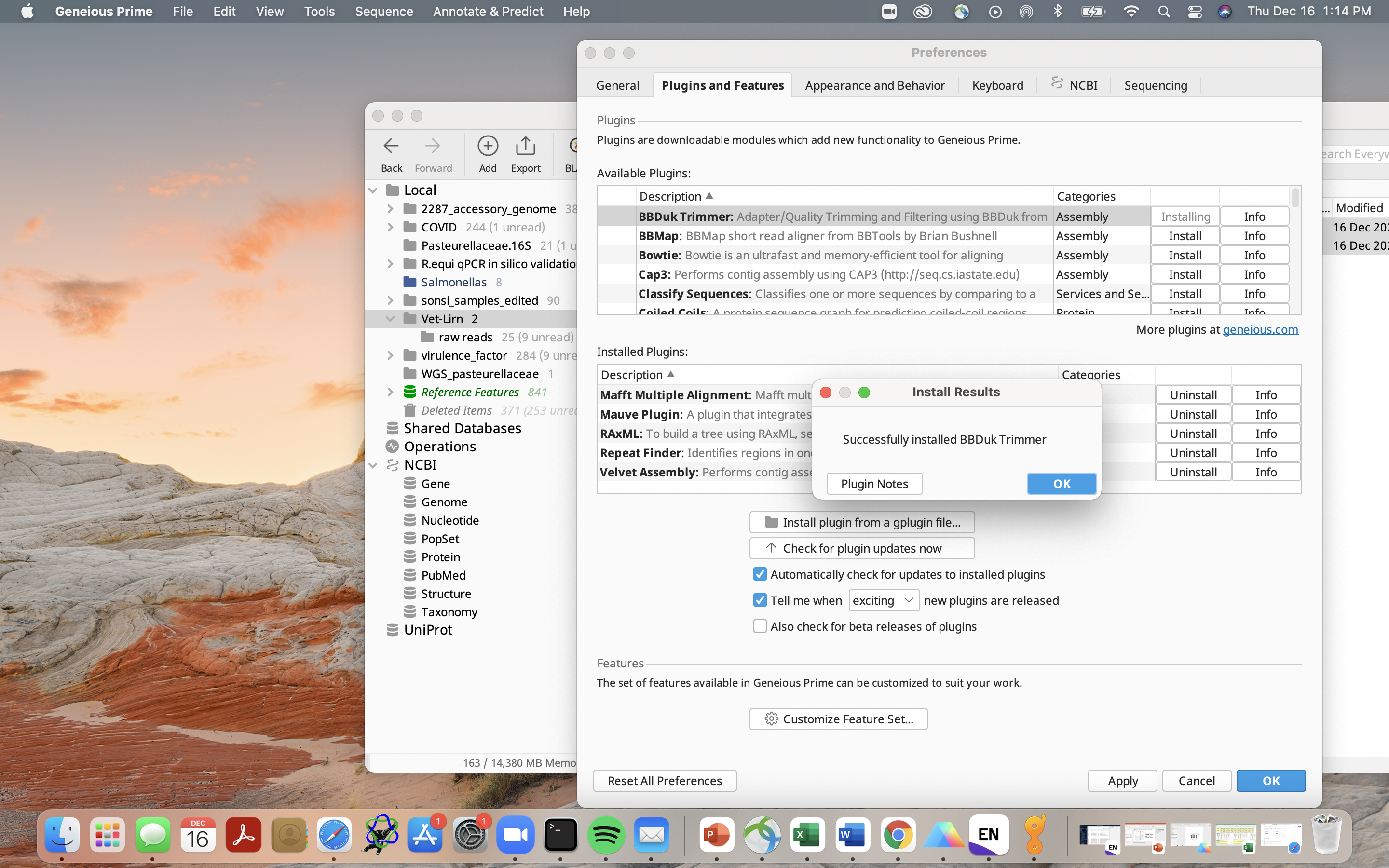


3. Now that BBDuk is installed and your reads are imported, go to the Vet-Lirn folder and select your read files by click on the square box next to their name. You will know that the sequences are selected as a blue tick sign will appear next to their name. Then click on “Annotate and Predict” and select “Trim using BBDuK”.


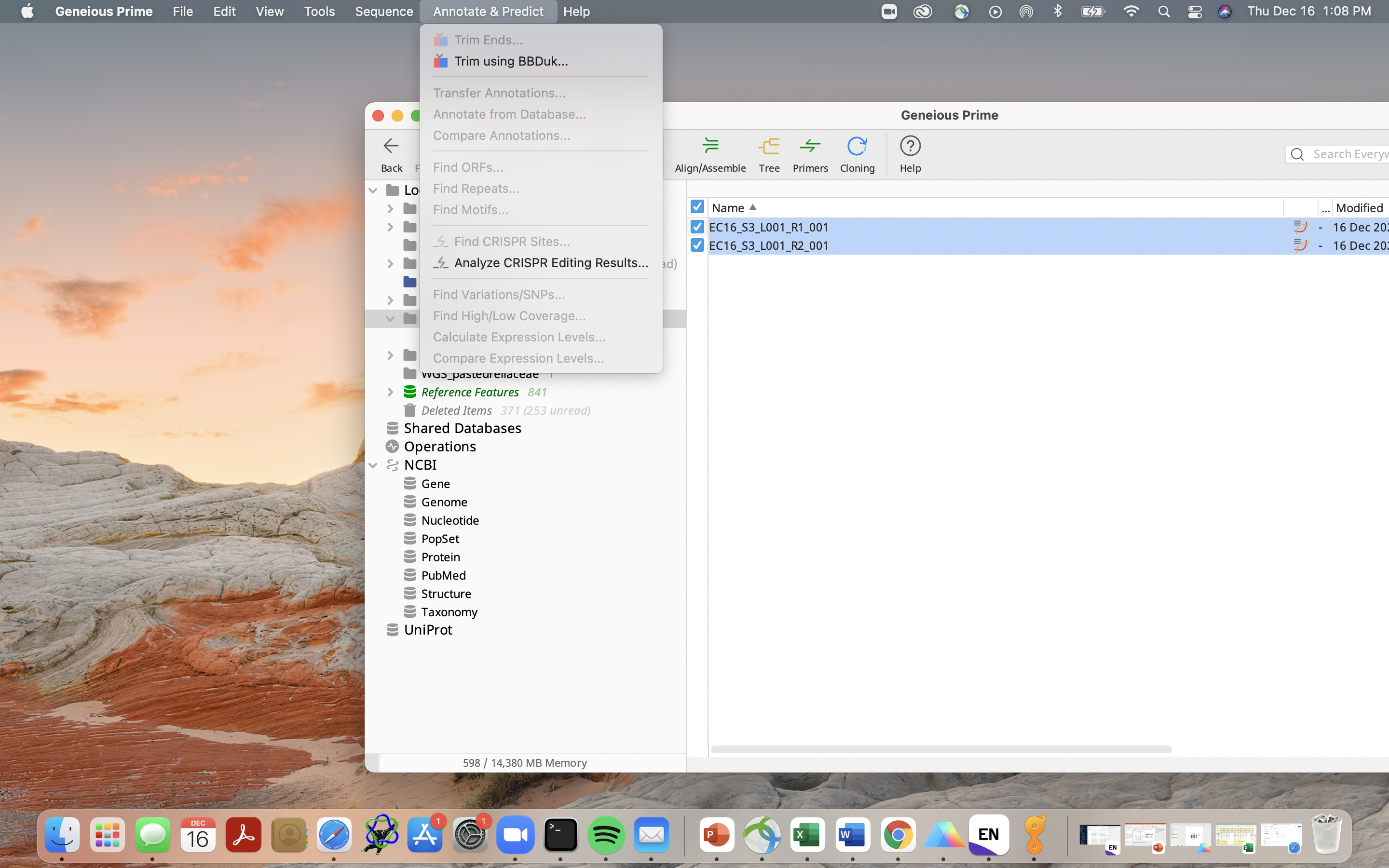


A new window called “Trim using BBDuk” will open. Select “Trim Adapters”🡪 All TruSeq, Nextera and PhiX, “Trim Low Quality”🡪 Both Ends Minimum Quality 6 and “Discard Short Reads” 🡪 Minimum length 10 bp.


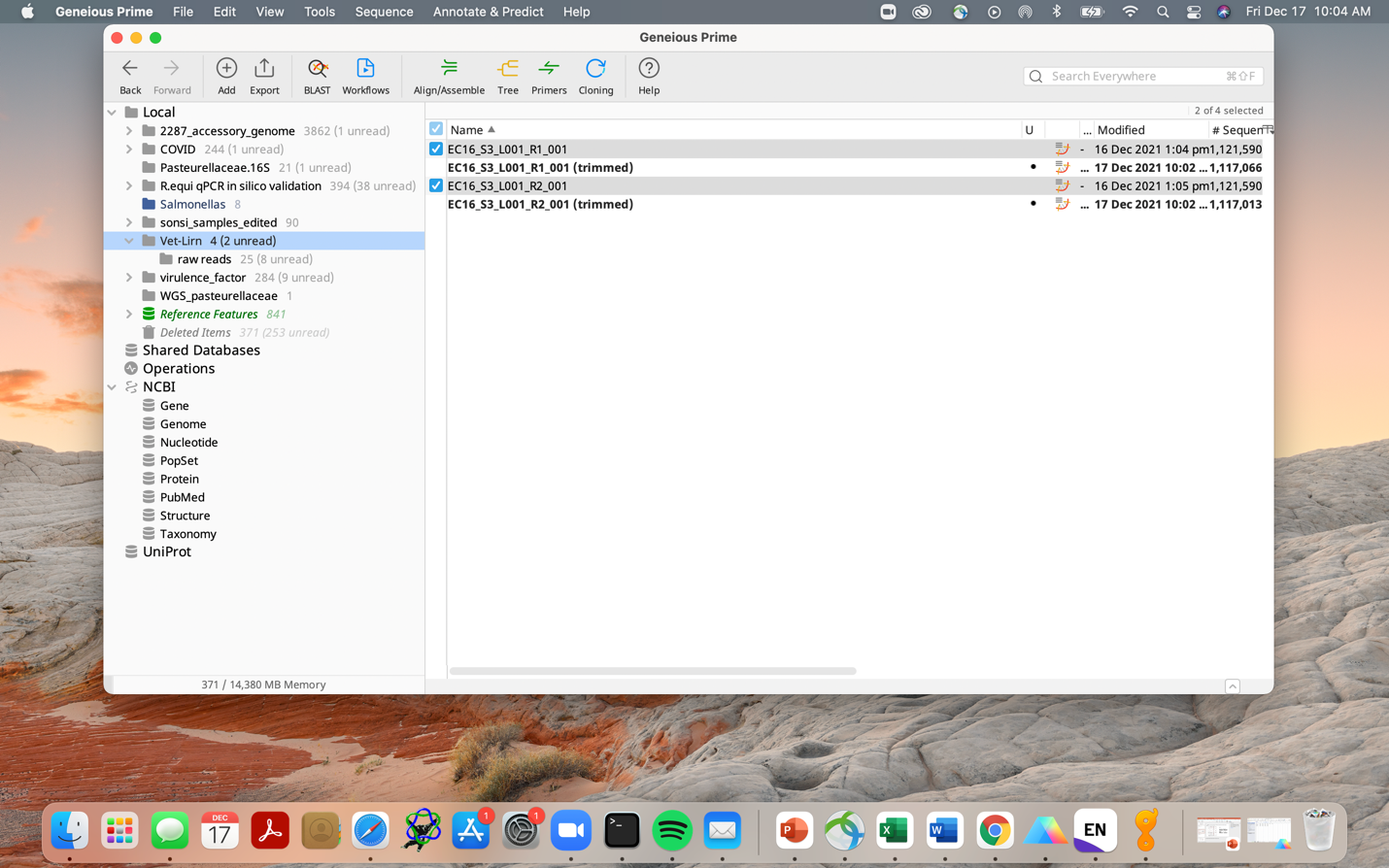

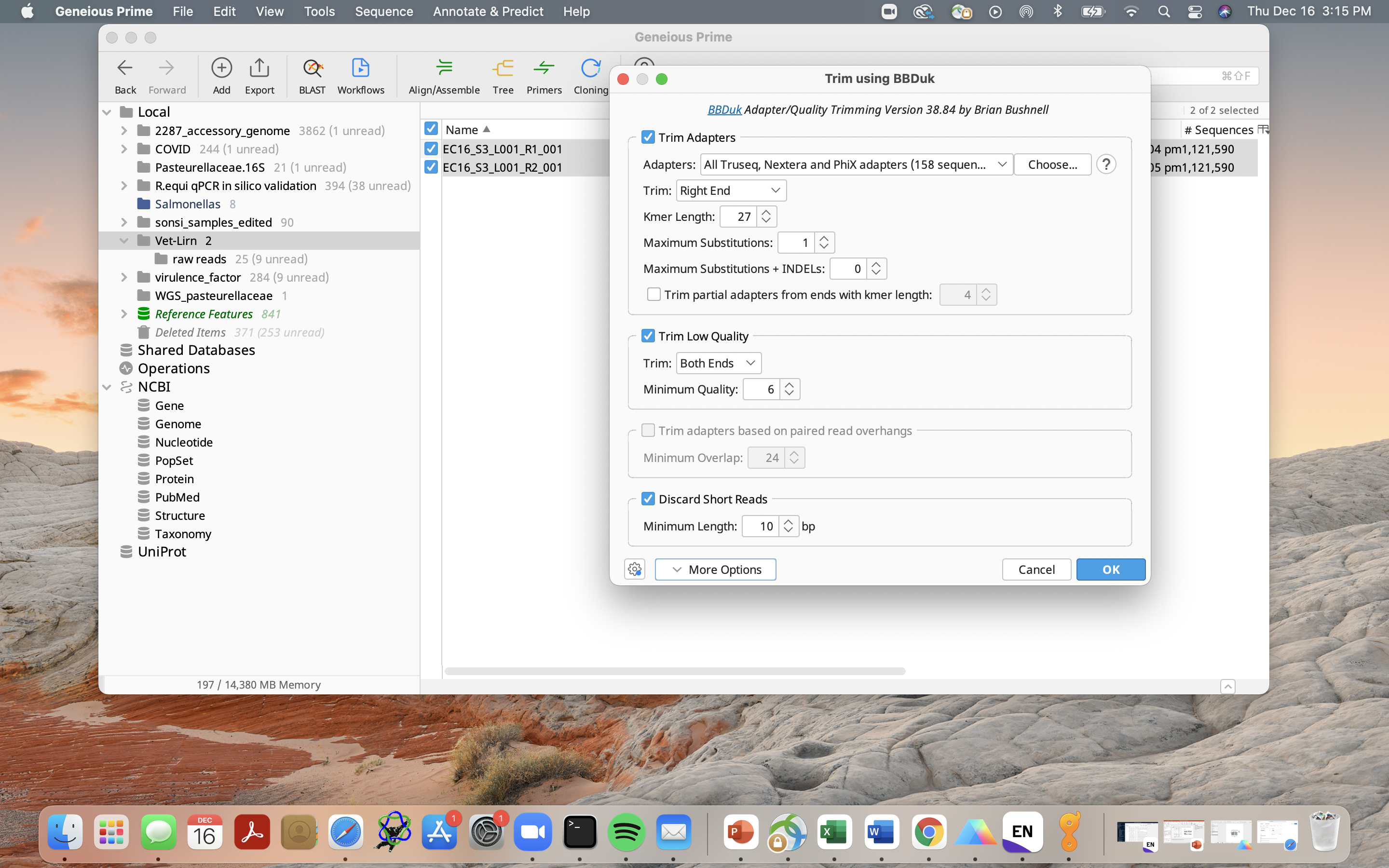


**CONTIG ASSEMBLY**

4. Select your trimmed read files by click on the square box next to their name. You will know that the sequences are selected as a blue tick sign will appear next to their name. Then click on “Tools”, “Align/Assemble”, and finally “De Novo Assemble”.


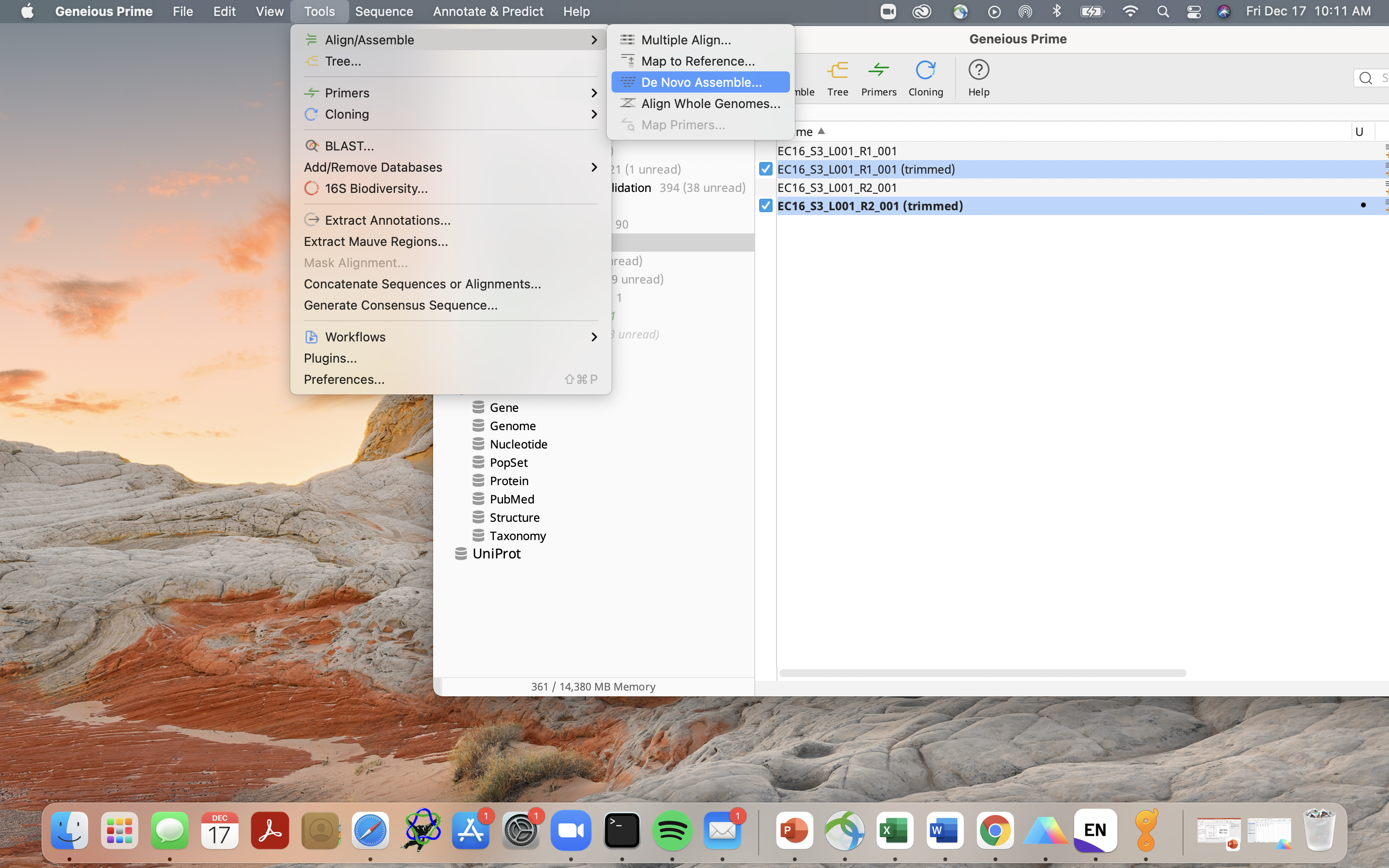


A new window will open “De Novo Assemble”. In “Method”, chose SPAdes as “Assembler”, Multi Cell as “Data Source”, Error correct & assemble as “Method” and select for “Careful Mode”. Reads are already trimmed, and quality filtered so in “Trim Before Assembly” choose Do not trim. In Results, you can change the Assembly Name and select for “Save assembly report”. Leave every other setting as default and click OK. This may take a while depending on the computer. Of note, if an input error appears you may need to install Ubuntu on your computers following the directions at <https://help.geneious.com/hc/en-us/articles/360045071771>. If your computer does not fit the requirements, another assembler program such as Velvet can be used.


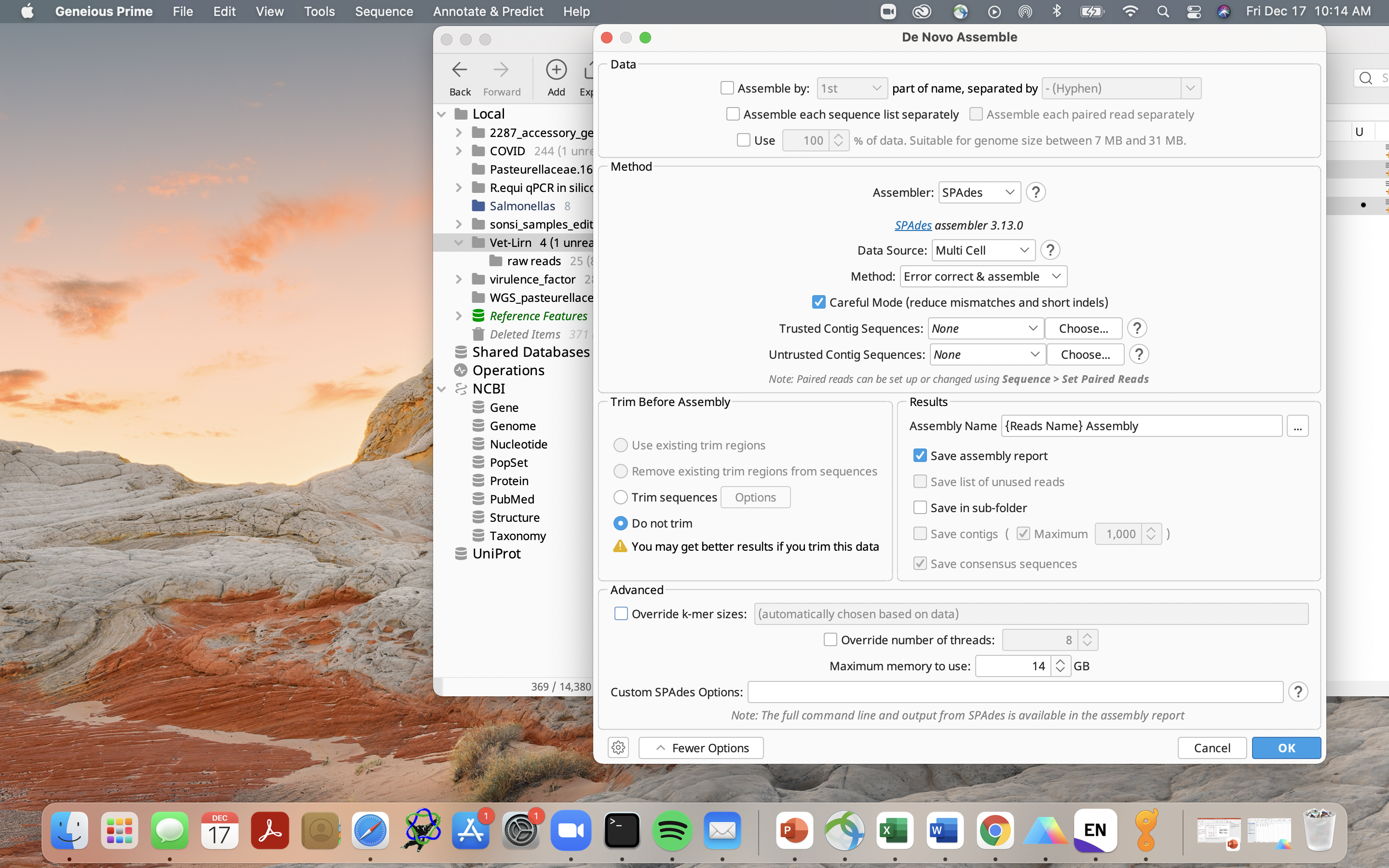


5. Three files will come out as a result of SPAdes: two fasta files containing the contigs (“_Assembly Contigs”) and scaffolds (“_Assembly Scaffolds”) and one file containing the assembly report. The fasta files contain the genetic sequences of your bacteria and can be used for bacteria species identification. To download them, select the files by click on the square box next to their name. The. Go to “File”, “Export” and click on “Documents”. A new windows will open for you to select the file format. Select “FASTA sequences/alignment (*.fasta) and click ok.


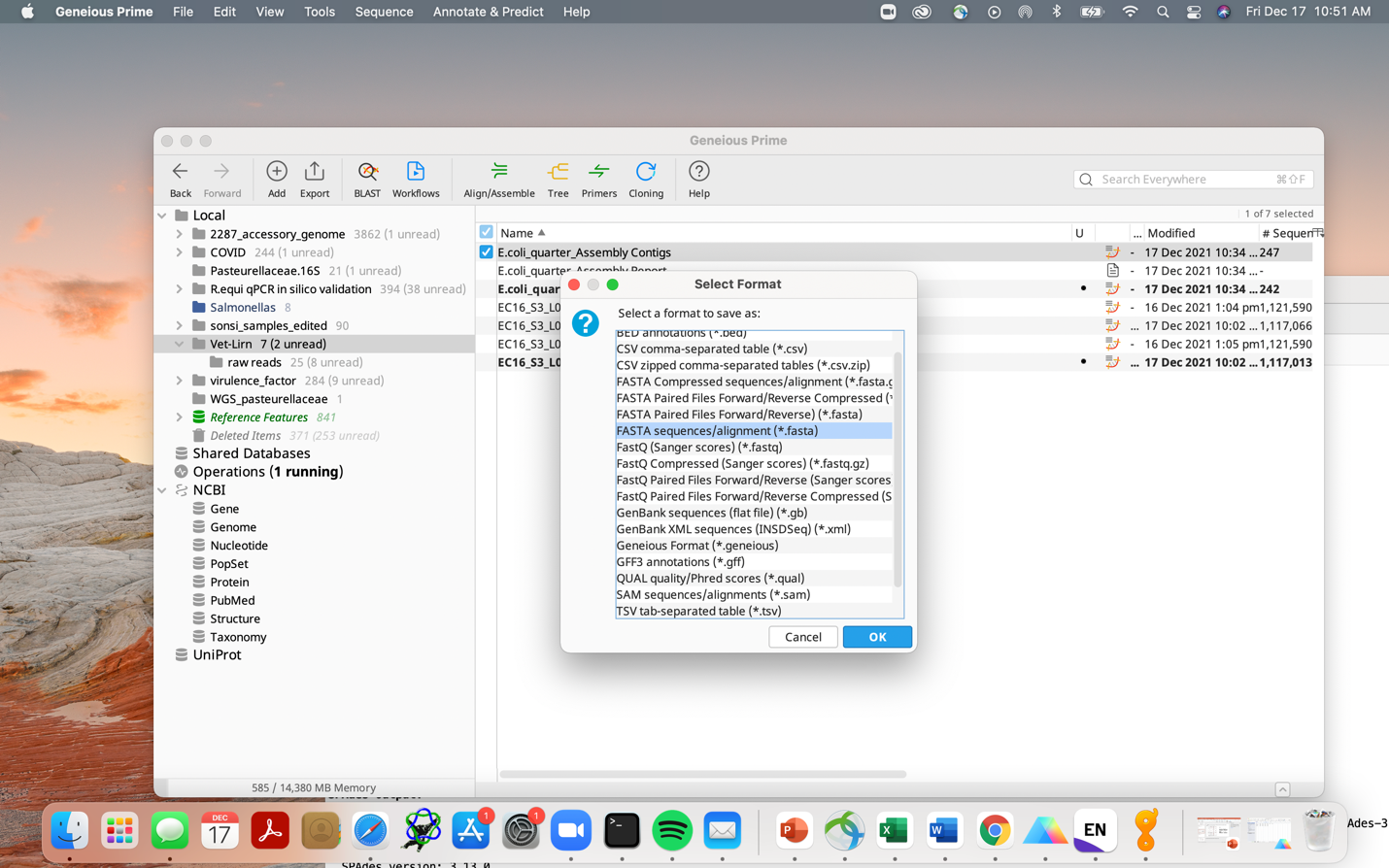

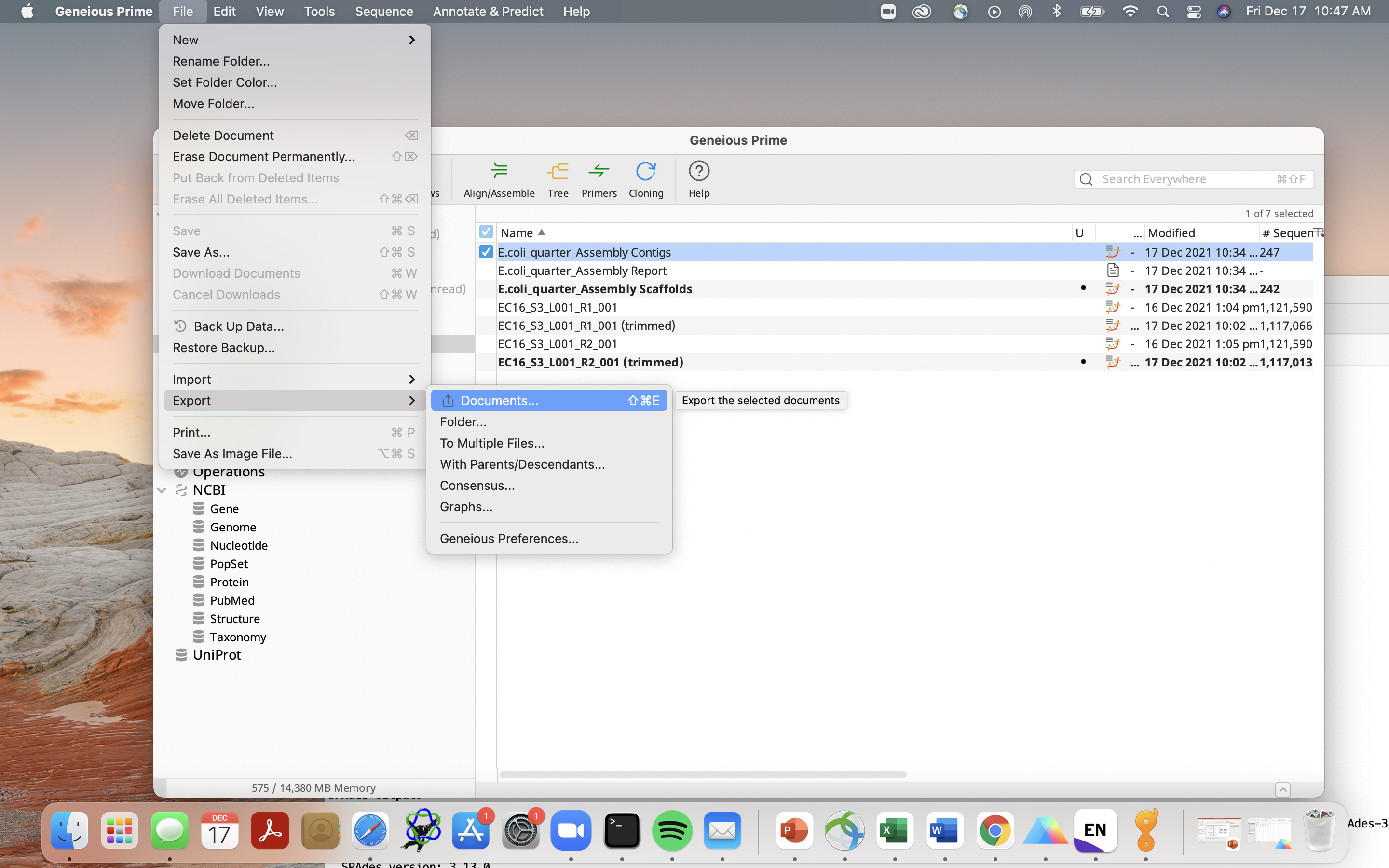


6. To visualize the assembly report, click on “Assembly report” and a new window will open.


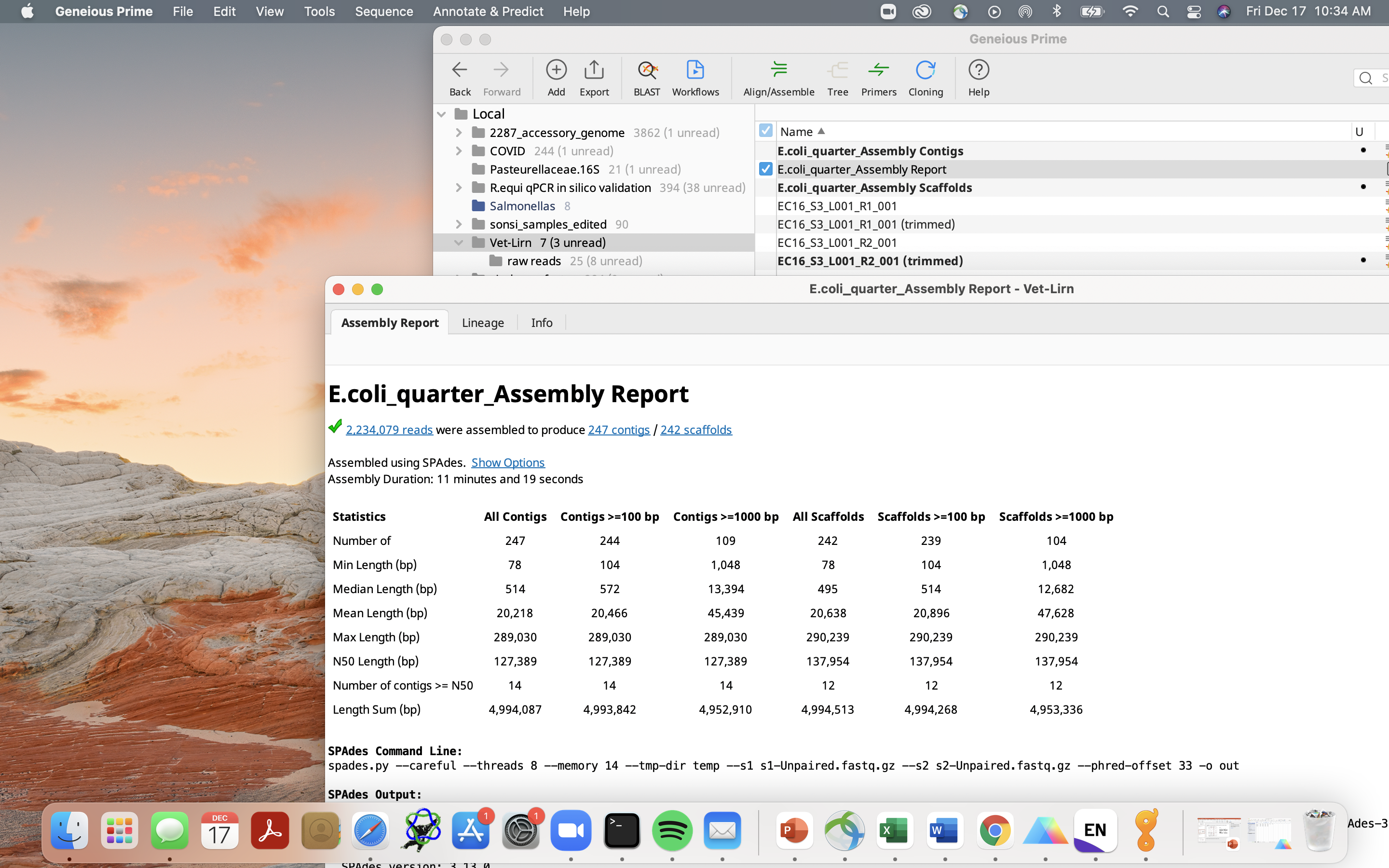


The main Assembly Statistics appear on a table in which columns describe:

-“All Contigs” is the total number of contigs in the assembly. This is the number of contigs that you will find in your fasta file

-“Contigs >=100bp” is the total number of contigs with more than 100 bps length in the assembly.

-“Contigs >=1000bp” is the total number of contigs with more than 1000 bps length in the assembly.

This same applied to the scaffolds, that are bigger contigs. You can use either or the files (contigs.fasta and scaffold.fasta) for species and ARGs identification.

Rows describe:

-“Number of” contigs/scaffolds in the assembly

-“Min Length (bp)”: Minimum length in base pairs of contigs/scaffolds in the assembly

-“Median Length (bp)”: Median length in base pairs of contigs/scaffolds in the assembly

-“Mean Length (bp)”: Mean length in base pairs of contigs/scaffolds in the assembly

-“Max Length (bp)”: Maximum length in base pairs of contigs/scaffolds in the assembly

-“N50 Length (bp)”: The size of the shortest contig in the set of longest contigs that together cover at least 50% of the total Genome Size [the bigger the number, the better the quality of the assembly]

-“Number of contigs >= N50” : Also known as L50, or the number of contigs equal or longer in size that N50

-“Length Sum (bp)”: Total number of bases in the assembly

**BACTERIA SPECIES IDENTIFICATION**

Geneious does not have a dedicated program for bacteria species identification. For this reason, we will use Basic Local Alignment Search Tool or BLAST tool with one contig (or piece) of the genome you just sequenced and assembled.

7. Double click on your “Assembly Contigs” file. A new window will open containing your assembled genomic sequences. In “Sequence view”, click on the magnifier glass symbol to enlarge the sequence. Then select the first 1000bps of your sequence by dragging your mouse over it. You will see the fragment of the sequence that gets selected as it will turn blue.


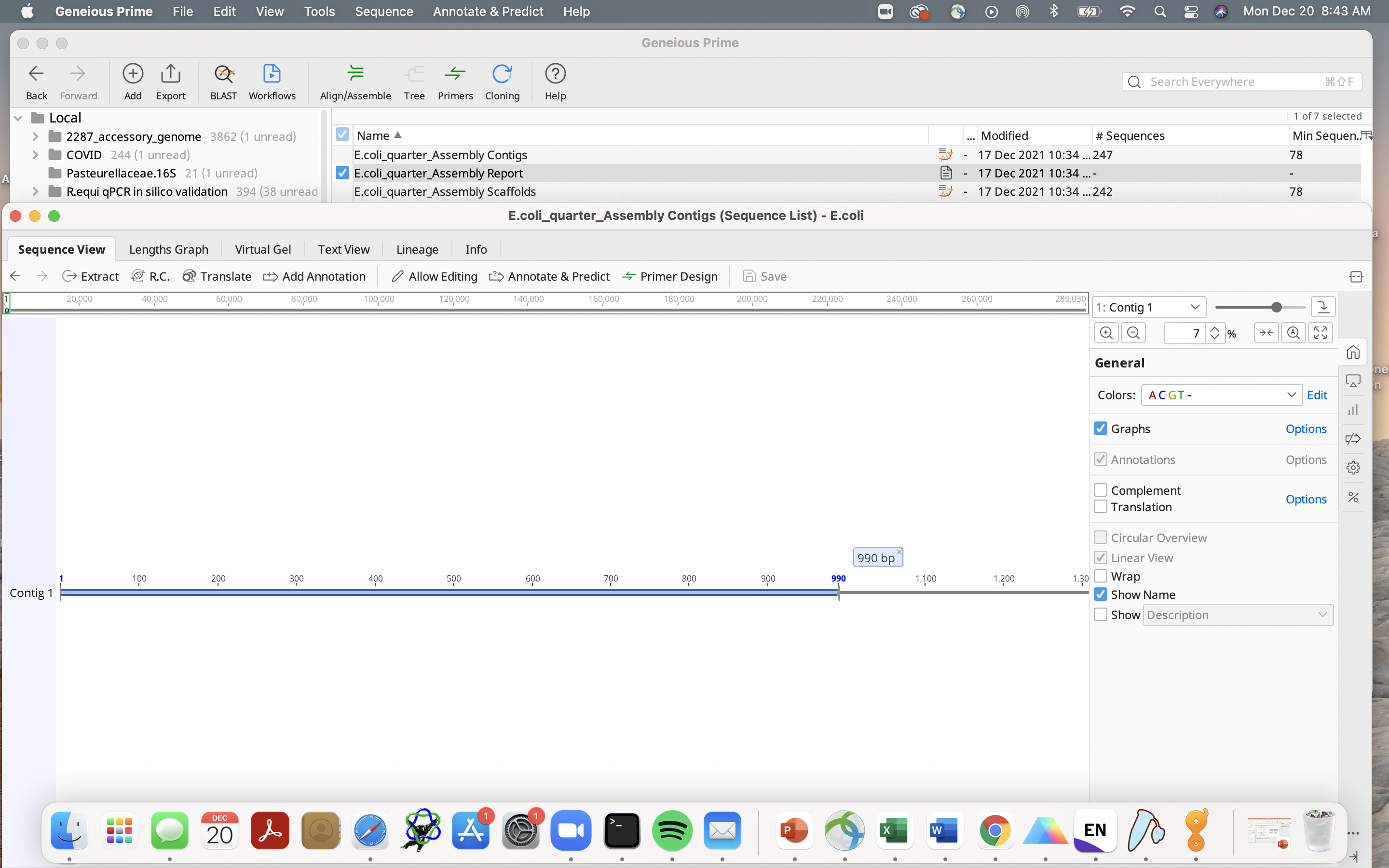


Then click on “Extract”. A new window will open (“Extract”) in which you can change the name of the genomic sequence you are about to extract. Then click ok.


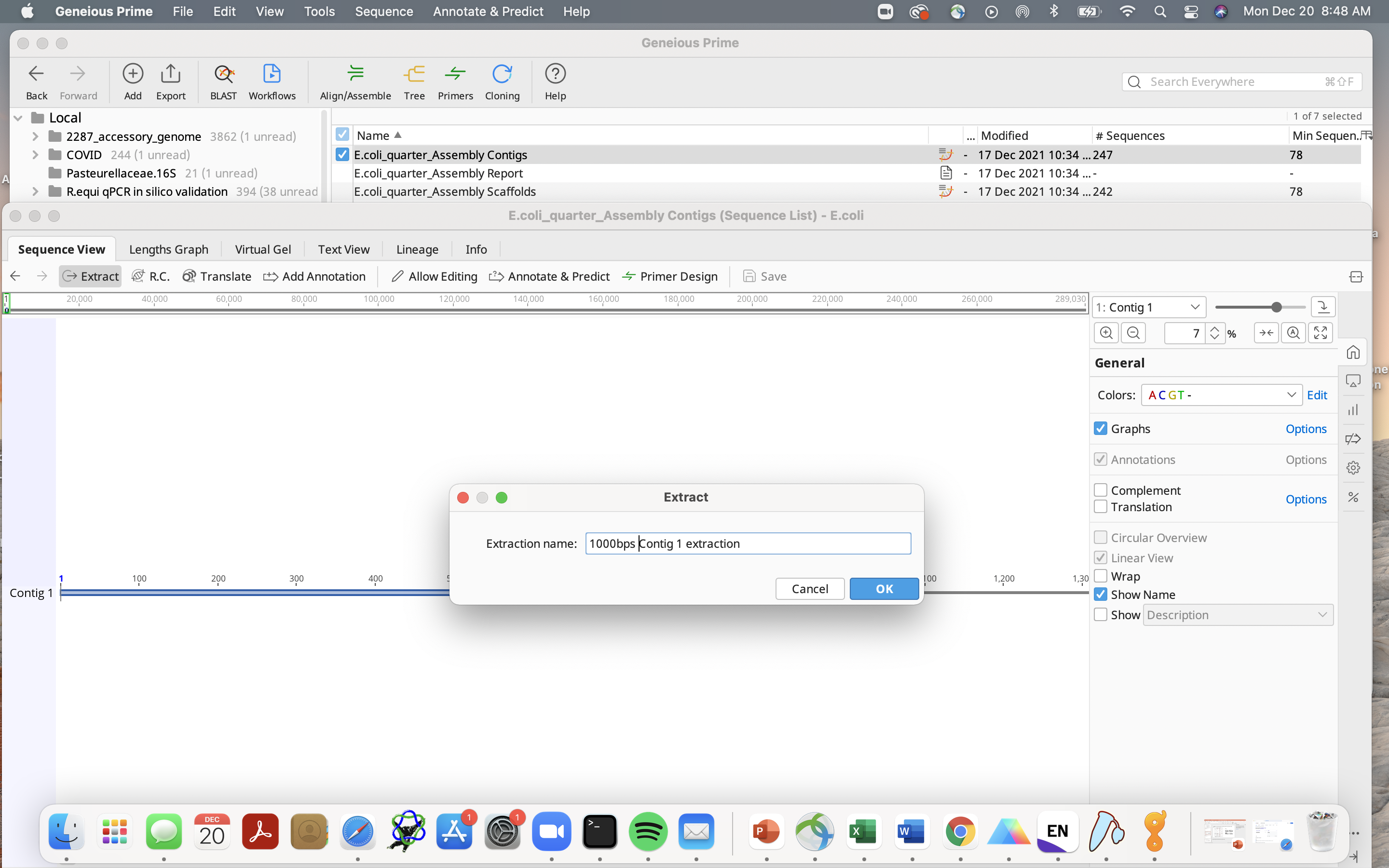


8. A new file will appear containing the sequence that you just extracted from the assembly (in this case 1000 bps of contig 1). Select that file and click in the BLAST tab. A new windows will open in which you select your sequence “contig1” as Query, “Nucleotide” as Database, “Megablast” as Program, “Hit table” as Results, Retrieve “Matching region” and a Maximum of 10 hits. Finally click Search. Alternatively, the highlighted 1000bp fragment can be copy and pasted into the NCBI BLASTn online tool.

<https://blast.ncbi.nlm.nih.gov/Blast.cgi?PROGRAM=blastn&BLAST_SPEC=GeoBlast&PAGE_TYPE=BlastSearch>


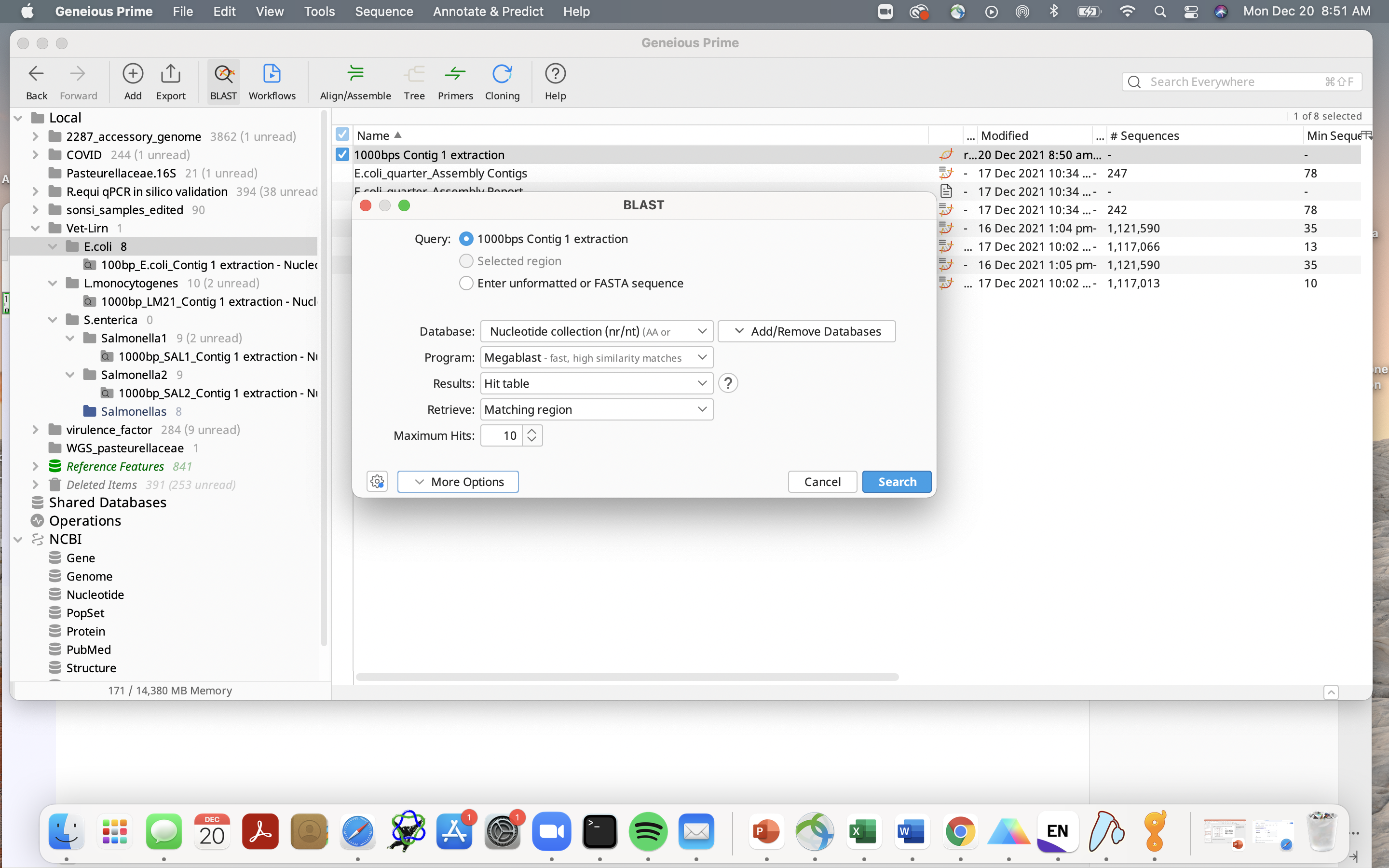


9. A new folder containing BLAST results will be created. Look at the “Hit Table”, it presents a lot of information.


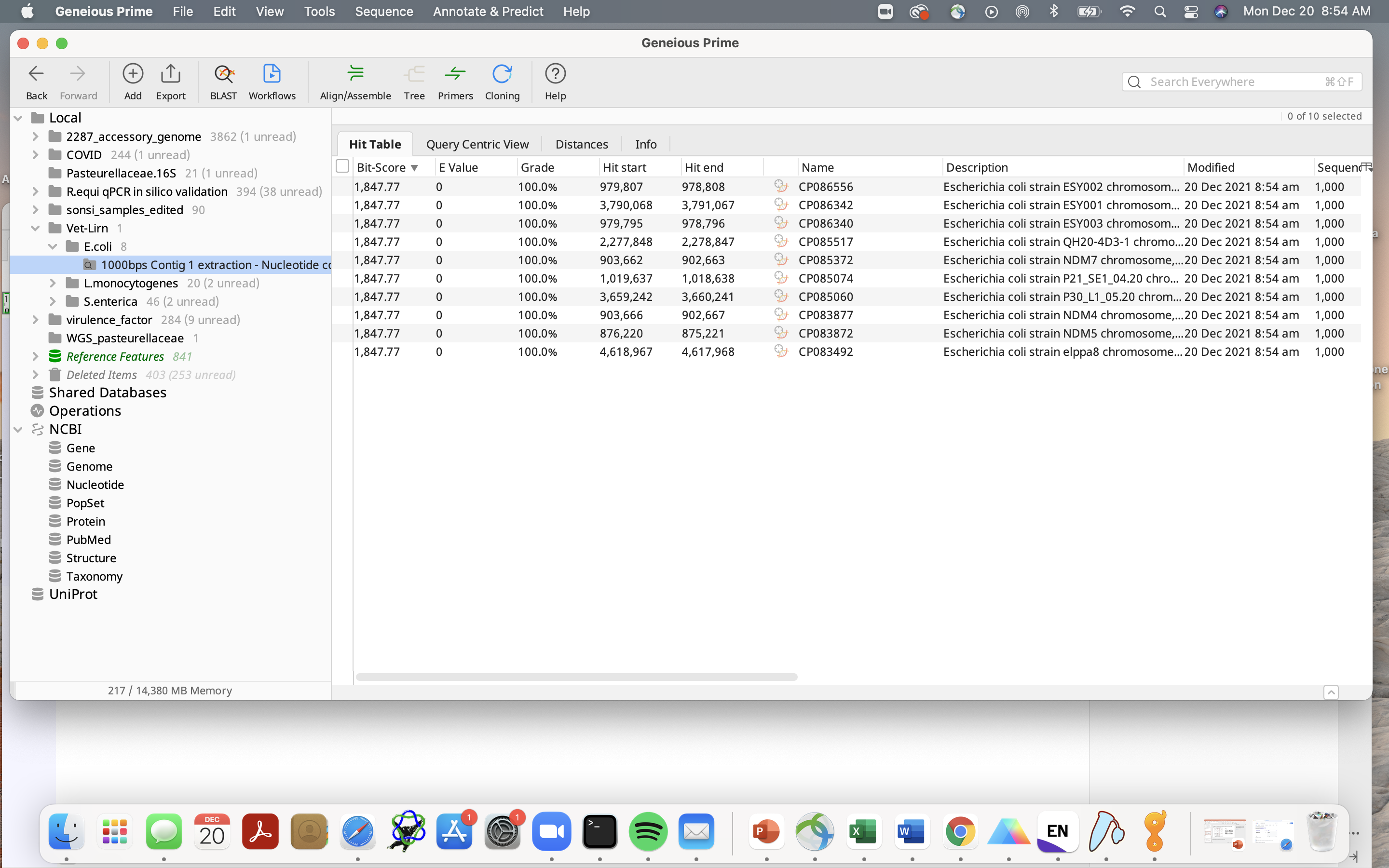


Of importance:

-Description: Identity of the sequence in the BLAST database that matched with your sequence

-Grade: shows the percentage of coverage, or how much of your sequence matched with the database sequence

-Name: NCBI accession number of the sequence in the BLAST database that matched with your sequence
