# Supplementary material for: Optimized conditions for Listeria, Salmonella and Escherichia whole genome sequencing using the Illumina iSeq100 platform with point-and-click bioinformatic analysis
Source: PLoS One. 2022 Nov 30;17(11):e0277659. doi: 10.1371/journal.pone.0277659 (PMC9710801; doi:10.1371/journal.pone.0277659)
Supplement: S3 File — (DOCX) [file pone.0277659.s003.docx]

*S3-GalaxyTrAkr*

*Step by step protocol for NGS raw data processing and bacteria species identification in 9 easy steps!*

*Sonsiray Alvarez Narvaez*

*University of Georgia |Georgia, Athens, US*

*December 2021*

**ACCESING GALAXYTRAKR**

1. Sign-in into your GalaxyTrkr account at <https://galaxytrakr.org>. Of note, this manual has been produced using macOS Big Sur v11.2 and GalaxyTrakr version of December 2021.
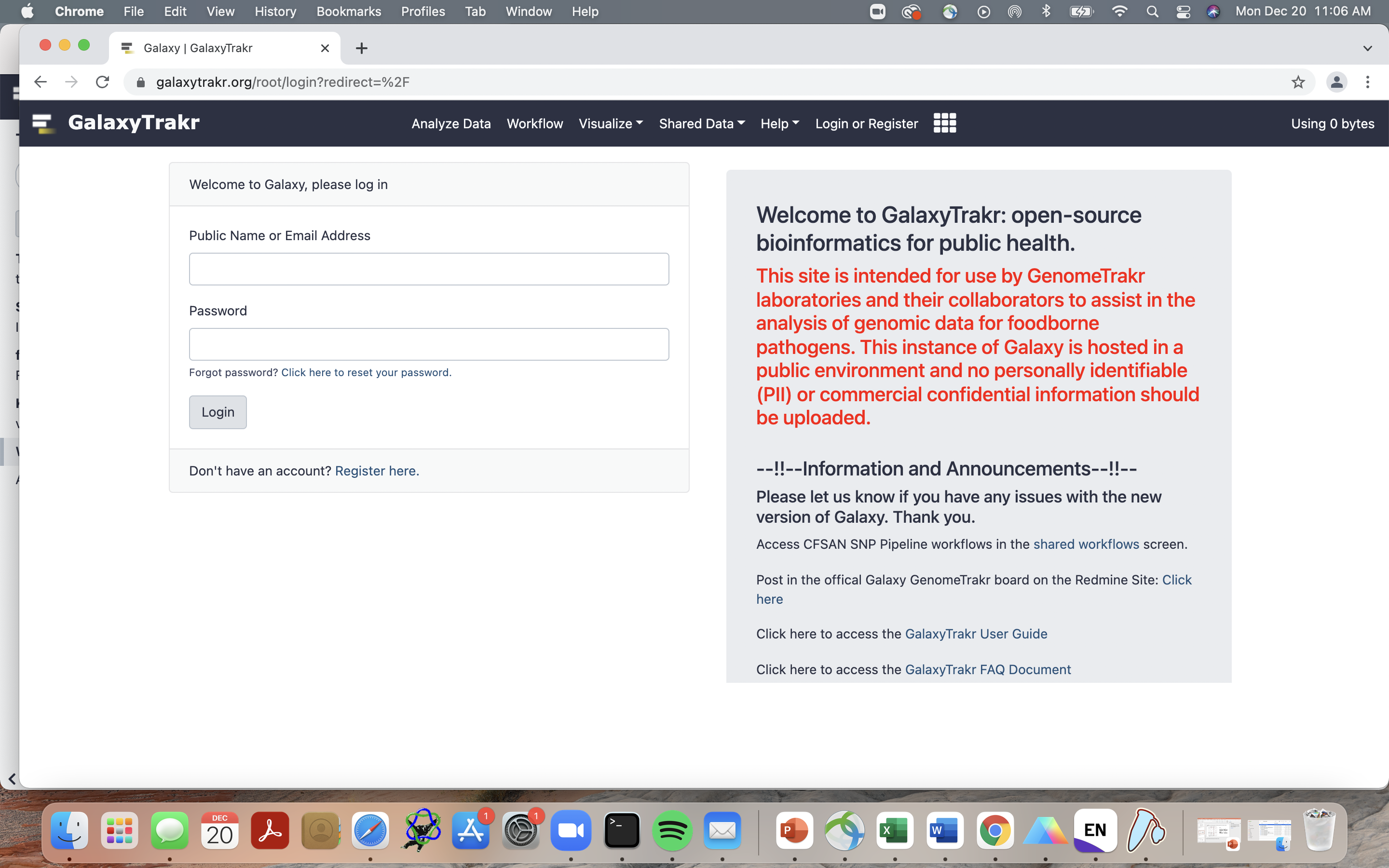


2. Once you enter the platform, create a new History (a new folder)by clicking on the plus sign next to “History”. Then, click in “Unnamed history” and rename your new folder.


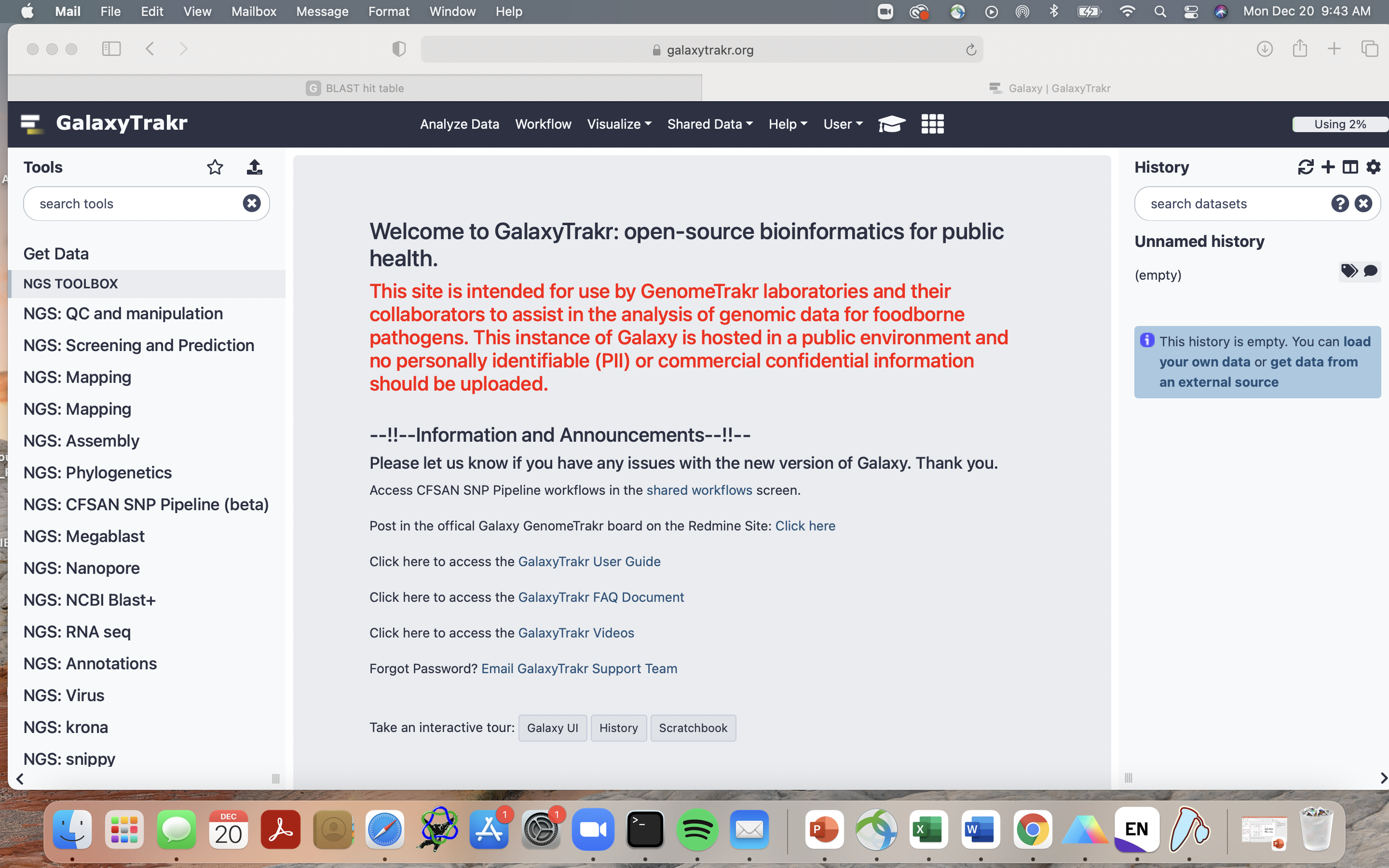


**READ IMPORT**

3. To upload your raw reads click on the upload/download button (arrow-up button next to the star in tools). That will open a new windows “ Download from web or upload from disk”. Drag the fastq files containing your raw reads from your local computer into the windows and click start.


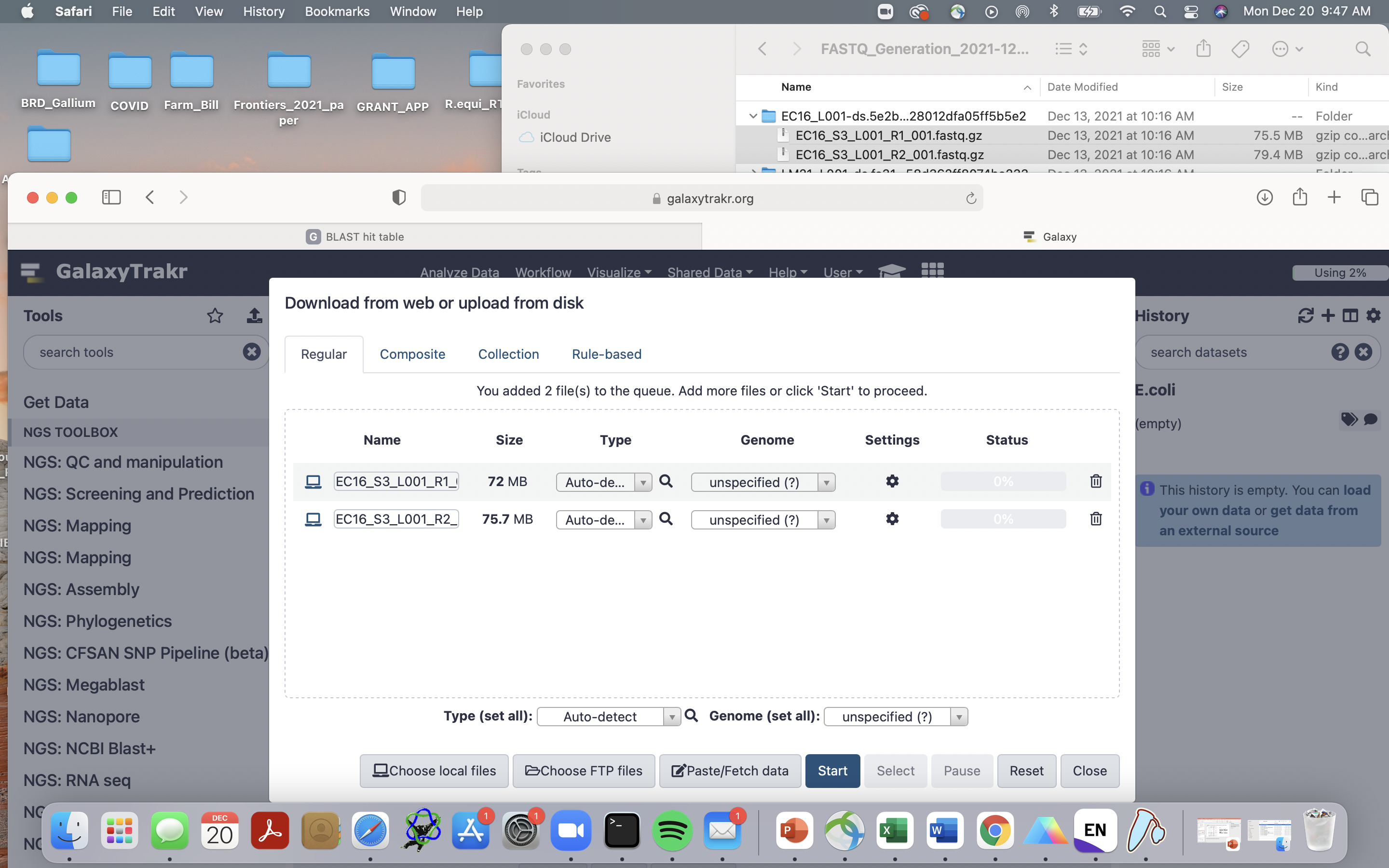


You will see that your documents have been uploaded into GalaxyTrakr when they turn green in the uploading windows. Also, you will see they will appear inside your History (right side of the screen).


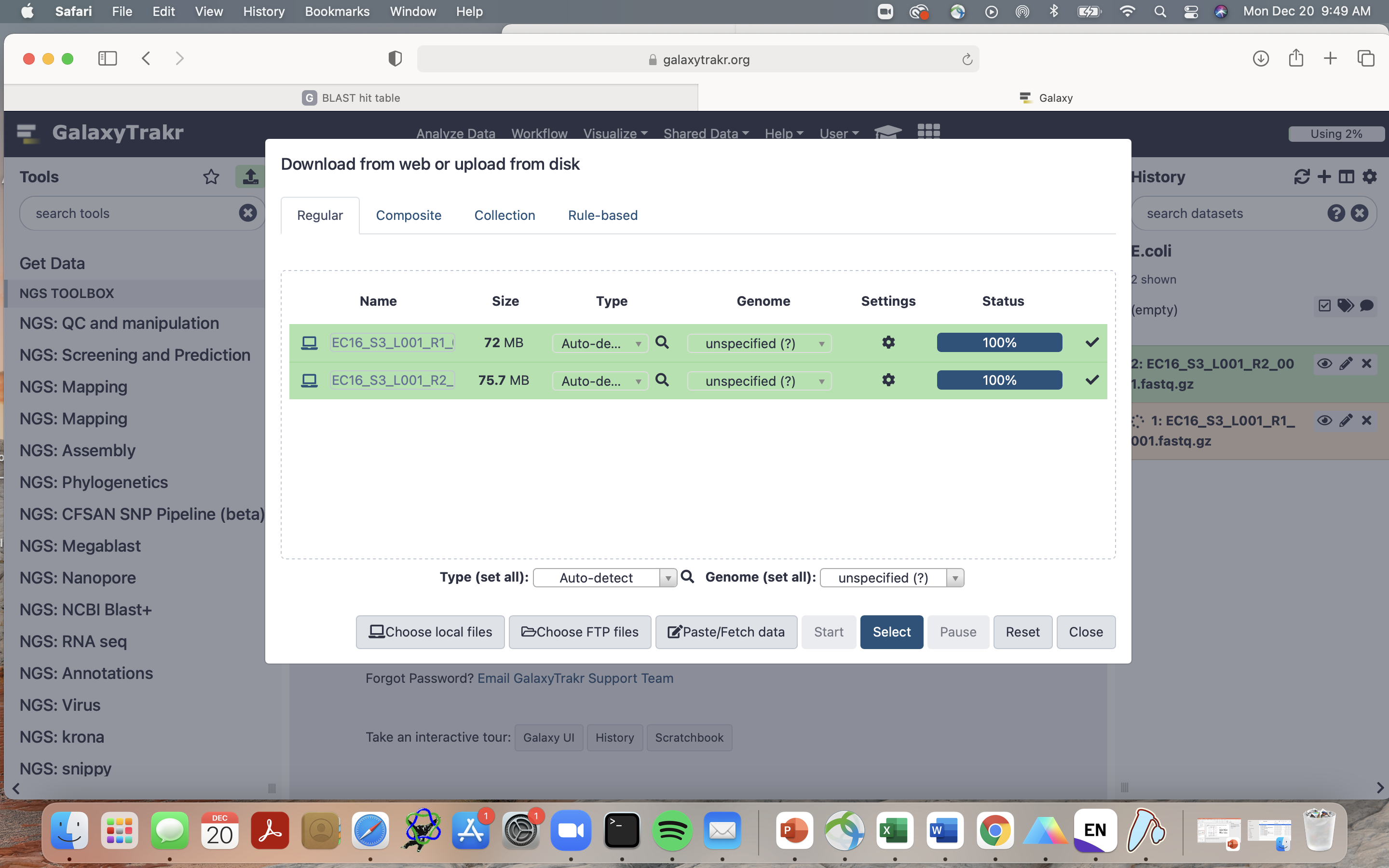


**RAW READS QUALITY CHECK**

4. We will use FastQC to look at the quality of the raw reads before starting the analysis. Under “Tools” write “fastQC” in the search tools space. A list of available programs will appear right under. Click on “FastQC Read Quality reports”. Under “Raw read data from your current history”, select the raw reads you want to analyze. Leave all settings as default and click “Execute”.


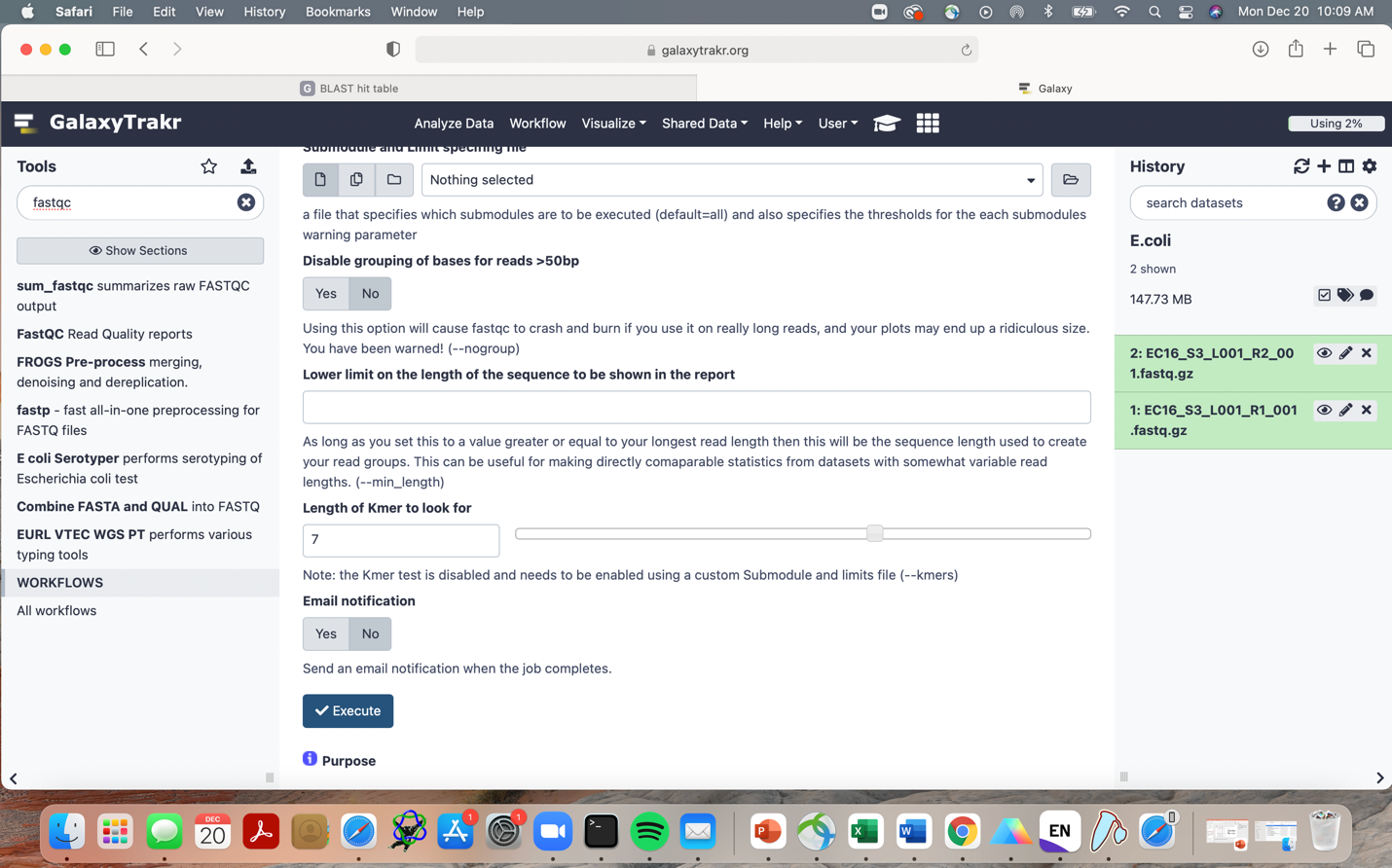

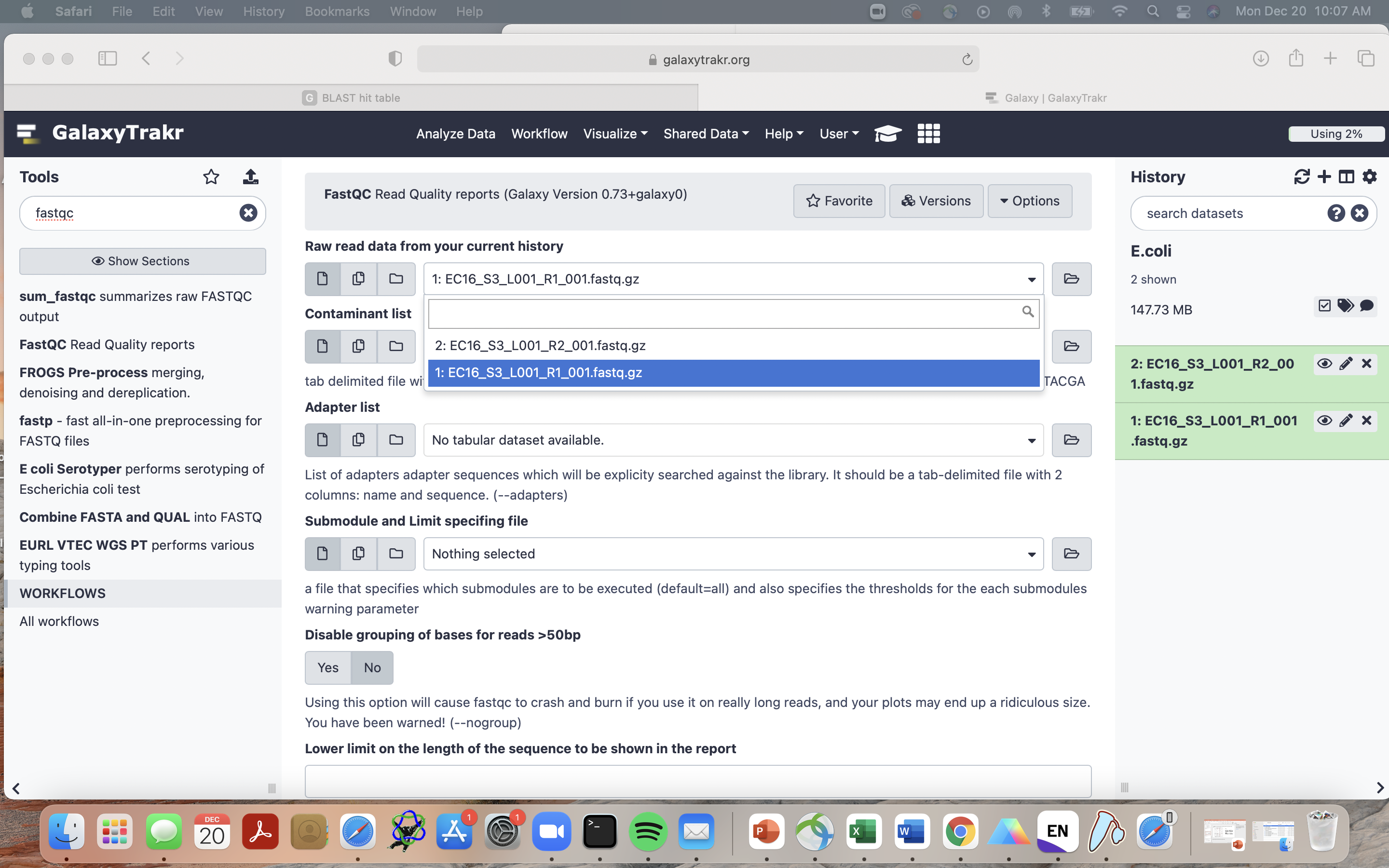


A green message will appear if the program has been added to the queue of GalaxyTrakr to get run, and 2 more elements will appear in your History: (i) FastQC on data 1: Webpage, and (ii) FastQC on data 1: RawData.


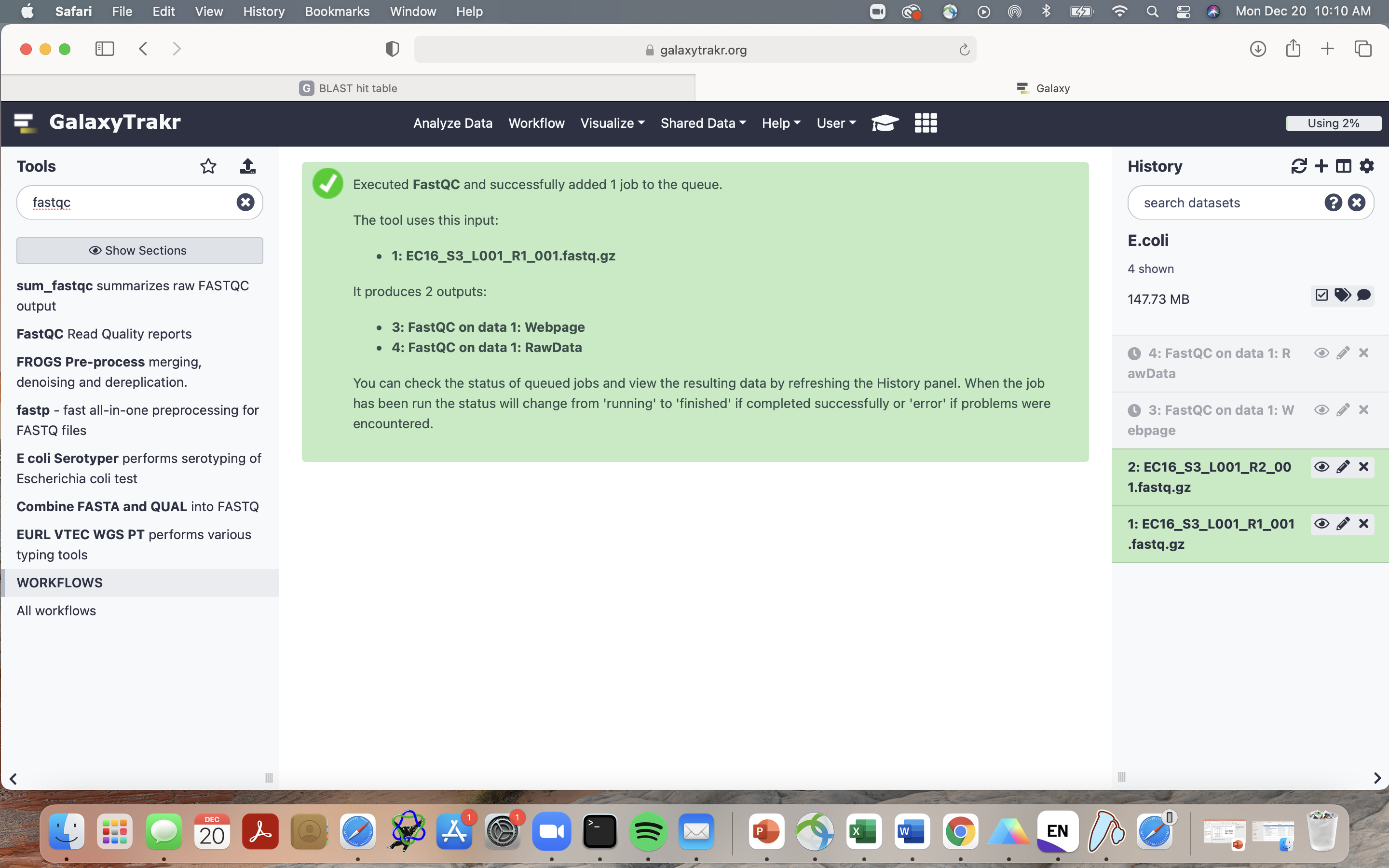


Click on “FastQC on data 1: Webpage” You can visualize your report in GalaxyTrakr by clicking the “eye” button or download your report by clicking the “download” button.


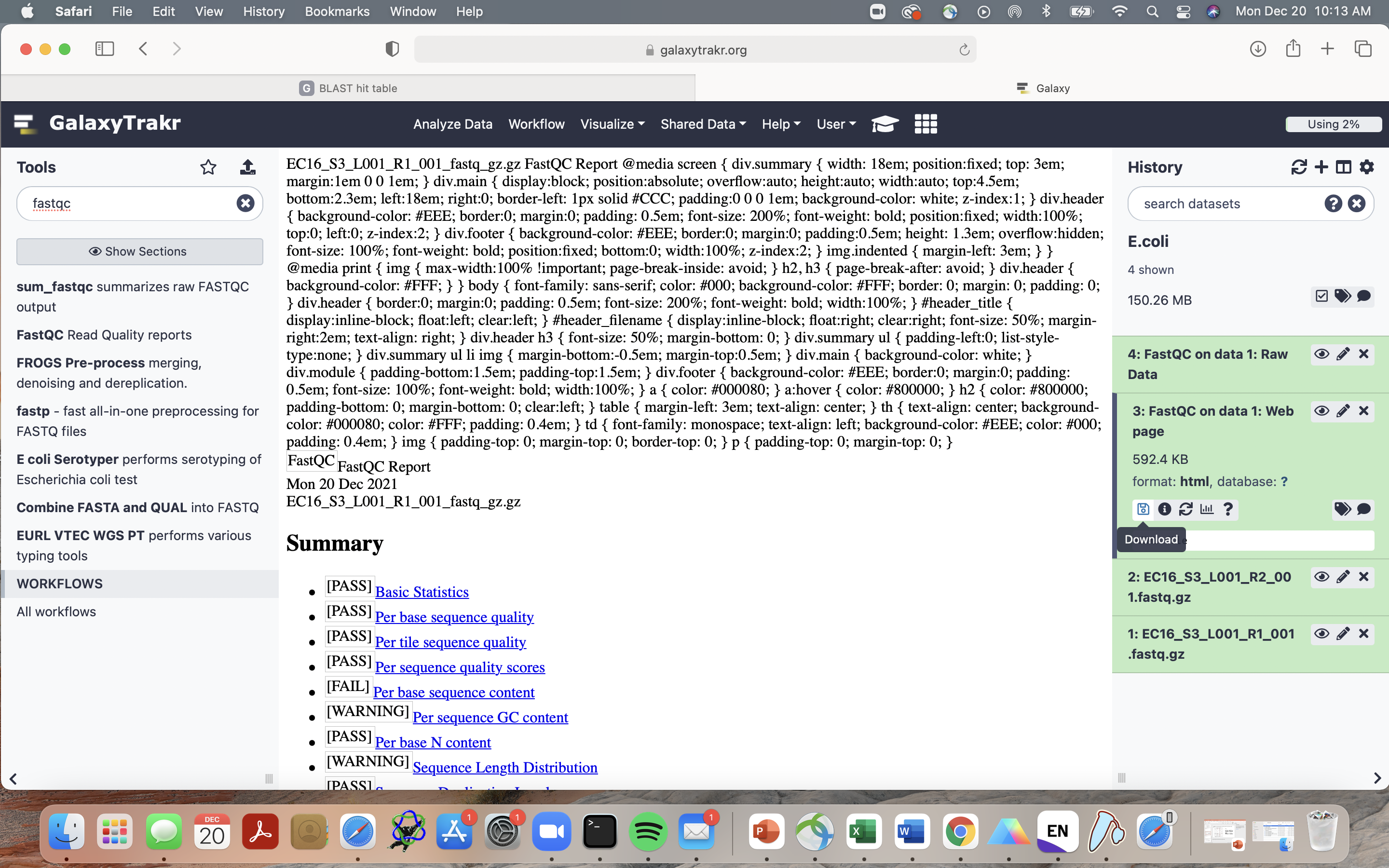


A folder will be downloaded to your download directory in your local computer. Open the file with the name “FastQC_on_data_1_Webpage.html.” The first three categories, “Basic Statistics,” “Per base sequence quality,” and “Per sequence quality scores,” are what require your attention. If these tests have a green tick, that means that the quality of your reads is good, and you can continue with your analysis. “Basic Statistics” gives you a summary of your analysis. “Per base sequence quality” shows an overview of the range of quality values across all bases at each position of the read (the higher the score, the better the quality). Finally, “Per sequence quality scores” shows the distribution of quality scores of your reads (you want to observe


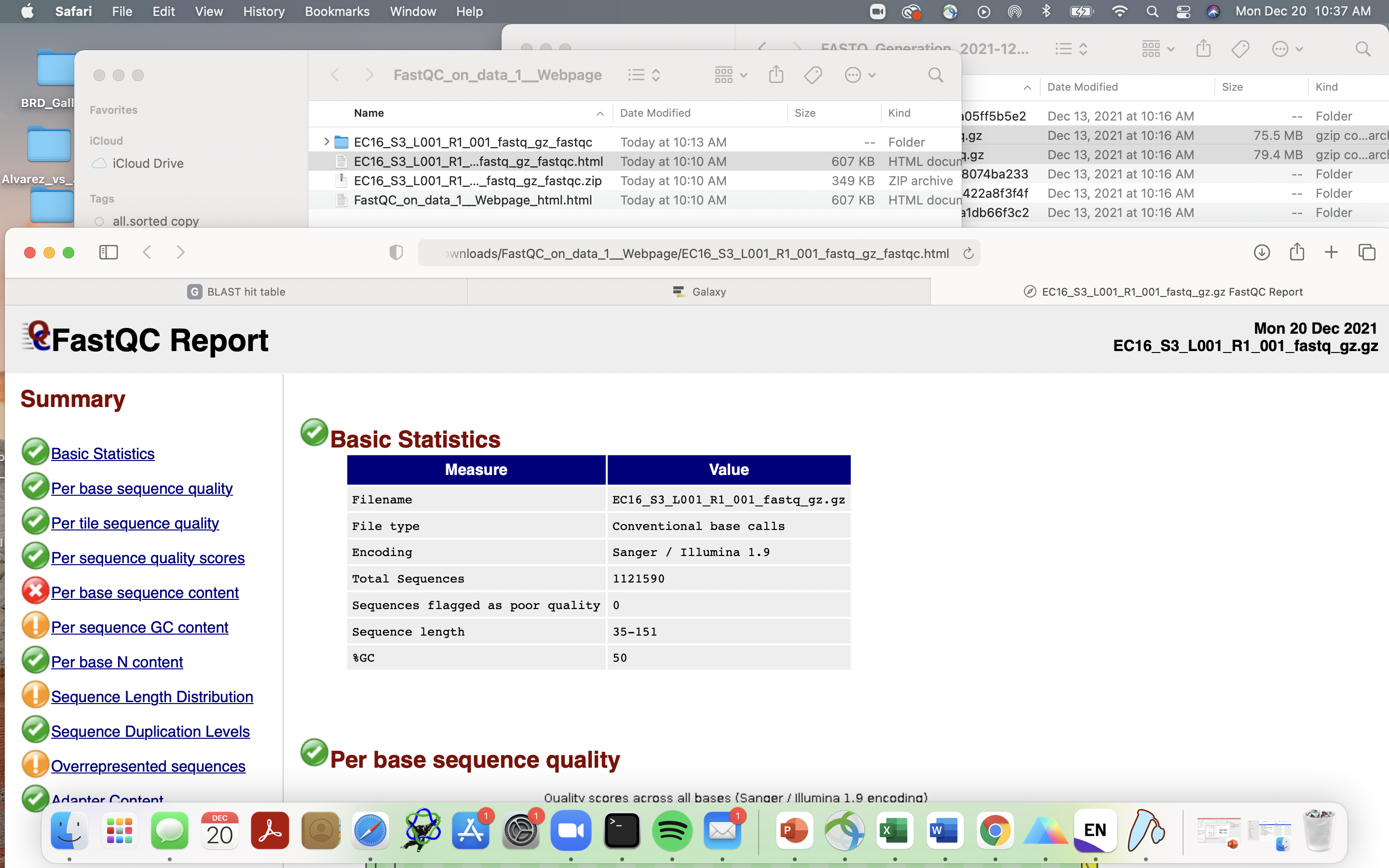


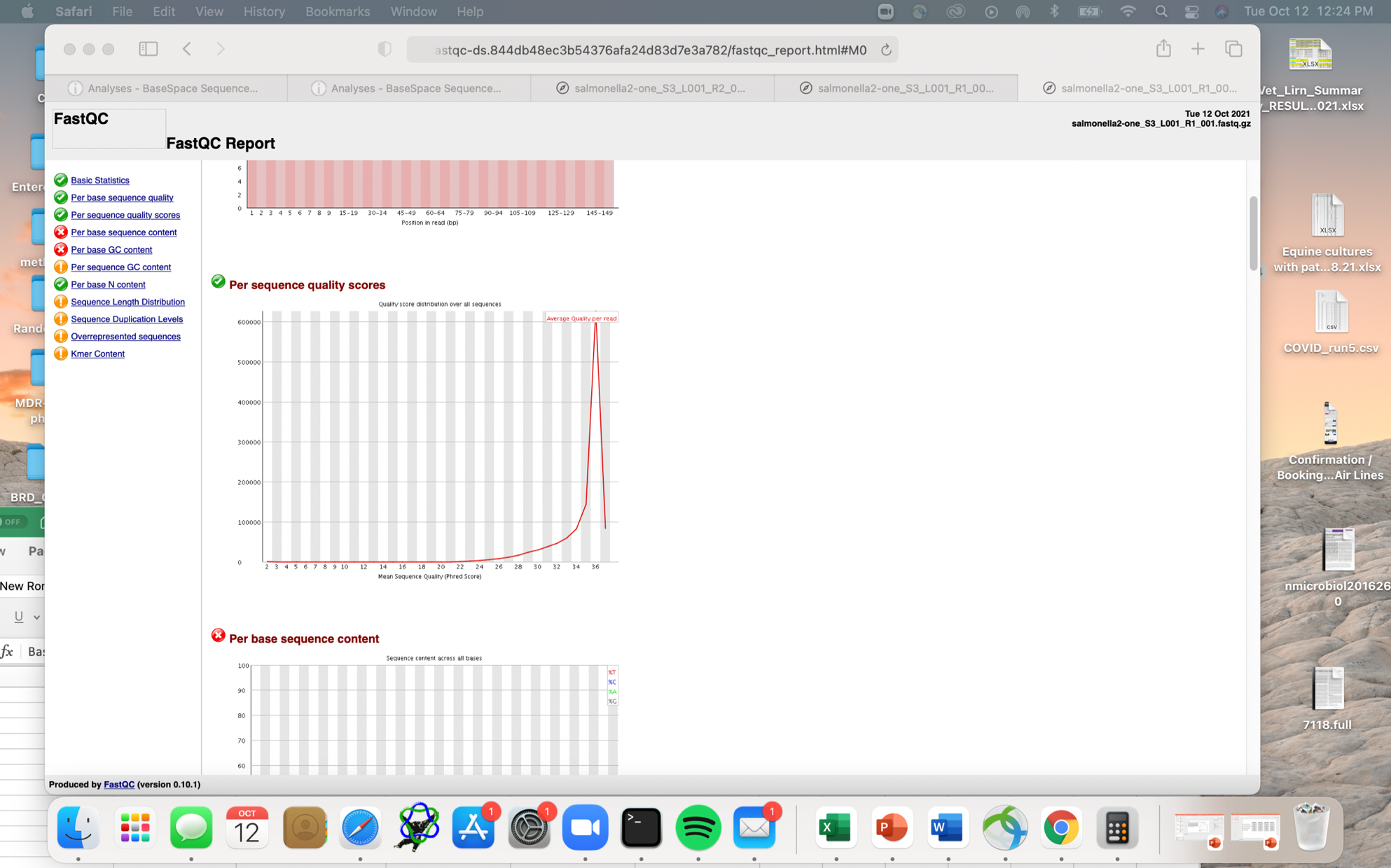

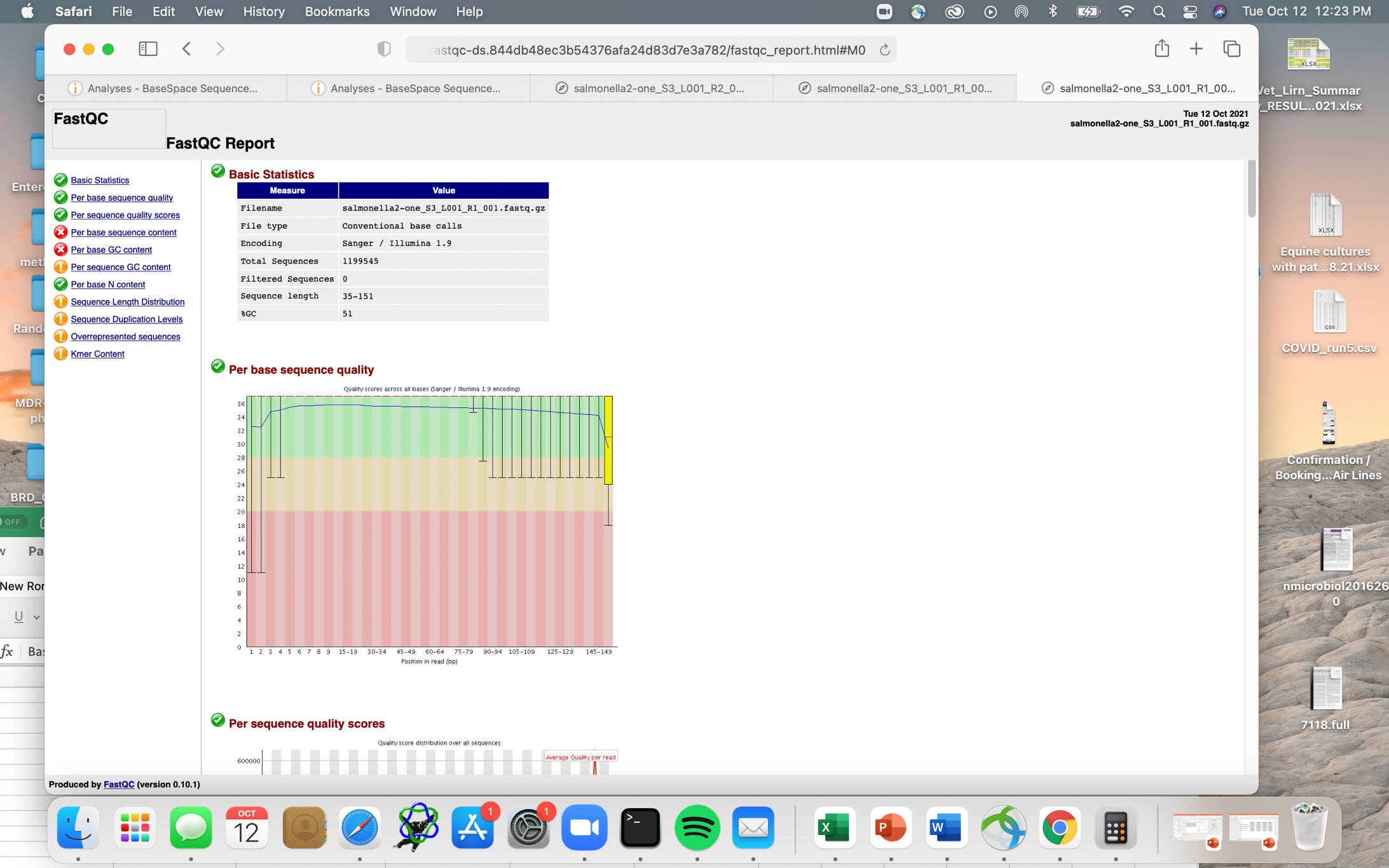


**RAW READS TRIMMING AND FILERING**

5. We will use Trimmomatic to look at the quality of the raw reads before starting the analysis. Under “Tools” write “Trimmomatic” in the search tools space. A list of available programs will appear right under. Click on “Trimmomatic flexible read trimming for Illumina NGS data”. Under “Single-end or paired-end reads?” select for “Paired-end (two separate input files)”. Under “input FASTQ file (R1/first of pair), select your raw read R1 file from the available list. Under “input FASTQ file (R2/second of pair), select your raw read R2 file from the available list.


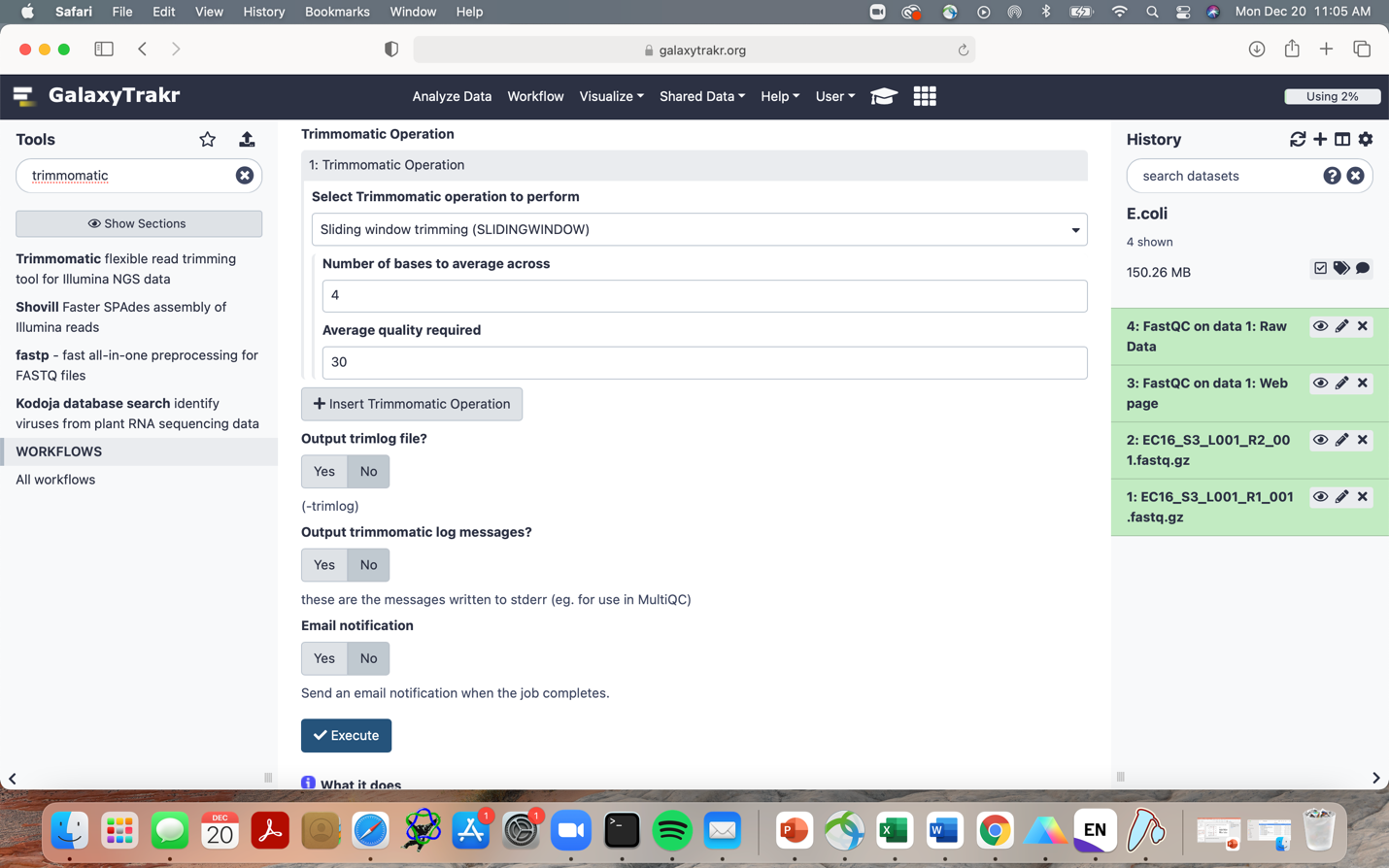

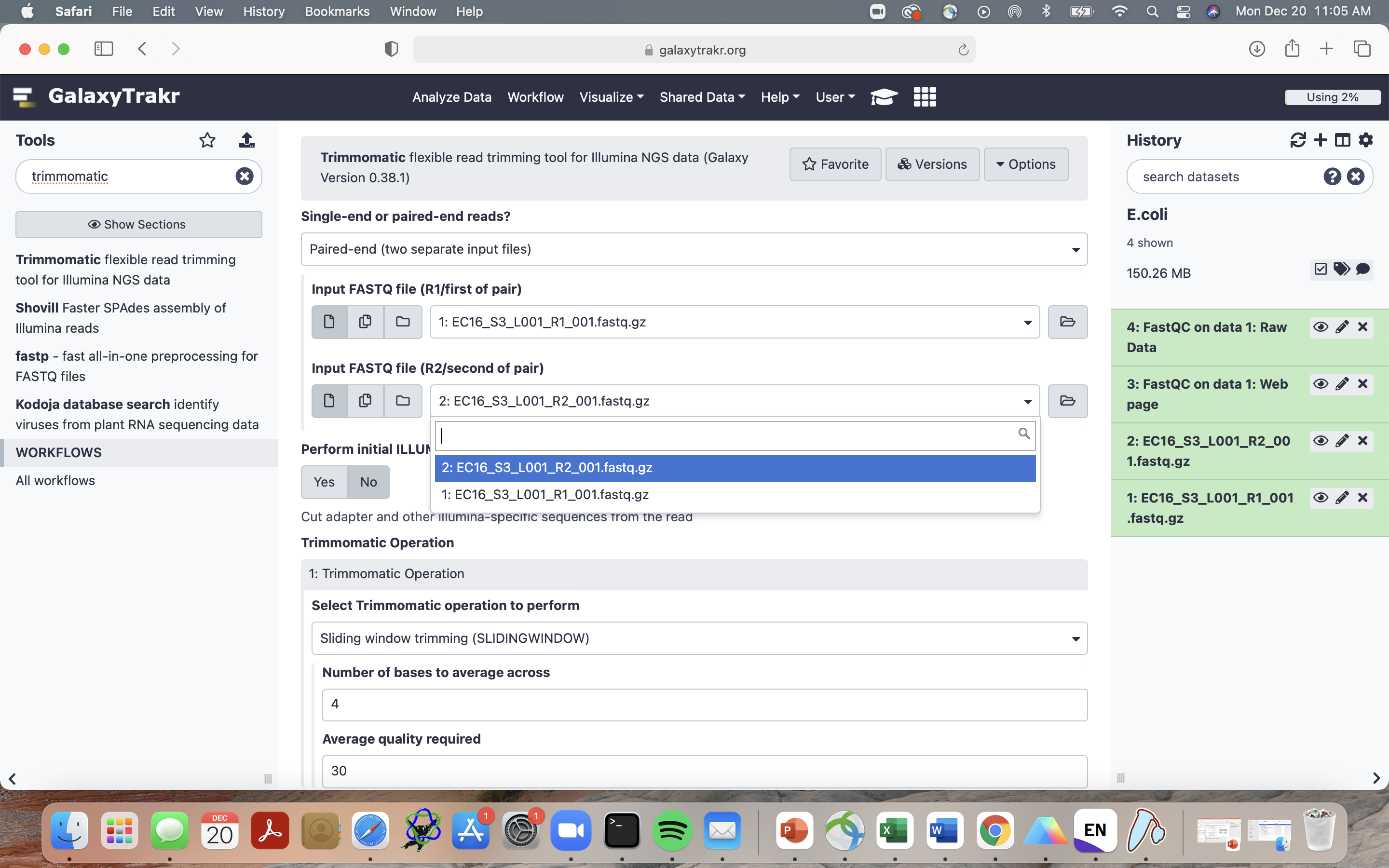


Under “Trimmomatic Operation”, select “Sliding window trimming (SLIDINGWINDOW) with 4 as “Number of bases to average across” and 30 as “Average quality required”. Leve every other setting as default and press “Execute”.

A green message will appear if the program has been added to the queue of GalaxyTrakr to get run, and 4 more elements will appear in your History: (i) Trimmomatic on R1 (R1 paired); (ii) Trimmomatic on R2 (R2 paired); (iii) Trimmomatic on R1 (R1 unpaired); and (iv) Trimmomatic on R2 (R2 unpaired). R1 paired and R2 paired correspond to the files containing your filter paired reads that you will use to continue with your analysis. R1 unpaired and R2 unpaired correspond to the reads that did not pass the filter.


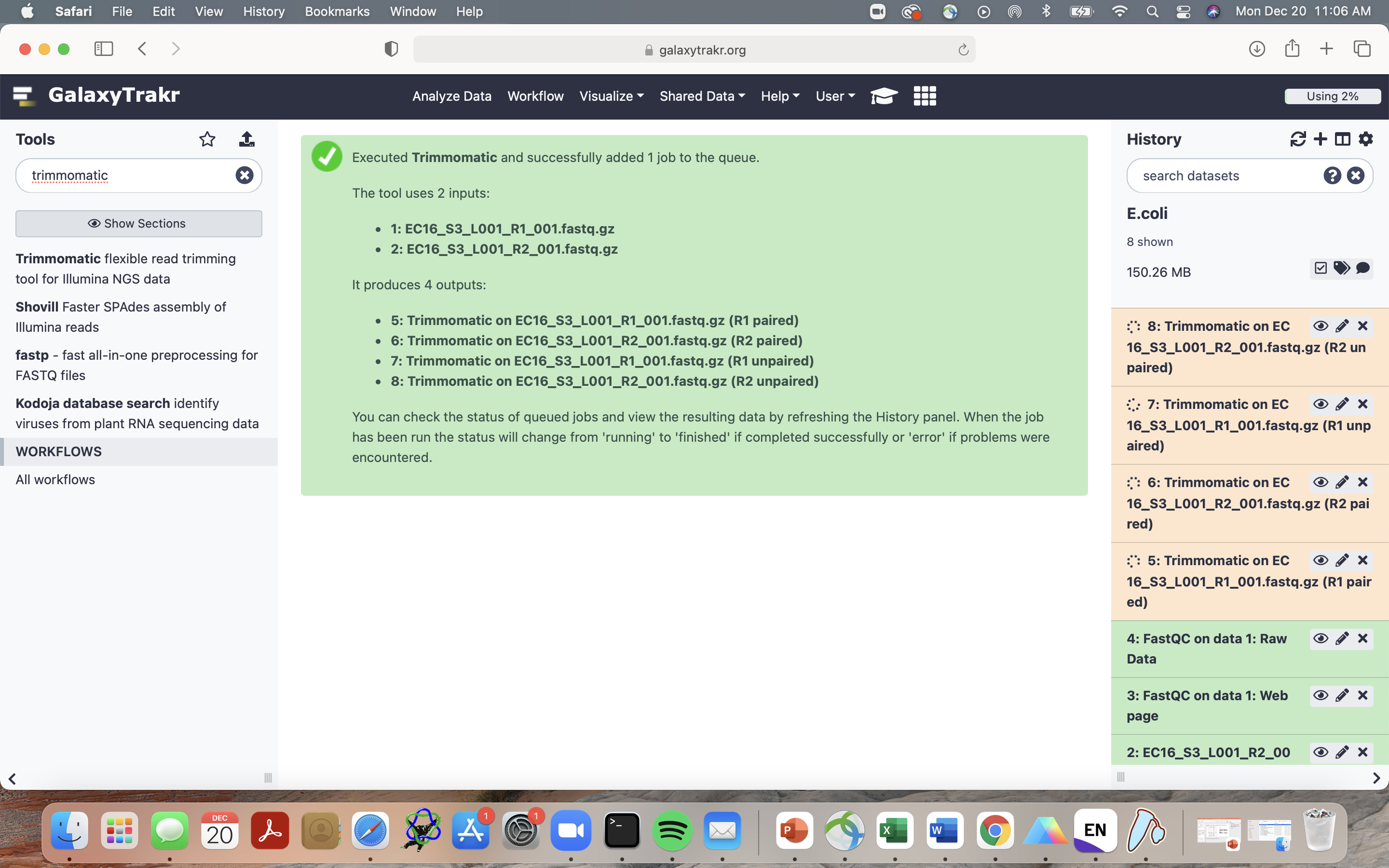


**READ ASSEMBLY**

6. We are going to use SPAdes to perform a de novo assembly of the filter and trimmed reads. Under “Tools” write “SPAdes” in the search tools space. A list of available programs will appear right under. Click on “SPAdes genome assembler for regular and single-cell projects”. Select for “careful correction-Yes” and “Automatically chose k-mer values-Yes”.


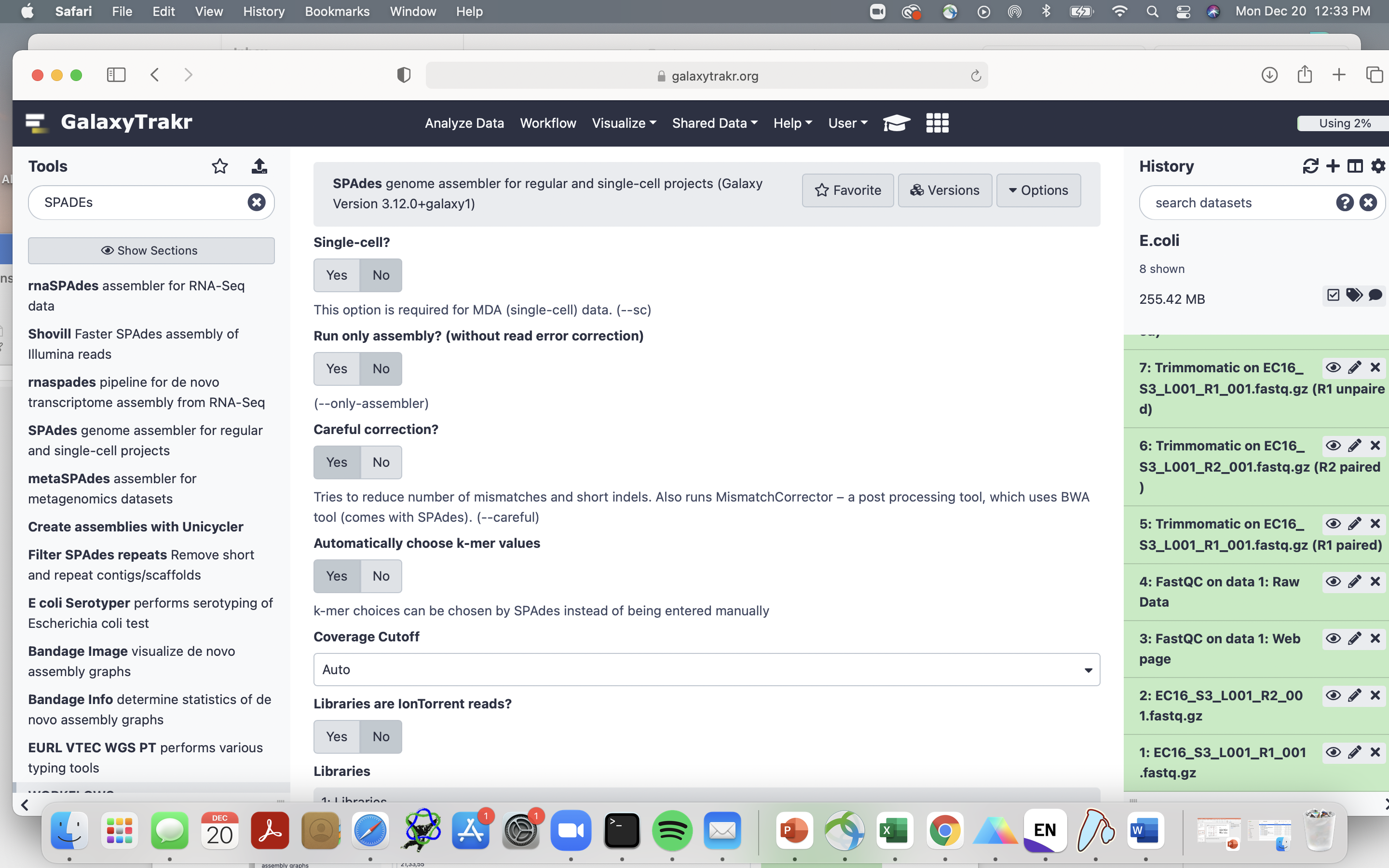


Then, select “Paired-end/ Single reads” as your Library type and Orientation “-><- fr”. Under files, select “Separate input files”. Under Forward reads, you may want to choose the file containing the forward reads that you previously clean and filtered (R1 paired). Under Reverse reads, select the file containing the reverse filtered reads (R2 paired).


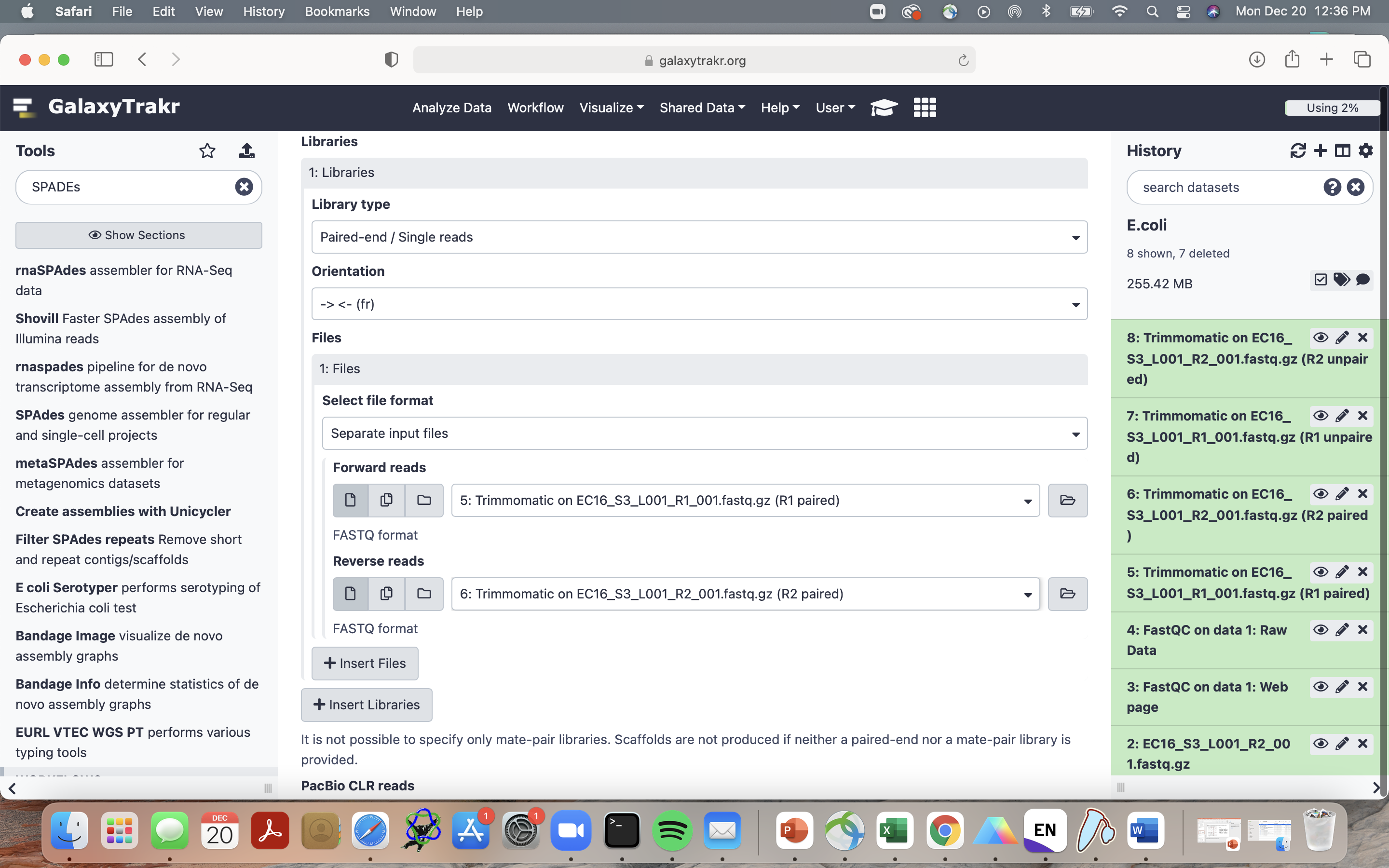


You are working with Illumina reads. Hence, ignore the fields for “PacBio CLR reads”, “Nanopore reads”, and “Sanger reads”. Ignore also the “Trusted contigs” and “Untrusted contigs” fields because we are performing an assembly de novo (not reference guided). It is very important that you do not click to any of the files in the above-mentioned fields. If you do, you will be selecting them to get incorporated into the analysis.


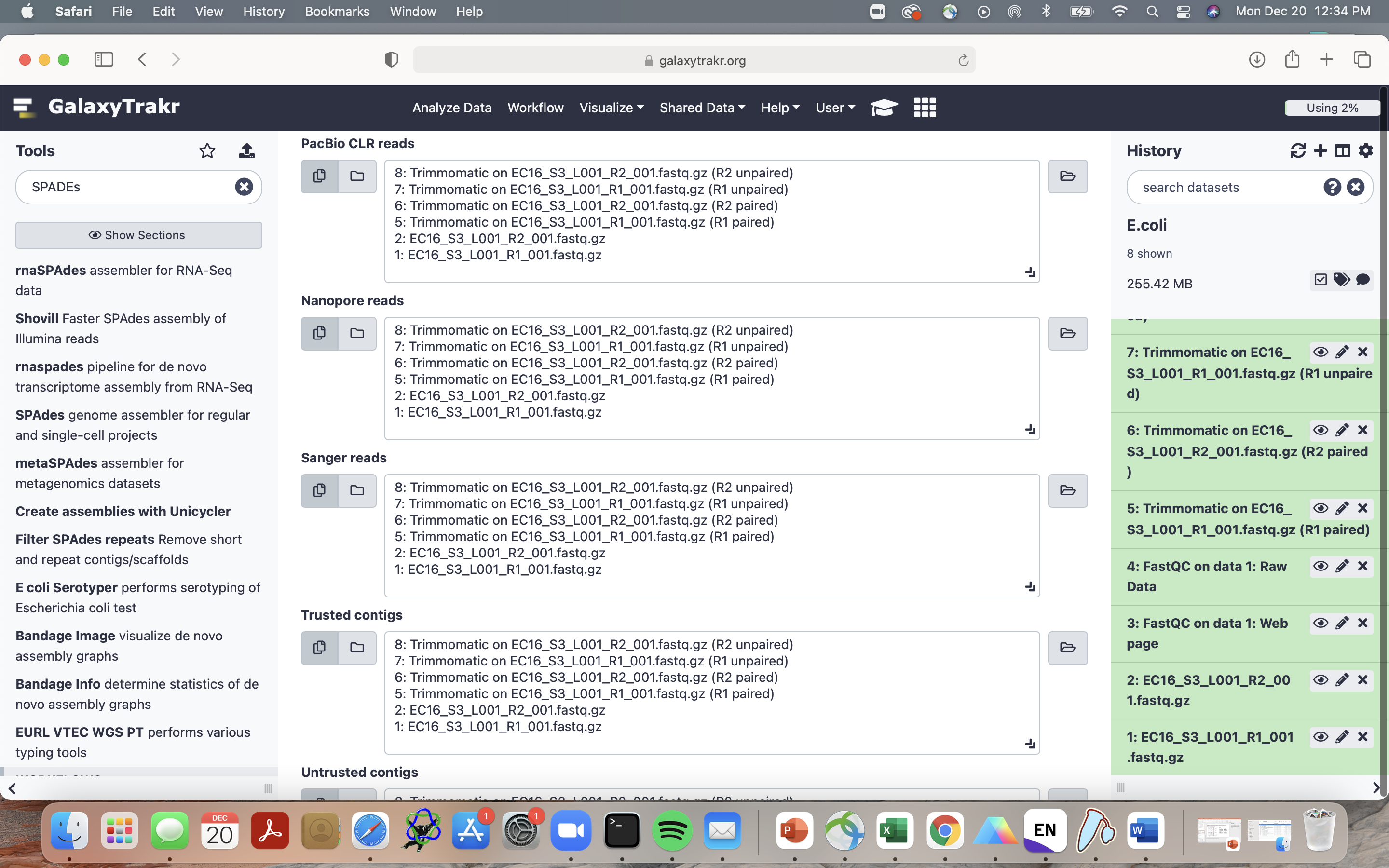


Leave the rest of the settings as they are (“Output final assembly graph”-No and “Email notification” – No). Then click execute. Five files will result: (i) contig stats, (ii) scaffold stats, (iii) contigs.fasta, (iv) scaffolds.fasta, and (v) data.log. You will use any of the fasta files to continue with your analysis.


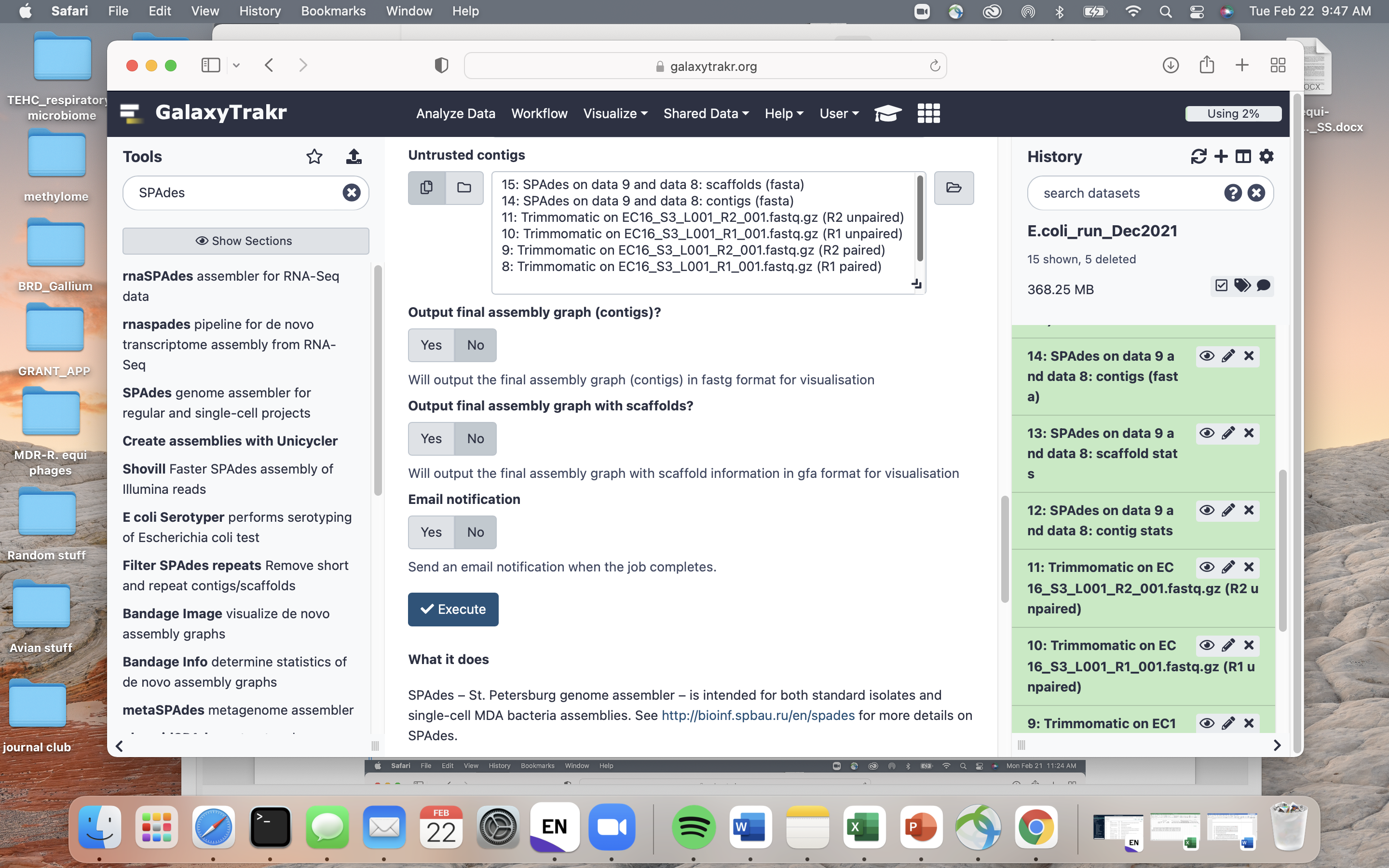


**ASSEMBLY REPORT**

7. We will use Quast to assess the quality of the read assembly into contigs or scaffolds. Under “Tools” write “quast” in the search tools space. A list of available programs will appear right under. Click on “Quast” genome assembly quality. Choose any of the two fasta files (scaffolds.fasta or contigs.fasta) to perform the analysis. Select Genome under “Type of assembly”, and No reference genome.


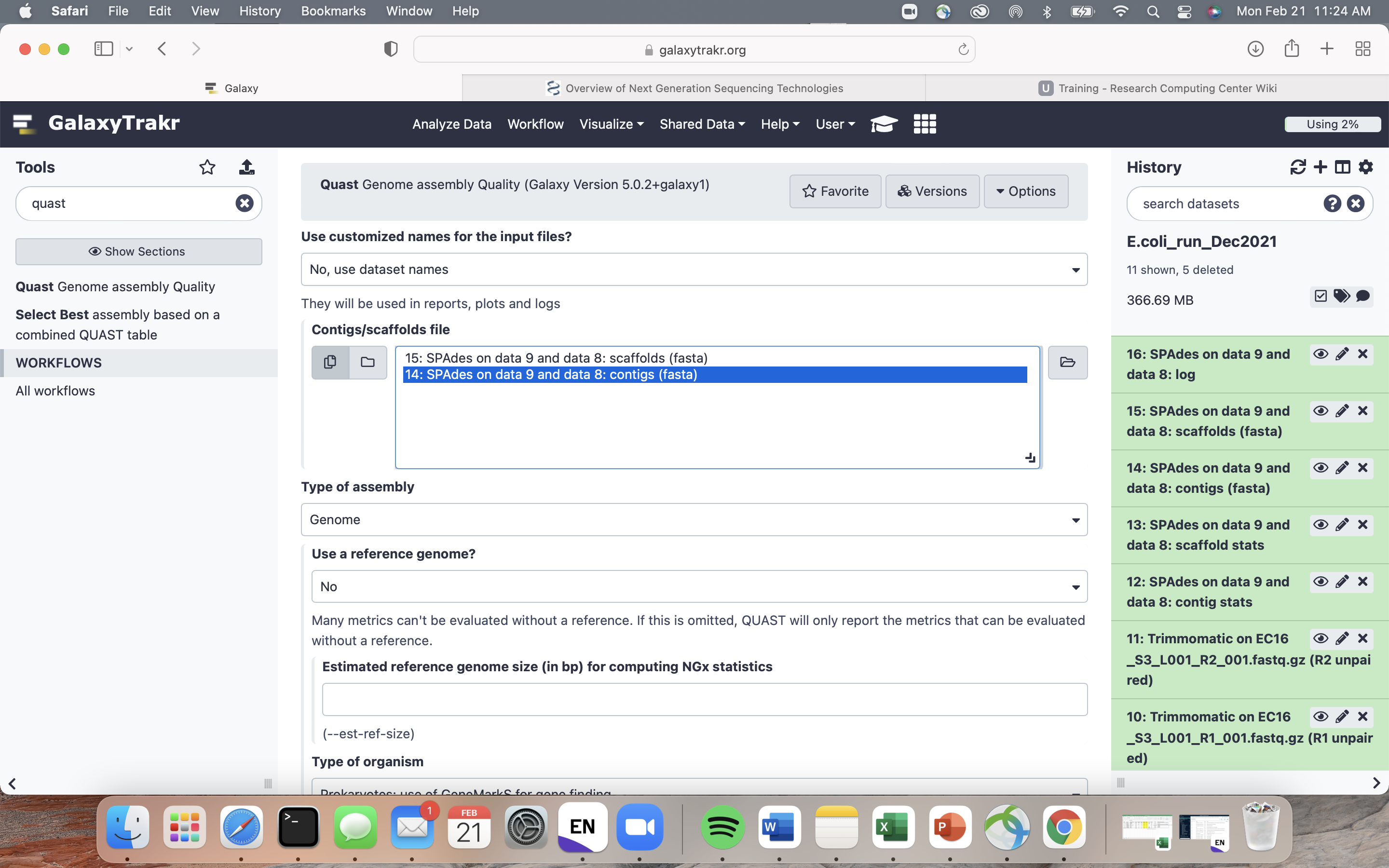


Finally, choose Prokaryotes as a “Type of organism”, leave all the other settings as default and click “Execute”. Four files will result: (i) tabular report, (ii) report.pdf, (iii) report.html and (iv) log.


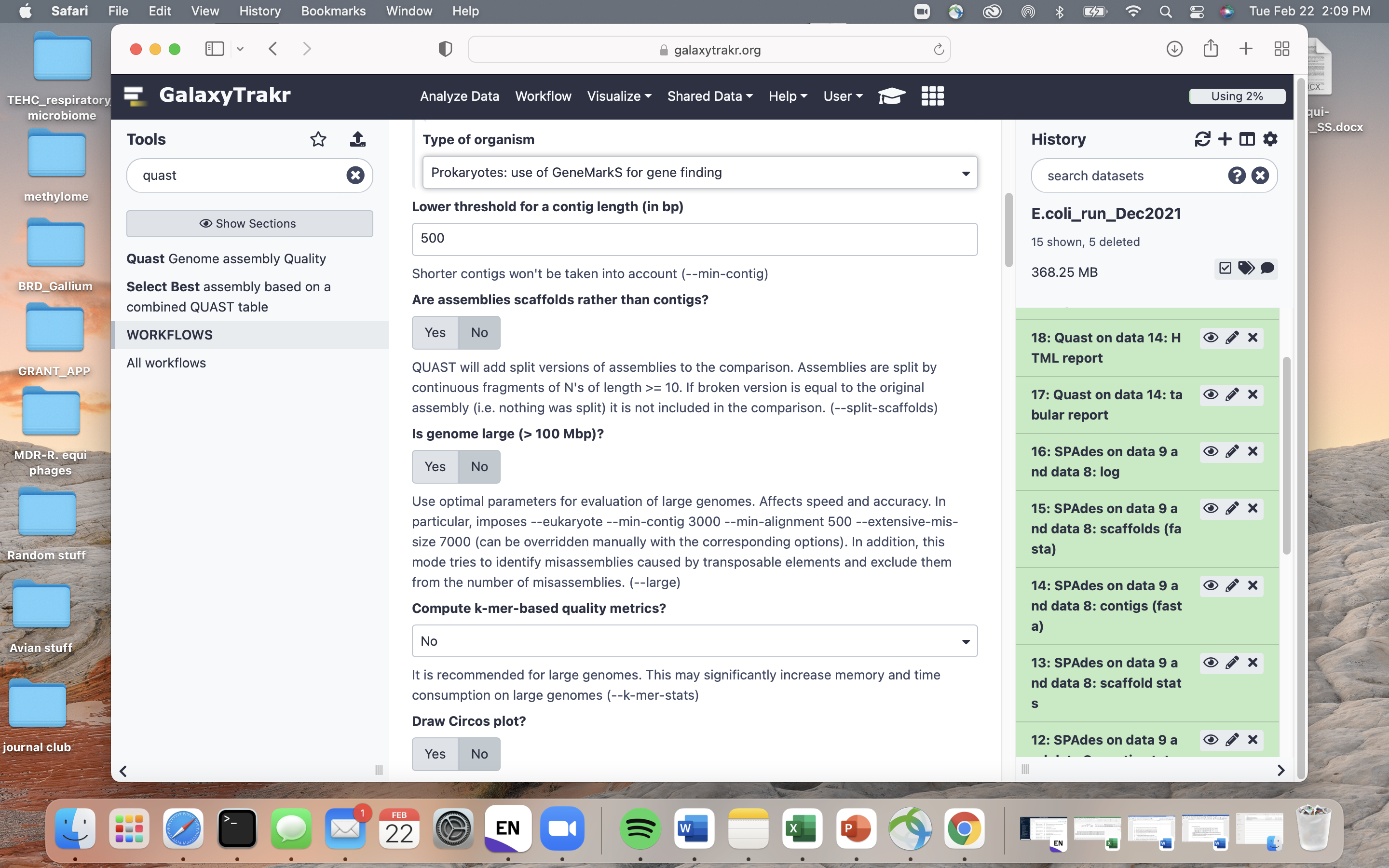


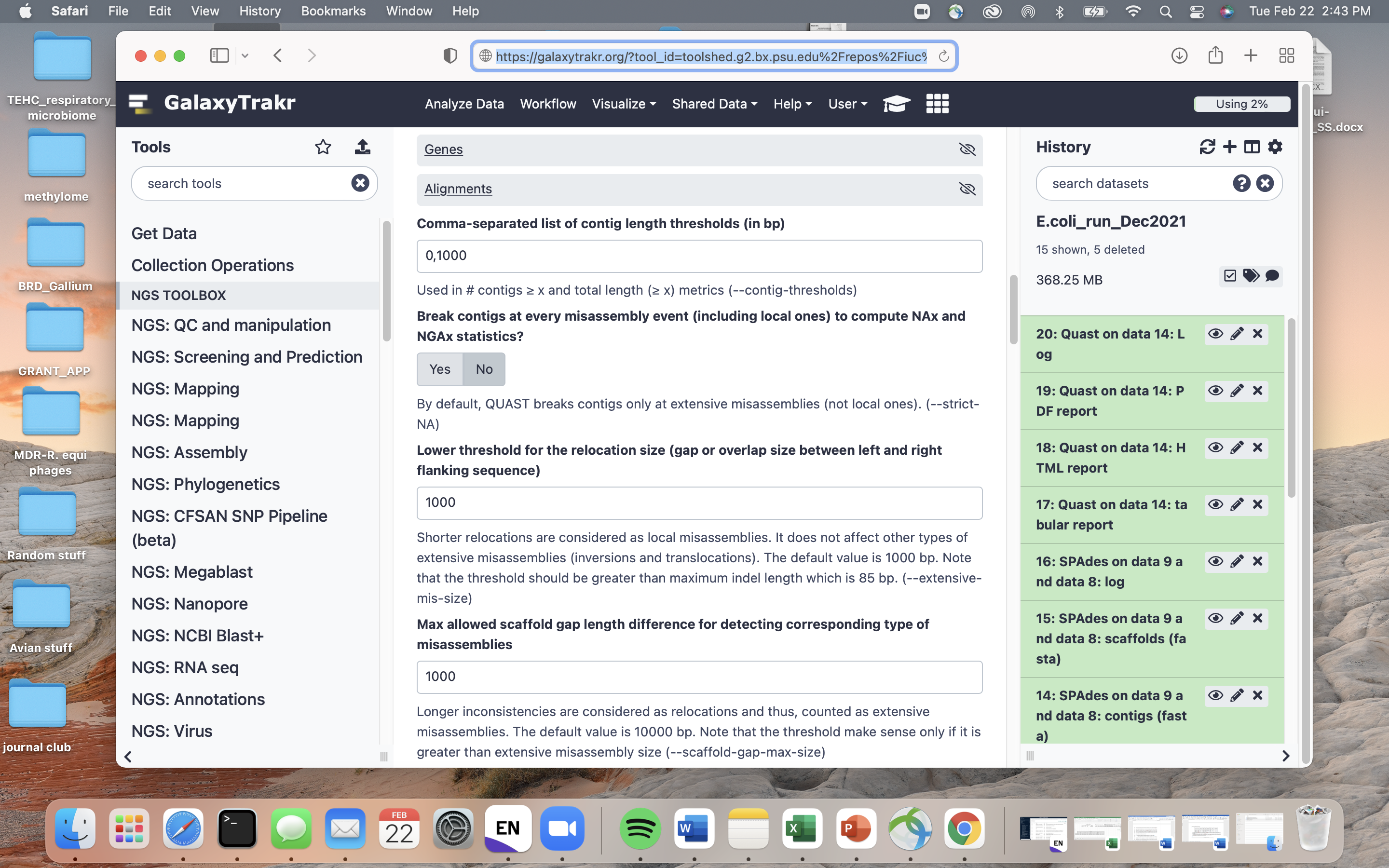


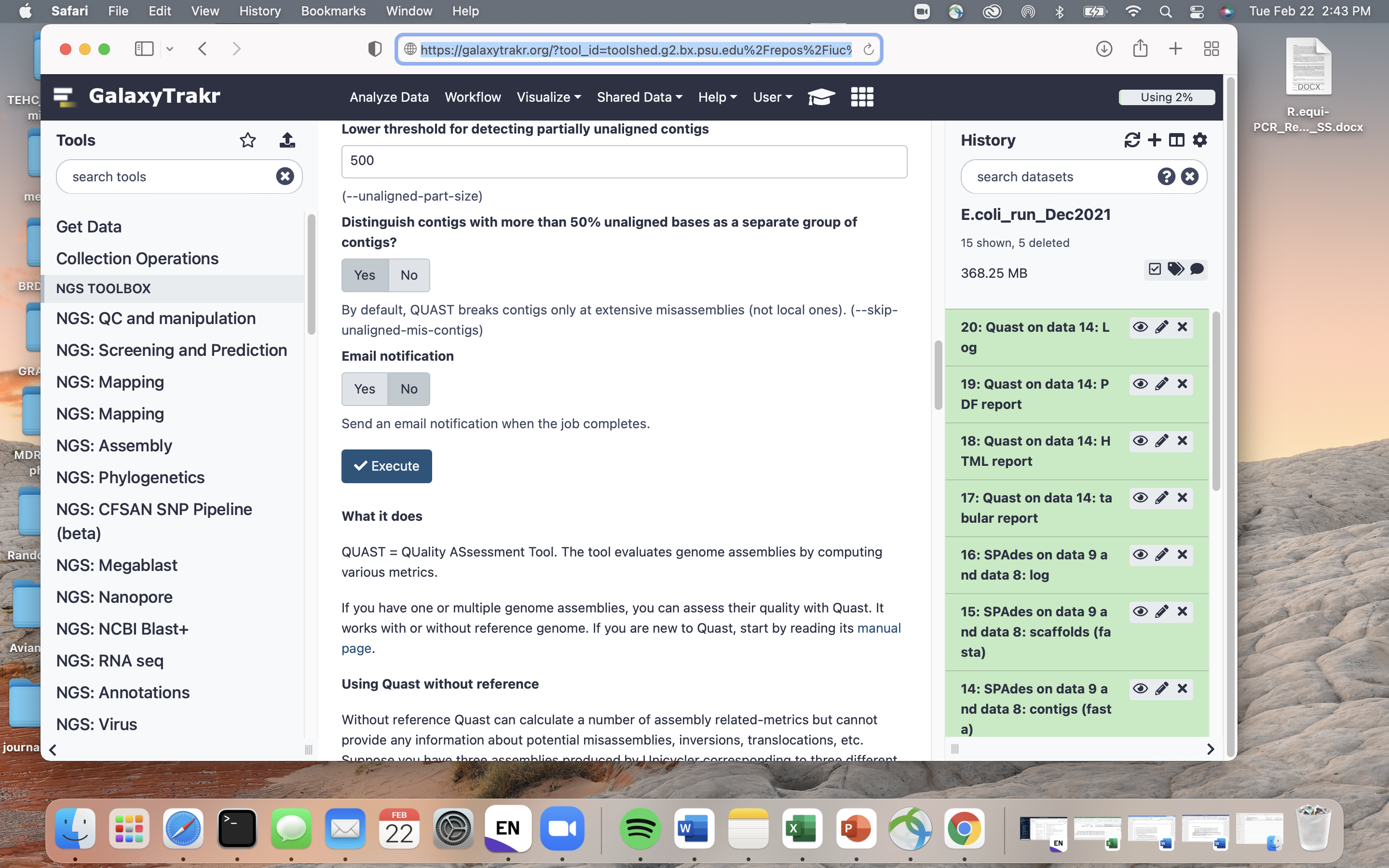


Look at your assembly quality report by clicking on the eye-shape button in “Quast tabular report”.


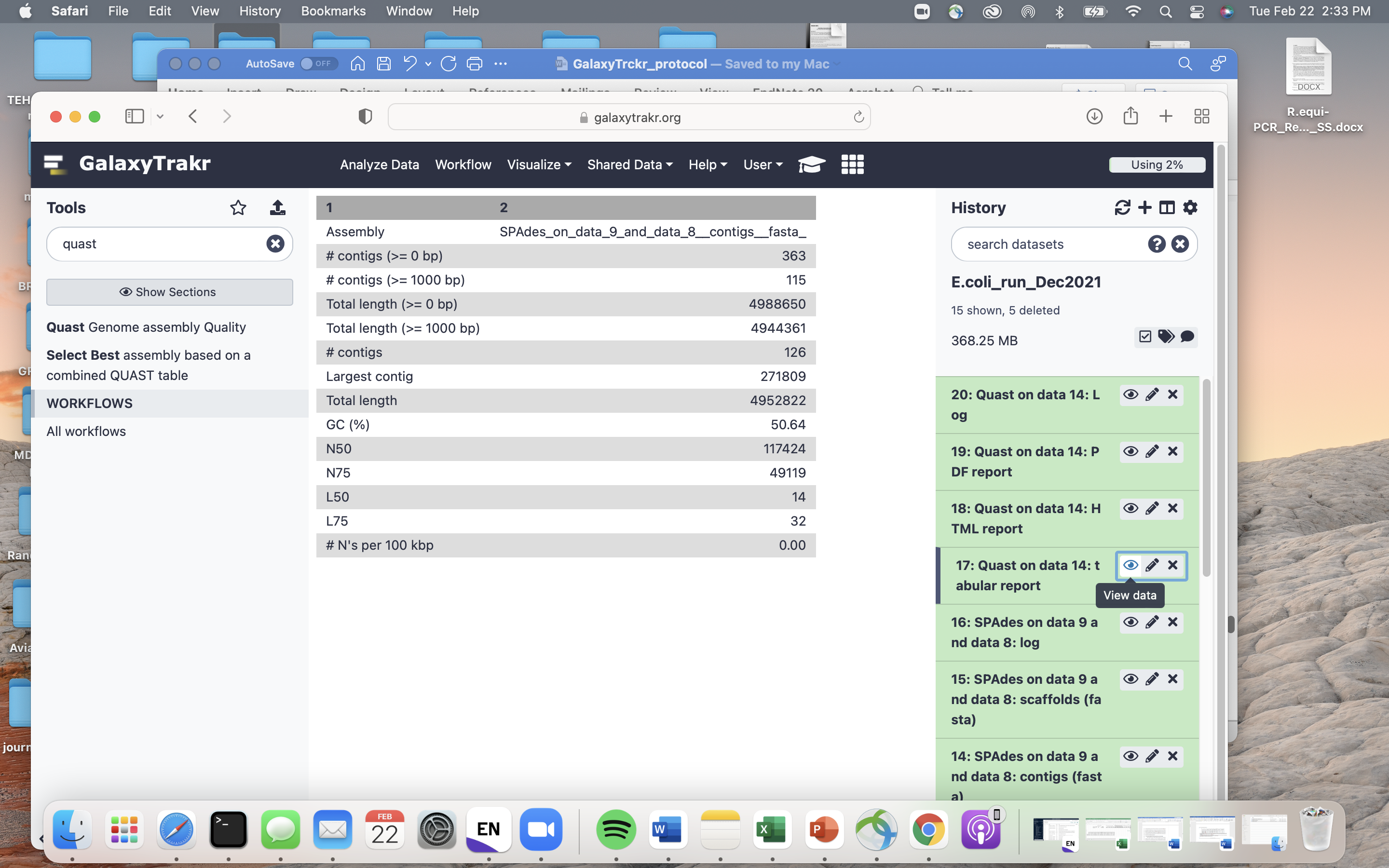


The main Assembly Statistics appear on a table in which rows describe:

-“ # contigs (>=0 bp)” is total number of contigs of length ≥ 0 bp

-“# contigs (>=1000 bp)” is total number of contigs of length ≥ 1000 bps

- “Total length (>=0 bp)” is the total number of bases in in contigs of length ≥ 0 bp

- “Total length (>=1000 bp)” is the total number of bases in contigs of length ≥ 1000 bps

- “# contigs” is the total number of contigs in the assembly.

- “Largest contig” is the length of the longest contig in the assembly.

- “Total length” is the total number of bases in the assembly.

- “GC (%)” is the total number of G and C nucleotides in the assembly, divided by the total length of the assembly.

- “N50” is the length for which the collection of all contigs of that length or longer covers at least half an assembly. [the bigger the number, the better the quality of the assembly]

- “N75” is the length for which the collection of all contigs of that length or longer covers at least 75% of an assembly. [the bigger the number, the better the quality of the assembly]

- “L50” is the minimal number of contigs that cover half the assembly. [the smaller the number, the better the quality of the assembly]

- “L75” is the minimal number of contigs that cover 75% of the assembly. [the bigger the number, the better the quality of the assembly]

This same applied to the scaffolds.fasta file, that contains scaffolds (bigger contigs). You can use either or the files (contigs.fasta and scaffold.fasta) for bacteria species and ARGs identification.

**BACTERIA SPECIES IDENTIFICATION**

We will use sendsketch to assess the quality of the read assembly into contigs or scaffolds. Under “Tools” write “sendsketch” in the search tools space. A list of available programs will appear right under. Click on “sendsketch identifying species using sketch”. Choose any of the two fasta files (scaffolds.fasta or contigs.fasta) to perform the analysis. Select “Only report record with at least this ANI” of 0.99 and “Only report record with at least this KWID” of 0.95. These two setting narrow your search for sequences in the database above 95% average nucleotide identity (ANI) and well-known ID (KWID). Leave any other setting as default (“No”) and click Execute


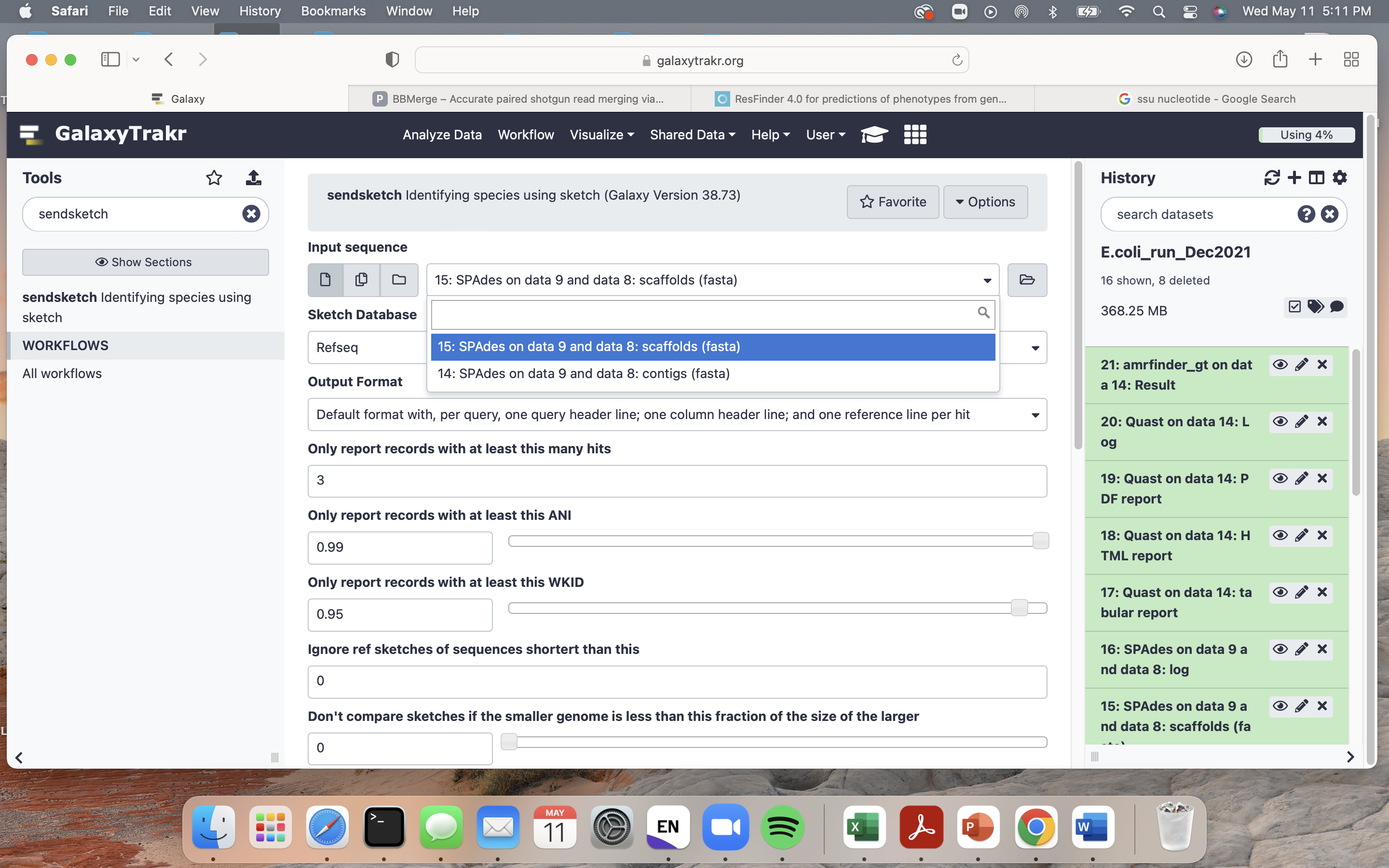


Look at your species identification report by clicking on the eye-shape button in “sendsketch Results”.

**
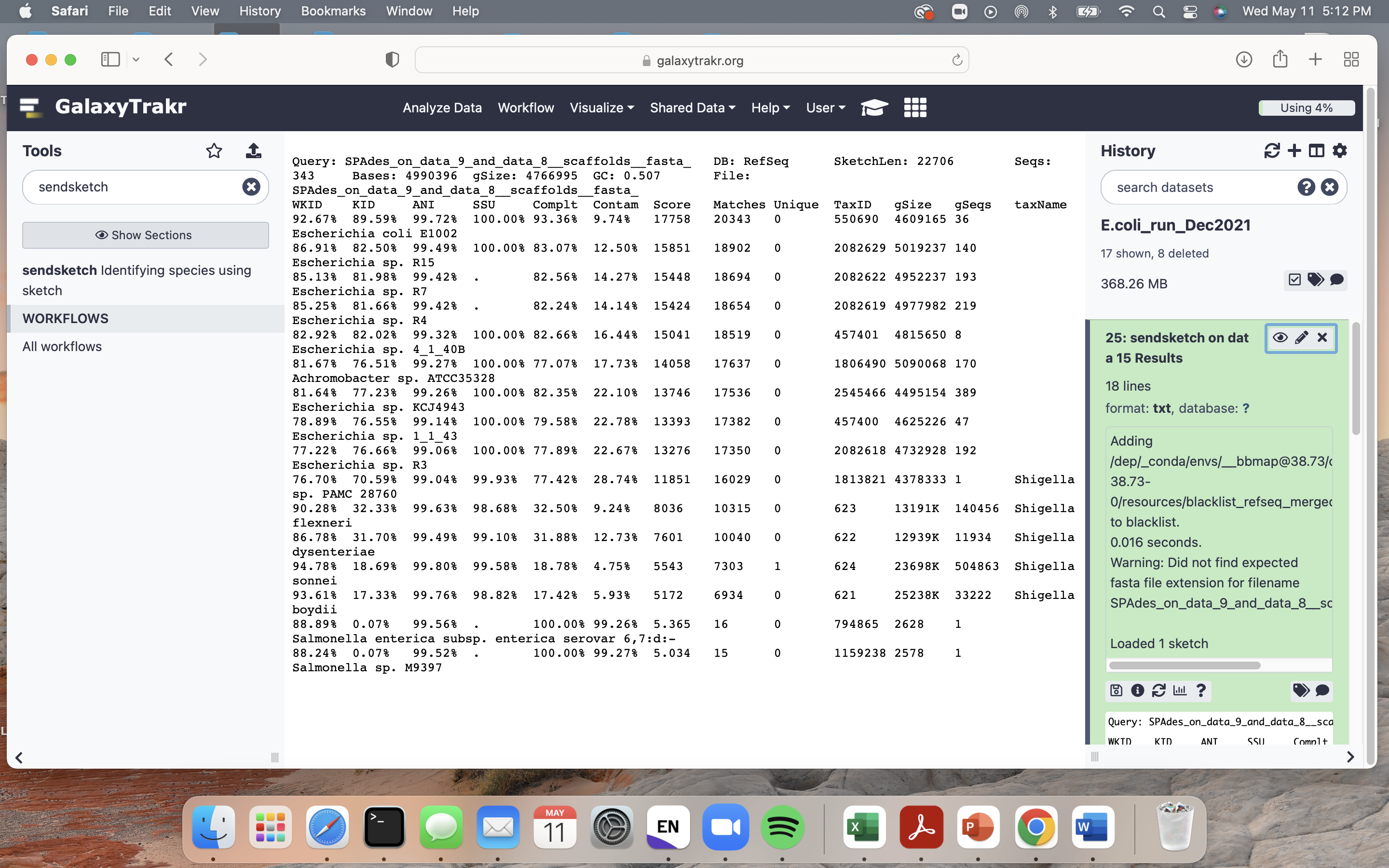
**

Looking closer at the table:

**
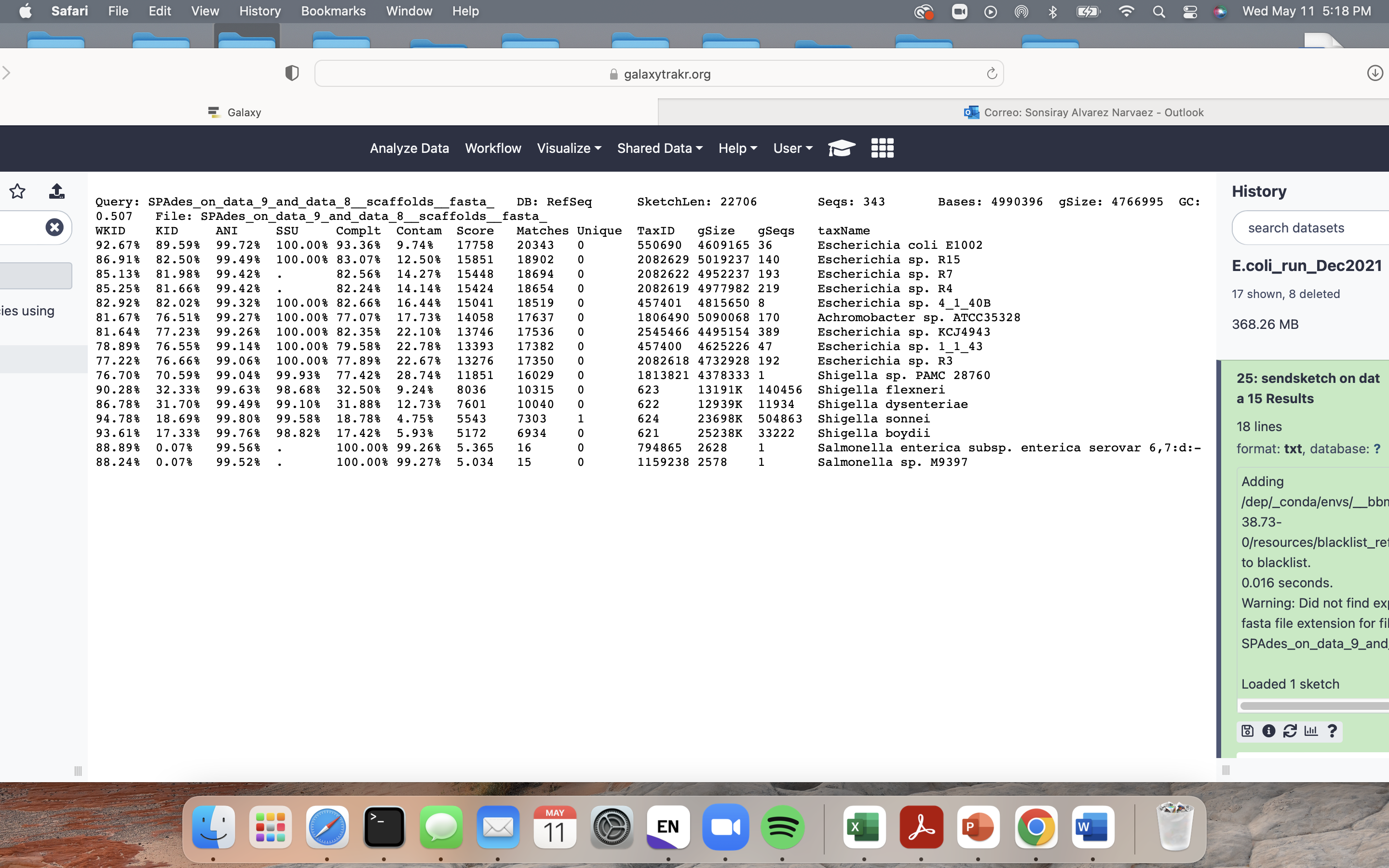
**

Of interest:

ANI: average nucleotide identity between the target genome and the genome in the database. You want this value above 99%

SSU: Similarity between Small subunit (SSU) ribosomal ribonucleic acid or 16SrRNA gene from the target genome and the genome in the database.

Complt: How much or your target genome covers the genome in the database. You want this value above 90%

Contam: How much of the database genome is not covered by the target genome (100% - Complt)

taxName: Name of the identified species

**ANTIMICROIBAL RESISTANCE GENES IDENTIFICATION**

We will use AMRfinder to assess the quality of the read assembly into contigs or scaffolds. Under “Tools” write “amrfinder” in the search tools space. A list of available programs will appear right under. Click on “amrfinder_gt identifies AMR genes using either protein annotations or nucleotide sequences”. Choose any of the two fasta files (scaffolds.fasta or contigs.fasta) to perform the analysis. Leave any other setting as default (“No”) and click Execute.


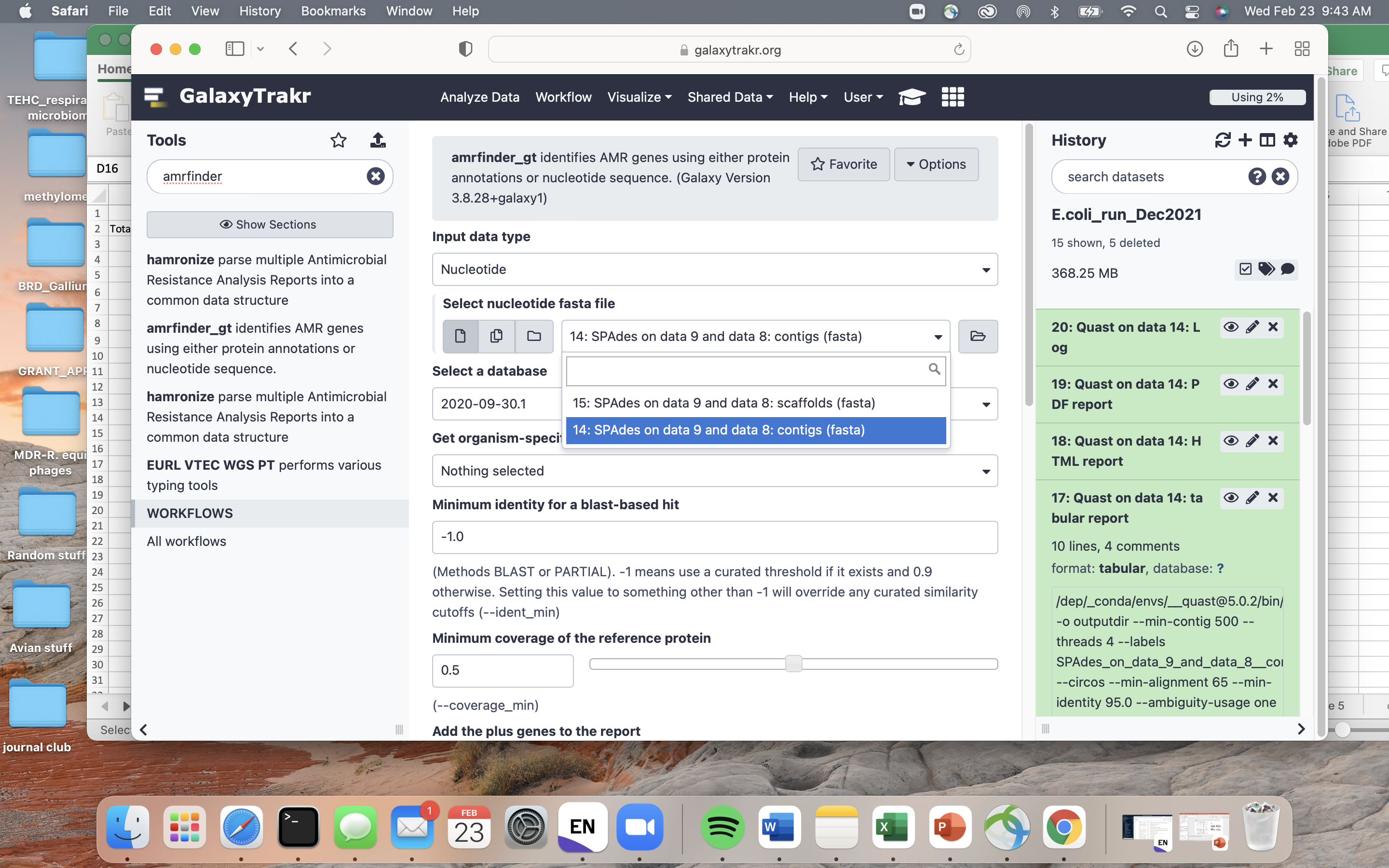


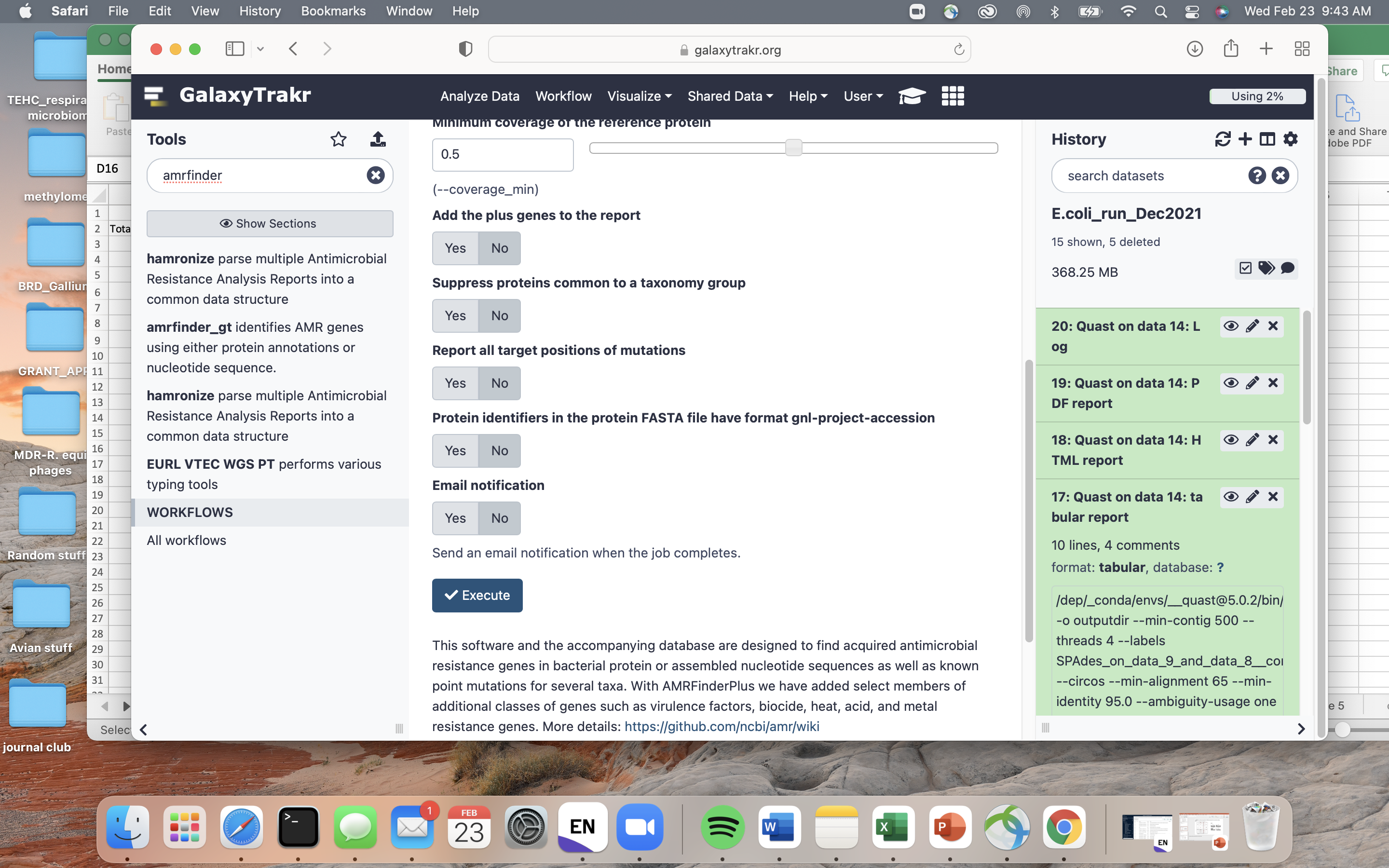


Look at your ARG report by clicking on the eye-shape button in “amrfinder_gt: Results”.


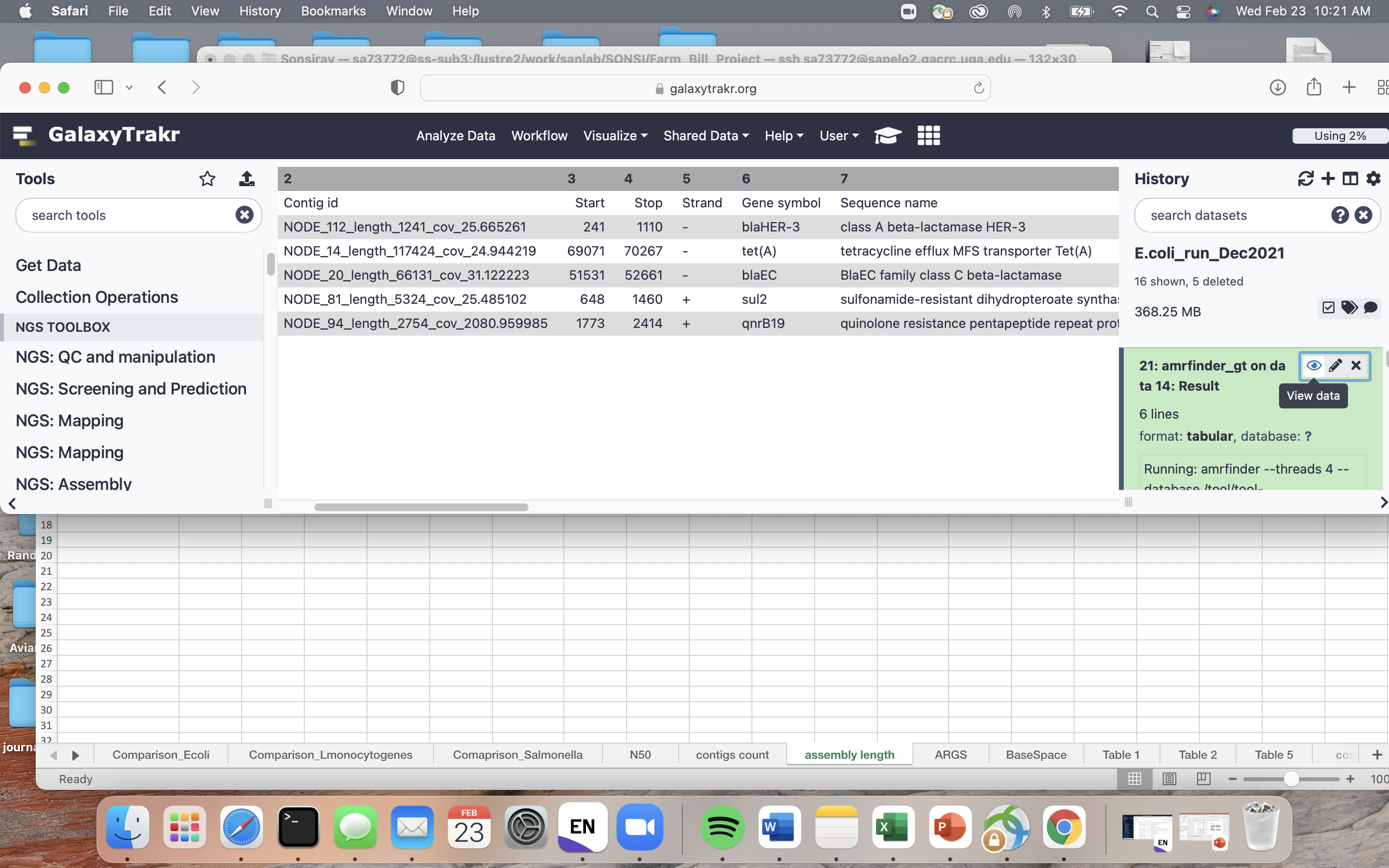


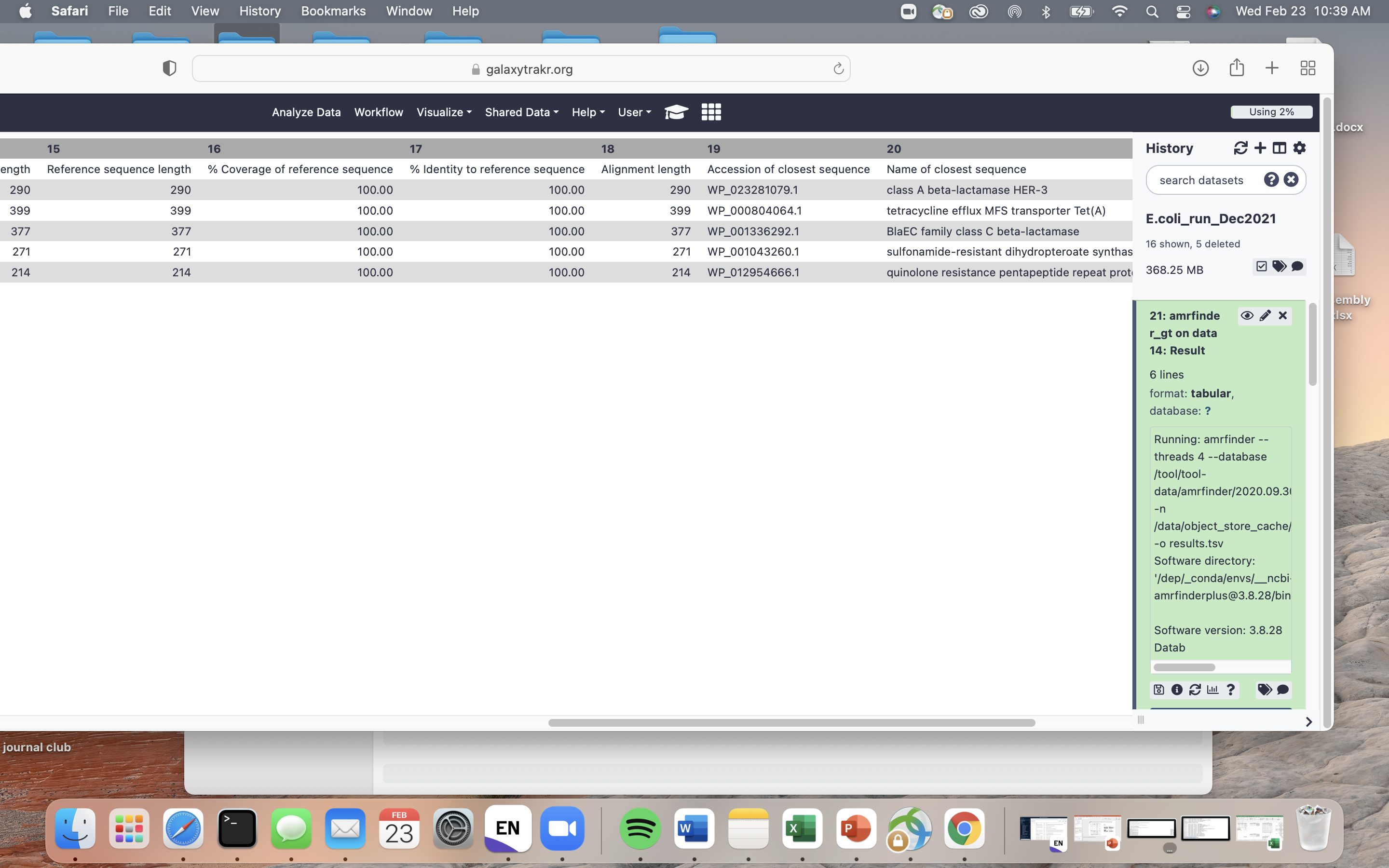


Of interest in the table:

- Column 6 - ARG gene symbol
- Column 7 - ARG sequence name
- Column 16 - % Coverage of reference ARG sequence
- Column 17 - % Identity to reference ARG sequence
- Column 19 - Accession of reference ARG sequence
- Column 20 – Name of reference ARG sequence
