# Supplementary material for: Optimized conditions for Listeria, Salmonella and Escherichia whole genome sequencing using the Illumina iSeq100 platform with point-and-click bioinformatic analysis
Source: PLoS One. 2022 Nov 30;17(11):e0277659. doi: 10.1371/journal.pone.0277659 (PMC9710801; doi:10.1371/journal.pone.0277659)
Supplement: S4 File — (PDF) [file pone.0277659.s004.pdf]

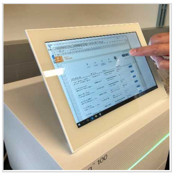

iSeq Bacterial WGS Protocol using a quarter of the reagents

Forked from iSeq Bacterial WGS Protocol

Sonsiray Alvarez Narvaez[1], Zhenyu Shen[2], Lifang Yan[3]

[1]University of Georgia; [2]University of Missouri; [3]Mississippi State University

1 Works for me

Reserved DOI:
10.17504/protocols.io.6qpvr44rbgmk/v1

Sonsiray Alvarez Narvaez

ABSTRACT

This is an adaptation of protocol https://dx.doi.org/10.17504/protocols.io.bij8kcrw in which initial the initial DNA concentration and library prep reagent volumes have been reduced to a quarter.

EXTERNAL LINK

https://doi.org/10.1099/mgen.0.000717

PROTOCOL INFO

Sonsiray Alvarez Narvaez, Zhenyu Shen, Lifang Yan . iSeq Bacterial WGS Protocol using a quarter of the reagents. **protocols.io** https://protocols.io/view/iseq-bacterial-wgs-protocol-using-a-quarter-of-the-ciuwuexe

MANUSCRIPT CITATION please remember to cite the following publication along with this protocol

Alvarez Narvaez S, Shen Z, Yan L, Stenger BLS, Goodman LB, Lim A, Nissly RH, Surendran Nair M, Zhang S, Sanchez S. Optimized conditions for Listeria, Salmonella and Escherichia whole genome sequencing using the Illumina iSeq100 platform with point-and-click bioinformatic analysis. PLOS One

FORK FROM

Forked from iSeq Bacterial WGS Protocol, Sarah Nemser

KEYWORDS

iSeq, Salmonella, Nextera Flex, Illumina DNA Prep, WGS, Vet-LIRN, bacterial WGS, low-throughput WGS, WGS training

CREATED

Nov 04, 2022

LAST MODIFIED

Nov 05, 2022

PROTOCOL INTEGER ID

72310

GUIDELINES

- Wear gloves and change gloves often to avoid cross contamination.
- Consider using separate laboratory coats and disposable sleeve covers for bacterial propagation, DNA extraction, and library preparation.
- Be sure to use clean, aerosol resistant pipette tips for addition of reagents to samples and in between samples to avoid cross-contamination.

MATERIALS TEXT

- Bacterial culture isolates
- Illumina DNA Prep (24 Samples) (20018704)
- Nextera DNA CD Indexes (24 indices in tubes or 96 in *plate format)

*The protocol is written for tube format to accommodate low-throughput application, but plates are more convenient as they have the two indexes mixed together.

- iSeq 100 i1 Reagent (this protocol was developed using the original v1 i1 reagent - the newer version can be used with a software update available from Illumina)
- PhiX Control V3 Kit (FC-110-3001)
- Magnetic stand – various options depending on desired throughput. Magnetic-Ring Stand 96 well (ThermoFisher catalog number: AM10050) or Rack e.g. GE MagRack 6 (VWR product 89129-096).
- Conventional thermocycler and PCR tubes (or plates) which fit the thermocycler
- Qiagen DNeasy Blood & Tissue Kit (cat. no. 69504 or 69506)
- Qiagen RNase A cat. no. 19101 (optional)
- Qiagen Buffer EB cat. no. 19086
- Qubit dsDNA HS Assay Kit for 100 (Invitrogen, Cat. No. Q33230) or 500 (Invitrogen, Cat. No. Q33231) assays
- Qubit dsDNA BR Assay Kit for 100 (Invitrogen, Cat. No. Q32850) or 500 (Invitrogen, Cat. No. Q32853) assays
- Qubit Assay Tubes (500 ct.) for use with the Qubit Fluorometer (Invitrogen, Cat. No. Q32856)
- HyClone HyPure Molecular Grade Water, 100 ml, SH30538.01 (VWR 82007-332) or equivalent
- Absolute Ethanol (200 proof), Molecular Biology Grade, 100 ml (Fisher BP2818500) or equivalent
- Index Replacement Caps, Illumina, #15026762
- Index plate fixture or something similar to hold index tubes (if using tube format)

Optional items for the 96-well format:

- 96-well PCR plate which fits the conventional thermocylcer
- Microseal A (BioRad MSA5001) or equivalent - peelable pressure seal
- Microseal B (BioRad MSB1001) or equivalent - adhesive seal
- Microseal F (BioRad MSF1001) or equivalent - piercable foil seal for freezer storage

See step 24 for a link to an Excel workbook template for calculating input concentrations and generating a sample sheet for the iSeq.

SAFETY WARNINGS

This document does not describe best practices for handling of biological infectious material, which should be determined by the user's institution.

Illumina DNA Prep Library preparation Chemical Safety Warning: Take proper

precautions. Wear appropriate PPE when handling potentially hazardous chemicals. Ensure that chemicals, spent containers, and unused contents are disposed of in accordance with your institutional standards.
TSB: GHS Category 1 for eye damage/irritant and is harmful to aquatic life.
TB1: GHS Category 4 for acute toxicity (dust/mist), Category 2A for eye irritant and Category 1B for reproductive toxicity. Contains N,N-Dimethylformamide.
EPM: GHS Category 4 for acute oral toxicity and Category 1 for specific organ toxicity. Contains tetramethylammonium chloride.

Bacterial propagation and DNA extraction

1 See "guidelines and warnings" for general precautions.

Assemble the following materials and reagents:

A	B	C
**Kit and Reagent**	**Components and notes**	**Volume for 1 Sample**
Qiagen DNeasy Blood & Tissue Kit	Buffer ATL	180 µL
Proteinase K (Qiagen 19131 or 19133)		20 µL – Gram negative bacteria 25 µL – Gram positive bacteria
Buffer AL		200 µL
Buffer ATL	for Gram negative bacteria only	180 µL
Buffer AW1	98mL concentrate+130mL 200 proof ethanol	500 µL
Buffer AW2	66mL concentrate+160mL 200 proof ethanol	500 µL
DNeasy additional collection tubes (Qiagen #19201)	Additional tubes recommended to reduce cross-contamination risk from re-using tubes during wash steps	3 tubes
Qiagen #19086	Buffer EB (for EDTA-free elution of samples)	100 µL
Enzymatic lysis (ELB)-lysozyme mixture for Gram positive isolates only	5mL 1 M Tris-HCl, pH 8.0, 1 mL 0.5M EDTA, 3 mL Triton X-100; add molecular-grade water to bring the volume up to 250 mL; store at RT for 1 year; add 3.6 mg lysozyme right before use	180 µL
Lysozyme- for Gram positive isolates only	(for making ELB)	NA
Triton X-100- for Gram positive isolates only	(for making ELB)	NA
1 M Tris-HCl, pH 8.0- for Gram positive isolates only	(for making ELB)	NA
0.5 M EDTA	(for making ELB)	NA
RNAse A, Qiagen #19101	(for optional RNAse treatment of DNA)	4 µL
200 proof or 96-100% ethanol, Molecular biology grade	NA	200 µL
Water, Molecular biology Grade	For resuspending colonies, preparing 80% EtOH, and dilution of DNA	200 µL
Inoculating loop (1 µL) (BD, #220215)		NA

2 Frozen isolates should be sub-cultured on Trypticase Soy + 5% Sheep Blood Agar plates (BAPs) or equivalent media. If the culture appears pure, pick an isolated colony, and streak it on a fresh BAP; incubate at 35°C overnight (24±2 hours) in aerobic conditions.

3 DNA extraction may be performed either from a colony from the second BAP or from an overnight liquid culture pellet from that colony.

From broth: Transfer 1-1.5 mL of culture to a labelled microcentrifuge tube and centrifuge at 5,000 g for 10 minutes. Remove the supernatant and store the pellet in the tube at -20°C until DNA is extracted.

Direct from colony: (when ready to proceed directly with extraction) from bacterial colonies on plate, fill out a 1 µL loop with overnight growth and add to a labelled microcentrifuge tube containing the appropriate extraction buffer for gram positive or gram negative bacteria.

For **Gram Positive bacteria**, proceed to step 4.

For **Gram Negative bacteria**, proceed to step 9.

4 **Gram Postive Bacteria.**

From broth: Add 180 µL of prepared enzymatic lysis (ELB)-lysozyme mixture to the tube and resuspend the pellet. Proceed to step 5.

From colony: Add overnight growth directly to 180 µL of prepared enzymatic lysis (ELB)-lysozyme mixture to the tube and resuspend the pellet. Proceed to step 5.

5 Incubate at 56°C for 30 minutes, vortexing periodically.

6 Optional: Add 4 µL of RNase A to each tube, vortex and then incubate at RT for 3-5 minutes.

7 Add 25 µL Proteinase K and 200 µL Buffer AL to the tube.

8 Mix thoroughly by vortexing, and incubate at 56°C for 30 minutes. Proceed to step 14.

9 **Gram Negative Bacteria.**

If the ATL buffer solution is cloudy and has precipitate formation, be sure to heat the solution at 50 - 55°C until solution is clear.

From broth: Add 180 µL Buffer ATL to the tube and resuspend the pellet. Add 20 µL Proteinase K to the tube, then vortex. Proceed to step 10.

From colony: Add overnight growth directly to 180 µL Buffer ATL to the tube and resuspend the pellet. Add 20 µL Buffer proteinase K to the tube, then vortex. Proceed to step 10.

10 Incubate at 56°C for a minimum of 1 hour or up to overnight, vortexing periodically.

11 Optional: Add 4 µL of RNase A to each tube, vortex and then incubate at RT for 3-5 minutes.

12 Add 200 µL Buffer AL to the tube.

13 Mix thoroughly by vortexing, and incubate at 56°C for 10 minutes. Proceed to step 14.

14 Add 200 µL ethanol (200 proof) to each tube and mix thoroughly by vortexing followed by a brief pulse spin.

15 Pipet the mixture from step 14 into a labelled DNeasy Mini spin column. Centrifuge at 6,000xg for 1 minute. Discard flow-through and the collection tube.

16 Add 500 µL Buffer AW1, and centrifuge for 1 minute at 6,000xg (8,000rpm). Discard the flow-through and the collection tube.

17 Add 500 µL Buffer AW2, and centrifuge for 1 minute at 6,000xg (8,000rpm). Discard the flow-through and the collection tube.

18 Transfer the spin column to a new collection tube.

19 Centrifuge for 1 minute at 18,000-20,000xg (14,000rpm) to dry.

20 Place the spin column in a clean, labelled 1.5 mL microcentrifuge tube, and pipet 100 µL Buffer EB directly onto the DNeasy membrane.

21 Incubate at room temperature for 1 minute, and then centrifuge for 1 minute at 18,000-20,000xg (14,000rpm) to elute. Record which speed used.

22 Discard spin column and store DNA sample at 4°C for up to 48 hours. For long term storage (>48 hours), store DNA sample at -20°C.

2. DNA quantification and quality check

23 **DNA quantification using the Qubit**.

The following protocol is for 4 samples. If needed, scale the volumes down or up depending on the sample number. The DNA input necessary for this procedure is >100ng. However, an input larger than 500ng is not recommended. Therefore, quantification is a recommended step to ensure proper amount of DNA is added.

24 Download the **iSeq_Prep-Salm-Workbook-public.xlsx** and fill in the isolate identifiers in column B. The blue boxes in the workbook should be filled in while following this protocol.

25 Assemble the following materials and reagents:

A	B	C	D
**Equipment, Kit, and Reagent**	**Components**	**Amount per Sample**	**Amount for 4 Samples + 2 Standards and +1 extra**
Qubit 2.0, 3.0, or 4.0 Fluorometer Invitrogen, #: Q32866	NA	NA	NA
Qubit dsDNA Broad Range (BR) Assay Kit (2-1000ng), #: Q32850	Qubit dsDNA BR Buffer (store at room temperature)	199µL	1393 µL
	Qubit dsDNA BR Reagent (200X) (store at room temperature protected from light)	1 µL	7 µL
	Qubit dsDNA BR Stanard #1 (store at 4°C)	10 µL	10 µL
	Qubit dsDNA BR Standard #2 (store at 4°C)	10 µL	10 µL
Qubit dsDNA High Sensitivity (HS) Assay Kit (0.2-100ng) #: Q32854	Qubit dsDNA HS Buffer (store at room temperature)	199 µL	1393 µL
	Qubit dsDNA HS Reagent (200X) (store at room temperature protected from light)	1 µL	7 µL
	Qubit dsDNA HS Standard #1 (store at 4°C)	10 µL	10 µL
	Qubit dsDNA HS Standard #2 (store at 4°C)	10 µL	10 µL
Qubit assay tubes, #: Q32856	NA	NA	NA
Water, molecular biology grade	For DNA dilution	NA	NA

26 Wear gloves to keep sides of reagent tubes clean and to protect yourself from the reagents. Remove components of Qubit dsDNA BR Assay Kit stored at 4°C and ensure they are at room temperature before use. Be sure to vortex and pulse spin the standards and reagent dye.

27 Prepare the Qubit working solution by diluting 7 µL Qubit dsDNA BR Reagent 200X in 1393 µL Qubit dsDNA BR Buffer in a clean, low-bind plastic tube.

28 Label the tops of Qubit assay tubes to correspond to sample label and BR dsDNA standard label. Do not label the tube sides.

29 Add 190 µL of Qubit working solution to each of the Qubit assay tubes used for Standard #1 and #2.

30 Add 196 µL Qubit working solution to individual Qubit assay tubes used for each sample.

31 Add 10 µL of each Qubit Standard to the appropriate standard tube, and add 4 µL DNA sample to sample tubes, then mix by vortexing. Briefly pulse spin to assure there are no bubbles present. *Note: the input range for the Qubit platform is 1-20 µL, and the volume can be adjusted if desired (as long as the final volume is 200 µL).*

32 Allow all tubes to incubate at room temperature for 2 minutes. Avoid unnecessary handling of tubes as transfer of heat from your hands may lower the reading.

33 On the Home screen of the Qubit 2.0/3.0/4.0 Fluorometer, press DNA, then select dsDNA Broad Range as the assay type. The “Standards” screen is displayed.

34 On the Standards screen, press Yes to read the standards.

35 Insert the tube containing Standard #1 into the sample chamber, close the lid, then press Read. When the reading is complete, remove Standard #1.

36 Insert the tube containing Standard #2 into the sample chamber, close the lid, then press Read. When the reading is complete, remove Standard #2.

37 Select 4 µL for Volume of Sample and insert a sample tube into the sample chamber, close the lid, then press Read. When the reading is complete, record the calculated stock concentration. Then remove the sample tube.

38 Repeat reading until all samples have been read.

39 Record the Qubit readings in the Initial Dilution tab, Column I, DNA Concentration, of the DNA Prep workbook.

40 **DNA quantification and/or quality check using NanoDrop.**

A	B	C	D
**Equipment, Kit, and Reagent**	**Components**	**Amount per Sample**	**Amount for 4 Samples + 2 Standards and +1 extra**
NanoDrop (ThermoFisher)	NA	NA	NA

This step is optional but recommended for first time users.

41 Select Nucleic Acid assay on computer or touchscreen interface of NanoDrop. You may need to do further selection to find the assay specifically for dsDNA.

42 Clean the upper and lower pedestals of the sample loading area with a laboratory wipe.

43 Select Nucleic Acid assay on computer or touchscreen interface of NanoDrop. You may need to do further selection to find the assay specifically for dsDNA.

44 Load 2 µL water on the lower pedestal and close the upper arm gently. Click “Initiate”. Wipe off water on the lower and upper pedestals.

45 Load 2 µL EB on the lower pedestal and close the upper arm gently. Click “Blank”. Wipe off water on the lower and upper pedestals.

46 Load 2 µL of sample on the lower pedestal and close the upper arm gently. Click “Measure”. Record concentration, 260/280, and 260/230. When recording concentration, make sure dsDNA assay is used. Wipe off water on the lower and upper pedestals. Load the next sample for test. It is not necessary to clean with water between sample tests.

47 Record the NanoDrop concentration readings in the Initial Dilution tab, Column H, DNA Concentration, of the DNAFlex workbook.

48 Record the 260/280 and 260/230 ratios in Columns F and G, Initial Dilution tab, of the DNA Flex Workbook. Refer to the Illumina DNA Prep guide for recommended ratios.

49 Clean both upper and lower pedestals of sample loading area with water and laboratory wipes. Close the upper arm.

3. Nextera DNA Flex/Prep Library preparation

50 Assemble the following materials and reagents:

A	B	C
**Kit and Reagent**	**Components**	**Amount for each sample**
Magnet stand, to handle tubes or plates, depending upon sample throughput	NA	NA
Illumina DNA Prep Library Kit, Box 1 (4 degrees storage)	Sample Purification Beads (SPB)	45 µL + 15 µL
	Tagment Stop Buffer (TSB)	10 µL
	Tagment Wash Buffer (TWB) Pipette slowly to minimize foaming	100 µL x 3
Illumina DNA Prep Library Prep Kit, Box 2 (-20 degrees storage)	Tagmentation Buffer 1 (TB1)	10 µL
	Enhanced PCR Mix (EPM)	20 µL
	Resuspension Buffer (RSB)	32 µL
Illumina DNA Prep Library Prep Kit, Box 3 (4 degrees storage)	Bead-Linked Transposomes (BLT) Store the BLT stock tube upright in the refrigerator so that the beads are always submerged in the buffer. Vortex the stock tube thoroughly until the beads are resuspended. It is not recommended to centrifuge the stock tube after vortexing.	10 µL
Ethanol 200 proof for molecular biology (any supplier)	Freshly prepared 80% ethanol	180 µL X 2
i7 and i5 index Nextera DNA CD Indexes (24 indexes, Cat. no. 20018707, tube format) OR in plate format Cat. no. 20018708	i7 indices: H705, H706, H707, H710	5 µL each
	i5 indices: H503, H505, H506, H517	5 µL each
PhiX Control v3, 10nM (Illumina, FC-110-3001)	NA	NA
Ice	NA	NA
DNA	NA	NA

Preparation of Sample Sheet.

51

The steps for Sample Plate and Sample Sheet setup in the Illumina Experiment Manager on the instrument can be completed at any point in this protocol but must be performed prior to the start of the run. However, it is recommended to create the Sample Plate and Sample Sheet at the beginning of library prep.

52 Depending upon desired sample throughput, the samples can be prepped using individual tubes with a magnetic stand device equipped to handle tubes. Alternatively, samples can be prepped using a 96-well format with a magnetic device that handle plates. A 96-well format lends itself for using a multi-channel pipette.

53 Prepare the "Initial dilution" tab from the "DNA Flex Workbook".

NOTE: *The workbook is designed with the following color scheme, in general, the blue colored fields should be filled in.*

54 Confirm or adjust the i7 and i5 indexes listed in Columns D and E. If using indexes purchased in the 96-well plate format, choose positions on the Nextera CD indexes map and note the wells used.

55 Check that the DNA input (ng) is ~75 ng in **Column J**. If it is below this range, adjust the volume multiplier (change the 2 to a higher number) in the **Column J formula** so that it falls in this range. If the original concentration falls above range (DNA is too concentrated), perform a dilution, re-check the concentration, and update row I.

NOTE: *We adjusted the concentration of the DNA input to 10ng/µL and we used 7.5 µL.*

56 Add enough nuclease-free water calculated in **Column L** of the "DNA Flex Workbook" to each sample in a clean PCR tube.

57 Add volume of DNA adjusted in **Column K** of the "DNA Flex Workbook" to the sample tubes now containing nuclease-free water.

58 Mix by pitetting 5-10 times. Close tubes (or seal plate) and store at 4-8º C overnight, or proceed to step 59.

59 **Tagment Genomic DNA.**

Remove the SPB, TSB, and TWB from Box 1 and bring to room temperature. The TSB and TWB can then be stored at room temperature. Remove one tube of BLT from Box 3 and bring to room temperature. Thaw the RSB, bring TB1 (from freezer) to room temperature.

60 Vortex BLT vigorously for 10 seconds, visually check the beads for complete resuspension and repeat as necessary. Vortex the TB1.

61 Prepare the tagmentation master mix according to the following table:

A	B	C
**Reagent**	**Volume per Sample**	**For 4 Samples**
TB1	2.5 µL	10 µL
BLT	2.5 µL	10 µL

62 Vortex the Tagmentation master mix thoroughly to make sure the BLT beads are evenly resuspended in the buffer.

63 Using fresh tips, transfer 5 µL of Tagmentation master mix to each well containing a sample.

64 Pipette up and down 10 times mixing the 12.5 µL reaction to resuspend the beads.

65 Close the PCR tube (or seal the plate with Microseal 'A' or equivalent if using a 96-well format).

66 Place the PCR tubes into the thermalcycler and run the tagmentation program FLEXTAG. If the thermalcycler does not have the program, create one using the following conditions with a lid pre-heated.

Incubation Conditions: (12.5 µL total reaction)
1. 55°C for 15 minutes
2. Hold at 10°C

Upon completion of the incubation, remove the PCR tubes from the thermalcycler and proceed to **Post Tagmentation Cleanup** steps.

67 **Post Tagmentation Cleanup.**

Check for precipitates in the TSB. If present, heat the buffer at 37°C for 10 minutes and vortex until dissolved.

68 Open the PCR tubes (or remove the seal from plate).

69 Add 2.5 µL of TSB to the tagmentation reaction.

70 Gently pipette up and down 10 times, mixing the entire volume to resuspend the beads.

71 Close the PCR tubes (or seal the plate with Microseal 'A' or equivalent).

72 Place the PCR tubes into the thermalcycler and run the program FLEXPOST. If the thermalcycler does not have the program, create one using the following with a lid pre-heated.

Incubation Conditions: (now 15 µL total reaction)
1. 37°C for 15 minutes
2. Hold at 10°C

73 Remove the EPM and indices from freezer and allow to come to room temperature while this incubation is running to prepare for later steps.

74 Remove the PCR tubes from the thermalcycler, quick spin down, then open the tubes (or remove the seal).

75 Place on the magnet for 3 minutes or until solution is clear.

76 Use a multichannel pipette, remove the supernatant and discard.

77 Remove the tubes from the magnet and add 100 µL of TWB directly to the beads. Gently pipette to mix until beads are fully resuspended.

NOTE: *It is challenging to resupend the pellet at this series of washes, check each well to ensure sufficient mixing of beads.*

78 Place the tubes on the magnet for 3 minutes or until solution is clear.

79 Remove the supernatant and discard.

80 Repeat steps 77 - 79 one more time for a total of two washes.

81 Remove the tubes from the magnet and add 100 µL of TWB directly to the beads. Gently pipette to mix until beads are fully resuspended.

82 Place the tubes with TWB on the magnet and allow to incubate until ready to proceed with adding the PCR master mix in the **Amplify Tagmented DNA** step. The tubes should incubate for at least 3 minutes. It is important to keep the pellet in TWB to prevent over drying of the beads.

83 **Amplify Tagemented DNA.**

Invert the EPM to mix.

84 Briefly spin the Index tubes.

85 Prepare the PCR master mix according to the following table:

A	B	C
**Reagent**	**Volume per Sample**	**For 4 Samples**
EPM	5 µL	20 µL
Molecular grade water	5 µL	20 µL

86 Vortex and spin down the PCR master mix.

87 Remove the third TWB wash from the samples while on the magnet. Remove any excess liquid from the tubes.

88 Remove the tubes from the magnet and immediately proceed to adding the master mix.

89 Add 10 µL of the PCR master mix to each sample well. Gently pipette to mix to ensure beads are resuspended.

90 Open the i7 primer tubes with orange caps and discard the caps. Add 1.25 µL of index primers to the NTA tube. After completion, use orange replacement caps to close the primer tubes.

91 Open the i5 primer tubes with white caps and discard the caps. Add 1.25 µL of index primers to the NTA tube. After completion, use the white replacement caps to close the primer tubes.

92 Use a pipette to mix a minimum of 10 times at a higher volume (30 µL) to ensure throrough mixing.

NOTE: *Be careful to avoid introducing air bubbles.*

93 Close the tubes (or seal the plate with Microseal 'A' or equivalent).

94 Place the tubes into the thermalcycler and run the following program (FLEXPCR):

PCR Conditions: (12.5 µL total reaction)

1. 68°C for 3 minutes
2. 98°C for 3 minutes
3. 98°C for 45 seconds
4. 62°C for 30 seconds
5. 68°C for 2 minutes
6. Repeat steps 3-5 an additional 4 times for a total of 5 cycles
7. 68°C for 1 minute
8. Hold at 10°C

95 Remove the tubes from the thermal cycler.

96 **This is a safe stopping point**. Store at 4°C for up to 3 days, if you decide to store the tubes (or with plates, replace the Microseal 'A' with a Microseal 'B'). If ready to **Clean Up Libraries**, proceed to the next step.

97 Remove the SPB from the refrigerator to equilibrate to room temperature if proceeding to **Clean Up Libraries** step.

98 **Clean Up Libraries.** (The library fragments are free of the BLT at this point).

Before starting, prepare reagents. Dilute fresh 80% ethanol sufficient for all samples:

Reagent	Volme per Sample	For 4 Samples
100% Ethanol	0.4 ml	1.6 ml
Molecular grade water	0.1 ml	0.4 ml

99 Be sure the SPB is at room temperature (at least 30 minutes from refrigerator).

100 Bring RSB to room temperature (from the freezer) and vortex to mix.

101 Vortex and invert SPB several times to fully re-suspend the beads.

102 Prepare SPB master mix:

A	B	C
**Reagent**	**Volume per Sample**	**For 4 Samples**
SPB	11.25 µL	45 µL
Molecular grad water	10 µL	40 µL

NOTE: *It is recommended to increase the number of samples 3-4 to ensure sufficient volume of master mix.*

103 If PCR tubes (or plate) were retrieved from cold storage, centrifuge at 280 x g for 1 minute.

104 Remove the seal.

105 Place the tubes on the magnet for 3-5 minutes (until supernatant is clear).

106 Transfer 10 µL of the ***PCR supernatant (now containing DNA)*** to clean tubes (or a different location on the sample plate or into a fresh 96-well plate).

107 Remove the tubes from magnet and discard.

108 Vortex and then add 21.25 µL of the SPB master mix prepared in step 102, to each well. **Be sure to not carry over extra SPB master mix on the outside of the tips. Wipe the outside of the tips on the reservoir to remove droplets before transferring the SPB master mix to the PCR supernatant.**

109 Pipette to mix a minimum of 10 times until thoroughly mixed (use caution as volume is > 100 µL).

110 Incubate at room temperature for 5 minutes.

111 Place tubes on the magnet for 5 minutes or until supernatant is clear.

112 During the incubation, vortex the SPB stock tube thoroughly, and add 3.75 µL to new tubes (one for each sample) (or on the same plate or in a new 96-well plate). **Be sure to remove excess SPB from the outside of tips before addition to new tubes.**

113 Transfer 31.25 µL of ***supernatant*** to the wells containing the 3.75 µLSPB (stock concentration).

114 Gently pipette to mix 10 times at a volume of ~85 µL, avoiding the introduction of air bubbles.

115 Incubate at room temperature for 5 minutes.

116 Place the tubes on the magnet for 5 minutes or until clear.

117 Remove and discard the supernatant without disrupting the beads.

118 With the tubes on the magnet, add 180 µL of freshly prepared 80% ethanol and incubate for 30 seconds.

119 Pipette to remove the ethanol supernatant.

120 Repeat steps 118 and 119 for a total of two washes.

121 Use a pipette to remove any excess liquid from tubes.

122 Leave beads to air dry on the magnet for 5 minutes.

123 Remove the tubes from the magnet and add 32 µLof RSB to the beads.

124 Pipette to mix until thoroughly resuspended.

125 Incubate at room temperature for 2-5 minutes.

126 Place the tubes onto the magnet for 3 minutes until clear.

127 Transfer 30 µL of the supernatant into a new tube for each sample (or new well on a 96-well plate for each sample).

If ready, continue to the **Pooling Libraries** step. Otherwise, this is a safe stopping point.

128 Close the tubes (or seal the plate with a freezer-safe seal) and store at -20°C for up to 30 days.

129 **Pooling of Libraries and dilution of the pool to 1 nM concentration.**

Perform quantitation on each of the individual libaries. Qubit HS assay is recommended by Illumina, but a NanoDrop can be used if this is the only instrument available. Enter the results into **Column B** on the "Normalization and Pooling" tab in the "DNA Flex Workbook".

NOTE: *This procedure assumes that all input DNA is >100 ng and therefore would yield a stable library. Nextera DNA Flex library preparation method produces normalized libraries and does not require a dilution-based normalization step.*

130 Pool 5 µL per sample into a single 1.5ml tube. Optional: for smaller bacterial genomes, reduce the input volume to 2.5 µL.

131 Mix by vortexing then spin down.

132 Quantify the pooled library using the Qubit HS assay or NanoDrop. Record result at the bottom of the “Normalization and Pooling” tab in the “DNA Flex Workbook”.

133 This SOP uses the CFSAN suggested conversion factor of 2.5 which assumes an average fragment size of roughly 600 bp. The “DNA Flex Workbook” will convert the final pooled library input to nM using this conversion factor.

134 The “DNA Flex Workbook” will then calculate the values necessary to dilute your library to a concentration of 4 nM in a volume of 15 µL (C1V1 = C2V2).

135 Dilute the final library to 4 nM according to the values on the workbook using RSB.

136 Add 5 µl of the 4 nM pool to 15 µL RSB to a new low bind microtube to make 1 nM pool.

137 Vortex briefly, and then quick-spin at 280 x g for 3 sec.

138 Set aside 1 nM library on ice or this is a safe stopping point. The 1 nM library can be stored at -20ºC for up to 1 month. Stopping at this point will also allow for thawing.

139 **Preparation of Cartridge.**

Wear powder-free gloves.Thaw by following one of three ways listed below. Prior to thawing, remove the cartridge from the box but do not open the white foil bag! After thawing, do not refreeze. Store cartridge at -15˚C to -25˚C at least one day before thawing using any of the methods listed below.

Method	Thaw time	Instruction
20˚C to 25˚C water bath	6 hours, not exceeding 18 hours	Use 6 L water per cartridge. Set a temperature-controlled water bath to 25˚C or mix hot and cold water to achieve 20˚C to 25˚C. Face the bag label up, submerge the cartridge completely, and apply ~2 kg weight to prevent floating. Do not stack cartridges in the water bath unless it is temperature-controlled.
2˚C to 8˚C refrigerator	36 hours, not exceeding 1 week	Position the cartridge so that the label faces up and air can circulate on all sides, including the bottom.
Room-temperature air (20˚C to 25˚C)	9 hours, not exceeding 18 hours	Position the cartridge so that the label faces up and air can circulate on all sides, including the bottom.

If cartridge is wet from a water bath, dry with paper towels.

140 **Dilute Pool to 200 pM Loading Concentration for iSeq.**

Add 20 µL of 1 nM pool to 80 µl of RSB to a new low bind microtube to produce 100 µL of 200 pM concentration.

141 Vortex briefly, and then centrifuge at 280 x g for 3 sec.

142 **Spiking of Library with PhiX.**

Add 2 µL of 10 nM PhiX to 18 µL of RSB in a new low bind microtube to make 1 nM PhiX. PhiX can be safely stored at -20°C for up to one month at this concentration.

143 Vortex briefly, and then centrifuge at 280 x g for 3 sec. Set aside on ice.

144 Further dilute the PhiX in a low bind microtube to 200 pM by adding 20 µL of 1 nM PhiX to 80 µL of RSB.

145 Vortex briefly, and then centrifuge at 280 x g for 3 sec.

146 Add 2 µL of 200 pM PhiX to the tube that contains 100 µL of 200 pM pooled library. (These volumes result in a ~2% PhiX spike-in. Actual percentage varies depending on library quality and quantity).

147 Set aside the library with PhiX spike-in on ice. This is the final loading product.

iSeq loading and set up

148 **Load the Flow Cell.**

Avoiding the access window on top of the cartridge, remove the cartridge from the bag. Discard the bag.

149 Invert the cartridge five times to mix reagents. Internal components can rattle during inversion, which is normal.

150 Tap the cartridge (label facing up) on the bench or other hard surface five times to ensure reagent aspiration.

151 Using a new pipette tip, pierce the Library reservoir and push the foil to the edges to enlarge the hole. Discard the pipette tip.

152 Add 20 µL diluted library to the ***bottom*** of the reservoir. Avoid touching the foil.

153 Open the white foil package containing the flow cell from the notches. Use within 24 hours of opening.

154 Carefully pull the flow cell out of the package. Touch only the plastic when handling the flow cell. Avoid touching the electrical interface, CMOS sensor, glass, and gaskets on either side of the glass. See the referenced iSeq guide for an illustration.

155 Hold the flow cell by the grip points with the label facing up.

156 Insert the flow cell into the slot on the front of the cartridge. An audible click indicates that the flow cell is in place. When properly loaded, the grip protrudes from the cartridge and the glass is visible from the access window.

157 **Set Up a Sequencing Run.**

From the control software menu, select **Local Run Manager**, and then select **Open Local Run Manager**.

158 Select **+ Create Run**.

159 From the three available options, select **Generate FASTQ**.

160 Enter **Run Name** in this format: YYMMDD-Vet-LIRN-LAB ["LAB" is your two-letter state abbreviation listed in Appendix B.] [Example, for a run starting on November 27, 2019 the run name would be entered as 191127-Vet-LIRN-NY].

161 Enter **Run Description** (e.g. "Practice Samples").

162 Under **Run Settings**, select the appropriate **Library Prep Kit** (Nextera DNA CD Indexes-24 index tube.)

163 **Index Reads**, select the box next to **"2."**

164 **Read Type**, select **Paired End**.

165 **Read Lengths**, be sure that 151 has been entered for **Read 1**.

166 **Read Lengths**, be sure that 151 has been entered for **Read 2**.

167 Under **Module-Specific Settings**, be sure the **Adapter Trimming** option **"On"** is selected.

168 For each sample being sequenced, enter the **Sample ID**, **Sample Description**, and **Sample Project. Do not modify the Sample ID, Sample Description, and Sample Project. It must be entered exactly as given below.**

The **Sample ID** is the same as the Isolate ID listed in Column B of the Initial Dilution Tab of the Flex workbook.

Sample Description is the same as the Organism in Column C of the Initial Dilution Tab of the Flex workbook. If labeling with genus and species a "-" must be included between the Genus and Species because the sequencer software does not recognize spaces.

For the Vet-LIRN iSeq study: The **Sample Project** name is: VL-iSeq-LAB, where "LAB" is your two-letter state abbreviation. Do not deviate from this naming format.

169 Under **INDEX plate location using the dropdown**.

170 Save the run.

171 In the control software, select **Sequence** from the Home screen. The software opens the door at an angle, ejects the tray, and initiates the series of run setup screens.

172 **Load the Cartridge onto the Instrument.**

Place the cartridge onto the tray so that the access window faces up and the flow cell is inside the instrument. Do not push the cartridge or tray into the instrument.

173 Select **Close Door** to retract the cartridge and close the door. A panel appears on the left side of the screen to show information from the scanned consumables. During scanning, you can continue with run setup.

174 **Sign in to BaseSpace Sequence Hub.**

Sign in to your BaseSpace Sequence Hub by entering your email address and password, and then select **Sign In**.

175 Under configurations, **make sure that "run monitoring and storage" has been checked.**

176 Select **Run Setup**.

177 **Select a Run and Review Pre-Run Checks.**

Select the run you saved (from step 170 above) from the **Run Name** list.

178 Select **Start Run** to start the pre-run check.

179 Wait about 15 minutes for pre-run checks to complete. The run will start automatically after successful completion. Do not open the door during the pre-run check or during the run because it can cause run failure.

180 If an error occurs during the instrument check, select **Retry** to redo the check.

181 **Unload Consumables.**

When sequencing is complete, select ***Eject Cartridge.*** The software ejects the used cartridge from the instrument.

182 Remove the cartridge from the tray.

183 Remove the flow cell from the cartridge.

184 Dispose of the flow cell, which has electronic components, in accordance with applicable standards for your institution.

185 Dispose of the cartridge, which contains used reagents, in accordance with applicable standards for your institution.

186 Select ***Close Door*** to reload the tray and return to the Home screen. The software automatically reloads the tray and sensors confirm the cartridge removal.

187 **Share the Run.**

Sign in to BaseSpace on a web browser and check that the run is complete.

188 Click on the run name. Select “Share”, enter the email of the person you would like to share with, and make sure that the check box for sharing the project remains clicked.

Analysis

189 Assessment of sequence quality can be performed using the following protocol: dx.doi.org/10.17504/protocols.io.bdvfi63n

References

190 Qiagen DNeasy Blood & Tissue Handbook. HB-2061-003 07/2020 https://www.qiagen.com/us/resources/resourcedetail?id=68f29296-5a9f-40fa-8b3d-1c148d0b3030&lang=en

191 Illumina DNA Prep documentation https://support.illumina.com/sequencing/sequencing_kits/illumina-dna-prep/documentation.html

192 Illumina iSeq 100 Sequencing System Guide https://support.illumina.com/downloads/iseq-100-system-guide-1000000036024.html

193 Illumina Index Pooling Guide https://support-docs.illumina.com/SHARE/IndexAdapterPooling/Content/SHARE/IndexAdapterPooling/Nextera/Pooling_fNXT.htm
